# Three-year patient-reported outcomes of the BOOG 2013-08 RCT evaluating omission of sentinel lymph node biopsy in early-stage breast cancer patients treated with breast conserving surgery: Impact of personality traits on health-related quality of life

**DOI:** 10.1093/bjs/znaf031

**Published:** 2025-05-14

**Authors:** Veerle M Wintraecken, Lori M van Roozendaal, Janine M Simons, Jolanda de Vries, Sander M J van Kuijk, Marissa L G Vane, Thijs van Dalen, Helena Sackey, Jos A van der Hage, Luc J A Strobbe, Sabine C Linn, Marc B I Lobbes, Philip M P Poortmans, Vivianne C G Tjan-Heijnen, Koen K B T van de Vijver, Helen H Westenberg, Carmen D Dirksen, Johan H W de Wilt, Liesbeth J Boersma, Marjolein L Smidt

**Affiliations:** GROW—Research Institute for Oncology and Reproduction, Maastricht University, Maastricht, The Netherlands; Department of Surgery, Maastricht University Medical Centre+, Maastricht, The Netherlands; Department of Surgical Oncology, Zuyderland Medical Centre, Sittard-Geleen, The Netherlands; GROW—Research Institute for Oncology and Reproduction, Maastricht University, Maastricht, The Netherlands; Department of Radiotherapy, Erasmus MC Cancer Institute, University Medical Center Rotterdam, Rotterdam, The Netherlands; Department of Medical and Clinical Psychology, Tilburg University, Tilburg, The Netherlands; Board member Admiraal de Ruyter Ziekenhuis, Goes, The Netherlands; Department of Clinical Epidemiology and Medical Technology Assessment, Maastricht University Medical Centre+, Maastricht, The Netherlands; GROW—Research Institute for Oncology and Reproduction, Maastricht University, Maastricht, The Netherlands; Department of Surgery, Maastricht University Medical Centre+, Maastricht, The Netherlands; Division of Surgical Oncology, Diakonessenhuis Hospital, Utrecht, The Netherlands; Department of Surgery, Erasmus Medical Centre, Rotterdam, The Netherlands; Department of Molecular Medicine and Surgery, Karolinska Institutet, Stockholm, Sweden; Department of Breast- Endocrine Tumors and Sarcoma, Karolinska University Hospital, Stockholm, Sweden; Division of Surgical Oncology, Leids University Medical Centre, Leiden, The Netherlands; Division of Surgical Oncology, Canisius-Wilhelmina Hospital, Nijmegen, The Netherlands; Division of Medical Oncology, Netherlands Cancer Institute—Antoni van Leeuwenhoek Hospital, Amsterdam, the Netherlands; Department of Medical Imaging, Zuyderland Medical Centre, Sittard-Geleen, The Netherlands; Department of Radiation Oncology, Iridium Network, Antwerp, Belgium; Faculty of Medicine and Health Sciences, University of Antwerp, Antwerp, Belgium; GROW—Research Institute for Oncology and Reproduction, Maastricht University, Maastricht, The Netherlands; Division of Medical Oncology, Maastricht University Medical Centre+, Maastricht, The Netherlands; Department of Pathology, Ghent University Hospital, Ghent, Belgium; Department of Diagnostic Sciences, Cancer Research Institute Ghent (CRIG), Ghent University, Ghent, Belgium; Centre for Gynaecological Oncology Amsterdam (CGOA), Department of Gynaecology, Netherlands Cancer Institute—Antoni van Leeuwenhoek Hospital, Amsterdam, The Netherlands; Radiation Oncology, Radiotherapiegroep location Arnhem, Arnhem, The Netherlands; Department of Clinical Epidemiology and Medical Technology Assessment, Maastricht University Medical Centre+, Maastricht, The Netherlands; Care and Public Health Research Institute (CAPHRI), Maastricht University, Maastricht, The Netherlands; Division of Surgical Oncology, Radboud University Medical Centre, Nijmegen, The Netherlands; Department of Radiation Oncology (Maastro), GROW—Research Institute for Oncology and Reproduction, Maastricht University Medical Centre+, Maastricht, The Netherlands; GROW—Research Institute for Oncology and Reproduction, Maastricht University, Maastricht, The Netherlands; Department of Surgery, Maastricht University Medical Centre+, Maastricht, The Netherlands

## Abstract

**Background:**

The non-inferiority randomized controlled trial BOOG 2013-08 investigates the oncological safety and impact on health-related quality of life (HRQoL) of sentinel lymph node biopsy (SNLB) omission in cT1–2 N0 breast cancer. The primary aim of the present study was to compare patient-reported arm function and HRQoL up to 3 years after study inclusion in cT1–2 N0 patients with breast cancer undergoing breast-conserving surgery (BCS) with or without SLNB. The secondary aim was to explore the association between personality traits ‘trait anxiety’ and ‘neuroticism’, and perceived arm function and HRQoL.

**Methods:**

A total of 1733 women with unilateral cT1–2 N0 invasive breast cancer treated with BCS with or without SLNB were included. The primary outcomes of arm function (assessed using the Lymphoedema Functioning, Disability, and Health Questionnaire) and HRQoL (assessed using the European Organisation for Research and Treatment of Cancer QLQ-C30 and QLQ-BR-23 questionnaires) were analysed.

**Results:**

Analyses included 821 patients (383 with SLNB and 438 without SLNB). Those in the SLNB group experienced a slight, temporary decline in arm function (*P* < 0.025) and reported more HRQoL arm and breast symptoms (*P* < 0.049). High trait anxiety or neuroticism was associated with significant poorer arm function and lower HRQoL.

**Conclusion:**

SLNB slightly reduced arm function, temporarily affecting HRQoL arm and breast symptoms. Neuroticism significantly negatively impacted arm function and HRQoL. Measuring and stratifying for personality traits is crucial for interpreting patient-reported outcomes and to identify patients needing additional support after surgery.

**Registration number:**

NCT02271828 (http://www.clinicaltrials.gov).

## Teaser

This paper provides distinctive 3-year follow-up data from 821 patients concerning patient-reported arm function and health-related quality of life between patients who underwent sentinel lymph node biopsy and those who did not. Sentinel lymph node biopsy slightly reduced arm function, temporarily affecting health-related quality-of-life arm and breast symptoms. The personality trait neuroticism significantly negatively impacted arm function and health-related quality of life, more so than clinical factors such as sentinel lymph node biopsy.

## Introduction

Sentinel lymph node biopsy (SLNB) is the standard nodal staging method in clinically node-negative (cN0) breast cancer patients^1^. Landmark studies have shown that SLNB, when compared with axillary lymph node dissection (ALND), provides comparable survival and regional control in cN0 breast cancer patients, even in the case of limited metastasis in sentinel lymph nodes^2–7^. In addition, while arm morbidity is reduced when omitting ALND^5,8–13^, studies report heterogeneous outcomes regarding the impact on overall health-related quality of life (HRQoL)^4,9,12–14^. Approximately 25% of patients experience short-term arm morbidity even after SLNB and up to 8% suffer from lifelong lymphoedema, which can affect daily life, such as causing difficulties in returning to work and decreased participation in social and physical activities^7–9,15–19^.

Current RCTs, including the BOOG 2013-08 trial, assess the safety of omitting SLNB in cT1–2 N0 breast cancer patients treated with breast-conserving surgery (BCS)^20–22^. In addition to long-term oncological endpoints, the BOOG 2013-08 trial assesses patient-reported arm function and HRQoL over time between patients having an SLNB and those not undergoing an SNLB. It was hypothesized that patients without SLNB would experience better arm function and higher physical HRQoL.

Patient-reported outcomes are crucial for determining the most optimal treatment. Clinical characteristics, such as disease stage and treatment, and patient characteristics, such as age and relationship status, are well-established factors influencing patient-reported outcomes. Numerous studies have further demonstrated that personality traits can positively or negatively affect HRQoL^23–26^. There is a particularly strong negative association between HRQoL and the personality traits ‘trait anxiety’ and ‘neuroticism’^25–27^. Therefore, it was hypothesized that patients with high trait anxiety and/or neuroticism levels would experience reduced arm function and lower HRQoL after BCS with or without SLNB^23–26^.

The primary aim of the present study was to compare patient-reported arm function and HRQoL scores up to 3 years after study inclusion in cT1–2 N0 breast cancer patients undergoing BCS with or without SLNB, followed by adjuvant whole breast radiotherapy (BCT) in the BOOG 2013-08 trial. The secondary aim was to assess the impact of the personality traits ‘trait anxiety’ and ‘neuroticism’ on arm function and HRQoL.

## Methods

### Study design and patients

The present study used data from the BOOG 2013-08 trial (NCT02271828), a Dutch multicentre non-inferiority RCT assessing whether omitting SLNB in cT1–2 N0 breast cancer patients treated with BCS is non-inferior compared with standard treatment with SLNB in terms of regional recurrence rate^20^. The trial was approved by the medical ethics committee of the Netherlands Cancer Institute-Antoni van Leeuwenhoek (NL49315.031.14/M14CNB). A total of 1733 women aged greater than or equal to 18 years with unilateral cT1–2 N0 invasive breast cancer were included between May 2015 and January 2022. Written informed consent was obtained from all patients.

Primary endpoint results regarding regional recurrence are expected in 2025. The present study focuses on the secondary endpoints arm function and HRQoL. The sample size estimate for secondary outcomes, including HRQoL, required a total of 1056 patients. Therefore, only the first 1056 patients that were enrolled in the BOOG 2013-08 trial were asked to complete questionnaires on arm function, HRQoL, and personality traits.

### Treatment

Patients were randomized between BCT with SLNB, or BCT without SLNB. If a sentinel lymph node metastasis was found, the decision for completion axillary treatment (that is completion ALND, axillary radiation therapy (RT), or no further axillary treatment) was discussed in local multidisciplinary meetings and determined by factors such as metastatic load and the presence of risk factors^28^.

### Data collection

Clinical data were collected from patients’ medical records by specially trained registration clerks of the Netherlands Comprehensive Cancer Organization (IKNL) and included demographical characteristics, tumour characteristics, surgical procedures, (neo)adjuvant systemic treatment, and RT details.

For patient-reported outcomes, arm function was assessed using the Lymphoedema Functioning, Disability, and Health questionnaire (Lymph-ICF)^29^. This is a validated breast-cancer specific questionnaire in Dutch that assesses arm-related impairments in function, activity limitations, and participation restrictions related to lymphoedema across five domains (physical function, mental function, household activities, mobility activities, and life and social activities)^29^. Higher scores represent reduced arm function^30^. To determine whether differences in mean scores between treatment groups were clinically relevant, minimal clinically important differences (MCIDs) of 9% for total score, 14% for physical function, 7% for mental function, 8% for household activities, 6% for mobility activities, and 5% for life and social activities, were used to indicate clinically relevant decreases in arm function^30^.

HRQoL was assessed using the European Organisation for Research and Treatment of Cancer (EORTC) QLQ-C30 and QLQ-BR-23 questionnaires (a quality-of-life questionnaire and a breast cancer module respectively)^31–33^. These validated questionnaires consist of functioning scales and symptom scales. A higher score represents better/healthier functioning and global HRQoL for a functioning scale or more symptoms/problems for the symptom scales. Similar to the INSEMA trial, an MCID of five points was used as a threshold for clinically relevant differences in HRQoL outcomes^34^.

The personality trait ‘trait anxiety’ was measured using the validated Spielberger State-Trait Anxiety Inventory (STAI-trait) and the personality trait ‘neuroticism’ was measured using the validated NEO Five-Factor Inventory (NEO-FFI)^35–37^. In accordance with the scoring manuals, an STAI-trait score of greater than or equal to 22 and an NEO-FFI score of greater than or equal to 38 were considered high. Based on these scores, patients were categorized into three groups: those with low trait anxiety and low neuroticism (low STAI-trait and NEO-FFI scores); those with high trait anxiety (high STAI-trait score and low NEO-FFI score); and those with high neuroticism (high NEO-FFI score and high/low STAI-trait score).

Patients were asked to complete all five questionnaires at baseline and subsequently at 6 months and 1, 2, and 3 years after study inclusion. Patients were included in the analysis when at least two questionnaires were completed, with the baseline questionnaire being mandatory.

### Statistical analysis

Baseline characteristics were stratified according to treatment allocation. Statistical analyses were performed according to the as-treated principle.

Missing HRQoL data were handled in accordance with the EORTC scoring manual^33^. Multiple imputation was used to deal with any remaining missing data. Categorical outcomes were compared between treatment groups using Pearson’s chi-squared test and continuous outcomes were compared between treatment groups using the independent-sample *t* test or the Kruskal–Wallis test. Linear mixed-effect models for repeated measures were used to compare total Lymph-ICF and global HRQoL scores over time between treatment groups, adjusted for potential confounders (personality group, age, treatment hospital, clinical and pathological tumour stage, co-morbidities, (neo)adjuvant treatment, marital and parenthood status, educational level, employment status, BMI, and smoking status). Variables were entered as fixed effects, except for treatment hospital, which was entered as a random effect. Backward elimination using Akaike information criterion (AIC) was used to select the best-fitting model to describe the relationship between HRQoL and personality.

Statistical tests were two-sided and *P* ≤ 0.050 was considered statistically significant. Data were analysed using SPSS^®^ (IBM, Armonk, NY, USA; version 28).

## Results

**Fig. 1:**
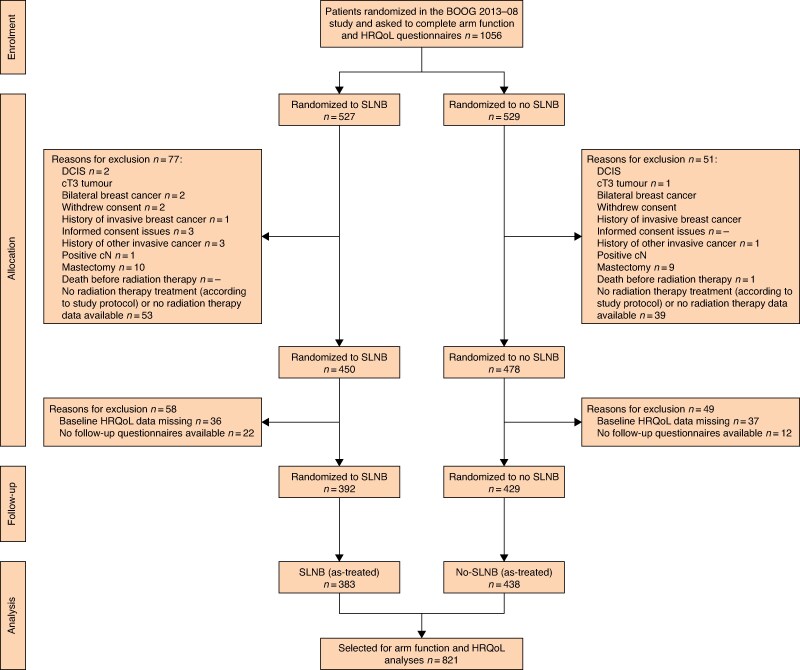
Flow chart HRQoL, health-related quality of life; DCIS, ductal carcinoma in situ; SLNB, sentinel lymph node biopsy.

A total of 1056 BOOG 2013-08 participants were asked to complete questionnaires. Response rates were 92.6% at baseline and 91.3% at 6 months, 86.7% at 1 year, 78.3% at 2 years, and 62.7% at 3 years after study inclusion. Due to BOOG 2013-08 trial protocol deviations, 128 of 1056 patients (12.1%) were excluded from further analyses (see *Fig. 1* for the CONSORT flow chart)^38^. Additionally, 107 patients (11.5%) were excluded due to missing baseline questionnaires and/or at least one follow-up questionnaire. After these exclusions, a total of 821 patients were included (392 patients in the SLNB group and 429 patients in the no-SLNB group). Baseline tumour and patient characteristics are summarized in *Table 1* and *Table 2*.

**Table 1:** Baseline clinicopathological characteristics of BOOG 2013-08 participants selected for arm function and health-related quality-of-life analysis according to intention-to-treat analysis

Characteristic	Overall (*n* = 821)	SLNB group (*n* = 392)	No-SLNB group (*n* = 429)
**cT stage**			
cT1	668 (81.4)	317 (80.9)	351 (81.8)
cT2	153 (18.6)	75 (19.1)	78 (18.2)
**Histological subtype**			
Invasive ductal carcinoma/invasive carcinoma no special type	635 (77.8)	304 (78.1)	331 (77.5)
Invasive lobular carcinoma	98 (12.0)	45 (11.6)	53 (12.4)
Other	83 (10.2)	40 (10.3)	43 (10.1)
Missing, *n*	5	3	2
**Pathological grade (Bloom Richardson)**			
I	239 (29.4)	124 (32.0)	115 (27.0)
II	426 (52.4)	192 (49.0)	234 (54.9)
III	148 (18.2)	71 (18.3)	77 (18.1)
Missing, *n*	8	5	3
**Hormone receptor status**			
ER+ and HER2+	58 (7.1)	26 (6.7)	32 (7.5)
ER− and HER2+	17 (2.1)	9 (2.3)	8 (1.9)
ER+ and HER2−	686 (84.1)	328 (84.3)	358 (83.8)
Triple negative	55 (6.7)	26 (6.7)	29 (6.8)
Missing, *n*	3	3	0
**Neoadjuvant therapy**			
None	733 (89.3)	351 (89.5)	382 (89.0)
Chemotherapy	40 (4.9)	15 (3.8)	25 (5.8)
Immunotherapy or targeted therapy	0	0	0
Hormonal therapy	16 (1.9)	11 (2.8)	5 (1.2)
Chemotherapy, as well as immunotherapy or targeted therapy	32 (3.9)	15 (3.8)	17 (4.0)
**pN stage**			
pN0	307 (37.4)	307 (80.9)	0
pN0(+i)	17 (2.1)	17 (4.5)	0
pN1mi	18 (2.2)	18 (4.7)	0
pN1	27 (3.3)	26 (6.8)	1 (0.2)*
pN2	0	0	0
pNX	452 (55.1)	24 (3.1)	428 (99.8)
**Additional axillary treatment**			
ALND only	3 (0.4)	3 (0.8)	0
Regional RT only	37 (4.5)	35 (9.0)	2 (0.5)
ALND and regional RT	3 (0.4)	3 (0.8)	0
**Adjuvant therapy**			
None	438 (53.3)	218 (55.6)	220 (51.3)
Chemotherapy	22 (2.7)	10 (2.6)	12 (2.8)
Immunotherapy or targeted therapy	6 (0.7)	4 (1.0)	2 (0.5)
Hormonal therapy	251 (30.6)	114 (29.1)	137 (31.9)
Chemotherapy, as well as immunotherapy or targeted therapy	11 (1.3)	3 (0.8)	8 (1.9)
Chemotherapy and hormonal therapy	56 (6.8)	25 (6.4)	31 (7.2)
Immunotherapy or targeted therapy, as well as hormonal therapy	23 (2.8)	10 (2.6)	13 (3.0)
Chemotherapy, immunotherapy or targeted therapy, as well as hormonal therapy	14 (1.7)	8 (2.0)	6 (1.4)

Values are *n* (%) unless otherwise indicated. Total does not add up due to missing values. Missing values were not included for calculation of the percentages. *The breast specimen of one participant in the ‘No-SLNB group’ contained a pathological macrometastasis. SLNB, sentinel lymph node biopsy; ER, oestrogen receptor; HER2, human epidermal growth factor receptor 2; ALND, axillary lymph node dissection; RT, radiation therapy.

**Table 2:** Baseline patient characteristics of BOOG 2013-08 participants selected for arm function and health-related quality-of-life analysis according to intention-to-treat analysis

Characteristic	Overall (*n* = 821)	SLNB group (*n* = 392)	No-SLNB group (*n* = 429)
**Age (years)**			
Mean(s.d.), range	61.6(9.3), 37–87	61.6(9.3), 38–85	61.6(9.3), 37–87
**BMI (kg/m^2^)**			
Mean(s.d.), range	27.1(4.9), 18–53	26.9(4.9), 18–53	27.3(4.9), 18–47
**Current smoker**			
Yes	115 (16.2)	55 (14.0)	60 (14.0)
Missing, *n*	110	46	64
**Relationship status**			
Married/committed	283 (72.6)	127 (69.0)	156 (75.7)
Divorced, separated, widowed, or single	94 (24.1)	52 (28.3)	42 (20.4)
Other	13 (3.3)	5 (2.7)	8 (3.9)
Missing, *n*	431	208	223
**Children**			
Yes	328 (84.1)	153 (83.2)	175 (85.0)
Missing, *n*	431	208	223
**Ethnicity**			
Dutch	379 (97.2)	182 (98.9)	197 (95.6)
European	6 (1.6)	0	6 (2.9)
Asian	2 (0.5)	1 (0.5)	1 (0.5)
Antillean	1 (0.3)	1 (0.5)	0
Surinamese	2 (0.5)	0	2 (1.0)
Missing, *n*	431	208	223
**Educational level**			
Low	174 (44.7)	82 (44.6)	92 (44.9)
Moderate	100 (25.7)	49 (26.6)	51 (24.9)
High	115 (29.6)	53 (28.8)	62 (30.2)
Missing, *n*	432	208	224
**Paid work**			
Yes	170 (44.0)	79 (43.6)	91 (44.4)
Missing, *n*	435	211	224
**Number of co-morbidities**			
0	30 (8.5)	15 (9.3)	15 (7.9)
1	108 (30.8)	54 (33.5)	54 (28.4)
≥2	213 (60.7)	92 (57.1)	121 (63.7)
Missing, *n*	470	231	239
**Trait anxiety**			
High	167 (20.3)	73 (18.6)	94 (21.9)
**Neuroticism**			
High	35 (4.3)	18 (4.6)	17 (4.0)

Values are *n* (%) unless otherwise indicated. Total does not add up due to missing values. Missing values were not included for calculation of the percentages. Educational level was categorized as low, moderate, or high; a low educational level includes primary school, lower vocational education, and low or intermediate general education, a moderate educational level includes intermediate vocational education and higher general education, and a high educational level includes higher vocational education and university. SLNB, sentinel lymph node biopsy.

### Arm function

**Fig. 2:**
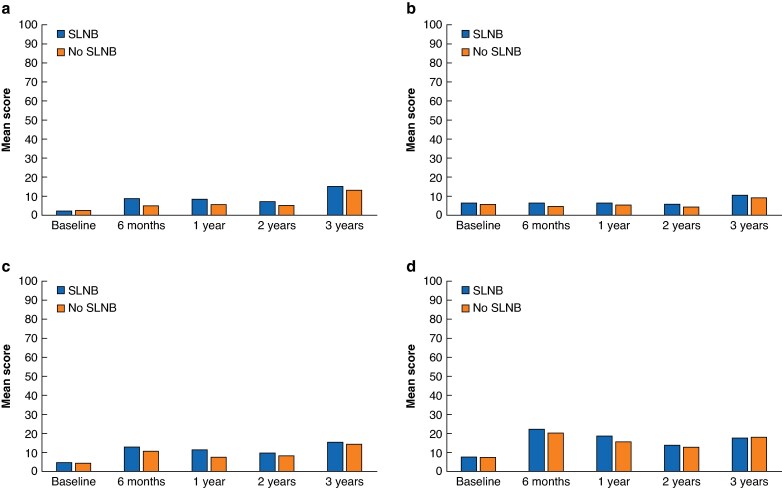
Arm function and health-related quality-of-life scores by treatment group **a** Lymph-ICF physical function score. **b** Lymph-ICF mental function score. **c** HRQoL arm symptom score. **d** HRQoL breast symptom score. SLNB, sentinel lymph node biopsy; Lymph-ICF, Lymphoedema Functioning, Disability, and Health questionnaire; HRQoL, health-related quality of life.

The unadjusted Lymph-ICF scores over time for each treatment group are presented in *Table S1*. At baseline, the scores were comparable between the SLNB group and the no-SLNB group. From 6 months up to 2 years after study inclusion, patients in the no-SLNB group experienced better physical functioning compared with patients in the SLNB group (5.2% *versus* 8.9% respectively at 6 months) and patients in the no-SLNB group experienced better mental functioning compared with patients in the SLNB group (5.1% *versus* 6.3% respectively at 6 months) (*P* values ranged between <0.001 and 0.042) (*Fig. 2a*,*b*). However, the statistically significant differences in these mean scores cannot be interpreted as clinically relevant. Other Lymph-ICF domains and the total score were not significantly different between treatment groups (*Table S1*).

### Health-related quality of life

*Table S1* also contains the unadjusted HRQoL scores for each treatment group. At baseline, significant differences were observed between treatment groups in the HRQoL scores for the physical functioning, fatigue, and dyspnoea scales, but these differences disappeared during follow-up. Mean HRQoL scores indicated no significant differences in global health scores or other EORTC QLQ-C30 or QLQ-BR-23 functioning scale scores between treatment groups at various time points (*Table S1*). Patients in the SLNB group experienced more EORTC QLQ-BR-23 arm symptoms at 6 months and 1 year after study inclusion (*P* = 0.049 and *P* < 0.001 respectively), as well as more breast symptoms after 1 year (*P* = 0.048) (*Fig. 2c*,*d*). None of these differences was clinically relevant. All other EORTC QLQ-BR-23 functioning and symptom scale scores did not show statistically significant differences between treatment groups at different time points.

### Relationship between personality and arm function or health-related quality of life

At baseline, 619 of 821 patients (75.4%) were categorized as having low trait anxiety and low neuroticism, 167 of 821 patients (20.3%) were categorized as having high trait anxiety, and 35 of 821 patients (4.3%) were categorized as having high neuroticism; 28 of 35 patients (80%) with high neuroticism levels also experienced high trait anxiety levels. Baseline patient and tumour characteristics categorized by personality group are shown in *Table S2*.

### Arm function stratified by personality group

**Fig. 3:**
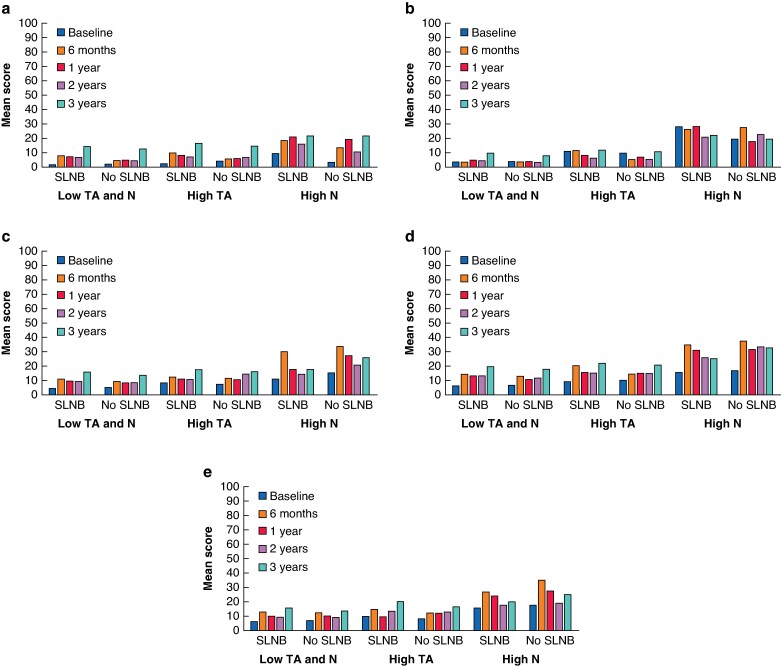
Arm function scores by personality group and by treatment group **a** Lymph-ICF physical function score. **b** Lymph-ICF mental function score. **c** Lymph-ICF household domain score. **d** Lymph-ICF mobility domain score. **e** Lymph-ICF social domain score. SLNB, sentinel lymph node biopsy; TA, trait anxiety; N, neuroticism; Lymph-ICF, Lymphoedema Functioning, Disability, and Health questionnaire.

The Lymph-ICF scores, stratified by personality group, are presented in *Table S3*. These scores indicate that the unadjusted baseline Lymph-ICF scores were similar across treatment groups. Analyses indicated that some Lymph-ICF domain scores favoured the no-SLNB group for patients with low trait anxiety and low neuroticism levels, as well as for those with high trait anxiety levels (*Fig. 3a–e* and *Table S3*). Irrespective of treatment group, patients with high neuroticism levels reported significantly more impairments in function, activities, and participation compared with those with low trait anxiety and low neuroticism levels and those with high trait anxiety levels. These differences were statistically significant and clinically relevant. The most notable and clinically relevant differences were seen in the mental, household, mobility, and life and social activities domains.

### Variables associated with total Lymph-ICF scores

The linear mixed-effect models for repeated measures for total Lymph-ICF scores (without personality group as a potential confounder) showed that SLNB (β = 2.7), multiple co-morbidities (β = 1.5), and higher BMI (β = 0.38) were all statistically significantly associated with reduced arm function, whereas retirement was associated with better arm function (β = −4.0) (*Table S4*; intention-to-treat analysis included as *Table S5*). When adding personality group to the linear mixed-effect models for repeated measures, systemic chemotherapy (β = 8.2) and a high neuroticism level (β = 13.1) also became statistically significantly associated with reduced arm function. Including or excluding personality group as a confounder in the linear mixed-effect models for repeated measures did not alter the direction or the significance of omitting the SLNB in the model.

### Health-related quality of life stratified by personality group

**Fig. 4:**
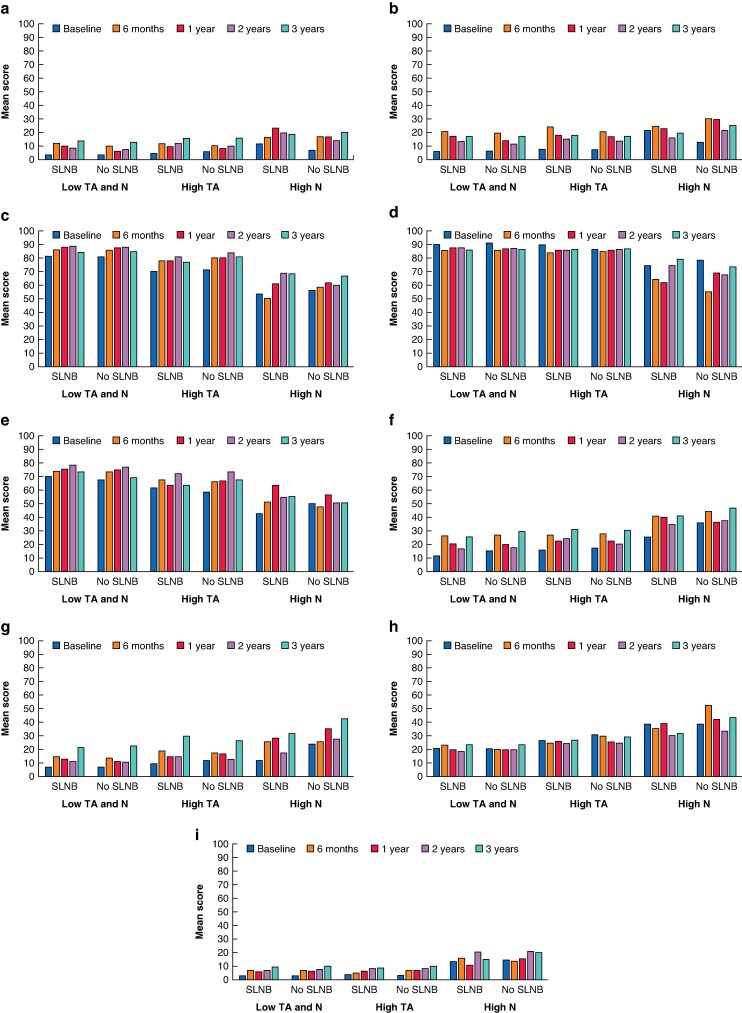
Health-related quality-of-life scores by personality group and by treatment group **a** HRQoL arm symptom score. **b** HRQoL breast symptom score. **c** HRQoL emotional functioning score. **d** HRQoL cognitive functioning score. **e** HRQoL future perspective score. **f** HRQoL fatigue symptom score. **g** HRQoL pain symptom score. **h** HRQoL insomnia symptom score. **i** HRQoL constipation symptom score. SLNB, sentinel lymph node biopsy; TA, trait anxiety; N, neuroticism; HRQoL, health-related quality of life.

*Table S3* also provides the unadjusted HRQoL scores for each treatment group, further stratified by personality group. The baseline HRQoL scores showed no statistically significant differences in functioning scale scores between treatment groups. At 6 months and at 1 year after study inclusion, patients with low trait anxiety and low neuroticism levels in the SLNB group reported more EORTC QLQ-BR-23 arm symptoms compared with those with the same personality traits in the no-SLNB group (*P* = 0.037 and *P* < 0.001 respectively) and, at 1 year after study inclusion, patients with low trait anxiety and low neuroticism levels in the SLNB group reported more EORTC QLQ-BR-23 breast symptoms compared with those with the same personality traits in the no-SLNB group (*P* = 0.007) (*Fig. 4a*,*b*). However, these statistically significant differences did not translate into clinically relevant differences. Irrespective of treatment group, patients with high trait anxiety or high neuroticism levels consistently reported lower HRQoL scores and more symptoms for nearly all HRQoL functioning and symptom scales compared with those with low trait anxiety and low neuroticism levels. Patients with high neuroticism levels consistently reported more symptoms compared with those with high trait anxiety levels. These differences were statistically significant and clinically relevant. The most prominent and clinically relevant differences in mean scores were seen for the emotional, cognitive, and future perspective functioning scales, as well as for the symptom scales for fatigue, pain, insomnia, constipation, and breast symptoms (*Fig. 4b–i*).

### Variables associated with global health-related quality-of-life scores

The linear mixed-effect models for repeated measures for global HRQoL scores (without personality group as a potential confounder) showed that SLNB was not associated with global HRQoL scores after adjusting for potential confounders (*P* = 0.557) (*Table S6*; intention-to-treat analysis included as *Table S7*). Systemic chemotherapy (β = −12.1), multiple co-morbidities (β = −3.1), moderate educational level (β = −5.0), and higher BMI (β = −0.30) were all statistically significantly associated with worse global HRQoL. When adding personality group to the linear mixed-effect models for repeated measures, a high neuroticism level (β = −12.1) and unemployment due to disability (β = −9.3) also became statistically significantly associated with worse global HRQoL. Including or excluding personality group as a confounder in the LMM did not alter the direction or the significance of omitting the SLNB in the model.

## Discussion

The primary aim of this study was to assess arm function and HRQoL in cT1–2 N0 breast cancer patients undergoing BCS with or without SLNB in the BOOG 2013-08 trial. Patients in the no-SLNB group experienced slightly better physical arm function up to 2 years after study inclusion and less HRQoL arm symptoms up to 1 year after study inclusion compared with the SLNB group. However, none of the differences in arm function scores exceeded the threshold of clinical relevance. The linear mixed-effect models for repeated measures showed that SLNB was significantly associated with reduced arm function when adjusted for potential confounders.

These results align with findings from comparable trials such as the INSEMA trial (NCT02466737) and the SOUND trial (NCT02167490)^21,39^. In the SOUND trial, using the QuickDASH questionnaire to assess physical function and upper-limb disorders^40^, SLNB patients reported 14.6% more upper limb symptoms 1 week after surgery compared with no-SLNB patients (*P* < 0.001). Similarly, in the present study, SLNB patients experienced more EORTC QLQ-BR-23 arm and breast symptoms, though this difference was smaller (3.7% at 6 months), and persisted up to 2 years after study inclusion. EORTC QLQ-BR-23 results from the INSEMA trial demonstrated that no-SLNB patients experienced fewer arm and breast symptoms up to 18 months after surgery compared with SLNB patients, a trend also observed in the present study, although not statistically significant. The INSEMA trial reported differences in EORTC QLQ-C30 physical functioning, favouring the no-SLNB group, whereas the present study found no difference in physical functioning between treatment groups. In the BOOG 2013-08 trial, none of the statistically significant differences between treatment groups was clinically relevant. This contrasts with the INSEMA trial, which showed a clinically relevant difference in EORTC QLQ-BR-23 arm function up to 18 months after surgery, favouring the no-SLNB group. This disparity may be due to the higher proportion of INSEMA patients (4.7%) undergoing additional ALND compared with BOOG 2013-08 patients (1.1%). Research has shown that ALND has a more profound negative impact on arm function and HRQoL than SLNB^41,42^, suggesting that the difference in ALND rates can significantly affect outcomes, contributing to the greater disparity observed in the INSEMA trial. Another explanation for the differences between the two trials may be the proportion of patients receiving tumour bed RT boosts; 88.1% in the INSEMA trial *versus* 39.6% in the BOOG 2013-08 trial^28,43^.

The secondary aim of this study was to assess the impact of the personality traits ‘trait anxiety’ and ‘neuroticism’ on arm function and global HRQoL. Previous studies suggested that patients with high trait anxiety levels perceive cancer diagnosis and treatment as more threatening, adversely affecting their HRQoL^23–26^. Similarly, patients with high neuroticism levels are more prone to negative emotions after diagnosis and treatment, leading to higher stress levels, sleep difficulties, and various mental and physical health symptoms^23–26^. The BOOG 2013-08 trial results confirmed that personality traits had a significantly negative impact on Lymph-ICF arm function and HRQoL, even more so than tumour characteristics or surgical procedures. Patients with high trait anxiety or neuroticism levels reported reduced arm function and worse HRQoL compared with those with low trait anxiety and neuroticism levels. Particularly, patients with high neuroticism levels experienced reduced arm function and lower HRQoL, especially in the psychosocial-related domains and symptom scales. The differences in mean scores between the personality groups were both statistically significant and clinically relevant at various time points. These findings indicate that breast cancer diagnosis and treatment have long-term effects on the well-being of patients with high trait anxiety or neuroticism, placing them at risk for diminished HRQoL. These results align with other breast cancer research, which has demonstrated a negative association and predictive value of trait anxiety^23,27,44–47^ and neuroticism^37,44,46,48–53^ with regard to HRQoL, whereas tumour- and treatment-related factors, such as tumour size and axillary treatment, have demonstrated little to no predictive value^47,49,54–58^.

Patient-reported outcomes provide crucial and unique insights into patients’ perceptions of their health and the effects of treatment, illuminating how diseases and surgical interventions impact various aspects of their lives^59,60^. Accurate interpretation of patient-reported outcomes at baseline and follow-up is essential for understanding their meaning and relevance when evaluating interventions. Adjusting for influential factors such as age, sex, disease stage, and educational level is crucial for accurate interpretation of HRQoL. Multiple studies, including this one, have shown a consistent association between personality traits and patient-reported outcomes such as HRQoL. Clinical studies rarely measure and adjust for personality traits, which raises questions about the interpretation of patient-reported outcomes. The findings of this study emphasize the need for systematic evaluation of personality traits as an integral part of oncological treatment. Early identification of patients who are psychologically more vulnerable to HRQoL deterioration should be implemented to offer these patients professional psychological support, helping them to manage the diagnosis, treatment, and possible side effects, thereby preventing a decline in HRQL.

While this is a large RCT with unique and detailed data on patient-reported outcomes and personality traits, this study also has a few limitations. To prevent lengthy questionnaires, the choice was made to only measure the personality traits ‘trait anxiety’ and ‘neuroticism’. This selection of personality traits prevented comprehension of the impact of other, possibly ‘protective’ personality dimensions, such as extraversion and conscientiousness, on arm function and HRQoL. Each personality dimension is linked with specific coping styles. The combination of personality and coping styles influences how patients manage the distress associated with diagnosis and treatment, or the adjustment to disease^46,54,57^. For this study, information on coping styles and other mediating and moderating effects was not available. Another limitation is the inability to determine whether the proportion of high trait anxiety or neuroticism patients was representative of the general population, as personality scores from the general population are missing. Comparison with similar studies showed that the proportions of patients with high trait anxiety and neuroticism levels in the BOOG 2013-08 trial are relatively low (high trait anxiety of 30–48% and high neuroticism of 21.3% in similar studies *versus* high trait anxiety of 20.3% and high neuroticism of 4.3% in the BOOG 2013-08 trial)^23,27,45^.

This study however has several strengths. The results are drawn from a large prospective and nationwide study sample with longitudinal data up to three years after study inclusion, including a large set of relevant and influential variables such as demographic- and tumour characteristics, and personality traits. Patient-reported outcomes were collected using validated and reliable questionnaires, with acceptable response rates.

The results from this study demonstrated that arm impairments and arm symptoms are significantly more prevalent after SLNB compared with its omission, but these did not impact physical HRQoL or lead to clinically relevant differences, suggesting a limited effect of SLNB combined with BCT on HRQoL. Far more striking are the results that high levels of trait anxiety and neuroticism had a significantly greater negative impact on arm function and HRQoL than clinical factors such as SLNB and tumour stage. These findings emphasize the need to assess personality traits to accurately interpret patient-reported outcomes and to identify those needing additional support after surgery.

## Supplementary Material

znaf031_Supplementary_Data

## Data Availability

The authors confirm that the data supporting the findings of this study are available within the article and its *Supplementary material*.
