# Supplementary material for: Three-year patient-reported outcomes of the BOOG 2013-08 RCT evaluating omission of sentinel lymph node biopsy in early-stage breast cancer patients treated with breast conserving surgery: Impact of personality traits on health-related quality of life
Source: Br J Surg. 2025 May 14;112(5):znaf031. doi: 10.1093/bjs/znaf031 (PMC12076147; doi:10.1093/bjs/znaf031)
Supplement: znaf031_Supplementary_Data [file znaf031_supplementary_data.zip › Amendementen_BOOG_2013-08._Part_A.pdf]

Sluiten Print

Formulier voor medisch-ethische beoordeling en registratie

ABR-formulier, versie december 2015

Onderzoeksdossiernummer

ABR Nummer	**49315**	Status	**Definitief**
Versie	**01**	Status per	**07-07-2014**
Jaar	**14**		
Dossiernummer	**NL49315.031.14**		

A. Sectie - Openbaar maken gegevens medisch wetenschappelijk onderzoek

A1. Het CCMO-register is een voor ieder toegankelijk openbaar trial register. De antwoorden op de vragen gemarkeerd met een wereldbol en de samenvatting bij dit formulier worden openbaar gemaakt in het CCMO register.

B. Sectie - Administratief

B1. Betreft het onderzoek met geneesmiddelen (inclusief gentherapie, somatische celtherapie, vaccinonderzoek, GGO's, zie verdere toelichting) als bedoeld in de Wet medisch-wetenschappelijk onderzoek met mensen (WMO)?

○ ja
◉ nee

B2. Houdt het onderzoek verband met een eerder door een erkende METC of door de CCMO beoordeelde studie of is het onderzoek reeds eerder bij een erkende METC ter beoordeling voorgelegd?

○ ja, het onderzoek houdt verband met – of is het vervolg op – een eerder beoordeelde studie
○ ja, het onderzoek is eerder ter beoordeling aan een erkende METC of de CCMO voorgelegd (stuur kopie besluit mee)
◉ nee

B4. Is het protocol (nog) in een ander openbaar trial register geregistreerd?

○ ja
◉ nee

B5. Naam indiener/contactpersoon voor de oordelende toetsingscommissie

**B5a.**	Achternaam indiener/contactpersoon	Gastel
	Titel en voorletters	ir. S.M.
	Tussenvoegsels	van
**B5b.**	Type organisatie/bedrijf	Overige Centra
	Organisatie/bedrijf	Anders, nl.
	Naam organisatie/bedrijf	IKNL trialbureau
	Afdeling	Trialbureau
	Adres	Hatertseweg 1
	Postcode en plaats	6533 AA Nijmegen
	Land	NL
**B5c.**	Intern adres	
	Telefoon	0243616837
	Fax	0243619080
	E-mail	s.vangastel@iknl.nl

B6. Is de indiener werkzaam bij de opdrachtgever/sponsor (verrichter) van het onderzoek?

○ Ja ◉ Nee

B7. Opdrachtgever/sponsor van het onderzoek (verrichter volgens de WMO)

Type Organisatie/Bedrijf	Universitair Medisch Centrum
Organisatie/Bedrijf	Medisch Universitair Ziekenhuis Maastricht
Adres	P. Debyelaan 25

Postcode en plaats 6229 HX Maastricht
Land NL
Telefoon 0433877477
Fax 0433875473
E-mail m.smidt@mumc.nl

C. Sectie - Onderzoek

C1. Volledige titel van het onderzoek
C1a. In het Engels
Clinically node negative breast cancer patients undergoing breast conserving therapy: Sentinel lymph node procedure versus follow-up. A Dutch randomized controlled multicentre trial.

C1b. In het Nederlands
De waarde van de schildwachtklierprocedure bij patiënten met borstkanker en een klinisch negatieve okselklierstatus die een borstsparende behandeling ondergaan. Een Nederlandse gerandomiseerde multicenter studie.

C2. Verkorte titel van het onderzoek/acroniem
C2a. In het Engels
BOOG 2013-08

C2b. In het Nederlands (Let op: deze korte titel wordt vermeld binnen ToetsingOnline)
BOOG 2013-08

C3. Trefwoorden (maximaal 4, plaats elk trefwoord op een aparte regel)
C3a. In het Engels
Breast neoplasms
Breast conserving therapy
Sentinel lymph node biopsy
Axilla

C3b. In het Nederlands
Borstkanker
Borstsparende behandeling
Schildwachtklierprocedure
Oksel

C4. Beschrijf het belang van het onderzoek en de beoogde toepassing van de resultaten (verwijs eventueel naar de relevante pagina's in het protocol).
Het doel van dit onderzoek is om:
1) Overbehandeling van de oksel te verminderen;
2) Het aantal korte en lange termijn complicaties te verminderen;
3) De kwaliteit van leven te verbeteren;
4) Met een even goede ziektecontrole en algehele overleving

Dit willen wij bereiken door te onderzoeken of de schildwachtklierprocedure veilig achterwege gelaten kan worden bij patiënten met borstkanker en een klinisch negatieve okselklierstatus behandeld met borstsparende therapie.

C6. Betreft het onderzoek een multicenter-onderzoek?
○ nee
◉ ja - alleen in Nederland
○ ja - internationaal binnen de Europese Unie
○ ja - internationaal ook buiten de Europese Unie

C7. Is er bij multicenter-onderzoek sprake van een coördinerend onderzoeker?
◉ ja namelijk — Naam onderzoeker: M.L. Smidt
○ nee

C8. Wie is/zijn medisch verantwoordelijk voor de proefpersonen die deelnemen aan het onderzoek
Behandelend arts en lokale hoofdonderzoeker

C9. In welk centrum/welke centra (incl. huisartsenpraktijken) in Nederland wordt het onderzoek uitgevoerd?

Centrum	Proefpersonen	Hoofdonderzoeker	Onafhankelijk arts
Albert Schweitzer Ziekenhuis	50	M.B.E. Menke-Pluijmers	S.O. Breukink (Maastricht UMC+)
Amphia Ziekenhuis	72	E.J.T. Luiten	S.O. Breukink (Maastricht UMC+)

Antonius Ziekenhuis, Sneek	58	C.G.J.M. van Riel	S.O. Breukink (Maastricht UMC+)
Atrium Medisch Centrum	40	E.R.M. van Haaren	S.O. Breukink (Maastricht UMC+)
Bronovo Ziekenhuis	24	O.R. Guicherit	S.O. Breukink (Maastricht UMC+)
Canisius Wilhelmina Ziekenhuis	48	L.J.A. Strobbe	S.O. Breukink (Maastricht UMC+)
Catharina-ziekenhuis	48	G.A.P. Nieuwenhuijzen	S.O. Breukink (Maastricht UMC+)
Deventer Ziekenhuis	40	H. de Vries	S.O. Breukink (Maastricht UMC+)
Zorggroep De Sionsberg	16	K. Blaauwendraat	S.O. Breukink (Maastricht UMC+)
Diakonessenhuis Utrecht	40	T. van Dalen	S.O. Breukink (Maastricht UMC+)
Gelre Ziekenhuizen	38	W.B. Lastdrager	S.O. Breukink (Maastricht UMC+)
Groene Hart Ziekenhuis	32	E. Linthorst	S.O. Breukink (Maastricht UMC+)
HagaZiekenhuis	42	J. Merkus	S.O. Breukink (Maastricht UMC+)
Ikazia Ziekenhuis	28	B.R. Toorenvliet	S.O. Breukink (Maastricht UMC+)
Isala Klinieken	56	A.B. Francken	S.O. Breukink (Maastricht UMC+)
Kennemer Gasthuis	45	R.J.C.L.M. Vuylsteke	S.O. Breukink (Maastricht UMC+)
LangeLand Ziekenhuis	14	B. Jas	S.O. Breukink (Maastricht UMC+)
Laurentius Ziekenhuis	24	A.V.R.J. Bell	S.O. Breukink (Maastricht UMC+)
Academisch Ziekenhuis Maastricht	43	M.L. Smidt	S.O. Breukink (Maastricht UMC+)
Martini Ziekenhuis	45	W. Kelder	S.O. Breukink (Maastricht UMC+)
Maxima Medisch Centrum	32	K. Schenk	S.O. Breukink (Maastricht UMC+)
Meander Medisch Centrum	45	A.J. van Overbeeke	S.O. Breukink (Maastricht UMC+)
Antoni van Leeuwenhoek Ziekenhuis	96	J.A. van der Hage	S.O. Breukink (Maastricht UMC+)
Onze Lieve Vrouwe Gasthuis	29	P.J. Borgstein	S.O. Breukink (Maastricht UMC+)
Orbis Medisch Centrum	24	Y. Vissers	S.O. Breukink (Maastricht UMC+)
Universitair Medisch Centrum Sint Radboud	32	J.H.W. de Wilt	S.O. Breukink (Maastricht UMC+)
Reinier de Graaf Groep	48	M. van 't Riet	S.O. Breukink (Maastricht UMC+)
Sint Antonius Ziekenhuis	60	R. Koelemij	S.O. Breukink (Maastricht UMC+)
Sint Elisabeth Ziekenhuis	38	P. Jansen	S.O. Breukink (Maastricht UMC+)
Streekziekenhuis Koningin Beatrix	19	A. Jonk	S.O. Breukink (Maastricht UMC+)
Tergooiziekenhuizen locatie Blaricum	44	E.J.C. Vriens	S.O. Breukink (Maastricht UMC+)
Universitair Medisch Centrum Groningen	18	J. de Vries	S.O. Breukink (Maastricht UMC+)
Universitair Medisch Centrum Utrecht	24	A.J. Witkamp	S.O. Breukink (Maastricht UMC+)
Viecuri Medisch Centrum voor Noord-Limburg	40	C.L.H. van Berlo	S.O. Breukink (Maastricht UMC+)
Westfries Gasthuis	28	J.W.D. de Waard	S.O. Breukink (Maastricht UMC+)
Waterland Ziekenhuis	22	L. de Widt	S.O. Breukink (Maastricht UMC+)
Zaans Medisch Centrum	16	S. Muller	S.O. Breukink (Maastricht UMC+)
Ziekenhuisvoorzieningen Gelderse Vallei	34	W.K. de Roos	S.O. Breukink (Maastricht UMC+)
Ziekenhuisgroep Twente	32	D. de Leeuw	S.O. Breukink (Maastricht UMC+)

Rivierenland Ziekenhuis	24	M.A.J. de Roos	S.O. Breukink (Maastricht UMC+)
Medisch Centrum Alkmaar	56	W.H. Schreurs	S.O. Breukink (Maastricht UMC+)
Medisch Centrum Haaglanden	32	A. Marinelli	S.O. Breukink (Maastricht UMC+)
Medisch Spectrum Twente	48	P. Steenvoorde	S.O. Breukink (Maastricht UMC+)

C10. Betreft het onderzoek met:

- ☑ mensen
- ☐ geslachtscellen
- ☐ (rest-)embryo's
- ☐ foetussen in utero

C11. Beoogd totaal aantal proefpersonen/(rest)embryo's/foetussen in utero:
C11a. In Nederland
1644

C13. Onderzoeksgebied

- ☐ etiologie
- ☐ organisatorisch/zorgonderzoek
- ☐ diagnostiek
- ☐ preventie
- ☑ therapie
- ☐ veiligheid
- ☐ werkzaamheid
- ☐ farmacokinetiek
- ☐ farmacodynamiek
- ☐ bio-equivalentie
- ☐ dosis-respons
- ☐ farmacogenomics
- ☐ farmaco-economie
- ☐ anders

C14. Type onderzoek

- ○ observationeel onderzoek zonder invasieve metingen
- ○ observationeel onderzoek met invasieve metingen
- ◉ interventie-onderzoek

C15. In welke fase kan het onderzoek worden ingedeeld?

- ○ fase I (a)
- ○ fase II (b)
- ◉ fase III (c)
- ○ fase IV (d)
- ○ overige onderzoeken waarbij geneesmiddelen worden toegepast (e)
- ○ niet van toepassing

C17. Is er sprake van een andere interventie dan met geneesmiddelen zoals bedoeld in de Wet medisch-wetenschappelijk onderzoek met mensen (WMO) (zie toelichting)?

- ◉ Ja
- ○ Nee

C17a. ☐ Medisch hulpmiddel, namelijk
C17b. ☐ Operatie, namelijk
C17c. ☐ Psychosociale interventie, namelijk
C17d. ☐ Voeding(stoffen), namelijk
C17e. ☐ Bewegingstherapie, namelijk
C17f. ☐ interventie met radioactieve straling, namelijk

C17g. ☐ Blootstellingsonderzoek (bijv pesticidenonderzoek), namelijk
C17h.
C17i. ☑ andere interventie, namelijk

In het Engels: No sentinel lymph node procedure
In het Nederlands: Géén schildwachtklierprocedure

C18. Worden de onderzoeksproducten voor deze studie door de verrichter gratis verstrekt?

○ ja
○ nee
○ gedeeltelijk namelijk
◉ niet van toepassing

C19. Is/zijn er (een) controlegroep (en)?

◉ ja
○ nee

☐ ja, geneesmiddel
☐ ja, placebo
☐ ja, geen interventie
☑ ja, anders

namelijkDe standaard behandeling: schildwachtklierprocedure

C20. Betreft het een gerandomiseerd onderzoek?

◉ ja
○ nee

namelijk

☑ open
☐ enkelblind
☐ dubbelblind
☐ parallel
☐ cross-over
☐ anders namelijk

C21. Op welke klasse(n) van aandoeningen heeft het onderzoek betrekking (maximaal 3)

☐ hartaandoeningen

☐ congenitale, familiaire en genetische aandoeningen
☐ bloed- en lymfestelsel aandoeningen
☐ zenuwstelsel aandoeningen
☐ oogaandoeningen
☐ evenwichtsorgaan- en ooraandoeningen
☐ ademhalingsstelsel-, thorax- en mediastinumaandoeningen
☐ maagdarmstelselaandoeningen
☐ nier- en urinewegaandoeningen
☐ huid- en onderhuidaandoeningen
☐ skeletspierstelsel- en bindweefselaandoeningen
☐ endocriene aandoeningen
☐ voedingsstoornissen en metabole ziekten
☐ infecties en parasitaire aandoeningen
☐ letsels, intoxicaties en verrichtingscomplicaties
☑ neoplasmata, benigne, maligne en niet-gespecificeerd (incl cysten en poliepen)
borstneoplasmata maligne en niet-gespecificeerd (incl. tepel)
☑ chirurgische en medische verrichtingen
borst therapeutische verrichtingen
☐ bloedvataandoeningen

☐ algemene aandoeningen en aandoeningen op de plek van toediening
☐ zwangerschap, perinatale periode en puerperium
☐ sociale omstandigheden
☐ immuunsysteemaandoeningen
☐ lever- en galaandoeningen
☐ voortplantingsstelsel- en borstaandoeningen
☐ psychische stoornissen
☐ overig, namelijk

C22.Geef twee synoniemen voor de aandoening die bestudeerd wordt, waarvan tenminste één lekenterm.

In het Engels:	breast cancer, sentinel lymph nodes, sentinel lymph node metastases
In het Nederlands:	borstkanker, schildwachtklieren, schildwachtkliermetastasen

C23. Beoogde start- en einddatum van het onderzoek

C23a. Start Datum (dd-mm-jjjj)	01-09-2014
C23b. Eind Datum (dd-mm-jjjj)	01-09-2026

C24. Zijn patiënten betrokken bij het ontwikkelen van het onderzoek? (Voor patiënten lees ook: patiëntenorganisaties, naasten of nabestaanden van patiënten en gezonde vrijwilligers)?

C30. Wordt de ervaring van proefpersonen na deelname aan het onderzoek geëvalueerd?

D. Sectie - Proefpersonen

D1. Is er een proefpersonenverzekering conform de WMO-eisen afgesloten of wordt aan de oordelende toetsingscommissie ontheffing gevraagd?

◉ proefpersonenverzekering is afgesloten bij verzekeringsmaatschappij
○ ontheffing van de verzekering wordt gevraagd
○ niet van toepassing - onderzoek valt onder de Embryowet en niet onder de WMO

Verzekeringsmaatschappij	Marketform (onderdeel van Lloyd's)

D2. Gezonde proefpersonen en/of patiënten

☐ Gezonde proefpersonen

☑ Patiënten

Aantal	1644

D4. Voornaamste inclusiecriteria

D4a. In het Engels
- Female
- Aged 18 years or older
- Pathologically confirmed invasive unilateral breast carcinoma
- A clinical T1-2 tumour (≤ 5cm)
- Will be treated with lumpectomy followed by whole breast radiotherapy
- Clinically node negative status: no signs of axillary lymph node metastases at physical examination and preoperative axillary ultrasound (or negative cyto-/histopathology)
- Written informed consent

D4b. In het Nederlands
- Vrouw
- Leeftijd 18 jaar of ouder
- Pathologisch bewezen invasief unilaterale borstkanker
- Klinisch T1-2 tumor (≤ 5cm)
- Zal worden behandeld met lumpectomie gevolgd door gehele borstbestraling
- Klinisch negatieve okselklierstatus: negatief lichamelijk onderzoek en echografie van de oksel (of negatieve cyto-/histologie)
- Schriftelijke informed consent

D5. Voornaamste exclusiecriteria

D5a. In het Engels
- Clinically node positive pre-operative
- Bilateral breast cancer
- Evidence of metastatic disease
- History of invasive breast cancer
- Previous treatment of the axilla with surgery or radiotherapy (except surgery for hidradenitis suppurativa or for other superficially located skin lesions, such as naevi)
- Pregnant or nursing

- Other prior malignancies within the past 5 years (except successfully treated basal cell and squamous cell skin cancer, carcinoma in situ of the cervix or carcinoma in situ of the ipsilateral or contralateral breast) or history of unsuccessfully treated malignancies
- Unable or unwilling to give informed consent

D5b. In het Nederlands

- Klinisch positieve okselklierstatus (preoperatief)
- Bilaterale borstkanker
- Primair (afstands-)gemetastaseerde borstkanker
- Invasieve borstkanker in de voorgeschiedenis
- Chirurgie of radiotherapie van oksel in de voorgeschiedenis (behalve chirurgie voor hidradenitis suppurativa of andere oppervlakkig gelegen huidlaesies, zoals naevi)
- Zwanger of lacterend
- Andere maligniteit in de afgelopen 5 jaar (behalve succesvol behandelde: huidkanker van type basaalcel of plaveiselcel; carcinoma in situ van de cervix; carcinoma in situ van de ipsilaterale of contralaterale mamma) of niet-curatief behandelde maligniteiten in de voorgeschiedenis

D6. Bij welke categorie proefpersonen wordt het onderzoek uitgevoerd (meerdere antwoorden mogelijk)

- [x] 18 jaar of ouder en wilsbekwaam
- [ ] 18 jaar of ouder en wilsonbekwaam
- [ ] 12 t/m 17 jaar en in staat tot het geven van geïnformeerde toestemming
- [ ] 12 t/m 17 jaar en niet in staat tot het geven van geïnformeerde toestemming (wilsonbekwaam)
- [ ] jonger dan 12 jaar

D10. Verkeren (sommige) proefpersonen in een afhankelijkheidssituatie ten opzichte van de onderzoeker of degene die de deelnemers werft? (lees de toelichting voor voorbeelden wanneer er sprake kan zijn van een afhankelijkheidssituatie)

(•) ja
( ) nee

D10a. Zo ja, waardoor?

Soms is de lokale onderzoeker ook de behandelend arts die de patiënt vraagt om deel te nemen aan de studie.

D10b. Waarom wordt het onderzoek juist met deze proefpersonen uitgevoerd en hoe worden de belangen van de proefpersonen gewaarborgd?

Het onderzoek moet met proefpersonen worden uitgevoerd die voldoen aan de beschreven in/exclusie criteria zodat de resultaten van de studie uiteindelijk ook naar de kliniek vertaald kunnen worden binnen deze patiëntenpopulatie. De belangen van de proefpersonen worden gewaarborgd door de geldende medisch ethische normen en richtlijn zoals vermeld in het protocol (o.a. onafhankelijk arts, uitgebreide patiëntinformatiefolder).

D11. Waaruit bestaat de vergoeding voor de proefpersonen?

- [x] geen vergoeding
- [ ] reiskosten
- [ ] financiële vergoeding (in Euro's)
- [ ] andere vergoeding

D12. Is deze vergoeding afhankelijk van bepaalde voorwaarden, bijvoorbeeld het voltooien van (een deel van) het onderzoek?

( ) ja (motiveer)
( ) nee
(•) niet van toepassing

E. Sectie - Voor- en nadelen

E1. Wordt er bij dit onderzoek een rechtstreeks therapeutisch effect beoogd bij de proefpersonen / patiënten?

(•) ja (therapeutisch onderzoek)
( ) nee (niet-therapeutisch onderzoek)

E1a. Zo ja, geef kort aan waaruit dit therapeutisch effect bestaat.

Het therapeutische effect bestaat uit:
- Het verlagen van het risico op okselbehandeling-gerelateerde korte en lange termijn complicaties;
- Het verbeteren van de kwaliteit van leven;
- Met een even goede ziektecontrole en algehele overleving.

E2. Waaruit bestaat de belasting van het onderzoek (en een eventueel daaraan voorafgaande keuring) voor de proefpersonen?

Tijdbeslag	per bezoek	0
	totaal	0

totale duur van de studie voor de individuele proefpersoon	Minder belasting dan normaal voor de studie-arm (geen schildwachtklierprocedure)

E3. Worden de proefpersonen in verband met het onderzoek in het ziekenhuis opgenomen of wordt een opname verlengd?

- ○ ja - het verblijf in het ziekenhuis/instituut wordt in verband met het onderzoek verlengd
- ○ ja - ze worden voor het onderzoek in het ziekenhuis/instituut opgenomen
- ◉ nee

E4. Beschrijf in hoeverre proefpersonen worden onderworpen aan handelingen dan wel een gedragswijze krijgen opgelegd, zoals vragenlijst, interviews, lichamelijk/psychologisch onderzoek, ontzegging, dieet (voor invasieve ingrepen: zie vraag E6)

In totaal zullen 700 proefpersonen (350 in de experimentele en 350 in de controlegroep) deelnemen aan het kwaliteit van leven onderzoek, bestaande uit een vragenlijst op het moment van randomisatie en op 5 tijdstippen na de randomisatie (na een half jaar, één, twee, vijf en 10 jaar).

E5. Worden de proefpersonen getest op bepaalde aandoeningen/condities?

- ○ ja (motiveer)
- ◉ nee

E6. Welke extra (invasieve) ingrepen (anders dan bij de standaard behandeling) moeten de proefpersonen in het kader van het onderzoek ondergaan:

- ☑ Niet van toepassing

E9. Geef aan welke risico's er voor proefpersonen zijn verbonden aan deelname aan het onderzoek.

Hoewel de hypothese luidt dat het achterwege laten van de schildwachtklierprocedure geen nadelige invloed heeft op de lokale controle van de ziekte en de overlevingskans, bestaat er toch een kans dat proefpersonen in de experimentele arm een hoger risico hebben op een recidief in de oksel (regionaal recidief) en daardoor tijdens follow-up alsnog een vorm van okselklierbehandeling moeten ondergaan. Uit onderzoek blijkt dat het uitgesteld uitvoeren van okselklierbehandeling (bijvoorbeeld met okselklierdissectie) géén nadelige gevolgen heeft voor de overleving.

E9a. Geef op grond van uw eigen afweging aan waarom het uitvoeren van het onderzoek, in het licht van de belasting en/of risico's die voor proefpersonen aan deelname verbonden zijn, gerechtvaardigd is?

Het mogelijk hogere risico op een recidief in de oksel (regionaal recidief) weegt op tegen de lagere kans op ernstige okselklierbehandeling-gerelateerde morbiditeit (lymfoedeem, bewegingsbeperking in de schouder, arm- en schouderpijn, verminderd gevoel of zenuwpijn) en de verbetering van kwaliteit van leven.

E10. Indien het onderzoek bij minderjarige en/of wilsonbekwame proefpersonen wordt uitgevoerd en geen direct therapeutisch effect wordt beoogd: waarom kunnen belasting en risico's als minimaal worden beschouwd? (Verwijs eventueel naar de relevante pagina's in het protocol.

- ☑ niet van toepassing

E11. Kan de eventuele therapie na beëindiging van het onderzoek worden voortgezet?

- ○ ja (motiveer)
- ○ nee (motiveer)
- ◉ niet van toepassing

E12. Heeft deelname aan het onderzoek voor de proefpersoon tot gevolg dat van de standaardbehandeling of -diagnostiek kan worden afgeweken of deze kan worden uitgesteld?

- ◉ ja
- ○ nee
- ○ niet van toepassing

E12a. Zo ja, waaruit bestaat de afwijking of het uitstel en waarom is afwijking/uitstel

Proefpersonen in de controlegroep ondergaan de standaard behandeling, namelijk schildwachtklierprocedure volgens de Nederlandse borstkankerrichtlijn. Proefpersonen in de experimentele arm ondergaan primair géén schildwachtklierprocedure. Beide groepen ondergaan dezelfde follow-up middels lichamelijk onderzoek en aanvullend onderzoek op indicatie. Indien een proefpersoon zich in de follow-up periode presenteert met lymfekliermetastasen, kan alsnog besloten worden om een vorm van okselklierbehandeling uit te voeren. Deze beslissing wordt gemaakt tijdens het multidisciplinair overleg in het desbetreffende centrum waar de patiënt onder behandeling staat.

F. Sectie - Informatie en privacy

F1. Hoe worden de proefpersonen geworven en door wie (onderzoeker, behandelend arts, andere persoon) wordt de proefpersoon/wettelijke vertegenwoordiger geïnformeerd en om toestemming gevraagd?

Op het moment in de behandeling van borstkanker dat de patiënt voldoet aan de in/exclusiecriteria zal de behandeld arts informatie geven over de mogelijkheid tot deelname aan deze studie. Een schriftelijke patiëntinformatiebrief zal worden meegegeven aan de patiënt. De patiënt krijgt ruim de tijd (ten minste één week) om deze informatie thuis na te lezen en een beslissing te nemen. De patiënt kan haar interesse in deelname kenbaar maken aan haar behandelend arts bij een volgend polibezoek. De behandelend arts zal haar dan om toestemming vragen voor deelname aan de studie.

F2. Hoeveel bedenktijd krijgen de proefpersonen/wettelijke vertegenwoordigers om te beslissen over deelname?
Zo lang als nodig, met ten minste één week

F3. Wordt de huisarts, behandelend specialist en/of apotheker van de proefpersoon geïnformeerd over diens deelname aan het onderzoek?

- (●) ja (de proefpersoon dient hiervoor toestemming te geven)
- ( ) nee

F4. Worden persoonsgegevens gecodeerd?

- (●) ja
- ( ) nee

F4a. Zo ja, hoe is deze codering opgebouwd?
Alle proefpersonen krijgen een uniek studienummer

F4b. Wie heeft toegang tot de sleutel van deze code?
De behandelend arts en de onderzoeker

F4c. Wie hebben toegang tot de brondocumenten en eventuele andere tot de persoon herleidbare gegevens?
De lokaal datamanagers en de onderzoeker

F5. Hoe wordt het lichaamsmateriaal gedurende het onderzoek bewaard?

- ( ) in tot de proefpersoon herleidbare vorm (gecodeerd)
- ( ) in niet tot de proefpersoon herleidbare vorm (volledig geanonimiseerd)
- (●) niet van toepassing

F6. Wordt afgenomen lichaamsmateriaal na afloop van het onderzoek vernietigd?

- ( ) ja
- ( ) nee (motiveer)
- (●) niet van toepassing

F7. Kunnen proefpersonen na afloop van het onderzoek opnieuw benaderd worden (bijvoorbeeld voor nader onderzoek of follow-up)?

- ( ) ja
- (●) nee

G. Sectie - Financieel

G1. Door welke geldstroom wordt het onderzoek gefinancierd?

- [ ] eerste geldstroom (Geld van Ministerie van OC&W aan universiteiten)
- [ ] tweede geldstroom (NWO of KNAW), namelijk
- [x] derde geldstroom (anders dan 1e of 2e geldstroom, zoals collectebusfondsen, Europese Unie, vakministeries of bedrijven), namelijk
KWF Kankerbestrijding

G2. Wordt het onderzoek (mede) gefinancierd door de industrie/bedrijven?

- ( ) ja - door de industrie/bedrijf zoals is opgegeven bij vraag B6/B7 (opdrachtgever van het onderzoek)
- ( ) ja - (ook) door andere industrie/bedrijven dan de opdrachtgever
- (●) nee

G3. Wat is de hoogte van de vergoeding die de arts/onderzoeker cq onderzoeksafdeling/maatschap ontvangt voor de uitvoering van het onderzoek?

- ( ) Per patiënt of proefpersoon
- (●) Per deelnemend centrum

bedrag afgerond in hele euro's: € 100,00

G3a. Hoe is de vergoeding opgebouwd?

De vergoeding betreft een financiële compensatie van € 100 excl. BTW als opstartvergoeding voor ieder deelnemend centrum.

G4. Heeft/hebben de onderzoeker(s) gedurende de afgelopen vijf jaar op een of andere wijze een persoonlijke financiële relatie (gehad) met de verrichter/sponsor van het huidige onderzoek?

- ○ ja (licht toe)
- ◉ nee

I. Sectie - Indiening en beoordeling

I1. Sla het formulier eerst op en selecteer vervolgens een METC of de CCMO

Commissie
METC NedMec

J. Sectie - Aanvullende opmerkingen

Aanvullende opmerkingen

K. Sectie - Samenvatting

Achtergrond van het onderzoek:

De schildwachtklierprocedure is de standaard methode voor lymfeklierstadiëring in borstkankerpatiënten met een klinisch negatieve okselklierstatus. In de algemene bevolking bevat de schildwachtklier in ongeveer 25% een uitzaaiing. Dit percentage is lager in patiënten met een kleine borsttumor en in borstsparend behandelde patiënten. De ACOSOG-Z0011 studie en de IBCSG 23-01 studie hebben recent aangetoond dat een completerende okselklierdissectie veilig achterwege gelaten kan worden bij borstsparend en met systemische therapie behandelde patiënten met een beperkt aantal schildwachtkliermetastasen. Dit roept de vraag op of de schildwachtklierprocedure nog wel noodzakelijk is voor borstsparend behandelde patiënten met een klinisch negatieve okselklierstatus. De klinisch negatieve okselklierstatus van de patiënten in de beschreven studies werd gesteld op basis van lichamelijk onderzoek. Het toevoegen van een preoperatieve echografie van de oksel zal zorgen voor een betere voorselectie van patiënten zonder lymfekliermetastasen, ten opzichte van lichamelijk onderzoek alleen. Daarbij sluit een negatieve echografie uitgevoerd in Nederland bijna alle patiënten uit met uitgebreide lymfekliermetastasen (≥4 lymfekliermetastasen). Bovendien omvat gehele borstbestraling na de borstsparende operatie in het algemeen het lagere deel van de oksel en behandelt daarmee eventueel achtergebleven uitzaaiingen. Hoewel de schildwachtklierprocedure minder invasief is vergeleken met een okselklierdissectie, komen complicaties nog steeds voor. Zo lijdt 6% van de patiënten die alleen een schildwachtklierprocedure ondergingen aan ernstige lymfoedeem na slechts 3 jaar. Deze studie gaat onderzoeken of de schildwachtklierprocedure veilig achterwege gelaten kan worden bij borstsparend behandelde patiënten, met een negatieve echografie van de oksel.

Doel van het onderzoek:

Het doel van deze studie is het verminderen van overbehandeling van de oksel bij patiënten met borstkanker en een klinisch negatieve okselklierstatus die behandeld worden met borstsparende therapie, door het achterwege laten van de schildwachtklierprocedure, om zodoende de kans op korte en lange termijn complicaties te verminderen en te zorgen voor een verbetering van de kwaliteit van leven met een even goede ziektecontrole en algehele overleving.

Onderzoeksopzet:

Het betreft een gerandomiseerde non-inferiority multicenter onderzoek. Patienten die voldoen aan de in- en exclusiecriteria worden na toestemming middels loting behandeld volgens één van de volgende behandelmethoden:

- Behandelmethode A (controle groep): schildwachtklierprocedure volgens de Nederlandse borstkankerrichtlijn.
- Behandelmethode B (studie groep): geen verdere okselklierstadiëring.

Stratificatiefactoren: leeftijd (≤50, 50≤75, >75 jaar), oestrogeen receptor status (positief vs. negatief), HER2neu status (geamplificeerd vs. niet geamplificeerd), klinisch tumor stadium (<3 cm vs. ≥3 cm), tumorgraad volgens gemodificeerde Bloom-Richardson (graad I-II vs. graad III), neoadjuvante systemische therapie, participerend centrum.
Overige behandelingen (o.a. hormonale therapie, chemotherapie) worden op indicatie gegeven volgens de Nederlandse borstkankerrichtlijn.
Tijdens de follow-up periode van 10 jaar wordt de oksel nauwkeurig onderzocht middels lichamelijk onderzoek en echografie op indicatie. Indien sprake is van een cyto-/histologisch bewezen lymfekliermetastase tijdens follow-up, dan kan in het multidisciplinair overleg van de desbetreffende patiënt worden besloten tot het uitvoeren van een vorm van okselklierbehandeling.
Gevalideerde vragenlijsten worden verstrekt voor het onderzoeken van de invloed op het aantal complicaties en kwaliteit van leven (Lymph-ICF, QLQ-C30, QLQ-BR23) en om te meten of angst en persoonlijkheidstrekken invloed hebben op de uitkomst van kwaliteit van leven (STAI-trait, NEO-FFI). Deze vragenlijsten worden verstrekt aan 700 van de 1644 patiënten (350 per arm), éénmal op het moment van randomisatie en vijfmaal na de randomisatie (6 maanden, 1 jaar, 2 jaar, 5 jaar en 10 jaar).

Onderzoekspopulatie:

Vrouwen van 18 jaar of ouder met pathologisch bewezen invasief mammacarcinoom die borstsparende therapie ondergaan.
cT1-2: primaire tumor is bij klinisch onderzoek kleiner dan 5 cm.
cN0: bij lichamelijk onderzoek en echografie van de oksel worden geen aanwijzingen voor lymfekliermetastasen gevonden of de cytologie/histologie na punctie/biopt van een lymfeklier is negatief.
Neoadjuvante systemische therapie is toegestaan indien de patiënt voor de start van de neoadjuvante systemische therapie een klinische T1-2N0 status heeft en waarvoor borstsparende therapie op dat moment al geschikt is.
De patiënt heeft informed consent persoonlijk ondertekend.

Interventie (indien van toepassing):

De controlegroep ondergaat wél de schildwachtklierprocedure volgens de Nederlands borstkankerrichtlijn. De experimentele arm ondergaan géén schildwachtklierprocedure.

Primaire onderzoeksvariabelen/uitkomstmaten:

Primaire uitkomstmaat:
- Regionaal recidief percentage

Secundaire onderzoeksvariabelen/uitkomstmaten (indien van toepassing):
Secundaire uitkomstmaten:
- Distant-disease free survival
- Overall survival
- Aantal later uitgevoerde okselklierdissecties ("delayed axillary lymph node dissection")
- Lokaal recidief percentage
- Overig-regionaal recidief percentage
- Contralaterale borstkanker
- Percentage (neo)adjuvante systemische therapie
- Kwaliteit van leven en okselbehandeling-gerelateerde morbiditeit

Omschrijving en inschatting van belasting en risico (indien van toepassing):

Wat betreft belasting is er in deze studie géén sprake van extra diagnostiek of interventies (juist minder) en hoeft de patiënt niet vaker dan normaal voor controle naar het ziekenhuis. De eventuele extra belasting voor de patiënt zal alleen bestaan uit het invullen van kwaliteit van leven vragenlijsten (bij 700 van 1644 patiënten) éénmaal op het moment van randomisatie en vijfmaal na randomisatie (6 maanden, 1 jaar, 2 jaar, 5 jaar en 10 jaar). De hypothese luidt dat het achterwege laten van de schildwachtklierprocedure even veilig is als het wél uitvoeren van deze procedure. Desondanks is er een risico dat in de experimentele arm meer regionale recidieven optreden en dat bij patiënten op een later tijdstip alsnog een vorm van okselklierbehandeling moet worden uitgevoerd. Daartegenover staat dat er in de experimentele arm door het niet uitvoeren van de schildwachtklierprocedure minder kans is op morbiditeit, minder operaties en opnames in het ziekenhuis en een betere kwaliteit van leven. Patiënten zullen op geen enkele manier vertraging ondervinden als gevolg van deelname aan de studie.

K2.Engelse Samenvatting

Background of the study:
The NSABP B-04 trial revealed that omitting primary axillary treatment of occult positive lymph nodes in clinically node negative breast cancer patients does not affect survival, even after 25 years of follow-up and without the use of adjuvant systemic or radiation therapy. A delayed axillary dissection in case lymph nodes become clinically positive does not affect survival and prevents axillary overtreatment in the majority of patients. The ACOSOG Z0011 and IBCSG 23-01 trials revealed that completion axillary dissection can be safely omitted in clinically node negative patients with metastatic sentinel nodes. Patients randomized for watchful waiting were likely to have residual nodal disease (13-27%) that was not surgically removed. Nevertheless, survival rates were not affected and regional recurrence rates low. Use of axillary ultrasound will improve the preoperative selection of node negative patients, as it selects patients with a more favourable tumour load and accurately excludes advanced nodal disease (≥4 metastatic nodes). Biology and adjuvant systemic and radiation therapy are all factors that most likely diminish the risk that possible metastases left in situ develop into clinically detectable lymph nodes. Patients treated by breast conserving therapy are significantly more often diagnosed with pathologic node negative disease or with micrometastatic disease, compared to patients treated by mastectomy. The risk for occult (macro)metastases in cT1-2N0 breast cancer patients treated by breast conserving therapy is low: about 88% will be node negative and about 95% of node positive patients will have no lymph node metastases beyond the sentinel node. Reflected by the low regional recurrence rate after a (false) negative sentinel node, only a small amount of patients (0.8%) with node positive disease is expected to develop clinically detectable nodal disease.

Objective of the study:
The objective of this study is to decrease the number of breast cancer patients receiving overtreatment of the axilla, in order to positively influence the axillary morbidity rate and quality of life. Therefore, we investigate whether the sentinel lymph node procedure can be safely omitted in clinically node negative breast cancer patients undergoing breast conserving therapy, in terms of non-inferior regional control, distant-disease free and overall survival. All objectives are measured during a follow-up of 10 years.

Study design:
A randomized controlled non-inferiority multicenter study. Patients who meet the in- and exclusioncriteria will be randomized after informed consent for one of the two treatment methods:
- Arm A (control arm): lumpectomy with sentinel lymph node procedure, followed by radiotherapy of the breast with or without completion axillary treatment according to the Dutch breast cancer guideline.
- Arm B (experimental arm): lumpectomy without further axillary staging, followed by radiotherapy of the breast.
Stratification: age (≤50, 50≤75, >75 years old), oestrogen receptor status (positive vs. negative), HER2neu status (amplified vs. not amplified), clinical tumour stage prior to any treatment (<3 cm vs. ≥3 cm), grading (grade I-II vs. III - according to Bloom-Richardson grading system), primary systemic therapy and participating centre.
Adjuvant systemic treatment is administered if indicated according to the Dutch breast cancer guideline.
Primary systemic therapy is allowed if the patient has a clinical T1-2N0 status (initial stage) that is amenable to lumpectomy pre-systemic therapy.
Yearly follow-up with physical examination of the axilla for 10 years. Axillary ultrasound (+/- tissue sampling) on indication. Staging for distant metastases and/or a delayed axillary lymph node dissection if indicated by the multidisciplinary team. Validated questionnaires are used to assess axillary morbidity rate and quality of life (Lymph-ICF, QLQ-C30, QLQ-BR23) and to measure if anxiety and personality traits influence the outcome of quality of life (STAI-trait, NEO-FFI) in 700 of 878 patients. These questionnaires are provided at the day of randomization and sequentially post randomization at 6 months, 1, 2, 5 and 10 years.

Study population:
Women aged 18 years or older with pathologically confirmed invasive breast carcinoma about to undergo breast conserving surgery followed by radiotherapy of the breast.
cT1-2: primary tumour is smaller than 5 cm.
cN0: no signs of axillary lymph node metastases at physical examination and axillary ultrasound (+/- tissue sampling).
Primary systemic treatment is allowed.
Patient is able to and signed the informed consent form.

Intervention (if applicable):
Patients in the control arm will be treated with the sentinel lymph node procedure according to the Dutch breast cancer guideline.
Patients in the experimental arm will not be treated with the sentinel lymph node procedure.

Primary study parameters/outcome of the study:
Primary endpoint:
- Regional recurrence rate

Secundary study parameters/outcome of the study (if applicable):
Secondary endpoints:
- Distant-disease free survival
- Overall survival
- Number of delayed axillary lymph node dissections
- Local recurrence rate
- Other-regional recurrence rate
- Contralateral breast cancer rate
- Percentage difference in the administration of (neo)adjuvant systemic therapy
- Quality of life and axillary morbidity rate

Nature and extent of the burden and risks associated with participation, benefit and group relatedness (if applicable):
There is no extra burden for participating patients as regard to additional diagnostic tests/interventions and follow-up. Any additional burden for the patient will only consist of completing quality of life questionnaires in 700 of 1644 study patients. These questionnaires are provided at the day of randomization and sequentially post randomization at 6 months, 1, 2, 5 and 10 years.
We hypothesize that omitting the sentinel lymph node procedure is not inferior to the standard treatment. Nevertheless, possible disadvantages are a worse regional recurrence rate, and delayed axillary treatment if indicated. Patients in the experimental arm have the possible advantages of less morbidity, fewer operations and hospitalizations and an improved quality of life, compared to patients treated with the sentinel lymph node procedure.
No patient will encounter any delay in their treatment as a result of inclusion.

ONDERTEKENING

De verrichter en indiener verklaren hierbij:
a. het formulier (en samenvatting) volledig en naar waarheid te hebben ingevuld;
b. de antwoorden op de vragen uit het ABR-formulier niet in strijd zijn met het bijbehorende onderzoeksdossier en onderzoekscontract

Naar waarheid getekend, door de verrichter (=opdrachtgever)		door de indiener	
datum	....	datum	....
Handtekening		Handtekening	
naam	M.J.H.M. Jacobs	naam	S.M. van Gastel
functie	Hoofd afdeling Chirurgie, MUMC+	functie	Trialconsulent, IKNL

Sluiten Print

Sluiten Print

Formulier voor medisch-ethische beoordeling en registratie

ABR-formulier, versie december 2015

Onderzoeksdossiernummer

ABR Nummer	**49315**	Status	**Beoordeeld - Positief**
Versie	**02**	Status per	**29-01-2015**
Jaar	**14**		
Dossiernummer	**NL49315.031.14**		
Reden voor	**Herindiening na vragenbrief PTC**		

A. Sectie - Openbaar maken gegevens medisch wetenschappelijk onderzoek

A1. Het CCMO-register is een voor ieder toegankelijk openbaar trial register. De antwoorden op de vragen gemarkeerd met een wereldbol en de samenvatting bij dit formulier worden openbaar gemaakt in het CCMO register.

B. Sectie - Administratief

B1. Betreft het onderzoek met geneesmiddelen (inclusief gentherapie, somatische celtherapie, vaccinonderzoek, GGO's, zie verdere toelichting) als bedoeld in de Wet medisch-wetenschappelijk onderzoek met mensen (WMO)?

○ ja
◉ nee

B2. Houdt het onderzoek verband met een eerder door een erkende METC of door de CCMO beoordeelde studie of is het onderzoek reeds eerder bij een erkende METC ter beoordeling voorgelegd?

○ ja, het onderzoek houdt verband met – of is het vervolg op – een eerder beoordeelde studie
○ ja, het onderzoek is eerder ter beoordeling aan een erkende METC of de CCMO voorgelegd (stuur kopie besluit mee)
◉ nee

B4. Is het protocol (nog) in een ander openbaar trial register geregistreerd?

○ ja
◉ nee

B5. Naam indiener/contactpersoon voor de oordelende toetsingscommissie

**B5a.**	Achternaam indiener/contactpersoon	Gastel
	Titel en voorletters	ir. S.M.
	Tussenvoegsels	van
**B5b.**	Type organisatie/bedrijf	Overige Centra
	Organisatie/bedrijf	Anders, nl.
	Naam organisatie/bedrijf	IKNL trialbureau
	Afdeling	Trialbureau
	Adres	Hatertseweg 1
	Postcode en plaats	6533 AA Nijmegen
	Land	NL
**B5c.**	Intern adres	
	Telefoon	0882346455
	Fax	0882346011
	E-mail	s.vangastel@iknl.nl

B6. Is de indiener werkzaam bij de opdrachtgever/sponsor (verrichter) van het onderzoek?

○ Ja ◉ Nee

B7. Opdrachtgever/sponsor van het onderzoek (verrichter volgens de WMO)

Type Organisatie/Bedrijf	Universitair Medisch Centrum
Organisatie/Bedrijf	Medisch Universitair Ziekenhuis Maastricht

Adres P. Debyelaan 25
Postcode en plaats 6229 HX Maastricht
Land NL
Telefoon 0433877477
Fax 0433875473
E-mail m.smidt@mumc.nl

C. Sectie - Onderzoek

C1. Volledige titel van het onderzoek
C1a. In het Engels
Clinically node negative breast cancer patients undergoing breast conserving therapy: Sentinel lymph node procedure versus follow-up. A Dutch randomized controlled multicentre trial.

C1b. In het Nederlands
Patiënten met borstkanker en een klinisch negatieve okselklierstatus die een borstsparende behandeling ondergaan: Schildwachtklierprocedure versus follow-up. Een Nederlandse gerandomiseerde multicenter studie.

C2. Verkorte titel van het onderzoek/acroniem
C2a. In het Engels
BOOG 2013-08

C2b. In het Nederlands (Let op: deze korte titel wordt vermeld binnen ToetsingOnline)
BOOG 2013-08

C3. Trefwoorden (maximaal 4, plaats elk trefwoord op een aparte regel)
C3a. In het Engels
Breast neoplasms
Breast conserving therapy
Sentinel lymph node biopsy
Axilla

C3b. In het Nederlands
Borstkanker
Borstsparende behandeling
Schildwachtklierprocedure
Oksel

C4. Beschrijf het belang van het onderzoek en de beoogde toepassing van de resultaten (verwijs eventueel naar de relevante pagina's in het protocol).
Het doel van dit onderzoek is om:
1) Overbehandeling van de oksel te verminderen;
2) Het aantal korte en lange termijn complicaties te verminderen;
3) De kwaliteit van leven te verbeteren;
4) Met een even goede ziektecontrole en algehele overleving

Dit willen wij bereiken door te onderzoeken of de schildwachtklierprocedure veilig achterwege gelaten kan worden bij patiënten met borstkanker en een klinisch negatieve okselklierstatus behandeld met borstsparende therapie.

C6. Betreft het onderzoek een multicenter-onderzoek?
- ○ nee
- ◉ ja - alleen in Nederland
- ○ ja - internationaal binnen de Europese Unie
- ○ ja - internationaal ook buiten de Europese Unie

C7. Is er bij multicenter-onderzoek sprake van een coördinerend onderzoeker?
- ◉ ja namelijk — Naam onderzoeker: M.L. Smidt
- ○ nee

C8. Wie is/zijn medisch verantwoordelijk voor de proefpersonen die deelnemen aan het onderzoek
Behandelend arts en lokale hoofdonderzoeker

C9. In welk centrum/welke centra (incl. huisartsenpraktijken) in Nederland wordt het onderzoek uitgevoerd?

Centrum	Proefpersonen	Hoofdonderzoeker	Onafhankelijk arts
Albert Schweitzer Ziekenhuis	49	M.B.E. Menke-Pluijmers	S.O. Breukink (Maastricht UMC+)
Amphia Ziekenhuis	69	E.J.T. Luiten	S.O. Breukink (Maastricht UMC+)

Antonius Ziekenhuis, Sneek	56	C.G.J.M. van Riel	S.O. Breukink (Maastricht UMC+)
Atrium Medisch Centrum	39	E.R.M. van Haaren	S.O. Breukink (Maastricht UMC+)
Bronovo Ziekenhuis	23	O.R. Guicherit	S.O. Breukink (Maastricht UMC+)
Canisius Wilhelmina Ziekenhuis	46	L.J.A. Strobbe	S.O. Breukink (Maastricht UMC+)
Catharina-ziekenhuis	46	G.A.P. Nieuwenhuijzen	S.O. Breukink (Maastricht UMC+)
Deventer Ziekenhuis	39	H. de Vries	S.O. Breukink (Maastricht UMC+)
Zorggroep De Sionsberg	15	K. Blaauwendraat	S.O. Breukink (Maastricht UMC+)
Diakonessenhuis Utrecht	39	T. van Dalen	S.O. Breukink (Maastricht UMC+)
Gelre Ziekenhuizen	37	W.B. Lastdrager	S.O. Breukink (Maastricht UMC+)
Groene Hart Ziekenhuis	31	E. Linthorst	S.O. Breukink (Maastricht UMC+)
HagaZiekenhuis	41	J. Merkus	S.O. Breukink (Maastricht UMC+)
Ikazia Ziekenhuis	27	B.R. Toorenvliet	S.O. Breukink (Maastricht UMC+)
Isala Klinieken	54	A.B. Francken	S.O. Breukink (Maastricht UMC+)
Kennemer Gasthuis	43	R.J.C.L.M. Vuylsteke	S.O. Breukink (Maastricht UMC+)
LangeLand Ziekenhuis	14	B. Jas	S.O. Breukink (Maastricht UMC+)
Laurentius Ziekenhuis	23	A.V.R.J. Bell	S.O. Breukink (Maastricht UMC+)
Academisch Ziekenhuis Maastricht	39	M.L. Smidt	S.O. Breukink (Maastricht UMC+)
Martini Ziekenhuis	43	W. Kelder	S.O. Breukink (Maastricht UMC+)
Maxima Medisch Centrum	31	K. Schenk	S.O. Breukink (Maastricht UMC+)
Meander Medisch Centrum	43	A.J. van Overbeeke	S.O. Breukink (Maastricht UMC+)
Antoni van Leeuwenhoek Ziekenhuis	93	J.A. van der Hage	S.O. Breukink (Maastricht UMC+)
Onze Lieve Vrouwe Gasthuis	28	P.J. Borgstein	S.O. Breukink (Maastricht UMC+)
Orbis Medisch Centrum	23	Y. Vissers	S.O. Breukink (Maastricht UMC+)
Universitair Medisch Centrum Sint Radboud	31	J.H.W. de Wilt	S.O. Breukink (Maastricht UMC+)
Reinier de Graaf Groep	46	M. van 't Riet	S.O. Breukink (Maastricht UMC+)
Sint Antonius Ziekenhuis	58	R. Koelemij	S.O. Breukink (Maastricht UMC+)
Sint Elisabeth Ziekenhuis	37	P. Jansen	S.O. Breukink (Maastricht UMC+)
Streekziekenhuis Koningin Beatrix	19	A. Jonk	S.O. Breukink (Maastricht UMC+)
Tergooiziekenhuizen locatie Blaricum	42	E.J.C. Vriens	S.O. Breukink (Maastricht UMC+)
Universitair Medisch Centrum Groningen	17	L. Jansen	S.O. Breukink (Maastricht UMC+)
Universitair Medisch Centrum Utrecht	23	A.J. Witkamp	S.O. Breukink (Maastricht UMC+)
Viecuri Medisch Centrum voor Noord-Limburg	39	C.L.H. van Berlo	S.O. Breukink (Maastricht UMC+)
Westfries Gasthuis	27	J.W.D. de Waard	S.O. Breukink (Maastricht UMC+)
Waterland Ziekenhuis	22	L. de Widt	S.O. Breukink (Maastricht UMC+)
Zaans Medisch Centrum	15	S. Muller	S.O. Breukink (Maastricht UMC+)
Ziekenhuisvoorzieningen Gelderse Vallei	32	W.K. de Roos	S.O. Breukink (Maastricht UMC+)
Ziekenhuisgroep Twente	31	D. de Leeuw	S.O. Breukink (Maastricht UMC+)

Rivierenland Ziekenhuis	23	M.A.J. de Roos	S.O. Breukink (Maastricht UMC+)
Medisch Centrum Alkmaar	54	W.H. Schreurs	S.O. Breukink (Maastricht UMC+)
Medisch Centrum Haaglanden	31	A. Marinelli	S.O. Breukink (Maastricht UMC+)
Medisch Spectrum Twente	46	P. Steenvoorde	S.O. Breukink (Maastricht UMC+)
Rijnstate Ziekenhuis	62	R. van Eekeren	S.O. Breukink (Maastricht UMC+)

C10. Betreft het onderzoek met:

- ☑ mensen
- ☐ geslachtscellen
- ☐ (rest-)embryo's
- ☐ foetussen in utero

C11. Beoogd totaal aantal proefpersonen/(rest)embryo's/foetussen in utero:
C11a. In Nederland
1644

C13. Onderzoeksgebied

- ☐ etiologie
- ☐ organisatorisch/zorgonderzoek
- ☐ diagnostiek
- ☐ preventie
- ☑ therapie
- ☐ veiligheid
- ☐ werkzaamheid
- ☐ farmacokinetiek
- ☐ farmacodynamiek
- ☐ bio-equivalentie
- ☐ dosis-respons
- ☐ farmacogenomics
- ☐ farmaco-economie
- ☐ anders

C14. Type onderzoek

- ○ observationeel onderzoek zonder invasieve metingen
- ○ observationeel onderzoek met invasieve metingen
- ◉ interventie-onderzoek

C15. In welke fase kan het onderzoek worden ingedeeld?

- ○ fase I (a)
- ○ fase II (b)
- ◉ fase III (c)
- ○ fase IV (d)
- ○ overige onderzoeken waarbij geneesmiddelen worden toegepast (e)
- ○ niet van toepassing

C17. Is er sprake van een andere interventie dan met geneesmiddelen zoals bedoeld in de Wet medisch-wetenschappelijk onderzoek met mensen (WMO) (zie toelichting)?

- ◉ Ja
- ○ Nee

C17a. ☐ Medisch hulpmiddel, namelijk
C17b. ☐ Operatie, namelijk
C17c. ☐ Psychosociale interventie, namelijk
C17d. ☐ Voeding(stoffen), namelijk
C17e. ☐ Bewegingstherapie, namelijk

C17f. ☐ interventie met radioactieve straling, namelijk
C17g. ☐ Blootstellingsonderzoek (bijv pesticidenonderzoek), namelijk
C17h.
C17i. ☑ andere interventie, namelijk

In het Engels: No sentinel lymph node procedure
In het Nederlands: Géén schildwachtklierprocedure

C18. Worden de onderzoeksproducten voor deze studie door de verrichter gratis verstrekt?

- ○ ja
- ○ nee
- ○ gedeeltelijk namelijk
- ◉ niet van toepassing

C19. Is/zijn er (een) controlegroep (en)?

- ◉ ja
- ○ nee

- ☐ ja, geneesmiddel
- ☐ ja, placebo
- ☐ ja, geen interventie
- ☑ ja, anders

namelijkDe standaard behandeling: schildwachtklierprocedure

C20. Betreft het een gerandomiseerd onderzoek?

- ◉ ja
- ○ nee

namelijk
- ☑ open
- ☐ enkelblind
- ☐ dubbelblind
- ☐ parallel
- ☐ cross-over
- ☐ anders namelijk

C21. Op welke klasse(n) van aandoeningen heeft het onderzoek betrekking (maximaal 3)

- ☐ hartaandoeningen
- ☐ congenitale, familiaire en genetische aandoeningen
- ☐ bloed- en lymfestelsel aandoeningen
- ☐ zenuwstelsel aandoeningen
- ☐ oogaandoeningen
- ☐ evenwichtsorgaan- en ooraandoeningen
- ☐ ademhalingsstelsel-, thorax- en mediastinumaandoeningen
- ☐ maagdarmstelselaandoeningen
- ☐ nier- en urinewegaandoeningen
- ☐ huid- en onderhuidaandoeningen
- ☐ skeletspierstelsel- en bindweefselaandoeningen
- ☐ endocriene aandoeningen
- ☐ voedingsstoornissen en metabole ziekten
- ☐ infecties en parasitaire aandoeningen
- ☐ letsels, intoxicaties en verrichtingscomplicaties
- ☑ neoplasmata, benigne, maligne en niet-gespecificeerd (incl cysten en poliepen)
  borstneoplasmata maligne en niet-gespecificeerd (incl. tepel)
- ☑ chirurgische en medische verrichtingen
  borst therapeutische verrichtingen

- ☐ bloedvataandoeningen
- ☐ algemene aandoeningen en aandoeningen op de plek van toediening
- ☐ zwangerschap, perinatale periode en puerperium
- ☐ sociale omstandigheden
- ☐ immuunsysteemaandoeningen
- ☐ lever- en galaandoeningen
- ☐ voortplantingsstelsel- en borstaandoeningen
- ☐ psychische stoornissen
- ☐ overig, namelijk

C22.Geef twee synoniemen voor de aandoening die bestudeerd wordt, waarvan tenminste één lekenterm.

In het Engels:	breast cancer, sentinel lymph nodes, sentinel lymph node metastases
In het Nederlands:	borstkanker, schildwachtklieren, schildwachtkliermetastasen

C23. Beoogde start- en einddatum van het onderzoek

C23a. Start Datum (dd-mm-jjjj)	01-01-2015
C23b. Eind Datum (dd-mm-jjjj)	01-01-2027

C24. Zijn patiënten betrokken bij het ontwikkelen van het onderzoek? (Voor patiënten lees ook: patiëntenorganisaties, naasten of nabestaanden van patiënten en gezonde vrijwilligers)?

C30. Wordt de ervaring van proefpersonen na deelname aan het onderzoek geëvalueerd?

D. Sectie - Proefpersonen

D1. Is er een proefpersonenverzekering conform de WMO-eisen afgesloten of wordt aan de oordelende toetsingscommissie ontheffing gevraagd?

- ◉ proefpersonenverzekering is afgesloten bij verzekeringsmaatschappij
- ○ ontheffing van de verzekering wordt gevraagd
- ○ niet van toepassing - onderzoek valt onder de Embryowet en niet onder de WMO

Verzekeringsmaatschappij	Marketform (onderdeel van Lloyd's)

D2. Gezonde proefpersonen en/of patiënten

- ☐ Gezonde proefpersonen

- ☑ Patiënten

Aantal	1644

D4. Voornaamste inclusiecriteria

D4a. In het Engels

- Female
- Aged 18 years or older
- Pathologically confirmed invasive unilateral breast carcinoma
- A clinical T1-2 tumour (≤ 5cm)
- Will be treated with lumpectomy followed by whole breast radiotherapy
- Clinically node negative status: no signs of axillary lymph node metastases at physical examination and preoperative axillary ultrasound (or negative cyto-/histopathology)
- Written informed consent

D4b. In het Nederlands

- Vrouw
- Leeftijd 18 jaar of ouder
- Pathologisch bewezen invasief unilaterale borstkanker
- Klinisch T1-2 tumor (≤ 5cm)
- Zal worden behandeld met lumpectomie gevolgd door gehele borstbestraling
- Klinisch negatieve okselklierstatus: negatief lichamelijk onderzoek en echografie van de oksel (of negatieve cyto-/histologie)
- Schriftelijke informed consent

D5. Voornaamste exclusiecriteria

D5a. In het Engels

- Clinically node positive pre-operative
- Bilateral breast cancer
- Evidence of metastatic disease
- History of invasive breast cancer
- Previous treatment of the ipsilateral axilla with surgery or radiotherapy, except surgery for hidradenitis suppurativa or for other superficially located skin lesions, such as naevi

- Pregnant or nursing
- Other prior malignancies within the past 5 years, except successfully treated malignancies that occurred more than five years before randomization, and except successfully treated basal cell and squamous cell skin cancer, carcinoma in situ of the cervix or carcinoma in situ of the ipsilateral or contralateral breast
- Unable or unwilling to give informed consent

D5b. In het Nederlands
- Klinisch positieve okselklierstatus (preoperatief)
- Bilaterale borstkanker
- Primair (afstands-)gemetastaseerde borstkanker
- Invasieve borstkanker in de voorgeschiedenis
- Chirurgie of radiotherapie van ipsilaterale oksel in de voorgeschiedenis, behalve chirurgie voor hidradenitis suppurativa of andere oppervlakkig gelegen huidlaesies, zoals naevi
- Zwanger of lacterend
- Andere maligniteit in de afgelopen 5 jaar, behalve succesvol behandelde maligniteit die meer dan vijf jaar voor randomisatie optrad, en behalve succesvol behandelde huidkanker van type basaalcel of plaveiselcel, carcinoma in situ van de cervix, carcinoma in situ van de ipsilaterale of contralaterale mamma
- Niet in staat of niet bereid tot informed consent

D6. Bij welke categorie proefpersonen wordt het onderzoek uitgevoerd (meerdere antwoorden mogelijk)

- [x] 18 jaar of ouder en wilsbekwaam
- [ ] 18 jaar of ouder en wilsonbekwaam
- [ ] 12 t/m 17 jaar en in staat tot het geven van geïnformeerde toestemming
- [ ] 12 t/m 17 jaar en niet in staat tot het geven van geïnformeerde toestemming (wilsonbekwaam)
- [ ] jonger dan 12 jaar

D10. Verkeren (sommige) proefpersonen in een afhankelijkheidssituatie ten opzichte van de onderzoeker of degene die de deelnemers werft? (lees de toelichting voor voorbeelden wanneer er sprake kan zijn van een afhankelijkheidssituatie)

◉ ja
○ nee

D10a. Zo ja, waardoor?
Soms is de lokale onderzoeker ook de behandelend arts die de patiënt vraagt om deel te nemen aan de studie.

D10b. Waarom wordt het onderzoek juist met deze proefpersonen uitgevoerd en hoe worden de belangen van de proefpersonen gewaarborgd?
Het onderzoek moet met proefpersonen worden uitgevoerd die voldoen aan de beschreven in/exclusie criteria zodat de resultaten van de studie uiteindelijk ook naar de kliniek vertaald kunnen worden binnen deze patiëntenpopulatie. De belangen van de proefpersonen worden gewaarborgd door de geldende medisch ethische normen en richtlijn zoals vermeld in het protocol (o.a. onafhankelijk arts, uitgebreide patiëntinformatiefolder).

D11. Waaruit bestaat de vergoeding voor de proefpersonen?

- [x] geen vergoeding
- [ ] reiskosten
- [ ] financiële vergoeding (in Euro's)
- [ ] andere vergoeding

D12. Is deze vergoeding afhankelijk van bepaalde voorwaarden, bijvoorbeeld het voltooien van (een deel van) het onderzoek?

○ ja (motiveer)
○ nee
◉ niet van toepassing

E. Sectie - Voor- en nadelen

E1. Wordt er bij dit onderzoek een rechtstreeks therapeutisch effect beoogd bij de proefpersonen / patiënten?

◉ ja (therapeutisch onderzoek)
○ nee (niet-therapeutisch onderzoek)

E1a. Zo ja, geef kort aan waaruit dit therapeutisch effect bestaat.
Het therapeutische effect bestaat uit:
- Het verlagen van het risico op okselbehandeling-gerelateerde korte en lange termijn complicaties;
- Het verbeteren van de kwaliteit van leven;
- Met een even goede ziektecontrole en algehele overleving.

E2. Waaruit bestaat de belasting van het onderzoek (en een eventueel daaraan voorafgaande keuring) voor de proefpersonen?

Tijdbeslag	per bezoek	20 min per keer voor invullen kwaliteit van leven vragenlijst
	totaal	20 min x 6 = 120 min
	totale duur van de studie voor de individuele proefpersoon	Tav de behandeling minder belasting dan normaal voor de studie-arm (geen schildwachtklierprocedure). Voor beide armen is extra het invullen van de kwaliteit van leven vragenlijst prerandomisatie en postrandomisatie op 5 momenten.

E3. Worden de proefpersonen in verband met het onderzoek in het ziekenhuis opgenomen of wordt een opname verlengd?

○ ja - het verblijf in het ziekenhuis/instituut wordt in verband met het onderzoek verlengd
○ ja - ze worden voor het onderzoek in het ziekenhuis/instituut opgenomen
◉ nee

E4. Beschrijf in hoeverre proefpersonen worden onderworpen aan handelingen dan wel een gedragswijze krijgen opgelegd, zoals vragenlijst, interviews, lichamelijk/psychologisch onderzoek, ontzegging, dieet (voor invasieve ingrepen: zie vraag E6)

In totaal zullen 700 proefpersonen (350 in de experimentele en 350 in de controlegroep) deelnemen aan het kwaliteit van leven onderzoek, bestaande uit een vragenlijst voor de randomisatie en op 5 tijdstippen na de randomisatie (na een half jaar, één, twee, vijf en 10 jaar).

E5. Worden de proefpersonen getest op bepaalde aandoeningen/condities?

○ ja (motiveer)
◉ nee

E6. Welke extra (invasieve) ingrepen (anders dan bij de standaard behandeling) moeten de proefpersonen in het kader van het onderzoek ondergaan:

☑ Niet van toepassing

E9. Geef aan welke risico's er voor proefpersonen zijn verbonden aan deelname aan het onderzoek.

Hoewel de hypothese luidt dat het achterwege laten van de schildwachtklierprocedure geen nadelige invloed heeft op de lokale controle van de ziekte en de overlevingskans, bestaat er toch een kans dat proefpersonen in de experimentele arm een hoger risico hebben op een recidief in de oksel (regionaal recidief) en daardoor tijdens follow-up alsnog een vorm van okselklierbehandeling moeten ondergaan. Uit onderzoek blijkt dat het uitgesteld uitvoeren van okselklierbehandeling (bijvoorbeeld met okselklierdissectie) géén nadelige gevolgen heeft voor de overleving.

E9a. Geef op grond van uw eigen afweging aan waarom het uitvoeren van het onderzoek, in het licht van de belasting en/of risico's die voor proefpersonen aan deelname verbonden zijn, gerechtvaardigd is?

Het mogelijk hogere risico op een recidief in de oksel (regionaal recidief) weegt op tegen de lagere kans op ernstige okselklierbehandeling-gerelateerde morbiditeit (lymfoedeem, bewegingsbeperking in de schouder, arm- en schouderpijn, verminderd gevoel of zenuwpijn) en de verbetering van kwaliteit van leven.

E10. Indien het onderzoek bij minderjarige en/of wilsonbekwame proefpersonen wordt uitgevoerd en geen direct therapeutisch effect wordt beoogd: waarom kunnen belasting en risico's als minimaal worden beschouwd? (Verwijs eventueel naar de relevante pagina's in het protocol.

☑ niet van toepassing

E11. Kan de eventuele therapie na beëindiging van het onderzoek worden voortgezet?

○ ja (motiveer)
○ nee (motiveer)
◉ niet van toepassing

E12. Heeft deelname aan het onderzoek voor de proefpersoon tot gevolg dat van de standaardbehandeling of -diagnostiek kan worden afgeweken of deze kan worden uitgesteld?

◉ ja
○ nee
○ niet van toepassing

E12a. Zo ja, waaruit bestaat de afwijking of het uitstel en waarom is afwijking/uitstel

Proefpersonen in de controlegroep ondergaan de standaard behandeling, namelijk schildwachtklierprocedure volgens de Nederlandse borstkankerrichtlijn. Proefpersonen in de experimentele arm ondergaan primair géén schildwachtklierprocedure. Beide groepen ondergaan dezelfde follow-up middels lichamelijk onderzoek en aanvullend onderzoek op indicatie. Indien een proefpersoon zich in de follow-up periode presenteert met lymfekliermetastasen, kan alsnog besloten worden om een vorm van okselklierbehandeling uit te voeren. Deze

beslissing wordt gemaakt tijdens het multidisciplinair overleg in het desbetreffende centrum waar de patiënt onder behandeling staat.

F. Sectie - Informatie en privacy

F1. Hoe worden de proefpersonen geworven en door wie (onderzoeker, behandelend arts, andere persoon) wordt de proefpersoon/wettelijke vertegenwoordiger geïnformeerd en om toestemming gevraagd?

Op het moment in de behandeling van borstkanker dat de patiënt voldoet aan de in/exclusiecriteria zal de behandeld arts informatie geven over de mogelijkheid tot deelname aan deze studie. Een schriftelijke patiëntinformatiebrief zal worden meegegeven aan de patiënt. De patiënt krijgt ruim de tijd (ten minste één week) om deze informatie thuis na te lezen en een beslissing te nemen. De patiënt kan haar interesse in deelname kenbaar maken aan haar behandelend arts bij een volgend polibezoek. De behandelend arts zal haar dan om toestemming vragen voor deelname aan de studie.

F2. Hoeveel bedenktijd krijgen de proefpersonen/wettelijke vertegenwoordigers om te beslissen over deelname?

Zo lang als nodig, met ten minste één week

F3. Wordt de huisarts, behandelend specialist en/of apotheker van de proefpersoon geïnformeerd over diens deelname aan het onderzoek?

- (●) ja (de proefpersoon dient hiervoor toestemming te geven)
- ( ) nee

F4. Worden persoonsgegevens gecodeerd?

- (●) ja
- ( ) nee

F4a. Zo ja, hoe is deze codering opgebouwd?

Alle proefpersonen krijgen een uniek studienummer. Het unieke studienummer betreft een opvolgnummer zonder verdere specifieke codering voor bijvoorbeeld centrum.

F4b. Wie heeft toegang tot de sleutel van deze code?

De behandelend arts en de onderzoeker

F4c. Wie hebben toegang tot de brondocumenten en eventuele andere tot de persoon herleidbare gegevens?

De lokaal datamanagers en de onderzoeker

F5. Hoe wordt het lichaamsmateriaal <u>gedurende</u> het onderzoek bewaard?

- ( ) in tot de proefpersoon herleidbare vorm (gecodeerd)
- ( ) in niet tot de proefpersoon herleidbare vorm (volledig geanonimiseerd)
- (●) niet van toepassing

F6. Wordt afgenomen lichaamsmateriaal na afloop van het onderzoek vernietigd?

- ( ) ja
- ( ) nee (motiveer)
- (●) niet van toepassing

F7. Kunnen proefpersonen na afloop van het onderzoek opnieuw benaderd worden (bijvoorbeeld voor nader onderzoek of follow-up)?

- ( ) ja
- (●) nee

G. Sectie - Financieel

G1. Door welke geldstroom wordt het onderzoek gefinancierd?

- [ ] eerste geldstroom (Geld van Ministerie van OC&W aan universiteiten)
- [ ] tweede geldstroom (NWO of KNAW), namelijk
- [x] derde geldstroom (anders dan 1e of 2e geldstroom, zoals collectebusfondsen, Europese Unie, vakministeries of bedrijven), namelijk
  KWF Kankerbestrijding

G2. Wordt het onderzoek (mede) gefinancierd door de industrie/bedrijven?

- ( ) ja - door de industrie/bedrijf zoals is opgegeven bij vraag B6/B7 (opdrachtgever van het onderzoek)
- ( ) ja - (ook) door andere industrie/bedrijven dan de opdrachtgever
- (●) nee

G3. Wat is de hoogte van de vergoeding die de arts/onderzoeker cq onderzoeksafdeling/maatschap ontvangt voor de uitvoering van het onderzoek?

○ Per patiënt of proefpersoon

◉ Per deelnemend centrum

bedrag afgerond in hele euro's: € 100,00

G3a. Hoe is de vergoeding opgebouwd?

De vergoeding betreft een financiële compensatie van € 100 excl. BTW als opstartvergoeding voor ieder deelnemend centrum.

G4. Heeft/hebben de onderzoeker(s) gedurende de afgelopen vijf jaar op een of andere wijze een persoonlijke financiële relatie (gehad) met de verrichter/sponsor van het huidige onderzoek?

○ ja (licht toe)

◉ nee

I. Sectie - Indiening en beoordeling

I1. Sla het formulier eerst op en selecteer vervolgens een METC of de CCMO

Commissie
METC NedMec

J. Sectie - Aanvullende opmerkingen

Aanvullende opmerkingen

K. Sectie - Samenvatting

Achtergrond van het onderzoek:

De schildwachtklierprocedure is de standaard methode voor lymfeklierstadiëring in borstkankerpatiënten met een klinisch negatieve okselklierstatus. In de algemene bevolking bevat de schildwachtklier in ongeveer 25% een uitzaaiing. Dit percentage is lager in patiënten met een kleine borsttumor en in borstsparend behandelde patiënten. De ACOSOG-Z0011 studie en de IBCSG 23-01 studie hebben recent aangetoond dat een completerende okselklierdissectie veilig achterwege gelaten kan worden bij borstsparend en met systemische therapie behandelde patiënten met een beperkt aantal schildwachtkliermetastasen. Dit roept de vraag op of de schildwachtklierprocedure nog wel noodzakelijk is voor borstsparend behandelde patiënten met een klinisch negatieve okselklierstatus. De klinisch negatieve okselklierstatus van de patiënten in de beschreven studies werd gesteld op basis van lichamelijk onderzoek. Het toevoegen van een preoperatieve echografie van de oksel zal zorgen voor een betere voorselectie van patiënten zonder lymfekliermetastasen, ten opzichte van lichamelijk onderzoek alleen. Daarbij sluit een negatieve echografie uitgevoerd in Nederland bijna alle patiënten uit met uitgebreide lymfekliermetastasen (≥4 lymfekliermetastasen). Bovendien omvat gehele borstbestraling na de borstsparende operatie in het algemeen het lagere deel van de oksel en behandelt daarmee eventueel achtergebleven uitzaaiingen. Hoewel de schildwachtklierprocedure minder invasief is vergeleken met een okselklierdissectie, komen complicaties nog steeds voor. Zo lijdt 6% van de patiënten die alleen een schildwachtklierprocedure ondergingen aan ernstige lymfoedeem na slechts 3 jaar. Deze studie gaat onderzoeken of de schildwachtklierprocedure veilig achterwege gelaten kan worden bij borstsparend behandelde patiënten, met een negatieve echografie van de oksel.

Doel van het onderzoek:

Het doel van deze studie is het verminderen van overbehandeling van de oksel bij patiënten met borstkanker en een klinisch negatieve okselklierstatus die behandeld worden met borstsparende therapie, door het achterwege laten van de schildwachtklierprocedure, om zodoende de kans op korte en lange termijn complicaties te verminderen en te zorgen voor een verbetering van de kwaliteit van leven met een even goede ziektecontrole en algehele overleving.

Onderzoeksopzet:

Het betreft een gerandomiseerde non-inferiority multicenter onderzoek. Patienten die voldoen aan de in- en exclusiecriteria worden na toestemming middels loting behandeld volgens één van de volgende behandelmethoden:

- Behandelmethode A (controle groep): schildwachtklierprocedure volgens de Nederlandse borstkankerrichtlijn.
- Behandelmethode B (studie groep): geen verdere okselklierstadiëring.

Stratificatiefactoren: leeftijd (≤50, 50≤75, >75 jaar), oestrogeen receptor status (positief vs. negatief), HER2neu status (geamplificeerd vs. niet geamplificeerd), klinisch tumor stadium (<3 cm vs. ≥3 cm), tumorgraad volgens gemodificeerde Bloom-Richardson (graad I-II vs. graad III), is of zal worden behandeld met primaire systemische therapie (ja/nee), participerend centrum.
Overige behandelingen (o.a. hormonale therapie, chemotherapie) worden op indicatie gegeven volgens de Nederlandse borstkankerrichtlijn.
Tijdens de follow-up periode van 10 jaar wordt de oksel nauwkeurig onderzocht middels lichamelijk onderzoek en echografie op indicatie. Indien sprake is van een cyto-/histologisch bewezen lymfekliermetastase tijdens follow-up, dan kan in het multidisciplinair overleg van de desbetreffende patiënt worden besloten tot het uitvoeren van een vorm van okselklierbehandeling.
Gevalideerde vragenlijsten worden verstrekt voor het onderzoeken van de invloed op het aantal complicaties en kwaliteit van leven (Lymph-ICF, QLQ-C30, QLQ-BR23) en om te meten of angst en persoonlijkheidstrekken invloed hebben op de uitkomst van kwaliteit van leven (STAI-trait, NEO-FFI). Deze vragenlijsten worden verstrekt aan 700 van de 1644 patiënten (350 per arm), éénmaal voor de randomisatie en vijfmaal na de randomisatie (6 maanden, 1 jaar, 2 jaar, 5 jaar en 10 jaar).

Onderzoekspopulatie:

Vrouwen van 18 jaar of ouder met pathologisch bewezen invasief mammacarcinoom die borstsparende therapie ondergaan.

cT1-2: primaire tumor is bij klinisch onderzoek kleiner dan 5 cm.
cN0: bij lichamelijk onderzoek en echografie van de oksel worden geen aanwijzingen voor lymfekliermetastasen gevonden of de cytologie/histologie na punctie/biopt van een lymfeklier is negatief.
Neoadjuvante systemische therapie is toegestaan indien de patiënt voor de start van de neoadjuvante systemische therapie een klinische T1-2N0 status heeft en waarvoor borstsparende therapie op dat moment al geschikt is.
De patiënt heeft informed consent persoonlijk ondertekend.

Interventie (indien van toepassing):

De controlegroep ondergaat wél de schildwachtklierprocedure volgens de Nederlands borstkankerrichtlijn. De experimentele arm ondergaan géén schildwachtklierprocedure.

Primaire onderzoeksvariabelen/uitkomstmaten:

Primaire uitkomstmaat:
- Regionaal recidief percentage

Secundaire onderzoeksvariabelen/uitkomstmaten (indien van toepassing):
Secundaire uitkomstmaten:
- Distant-disease free survival
- Overall survival
- Later uitgevoerde okselbehandeling
- Lokaal recidief percentage
- Overig-regionaal recidief percentage
- Contralaterale borstkanker
- Percentage (neo)adjuvante systemische therapie
- Kwaliteit van leven en okselbehandeling-gerelateerde morbiditeit

Omschrijving en inschatting van belasting en risico (indien van toepassing):

Wat betreft belasting is er in deze studie géén sprake van extra diagnostiek of interventies (juist minder) en hoeft de patiënt niet vaker dan normaal voor controle naar het ziekenhuis. De eventuele extra belasting voor de patiënt zal alleen bestaan uit het invullen van kwaliteit van leven vragenlijsten (bij 700 van 1644 patiënten) éénmaal voor de randomisatie en vijfmaal na randomisatie (6 maanden, 1 jaar, 2 jaar, 5 jaar en 10 jaar). De hypothese luidt dat het achterwege laten van de schildwachtklierprocedure even veilig is als het wél uitvoeren van deze procedure. Desondanks is er een risico dat in de experimentele arm meer regionale recidieven optreden en dat bij patiënten op een later tijdstip alsnog een vorm van okselklierbehandeling moet worden uitgevoerd. Daartegenover staat dat er in de experimentele arm door het niet uitvoeren van de schildwachtklierprocedure minder kans is op morbiditeit, minder operaties en opnames in het ziekenhuis en een betere kwaliteit van leven. Patiënten zullen op geen enkele manier vertraging ondervinden als gevolg van deelname aan de studie.

K2.Engelse Samenvatting

Background of the study:
The NSABP B-04 trial revealed that omitting primary axillary treatment of occult positive lymph nodes in clinically node negative breast cancer patients does not affect survival, even after 25 years of follow-up and without the use of adjuvant systemic or radiation therapy. A delayed axillary dissection in case lymph nodes become clinically positive does not affect survival and prevents axillary overtreatment in the majority of patients. The ACOSOG Z0011 and IBCSG 23-01 trials revealed that completion axillary dissection can be safely omitted in clinically node negative patients with metastatic sentinel nodes. Patients randomized for watchful waiting were likely to have residual nodal disease (13-27%) that was not surgically removed. Nevertheless, survival rates were not affected and regional recurrence rates low. Use of axillary ultrasound will improve the preoperative selection of node negative patients, as it selects patients with a more favourable tumour load and accurately excludes advanced nodal disease (≥4 metastatic nodes). Biology and adjuvant systemic and radiation therapy are all factors that most likely diminish the risk that possible metastases left in situ develop into clinically detectable lymph nodes. Patients treated with breast conserving therapy are significantly more often diagnosed with pathologic node negative disease or with micrometastatic disease, compared to patients treated with mastectomy. The risk for occult (macro)metastases in cT1-2N0 breast cancer patients treated with breast conserving therapy is low: about 88% will be node negative and about 95% of node positive patients will have no lymph node metastases beyond the sentinel node. Reflected by the low regional recurrence rate after a (false) negative sentinel node, only a small amount of patients (0.8%) with node positive disease is expected to develop clinically detectable nodal disease.

Objective of the study:
The objective of this study is to decrease the number of breast cancer patients receiving overtreatment of the axilla, in order to positively influence the axillary morbidity rate and quality of life. Therefore, we investigate whether the sentinel lymph node procedure can be safely omitted in clinically node negative breast cancer patients undergoing breast conserving therapy, in terms of non-inferior regional control, distant-disease free and overall survival. All objectives are measured during a follow-up of 10 years.

Study design:
A randomized controlled non-inferiority multicenter study. Patients who meet the in- and exclusioncriteria will be randomized after informed consent for one of the two treatment methods:
- Arm A (control arm): lumpectomy with sentinel lymph node procedure, followed by radiotherapy of the breast with or without completion axillary treatment according to the Dutch breast cancer guideline.
- Arm B (experimental arm): lumpectomy without further axillary staging, followed by radiotherapy of the breast.
Stratification: age (≤50, 50≤75, >75 years old), oestrogen receptor status (positive vs. negative), HER2neu status (amplified vs. not amplified), clinical tumour stage prior to any treatment (<3 cm vs. ≥3 cm), grading (grade I-II vs. III - according to Bloom-Richardson grading system), is or will be treated with primary systemic therapy (yes/no) and participating centre.
Adjuvant systemic treatment is administered if indicated according to the Dutch breast cancer guideline.

Primary systemic therapy is allowed if the patient has a clinical T1-2N0 status (initial stage) that is amenable to lumpectomy pre-systemic therapy.
Yearly follow-up with physical examination of the axilla for 10 years. Axillary ultrasound (+/- tissue sampling) on indication. Staging for distant metastases and/or a delayed axillary lymph node dissection if indicated by the multidisciplinary team. Validated questionnaires are used to assess axillary morbidity rate and quality of life (Lymph-ICF, QLQ-C30, QLQ-BR23) and to measure if anxiety and personality traits influence the outcome of quality of life (STAI-trait, NEO-FFI) in 700 of 878 patients. These questionnaires are provided before randomization and sequentially post randomization at 6 months, 1, 2, 5 and 10 years.

Study population:
Women aged 18 years or older with pathologically confirmed invasive breast carcinoma about to undergo breast conserving surgery followed by radiotherapy of the breast.
cT1-2: primary tumour is smaller than 5 cm.
cN0: no signs of axillary lymph node metastases at physical examination and axillary ultrasound (+/- tissue sampling).
Primary systemic treatment is allowed.
Patient is able to and signed the informed consent form.

Intervention (if applicable):
Patients in the control arm will be treated with the sentinel lymph node procedure according to the Dutch breast cancer guideline.
Patients in the experimental arm will not be treated with the sentinel lymph node procedure.

Primary study parameters/outcome of the study:
Primary endpoint:
- Regional recurrence rate

Secundary study parameters/outcome of the study (if applicable):
Secondary endpoints:
- Distant-disease free survival
- Overall survival
- Delayed axillary treatment
- Local recurrence rate
- Other-regional recurrence rate
- Contralateral breast cancer rate
- Percentage difference in the administration of (neo)adjuvant systemic therapy
- Quality of life and axillary morbidity rate

Nature and extent of the burden and risks associated with participation, benefit and group relatedness (if applicable):
There is no extra burden for participating patients as regard to additional diagnostic tests/interventions and follow-up. Any additional burden for the patient will only consist of completing quality of life questionnaires in 700 of 1644 study patients. These questionnaires are provided before randomization and sequentially post randomization at 6 months, 1, 2, 5 and 10 years.
We hypothesize that omitting the sentinel lymph node procedure is not inferior to the standard treatment. Nevertheless, possible disadvantages are a worse regional recurrence rate, and delayed axillary treatment if indicated. Patients in the experimental arm have the possible advantages of less morbidity, fewer operations and hospitalizations and an improved quality of life, compared to patients treated with the sentinel lymph node procedure.
No patient will encounter any delay in their treatment as a result of inclusion.

ONDERTEKENING

De verrichter en indiener verklaren hierbij:
a. het formulier (en samenvatting) volledig en naar waarheid te hebben ingevuld;
b. de antwoorden op de vragen uit het ABR-formulier niet in strijd zijn met het bijbehorende onderzoeksdossier en onderzoekscontract

Naar waarheid getekend, door de verrichter (=opdrachtgever)		door de indiener	
datum	....	datum	....
Handtekening		Handtekening	
naam	M.J.H.M. Jacobs	naam	S.M. van Gastel
functie	Hoofd afdeling Chirurgie, MUMC+	functie	Trialconsulent, IKNL

Sluiten Print

Sluiten Print

Formulier voor medisch-ethische beoordeling en registratie

ABR-formulier, versie december 2015

Onderzoeksdossiernummer

ABR Nummer	**49315**	Status	**Beoordeeld - Positief**
Versie	**03**	Status per	**16-12-2015**
Jaar	**15**		
Dossiernummer	**NL49315.031.14**		
Reden voor	**Toevoeging centra**		

A. Sectie - Openbaar maken gegevens medisch wetenschappelijk onderzoek

A1. Het CCMO-register is een voor ieder toegankelijk openbaar trial register. De antwoorden op de vragen gemarkeerd met een wereldbol en de samenvatting bij dit formulier worden openbaar gemaakt in het CCMO register.

B. Sectie - Administratief

B1. Betreft het onderzoek met geneesmiddelen (inclusief gentherapie, somatische celtherapie, vaccinonderzoek, GGO's, zie verdere toelichting) als bedoeld in de Wet medisch-wetenschappelijk onderzoek met mensen (WMO)?

○ ja
◉ nee

B2. Houdt het onderzoek verband met een eerder door een erkende METC of door de CCMO beoordeelde studie of is het onderzoek reeds eerder bij een erkende METC ter beoordeling voorgelegd?

○ ja, het onderzoek houdt verband met – of is het vervolg op – een eerder beoordeelde studie
○ ja, het onderzoek is eerder ter beoordeling aan een erkende METC of de CCMO voorgelegd (stuur kopie besluit mee)
◉ nee

B4. Is het protocol (nog) in een ander openbaar trial register geregistreerd?

○ ja
◉ nee

B5. Naam indiener/contactpersoon voor de oordelende toetsingscommissie

**B5a.**	Achternaam indiener/contactpersoon	Hage
	Titel en voorletters	dr. J.A.
	Tussenvoegsels	van der
**B5b.**	Type organisatie/bedrijf	Overige Ziekenhuizen
	Organisatie/bedrijf	Antoni van Leeuwenhoek Ziekenhuis
	Naam organisatie/bedrijf	IKNL trialbureau
	Afdeling	Chirurgie
	Adres	Plesmanlaan 121
	Postcode en plaats	1066 CX Amsterdam
	Land	NL
**B5c.**	Intern adres	afdeling Heelkunde
	Telefoon	020 512 9111
	Fax	020 512 2554
	E-mail	j.vd.hage@nki.nl

B6. Is de indiener werkzaam bij de opdrachtgever/sponsor (verrichter) van het onderzoek?

○ Ja ◉ Nee

B7. Opdrachtgever/sponsor van het onderzoek (verrichter volgens de WMO)

Type Organisatie/Bedrijf	Universitair Medisch Centrum
Organisatie/Bedrijf	Medisch Universitair Ziekenhuis Maastricht

Adres	P. Debyelaan 25
Postcode en plaats	6229 HX Maastricht
Land	NL
Telefoon	0433877477
Fax	0433875473
E-mail	m.smidt@mumc.nl

C. Sectie - Onderzoek

C1. Volledige titel van het onderzoek
C1a. In het Engels
Clinically node negative breast cancer patients undergoing breast conserving therapy: Sentinel lymph node procedure versus follow-up. A Dutch randomized controlled multicentre trial.

C1b. In het Nederlands
Patiënten met borstkanker en een klinisch negatieve okselklierstatus die een borstsparende behandeling ondergaan: Schildwachtklierprocedure versus follow-up. Een Nederlandse gerandomiseerde multicenter studie.

C2. Verkorte titel van het onderzoek/acroniem
C2a. In het Engels
BOOG 2013-08

C2b. In het Nederlands (Let op: deze korte titel wordt vermeld binnen ToetsingOnline)
BOOG 2013-08

C3. Trefwoorden (maximaal 4, plaats elk trefwoord op een aparte regel)
C3a. In het Engels
Breast neoplasms
Breast conserving therapy
Sentinel lymph node biopsy
Axilla

C3b. In het Nederlands
Borstkanker
Borstsparende behandeling
Schildwachtklierprocedure
Oksel

C4. Beschrijf het belang van het onderzoek en de beoogde toepassing van de resultaten (verwijs eventueel naar de relevante pagina's in het protocol).
Het doel van dit onderzoek is om:
1) Overbehandeling van de oksel te verminderen;
2) Het aantal korte en lange termijn complicaties te verminderen;
3) De kwaliteit van leven te verbeteren;
4) Met een even goede ziektecontrole en algehele overleving

Dit willen wij bereiken door te onderzoeken of de schildwachtklierprocedure veilig achterwege gelaten kan worden bij patiënten met borstkanker en een klinisch negatieve okselklierstatus behandeld met borstsparende therapie.

C6. Betreft het onderzoek een multicenter-onderzoek?
○ nee
◉ ja - alleen in Nederland
○ ja - internationaal binnen de Europese Unie
○ ja - internationaal ook buiten de Europese Unie

C7. Is er bij multicenter-onderzoek sprake van een coördinerend onderzoeker?
◉ ja namelijk
○ nee
Naam onderzoeker:
M.L. Smidt

C8. Wie is/zijn medisch verantwoordelijk voor de proefpersonen die deelnemen aan het onderzoek
Behandelend arts en lokale hoofdonderzoeker

C9. In welk centrum/welke centra (incl. huisartsenpraktijken) in Nederland wordt het onderzoek uitgevoerd?

Centrum	Proefpersonen	Hoofdonderzoeker	Onafhankelijk arts
Albert Schweitzer Ziekenhuis	49	M.B.E. Menke-Pluijmers	S.O. Breukink (Maastricht UMC+)
Amphia Ziekenhuis	69	E.J.T. Luiten	S.O. Breukink (Maastricht UMC+)

Antonius Ziekenhuis, Sneek	56	C.G.J.M. van Riel	S.O. Breukink (Maastricht UMC+)
Atrium Medisch Centrum	39	E.R.M. van Haaren	S.O. Breukink (Maastricht UMC+)
Bronovo Ziekenhuis	23	O.R. Guicherit	S.O. Breukink (Maastricht UMC+)
Canisius Wilhelmina Ziekenhuis	46	L.J.A. Strobbe	S.O. Breukink (Maastricht UMC+)
Catharina-ziekenhuis	46	G.A.P. Nieuwenhuijzen	S.O. Breukink (Maastricht UMC+)
Deventer Ziekenhuis	39	H. de Vries	S.O. Breukink (Maastricht UMC+)
Zorggroep De Sionsberg	15	K. Blaauwendraat	S.O. Breukink (Maastricht UMC+)
Diakonessenhuis Utrecht	39	T. van Dalen	S.O. Breukink (Maastricht UMC+)
Gelre Ziekenhuizen	37	W.B. Lastdrager	S.O. Breukink (Maastricht UMC+)
Groene Hart Ziekenhuis	31	E. Linthorst	S.O. Breukink (Maastricht UMC+)
HagaZiekenhuis	41	I. Jannink	S.O. Breukink (Maastricht UMC+)
Ikazia Ziekenhuis	27	B.R. Toorenvliet	S.O. Breukink (Maastricht UMC+)
Isala Klinieken	54	A.B. Francken	S.O. Breukink (Maastricht UMC+)
Kennemer Gasthuis	43	R.J.C.L.M. Vuylsteke	S.O. Breukink (Maastricht UMC+)
LangeLand Ziekenhuis	14	B. Jas	S.O. Breukink (Maastricht UMC+)
Laurentius Ziekenhuis	23	A.V.R.J. Bell	S.O. Breukink (Maastricht UMC+)
Academisch Ziekenhuis Maastricht	39	M.L. Smidt	S.O. Breukink (Maastricht UMC+)
Martini Ziekenhuis	43	W. Kelder	S.O. Breukink (Maastricht UMC+)
Maxima Medisch Centrum	31	K. Schenk	S.O. Breukink (Maastricht UMC+)
Meander Medisch Centrum	43	A.J. van Overbeeke	S.O. Breukink (Maastricht UMC+)
Antoni van Leeuwenhoek Ziekenhuis	93	J.A. van der Hage	S.O. Breukink (Maastricht UMC+)
Onze Lieve Vrouwe Gasthuis	28	P.J. Borgstein	S.O. Breukink (Maastricht UMC+)
Orbis Medisch Centrum	23	Y. Vissers	S.O. Breukink (Maastricht UMC+)
Universitair Medisch Centrum Sint Radboud	31	J.H.W. de Wilt	S.O. Breukink (Maastricht UMC+)
Reinier de Graaf Groep	46	M. van 't Riet	S.O. Breukink (Maastricht UMC+)
Sint Antonius Ziekenhuis	58	R. Koelemij	S.O. Breukink (Maastricht UMC+)
Sint Elisabeth Ziekenhuis	37	P. Jansen	S.O. Breukink (Maastricht UMC+)
Streekziekenhuis Koningin Beatrix	19	A. Jonk	S.O. Breukink (Maastricht UMC+)
Tergooiziekenhuizen locatie Blaricum	42	E.J.C. Vriens	S.O. Breukink (Maastricht UMC+)
Universitair Medisch Centrum Groningen	17	L. Jansen	S.O. Breukink (Maastricht UMC+)
Universitair Medisch Centrum Utrecht	23	A.J. Witkamp	S.O. Breukink (Maastricht UMC+)
Viecuri Medisch Centrum voor Noord-Limburg	39	C.L.H. van Berlo	S.O. Breukink (Maastricht UMC+)
Westfries Gasthuis	27	J.W.D. de Waard	S.O. Breukink (Maastricht UMC+)
Waterland Ziekenhuis	22	L. de Widt	S.O. Breukink (Maastricht UMC+)
Zaans Medisch Centrum	15	S. Muller	S.O. Breukink (Maastricht UMC+)
Ziekenhuisvoorzieningen Gelderse Vallei	32	W.K. de Roos	S.O. Breukink (Maastricht UMC+)
Ziekenhuisgroep Twente	31	D. de Leeuw	S.O. Breukink (Maastricht UMC+)

Rivierenland Ziekenhuis	23	M.A.J. de Roos	S.O. Breukink (Maastricht UMC+)
Medisch Centrum Alkmaar	54	W.H. Schreurs	S.O. Breukink (Maastricht UMC+)
Medisch Centrum Haaglanden	31	A. Marinelli	S.O. Breukink (Maastricht UMC+)
Medisch Spectrum Twente	46	P. Steenvoorde	S.O. Breukink (Maastricht UMC+)
Rijnstate Ziekenhuis	62	R. van Eekeren	S.O. Breukink (Maastricht UMC+)
Flevoziekenhuis	50	S. van der Meij	S.O. Breukink (Maastricht UMC+)
Zuwehofpoort	15	R.C. van Doorn	S.O. Breukink (Maastricht UMC+)

C10. Betreft het onderzoek met:

- [x] mensen
- [ ] geslachtscellen
- [ ] (rest-)embryo's
- [ ] foetussen in utero

C11. Beoogd totaal aantal proefpersonen/(rest)embryo's/foetussen in utero:
C11a. In Nederland
1644

C13. Onderzoeksgebied

- [ ] etiologie
- [ ] organisatorisch/zorgonderzoek
- [ ] diagnostiek
- [ ] preventie
- [x] therapie
- [ ] veiligheid
- [ ] werkzaamheid
- [ ] farmacokinetiek
- [ ] farmacodynamiek
- [ ] bio-equivalentie
- [ ] dosis-respons
- [ ] farmacogenomics
- [ ] farmaco-economie
- [ ] anders

C14. Type onderzoek

- ( ) observationeel onderzoek zonder invasieve metingen
- ( ) observationeel onderzoek met invasieve metingen
- (●) interventie-onderzoek

C15. In welke fase kan het onderzoek worden ingedeeld?

- ( ) fase I (a)
- ( ) fase II (b)
- (●) fase III (c)
- ( ) fase IV (d)
- ( ) overige onderzoeken waarbij geneesmiddelen worden toegepast (e)
- ( ) niet van toepassing

C17. Is er sprake van een andere interventie dan met geneesmiddelen zoals bedoeld in de Wet medisch-wetenschappelijk onderzoek met mensen (WMO) (zie toelichting)?

- (●) Ja
- ( ) Nee

C17a. [ ] Medisch hulpmiddel, namelijk
C17b. [ ] Operatie, namelijk

C17c. ☐ Psychosociale interventie, namelijk
C17d. ☐ Voeding(stoffen), namelijk
C17e. ☐ Bewegingstherapie, namelijk
C17f. ☐ interventie met radioactieve straling, namelijk
C17g. ☐ Blootstellingsonderzoek (bijv pesticidenonderzoek), namelijk
C17h.
C17i. ☑ andere interventie, namelijk

In het Engels: No sentinel lymph node procedure
In het Nederlands: Géén schildwachtklierprocedure

C18. Worden de onderzoeksproducten voor deze studie door de verrichter gratis verstrekt?

- ○ ja
- ○ nee
- ○ gedeeltelijk namelijk
- ● niet van toepassing

C19. Is/zijn er (een) controlegroep (en)?

- ● ja
- ○ nee

- ☐ ja, geneesmiddel
- ☐ ja, placebo
- ☐ ja, geen interventie
- ☑ ja, anders

namelijk De standaard behandeling: schildwachtklierprocedure

C20. Betreft het een gerandomiseerd onderzoek?

- ● ja
- ○ nee

namelijk

- ☑ open
- ☐ enkelblind
- ☐ dubbelblind
- ☐ parallel
- ☐ cross-over
- ☐ anders namelijk

C21. Op welke klasse(n) van aandoeningen heeft het onderzoek betrekking (maximaal 3)

- ☐ hartaandoeningen
- ☐ congenitale, familiaire en genetische aandoeningen
- ☐ bloed- en lymfestelsel aandoeningen
- ☐ zenuwstelsel aandoeningen
- ☐ oogaandoeningen
- ☐ evenwichtsorgaan- en ooraandoeningen
- ☐ ademhalingsstelsel-, thorax- en mediastinumaandoeningen
- ☐ maagdarmstelselaandoeningen
- ☐ nier- en urinewegaandoeningen
- ☐ huid- en onderhuidaandoeningen
- ☐ skeletspierstelsel- en bindweefselaandoeningen
- ☐ endocriene aandoeningen
- ☐ voedingsstoornissen en metabole ziekten
- ☐ infecties en parasitaire aandoeningen
- ☐ letsels, intoxicaties en verrichtingscomplicaties
- ☑ neoplasmata, benigne, maligne en niet-gespecificeerd (incl cysten en poliepen)

borstneoplasmata maligne en niet-gespecificeerd (incl. tepel)

- [x] chirurgische en medische verrichtingen
  borst therapeutische verrichtingen
- [ ] bloedvataandoeningen
- [ ] algemene aandoeningen en aandoeningen op de plek van toediening
- [ ] zwangerschap, perinatale periode en puerperium
- [ ] sociale omstandigheden
- [ ] immuunsysteemaandoeningen
- [ ] lever- en galaandoeningen
- [ ] voortplantingsstelsel- en borstaandoeningen
- [ ] psychische stoornissen
- [ ] overig, namelijk

C22.Geef twee synoniemen voor de aandoening die bestudeerd wordt, waarvan tenminste één lekenterm.

In het Engels:	breast cancer, sentinel lymph nodes, sentinel lymph node metastases
In het Nederlands:	borstkanker, schildwachtklieren, schildwachtkliermetastasen

C23. Beoogde start- en einddatum van het onderzoek

C23a. Start Datum (dd-mm-jjjj)	01-01-2015
C23b. Eind Datum (dd-mm-jjjj)	01-01-2027

C24. Zijn patiënten betrokken bij het ontwikkelen van het onderzoek? (Voor patiënten lees ook: patiëntenorganisaties, naasten of nabestaanden van patiënten en gezonde vrijwilligers)?

C30. Wordt de ervaring van proefpersonen na deelname aan het onderzoek geëvalueerd?

D. Sectie - Proefpersonen

D1. Is er een proefpersonenverzekering conform de WMO-eisen afgesloten of wordt aan de oordelende toetsingscommissie ontheffing gevraagd?

- (●) proefpersonenverzekering is afgesloten bij verzekeringsmaatschappij
- ( ) ontheffing van de verzekering wordt gevraagd
- ( ) niet van toepassing - onderzoek valt onder de Embryowet en niet onder de WMO

Verzekeringsmaatschappij	Marketform (onderdeel van Lloyd's)

D2. Gezonde proefpersonen en/of patiënten

- [ ] Gezonde proefpersonen

- [x] Patiënten

Aantal	1644

D4. Voornaamste inclusiecriteria

D4a. In het Engels

- Female
- Aged 18 years or older
- Pathologically confirmed invasive unilateral breast carcinoma
- A clinical T1-2 tumour (≤ 5cm)
- Will be treated with lumpectomy followed by whole breast radiotherapy
- Clinically node negative status: no signs of axillary lymph node metastases at physical examination and preoperative axillary ultrasound (or negative cyto-/histopathology)
- Written informed consent

D4b. In het Nederlands

- Vrouw
- Leeftijd 18 jaar of ouder
- Pathologisch bewezen invasief unilaterale borstkanker
- Klinisch T1-2 tumor (≤ 5cm)
- Zal worden behandeld met lumpectomie gevolgd door gehele borstbestraling
- Klinisch negatieve okselklierstatus: negatief lichamelijk onderzoek en echografie van de oksel (of negatieve cyto-/histologie)
- Schriftelijke informed consent

D5. Voornaamste exclusiecriteria

D5a. In het Engels

- Clinically node positive pre-operative
- Bilateral breast cancer

- Evidence of metastatic disease
- History of invasive breast cancer
- Previous treatment of the ipsilateral axilla with surgery or radiotherapy, except surgery for hidradenitis suppurativa or for other superficially located skin lesions, such as naevi
- Pregnant or nursing
- Other prior malignancies within the past 5 years, except successfully treated malignancies that occurred more than five years before randomization, and except successfully treated basal cell and squamous cell skin cancer, carcinoma in situ of the cervix or carcinoma in situ of the ipsilateral or contralateral breast
- Unable or unwilling to give informed consent

D5b. In het Nederlands

- Klinisch positieve okselklierstatus (preoperatief)
- Bilaterale borstkanker
- Primair (afstands-)gemetastaseerde borstkanker
- Invasieve borstkanker in de voorgeschiedenis
- Chirurgie of radiotherapie van ipsilaterale oksel in de voorgeschiedenis, behalve chirurgie voor hidradenitis suppurativa of andere oppervlakkig gelegen huidlaesies, zoals naevi
- Zwanger of lacterend
- Andere maligniteit in de afgelopen 5 jaar, behalve succesvol behandelde maligniteit die meer dan vijf jaar voor randomisatie optrad, en behalve succesvol behandelde huidkanker van type basaalcel of plaveiselcel, carcinoma in situ van de cervix, carcinoma in situ van de ipsilaterale of contralaterale mamma
- Niet in staat of niet bereid tot informed consent

D6. Bij welke categorie proefpersonen wordt het onderzoek uitgevoerd (meerdere antwoorden mogelijk)

- [x] 18 jaar of ouder en wilsbekwaam
- [ ] 18 jaar of ouder en wilsonbekwaam
- [ ] 12 t/m 17 jaar en in staat tot het geven van geïnformeerde toestemming
- [ ] 12 t/m 17 jaar en niet in staat tot het geven van geïnformeerde toestemming (wilsonbekwaam)
- [ ] jonger dan 12 jaar

D10. Verkeren (sommige) proefpersonen in een afhankelijkheidssituatie ten opzichte van de onderzoeker of degene die de deelnemers werft? (lees de toelichting voor voorbeelden wanneer er sprake kan zijn van een afhankelijkheidssituatie)

- (●) ja
- ( ) nee

D10a. Zo ja, waardoor?

Soms is de lokale onderzoeker ook de behandelend arts die de patiënt vraagt om deel te nemen aan de studie.

D10b. Waarom wordt het onderzoek juist met deze proefpersonen uitgevoerd en hoe worden de belangen van de proefpersonen gewaarborgd?

Het onderzoek moet met proefpersonen worden uitgevoerd die voldoen aan de beschreven in/exclusie criteria zodat de resultaten van de studie uiteindelijk ook naar de kliniek vertaald kunnen worden binnen deze patiëntenpopulatie. De belangen van de proefpersonen worden gewaarborgd door de geldende medisch ethische normen en richtlijn zoals vermeld in het protocol (o.a. onafhankelijk arts, uitgebreide patiëntinformatiefolder).

D11. Waaruit bestaat de vergoeding voor de proefpersonen?

- [x] geen vergoeding
- [ ] reiskosten
- [ ] financiële vergoeding (in Euro's)
- [ ] andere vergoeding

D12. Is deze vergoeding afhankelijk van bepaalde voorwaarden, bijvoorbeeld het voltooien van (een deel van) het onderzoek?

- ( ) ja (motiveer)
- ( ) nee
- (●) niet van toepassing

E. Sectie - Voor- en nadelen

E1. Wordt er bij dit onderzoek een rechtstreeks therapeutisch effect beoogd bij de proefpersonen / patiënten?

- (●) ja (therapeutisch onderzoek)
- ( ) nee (niet-therapeutisch onderzoek)

E1a. Zo ja, geef kort aan waaruit dit therapeutisch effect bestaat.

Het therapeutische effect bestaat uit:
- Het verlagen van het risico op okselbehandeling-gerelateerde korte en lange termijn complicaties;
- Het verbeteren van de kwaliteit van leven;
- Met een even goede ziektecontrole en algehele overleving.

E2. Waaruit bestaat de belasting van het onderzoek (en een eventueel daaraan voorafgaande keuring) voor de proefpersonen?

Tijdbeslag	per bezoek	20 min per keer voor invullen kwaliteit van leven vragenlijst
	totaal	20 min x 6 = 120 min
	totale duur van de studie voor de individuele proefpersoon	Tav de behandeling minder belasting dan normaal voor de studie-arm (geen schildwachtklierprocedure). Voor beide armen is extra het invullen van de kwaliteit van leven vragenlijst prerandomisatie en postrandomisatie op 5 momenten.

E3. Worden de proefpersonen in verband met het onderzoek in het ziekenhuis opgenomen of wordt een opname verlengd?

- ○ ja - het verblijf in het ziekenhuis/instituut wordt in verband met het onderzoek verlengd
- ○ ja - ze worden voor het onderzoek in het ziekenhuis/instituut opgenomen
- ◉ nee

E4. Beschrijf in hoeverre proefpersonen worden onderworpen aan handelingen dan wel een gedragswijze krijgen opgelegd, zoals vragenlijst, interviews, lichamelijk/psychologisch onderzoek, ontzegging, dieet (voor invasieve ingrepen: zie vraag E6)

In totaal zullen 700 proefpersonen (350 in de experimentele en 350 in de controlegroep) deelnemen aan het kwaliteit van leven onderzoek, bestaande uit een vragenlijst voor de randomisatie en op 5 tijdstippen na de randomisatie (na een half jaar, één, twee, vijf en 10 jaar).

E5. Worden de proefpersonen getest op bepaalde aandoeningen/condities?

- ○ ja (motiveer)
- ◉ nee

E6. Welke extra (invasieve) ingrepen (anders dan bij de standaard behandeling) moeten de proefpersonen in het kader van het onderzoek ondergaan:

- ☑ Niet van toepassing

E9. Geef aan welke risico's er voor proefpersonen zijn verbonden aan deelname aan het onderzoek.

Hoewel de hypothese luidt dat het achterwege laten van de schildwachtklierprocedure geen nadelige invloed heeft op de lokale controle van de ziekte en de overlevingskans, bestaat er toch een kans dat proefpersonen in de experimentele arm een hoger risico hebben op een recidief in de oksel (regionaal recidief) en daardoor tijdens follow-up alsnog een vorm van okselklierbehandeling moeten ondergaan. Uit onderzoek blijkt dat het uitgesteld uitvoeren van okselklierbehandeling (bijvoorbeeld met okselklierdissectie) géén nadelige gevolgen heeft voor de overleving.

E9a. Geef op grond van uw eigen afweging aan waarom het uitvoeren van het onderzoek, in het licht van de belasting en/of risico's die voor proefpersonen aan deelname verbonden zijn, gerechtvaardigd is?

Het mogelijk hogere risico op een recidief in de oksel (regionaal recidief) weegt op tegen de lagere kans op ernstige okselklierbehandeling-gerelateerde morbiditeit (lymfoedeem, bewegingsbeperking in de schouder, arm- en schouderpijn, verminderd gevoel of zenuwpijn) en de verbetering van kwaliteit van leven.

E10. Indien het onderzoek bij minderjarige en/of wilsonbekwame proefpersonen wordt uitgevoerd en geen direct therapeutisch effect wordt beoogd: waarom kunnen belasting en risico's als minimaal worden beschouwd? (Verwijs eventueel naar de relevante pagina's in het protocol.

- ☑ niet van toepassing

E11. Kan de eventuele therapie na beëindiging van het onderzoek worden voortgezet?

- ○ ja (motiveer)
- ○ nee (motiveer)
- ◉ niet van toepassing

E12. Heeft deelname aan het onderzoek voor de proefpersoon tot gevolg dat van de standaardbehandeling of -diagnostiek kan worden afgeweken of deze kan worden uitgesteld?

- ◉ ja
- ○ nee
- ○ niet van toepassing

E12a. Zo ja, waaruit bestaat de afwijking of het uitstel en waarom is afwijking/uitstel

Proefpersonen in de controlegroep ondergaan de standaard behandeling, namelijk schildwachtklierprocedure volgens de Nederlandse borstkankerrichtlijn. Proefpersonen in de experimentele arm ondergaan primair géén schildwachtklierprocedure. Beide groepen ondergaan dezelfde follow-up middels lichamelijk onderzoek en

aanvullend onderzoek op indicatie. Indien een proefpersoon zich in de follow-up periode presenteert met lymfekliermetastasen, kan alsnog besloten worden om een vorm van okselklierbehandeling uit te voeren. Deze beslissing wordt gemaakt tijdens het multidisciplinair overleg in het desbetreffende centrum waar de patiënt onder behandeling staat.

F. Sectie - Informatie en privacy

F1. Hoe worden de proefpersonen geworven en door wie (onderzoeker, behandelend arts, andere persoon) wordt de proefpersoon/wettelijke vertegenwoordiger geïnformeerd en om toestemming gevraagd?

Op het moment in de behandeling van borstkanker dat de patiënt voldoet aan de in/exclusiecriteria zal de behandeld arts informatie geven over de mogelijkheid tot deelname aan deze studie. Een schriftelijke patiëntinformatiebrief zal worden meegegeven aan de patiënt. De patiënt krijgt ruim de tijd (ten minste één week) om deze informatie thuis na te lezen en een beslissing te nemen. De patiënt kan haar interesse in deelname kenbaar maken aan haar behandelend arts bij een volgend polibezoek. De behandelend arts zal haar dan om toestemming vragen voor deelname aan de studie.

F2. Hoeveel bedenktijd krijgen de proefpersonen/wettelijke vertegenwoordigers om te beslissen over deelname?

Zo lang als nodig, met ten minste één week

F3. Wordt de huisarts, behandelend specialist en/of apotheker van de proefpersoon geïnformeerd over diens deelname aan het onderzoek?

- (●) ja (de proefpersoon dient hiervoor toestemming te geven)
- ( ) nee

F4. Worden persoonsgegevens gecodeerd?

- (●) ja
- ( ) nee

F4a. Zo ja, hoe is deze codering opgebouwd?

Alle proefpersonen krijgen een uniek studienummer. Het unieke studienummer betreft een opvolgnummer zonder verdere specifieke codering voor bijvoorbeeld centrum.

F4b. Wie heeft toegang tot de sleutel van deze code?

De behandelend arts en de onderzoeker

F4c. Wie hebben toegang tot de brondocumenten en eventuele andere tot de persoon herleidbare gegevens?

De lokaal datamanagers en de onderzoeker

F5. Hoe wordt het lichaamsmateriaal gedurende het onderzoek bewaard?

- ( ) in tot de proefpersoon herleidbare vorm (gecodeerd)
- ( ) in niet tot de proefpersoon herleidbare vorm (volledig geanonimiseerd)
- (●) niet van toepassing

F6. Wordt afgenomen lichaamsmateriaal na afloop van het onderzoek vernietigd?

- ( ) ja
- ( ) nee (motiveer)
- (●) niet van toepassing

F7. Kunnen proefpersonen na afloop van het onderzoek opnieuw benaderd worden (bijvoorbeeld voor nader onderzoek of follow-up)?

- ( ) ja
- (●) nee

G. Sectie - Financieel

G1. Door welke geldstroom wordt het onderzoek gefinancierd?

- [ ] eerste geldstroom (Geld van Ministerie van OC&W aan universiteiten)
- [ ] tweede geldstroom (NWO of KNAW), namelijk
- [x] derde geldstroom (anders dan 1e of 2e geldstroom, zoals collectebusfondsen, Europese Unie, vakministeries of bedrijven), namelijk
  KWF Kankerbestrijding

G2. Wordt het onderzoek (mede) gefinancierd door de industrie/bedrijven?

- ( ) ja - door de industrie/bedrijf zoals is opgegeven bij vraag B6/B7 (opdrachtgever van het onderzoek)
- ( ) ja - (ook) door andere industrie/bedrijven dan de opdrachtgever
- (●) nee

G3. Wat is de hoogte van de vergoeding die de arts/onderzoeker cq onderzoeksafdeling/maatschap ontvangt voor de uitvoering van het onderzoek?

○ Per patiënt of proefpersoon
◉ Per deelnemend centrum

bedrag afgerond in hele euro's: € 100,00

G3a. Hoe is de vergoeding opgebouwd?

De vergoeding betreft een financiële compensatie van € 100 excl. BTW als opstartvergoeding voor ieder deelnemend centrum.

G4. Heeft/hebben de onderzoeker(s) gedurende de afgelopen vijf jaar op een of andere wijze een persoonlijke financiële relatie (gehad) met de verrichter/sponsor van het huidige onderzoek?

○ ja (licht toe)
◉ nee

I. Sectie - Indiening en beoordeling

I1. Sla het formulier eerst op en selecteer vervolgens een METC of de CCMO

Commissie
METC NedMec

J. Sectie - Aanvullende opmerkingen

Aanvullende opmerkingen

K. Sectie - Samenvatting

Achtergrond van het onderzoek:

De schildwachtklierprocedure is de standaard methode voor lymfeklierstadiëring in borstkankerpatiënten met een klinisch negatieve okselklierstatus. In de algemene bevolking bevat de schildwachtklier in ongeveer 25% een uitzaaiing. Dit percentage is lager in patiënten met een kleine borsttumor en in borstsparend behandelde patiënten. De ACOSOG-Z0011 studie en de IBCSG 23-01 studie hebben recent aangetoond dat een completerende okselklierdissectie veilig achterwege gelaten kan worden bij borstsparend en met systemische therapie behandelde patiënten met een beperkt aantal schildwachtkliermetastasen. Dit roept de vraag op of de schildwachtklierprocedure nog wel noodzakelijk is voor borstsparend behandelde patiënten met een klinisch negatieve okselklierstatus. De klinisch negatieve okselklierstatus van de patiënten in de beschreven studies werd gesteld op basis van lichamelijk onderzoek. Het toevoegen van een preoperatieve echografie van de oksel zal zorgen voor een betere voorselectie van patiënten zonder lymfekliermetastasen, ten opzichte van lichamelijk onderzoek alleen. Daarbij sluit een negatieve echografie uitgevoerd in Nederland bijna alle patiënten uit met uitgebreide lymfekliermetastasen (≥4 lymfekliermetastasen). Bovendien omvat gehele borstbestraling na de borstsparende operatie in het algemeen het lagere deel van de oksel en behandelt daarmee eventueel achtergebleven uitzaaiingen. Hoewel de schildwachtklierprocedure minder invasief is vergeleken met een okselklierdissectie, komen complicaties nog steeds voor. Zo lijdt 6% van de patiënten die alleen een schildwachtklierprocedure ondergingen aan ernstige lymfoedeem na slechts 3 jaar. Deze studie gaat onderzoeken of de schildwachtklierprocedure veilig achterwege gelaten kan worden bij borstsparend behandelde patiënten, met een negatieve echografie van de oksel.

Doel van het onderzoek:

Het doel van deze studie is het verminderen van overbehandeling van de oksel bij patiënten met borstkanker en een klinisch negatieve okselklierstatus die behandeld worden met borstsparende therapie, door het achterwege laten van de schildwachtklierprocedure, om zodoende de kans op korte en lange termijn complicaties te verminderen en te zorgen voor een verbetering van de kwaliteit van leven met een even goede ziektecontrole en algehele overleving.

Onderzoeksopzet:

Het betreft een gerandomiseerde non-inferiority multicenter onderzoek. Patienten die voldoen aan de in- en exclusiecriteria worden na toestemming middels loting behandeld volgens één van de volgende behandelmethoden:

- Behandelmethode A (controle groep): schildwachtklierprocedure volgens de Nederlandse borstkankerrichtlijn.
- Behandelmethode B (studie groep): geen verdere okselklierstadiëring.

Stratificatiefactoren: leeftijd (≤50, 50≤75, >75 jaar), oestrogeen receptor status (positief vs. negatief), HER2neu status (geamplificeerd vs. niet geamplificeerd), klinisch tumor stadium (<3 cm vs. ≥3 cm), tumorgraad volgens gemodificeerde Bloom-Richardson (graad I-II vs. graad III), is of zal worden behandeld met primaire systemische therapie (ja/nee), participerend centrum.
Overige behandelingen (o.a. hormonale therapie, chemotherapie) worden op indicatie gegeven volgens de Nederlandse borstkankerrichtlijn.
Tijdens de follow-up periode van 10 jaar wordt de oksel nauwkeurig onderzocht middels lichamelijk onderzoek en echografie op indicatie. Indien sprake is van een cyto-/histologisch bewezen lymfekliermetastase tijdens follow-up, dan kan in het multidisciplinair overleg van de desbetreffende patiënt worden besloten tot het uitvoeren van een vorm van okselklierbehandeling.
Gevalideerde vragenlijsten worden verstrekt voor het onderzoeken van de invloed op het aantal complicaties en kwaliteit van leven (Lymph-ICF, QLQ-C30, QLQ-BR23) en om te meten of angst en persoonlijkheidstrekken invloed hebben op de uitkomst van kwaliteit van leven (STAI-trait, NEO-FFI). Deze vragenlijsten worden verstrekt aan 700 van de 1644 patiënten (350 per arm), éénmaal voor de randomisatie en vijfmaal na de randomisatie (6 maanden, 1 jaar, 2 jaar, 5 jaar en 10 jaar).

Onderzoekspopulatie:

Vrouwen van 18 jaar of ouder met pathologisch bewezen invasief mammacarcinoom die borstsparende therapie ondergaan.

cT1-2: primaire tumor is bij klinisch onderzoek kleiner dan 5 cm.
cN0: bij lichamelijk onderzoek en echografie van de oksel worden geen aanwijzingen voor lymfekliermetastasen gevonden of de cytologie/histologie na punctie/biopt van een lymfeklier is negatief.
Neoadjuvante systemische therapie is toegestaan indien de patiënt voor de start van de neoadjuvante systemische therapie een klinische T1-2N0 status heeft en waarvoor borstsparende therapie op dat moment al geschikt is.
De patiënt heeft informed consent persoonlijk ondertekend.

Interventie (indien van toepassing):

De controlegroep ondergaat wél de schildwachtklierprocedure volgens de Nederlands borstkankerrichtlijn. De experimentele arm ondergaan géén schildwachtklierprocedure.

Primaire onderzoeksvariabelen/uitkomstmaten:

Primaire uitkomstmaat:
- Regionaal recidief percentage

Secundaire onderzoeksvariabelen/uitkomstmaten (indien van toepassing):
Secundaire uitkomstmaten:
- Distant-disease free survival
- Overall survival
- Later uitgevoerde okselbehandeling
- Lokaal recidief percentage
- Overig-regionaal recidief percentage
- Contralaterale borstkanker
- Percentage (neo)adjuvante systemische therapie
- Kwaliteit van leven en okselbehandeling-gerelateerde morbiditeit

Omschrijving en inschatting van belasting en risico (indien van toepassing):

Wat betreft belasting is er in deze studie géén sprake van extra diagnostiek of interventies (juist minder) en hoeft de patiënt niet vaker dan normaal voor controle naar het ziekenhuis. De eventuele extra belasting voor de patiënt zal alleen bestaan uit het invullen van kwaliteit van leven vragenlijsten (bij 700 van 1644 patiënten) éénmaal voor de randomisatie en vijfmaal na randomisatie (6 maanden, 1 jaar, 2 jaar, 5 jaar en 10 jaar). De hypothese luidt dat het achterwege laten van de schildwachtklierprocedure even veilig is als het wél uitvoeren van deze procedure. Desondanks is er een risico dat in de experimentele arm meer regionale recidieven optreden en dat bij patiënten op een later tijdstip alsnog een vorm van okselklierbehandeling moet worden uitgevoerd. Daartegenover staat dat er in de experimentele arm door het niet uitvoeren van de schildwachtklierprocedure minder kans is op morbiditeit, minder operaties en opnames in het ziekenhuis en een betere kwaliteit van leven. Patiënten zullen op geen enkele manier vertraging ondervinden als gevolg van deelname aan de studie.

K2.Engelse Samenvatting

Background of the study:
The NSABP B-04 trial revealed that omitting primary axillary treatment of occult positive lymph nodes in clinically node negative breast cancer patients does not affect survival, even after 25 years of follow-up and without the use of adjuvant systemic or radiation therapy. A delayed axillary dissection in case lymph nodes become clinically positive does not affect survival and prevents axillary overtreatment in the majority of patients. The ACOSOG Z0011 and IBCSG 23-01 trials revealed that completion axillary dissection can be safely omitted in clinically node negative patients with metastatic sentinel nodes. Patients randomized for watchful waiting were likely to have residual nodal disease (13-27%) that was not surgically removed. Nevertheless, survival rates were not affected and regional recurrence rates low. Use of axillary ultrasound will improve the preoperative selection of node negative patients, as it selects patients with a more favourable tumour load and accurately excludes advanced nodal disease (≥4 metastatic nodes). Biology and adjuvant systemic and radiation therapy are all factors that most likely diminish the risk that possible metastases left in situ develop into clinically detectable lymph nodes. Patients treated with breast conserving therapy are significantly more often diagnosed with pathologic node negative disease or with micrometastatic disease, compared to patients treated with mastectomy. The risk for occult (macro)metastases in cT1-2N0 breast cancer patients treated with breast conserving therapy is low: about 88% will be node negative and about 95% of node positive patients will have no lymph node metastases beyond the sentinel node. Reflected by the low regional recurrence rate after a (false) negative sentinel node, only a small amount of patients (0.8%) with node positive disease is expected to develop clinically detectable nodal disease.

Objective of the study:
The objective of this study is to decrease the number of breast cancer patients receiving overtreatment of the axilla, in order to positively influence the axillary morbidity rate and quality of life. Therefore, we investigate whether the sentinel lymph node procedure can be safely omitted in clinically node negative breast cancer patients undergoing breast conserving therapy, in terms of non-inferior regional control, distant-disease free and overall survival. All objectives are measured during a follow-up of 10 years.

Study design:
A randomized controlled non-inferiority multicenter study. Patients who meet the in- and exclusioncriteria will be randomized after informed consent for one of the two treatment methods:
- Arm A (control arm): lumpectomy with sentinel lymph node procedure, followed by radiotherapy of the breast with or without completion axillary treatment according to the Dutch breast cancer guideline.
- Arm B (experimental arm): lumpectomy without further axillary staging, followed by radiotherapy of the breast.
Stratification: age (≤50, 50≤75, >75 years old), oestrogen receptor status (positive vs. negative), HER2neu status (amplified vs. not amplified), clinical tumour stage prior to any treatment (<3 cm vs. ≥3 cm), grading (grade I-II vs. III - according to Bloom-Richardson grading system), is or will be treated with primary systemic therapy (yes/no) and participating centre.
Adjuvant systemic treatment is administered if indicated according to the Dutch breast cancer guideline.

Primary systemic therapy is allowed if the patient has a clinical T1-2N0 status (initial stage) that is amenable to lumpectomy pre-systemic therapy.
Yearly follow-up with physical examination of the axilla for 10 years. Axillary ultrasound (+/- tissue sampling) on indication. Staging for distant metastases and/or a delayed axillary lymph node dissection if indicated by the multidisciplinary team. Validated questionnaires are used to assess axillary morbidity rate and quality of life (Lymph-ICF, QLQ-C30, QLQ-BR23) and to measure if anxiety and personality traits influence the outcome of quality of life (STAI-trait, NEO-FFI) in 700 of 878 patients. These questionnaires are provided before randomization and sequentially post randomization at 6 months, 1, 2, 5 and 10 years.

Study population:
Women aged 18 years or older with pathologically confirmed invasive breast carcinoma about to undergo breast conserving surgery followed by radiotherapy of the breast.
cT1-2: primary tumour is smaller than 5 cm.
cN0: no signs of axillary lymph node metastases at physical examination and axillary ultrasound (+/- tissue sampling).
Primary systemic treatment is allowed.
Patient is able to and signed the informed consent form.

Intervention (if applicable):
Patients in the control arm will be treated with the sentinel lymph node procedure according to the Dutch breast cancer guideline.
Patients in the experimental arm will not be treated with the sentinel lymph node procedure.

Primary study parameters/outcome of the study:
Primary endpoint:
- Regional recurrence rate

Secundary study parameters/outcome of the study (if applicable):
Secondary endpoints:
- Distant-disease free survival
- Overall survival
- Delayed axillary treatment
- Local recurrence rate
- Other-regional recurrence rate
- Contralateral breast cancer rate
- Percentage difference in the administration of (neo)adjuvant systemic therapy
- Quality of life and axillary morbidity rate

Nature and extent of the burden and risks associated with participation, benefit and group relatedness (if applicable):
There is no extra burden for participating patients as regard to additional diagnostic tests/interventions and follow-up. Any additional burden for the patient will only consist of completing quality of life questionnaires in 700 of 1644 study patients. These questionnaires are provided before randomization and sequentially post randomization at 6 months, 1, 2, 5 and 10 years.
We hypothesize that omitting the sentinel lymph node procedure is not inferior to the standard treatment. Nevertheless, possible disadvantages are a worse regional recurrence rate, and delayed axillary treatment if indicated. Patients in the experimental arm have the possible advantages of less morbidity, fewer operations and hospitalizations and an improved quality of life, compared to patients treated with the sentinel lymph node procedure.
No patient will encounter any delay in their treatment as a result of inclusion.

ONDERTEKENING

De verrichter en indiener verklaren hierbij:
a. het formulier (en samenvatting) volledig en naar waarheid te hebben ingevuld;
b. de antwoorden op de vragen uit het ABR-formulier niet in strijd zijn met het bijbehorende onderzoeksdossier en onderzoekscontract

Naar waarheid getekend, door de verrichter (=opdrachtgever)		door de indiener	
datum	....	datum	....
Handtekening		Handtekening	
naam	M.J.H.M. Jacobs	naam	J.A. van der Hage
functie	Hoofd afdeling Chirurgie, MUMC+	functie	Chirurg, NKI-AVL

Sluiten Print

Sluiten Print

Formulier voor medisch-ethische beoordeling en registratie

ABR-formulier, versie december 2015

Onderzoeksdossiernummer

ABR Nummer	**49315**	Status	**Beoordeeld - Positief**
Versie	**05**	Status per	**15-07-2016**
Jaar	**16**		
Dossiernummer	**NL49315.031.14**		
Reden voor	**Toevoegen centra**		

A. Sectie - Openbaar maken gegevens medisch wetenschappelijk onderzoek

A1. Het CCMO-register is een voor ieder toegankelijk openbaar trial register. De antwoorden op de vragen gemarkeerd met een wereldbol en de samenvatting bij dit formulier worden openbaar gemaakt in het CCMO register.

B. Sectie - Administratief

B1. Betreft het onderzoek met geneesmiddelen (inclusief gentherapie, somatische celtherapie, vaccinonderzoek, GGO's, zie verdere toelichting) als bedoeld in de Wet medisch-wetenschappelijk onderzoek met mensen (WMO)?

○ ja
◉ nee

B2. Houdt het onderzoek verband met een eerder door een erkende METC of door de CCMO beoordeelde studie of is het onderzoek reeds eerder bij een erkende METC ter beoordeling voorgelegd?

○ ja, het onderzoek houdt verband met – of is het vervolg op – een eerder beoordeelde studie
○ ja, het onderzoek is eerder ter beoordeling aan een erkende METC of de CCMO voorgelegd (stuur kopie besluit mee)
◉ nee

B4. Is het protocol (nog) in een ander openbaar trial register geregistreerd?

○ ja
◉ nee

B5. Naam indiener/contactpersoon voor de oordelende toetsingscommissie

**B5a.**	Achternaam indiener/contactpersoon	Hage
	Titel en voorletters	dr. J.A.
	Tussenvoegsels	van der
**B5b.**	Type organisatie/bedrijf	Overige Ziekenhuizen
	Organisatie/bedrijf	Antoni van Leeuwenhoek Ziekenhuis
	Naam organisatie/bedrijf	IKNL trialbureau
	Afdeling	Chirurgie
	Adres	Plesmanlaan 121
	Postcode en plaats	1066 CX Amsterdam
	Land	NL
**B5c.**	Intern adres	afdeling Heelkunde
	Telefoon	020 512 9111
	Fax	020 512 2554
	E-mail	j.vd.hage@nki.nl

B6. Is de indiener werkzaam bij de opdrachtgever/sponsor (verrichter) van het onderzoek?

○ Ja ◉ Nee

B7. Opdrachtgever/sponsor van het onderzoek (verrichter volgens de WMO)

Type Organisatie/Bedrijf	Universitair Medisch Centrum
Organisatie/Bedrijf	Medisch Universitair Ziekenhuis Maastricht

Adres	P. Debyelaan 25
Postcode en plaats	6229 HX Maastricht
Land	NL
Telefoon	0433877477
Fax	0433875473
E-mail	m.smidt@mumc.nl

C. Sectie - Onderzoek

C1. Volledige titel van het onderzoek
C1a. In het Engels
Clinically node negative breast cancer patients undergoing breast conserving therapy: Sentinel lymph node procedure versus follow-up. A Dutch randomized controlled multicentre trial.

C1b. In het Nederlands
Patiënten met borstkanker en een klinisch negatieve okselklierstatus die een borstsparende behandeling ondergaan: Schildwachtklierprocedure versus follow-up. Een Nederlandse gerandomiseerde multicenter studie.

C2. Verkorte titel van het onderzoek/acroniem
C2a. In het Engels
BOOG 2013-08

C2b. In het Nederlands (Let op: deze korte titel wordt vermeld binnen ToetsingOnline)
BOOG 2013-08

C3. Trefwoorden (maximaal 4, plaats elk trefwoord op een aparte regel)
C3a. In het Engels
Breast neoplasms
Breast conserving therapy
Sentinel lymph node biopsy
Axilla

C3b. In het Nederlands
Borstkanker
Borstsparende behandeling
Schildwachtklierprocedure
Oksel

C4. Beschrijf het belang van het onderzoek en de beoogde toepassing van de resultaten (verwijs eventueel naar de relevante pagina's in het protocol).
Het doel van dit onderzoek is om:
1) Overbehandeling van de oksel te verminderen;
2) Het aantal korte en lange termijn complicaties te verminderen;
3) De kwaliteit van leven te verbeteren;
4) Met een even goede ziektecontrole en algehele overleving

Dit willen wij bereiken door te onderzoeken of de schildwachtklierprocedure veilig achterwege gelaten kan worden bij patiënten met borstkanker en een klinisch negatieve okselklierstatus behandeld met borstsparende therapie.

C6. Betreft het onderzoek een multicenter-onderzoek?
○ nee
◉ ja - alleen in Nederland
○ ja - internationaal binnen de Europese Unie
○ ja - internationaal ook buiten de Europese Unie

C7. Is er bij multicenter-onderzoek sprake van een coördinerend onderzoeker?
◉ ja namelijk — Naam onderzoeker: M.L. Smidt
○ nee

C8. Wie is/zijn medisch verantwoordelijk voor de proefpersonen die deelnemen aan het onderzoek
Behandelend arts en lokale hoofdonderzoeker

C9. In welk centrum/welke centra (incl. huisartsenpraktijken) in Nederland wordt het onderzoek uitgevoerd?

Centrum	Proefpersonen	Hoofdonderzoeker	Onafhankelijk arts
Albert Schweitzer Ziekenhuis	49	M.B.E. Menke-Pluijmers	S.O. Breukink (Maastricht UMC+)
Amphia Ziekenhuis	69	E.J.T. Luiten	S.O. Breukink (Maastricht UMC+)

Antonius Ziekenhuis, Sneek	56	C.G.J.M. van Riel	S.O. Breukink (Maastricht UMC+)
Atrium Medisch Centrum	39	E.R.M. van Haaren	S.O. Breukink (Maastricht UMC+)
Bronovo Ziekenhuis	23	O.R. Guicherit	S.O. Breukink (Maastricht UMC+)
Canisius Wilhelmina Ziekenhuis	46	L.J.A. Strobbe	S.O. Breukink (Maastricht UMC+)
Catharina-ziekenhuis	46	G.A.P. Nieuwenhuijzen	S.O. Breukink (Maastricht UMC+)
Deventer Ziekenhuis	39	H. de Vries	S.O. Breukink (Maastricht UMC+)
Zorggroep De Sionsberg	15	K. Blaauwendraat	S.O. Breukink (Maastricht UMC+)
Diakonessenhuis Utrecht	39	T. van Dalen	S.O. Breukink (Maastricht UMC+)
Gelre Ziekenhuizen	37	W.B. Lastdrager	S.O. Breukink (Maastricht UMC+)
Groene Hart Ziekenhuis	31	E. Linthorst	S.O. Breukink (Maastricht UMC+)
HagaZiekenhuis	41	I. Jannink	S.O. Breukink (Maastricht UMC+)
Ikazia Ziekenhuis	27	B.R. Toorenvliet	S.O. Breukink (Maastricht UMC+)
Isala Klinieken	54	A.B. Francken	S.O. Breukink (Maastricht UMC+)
Kennemer Gasthuis	43	R.J.C.L.M. Vuylsteke	S.O. Breukink (Maastricht UMC+)
LangeLand Ziekenhuis	14	B. Jas	S.O. Breukink (Maastricht UMC+)
Laurentius Ziekenhuis	23	A.V.R.J. Bell	S.O. Breukink (Maastricht UMC+)
Academisch Ziekenhuis Maastricht	39	M.L. Smidt	S.O. Breukink (Maastricht UMC+)
Martini Ziekenhuis	43	W. Kelder	S.O. Breukink (Maastricht UMC+)
Maxima Medisch Centrum	31	K. Schenk	S.O. Breukink (Maastricht UMC+)
Meander Medisch Centrum	43	A.J. van Overbeeke	S.O. Breukink (Maastricht UMC+)
Antoni van Leeuwenhoek Ziekenhuis	93	J.A. van der Hage	S.O. Breukink (Maastricht UMC+)
Onze Lieve Vrouwe Gasthuis	28	P.J. Borgstein	S.O. Breukink (Maastricht UMC+)
Orbis Medisch Centrum	23	Y. Vissers	S.O. Breukink (Maastricht UMC+)
Universitair Medisch Centrum Sint Radboud	31	J.H.W. de Wilt	S.O. Breukink (Maastricht UMC+)
Reinier de Graaf Groep	46	M. van 't Riet	S.O. Breukink (Maastricht UMC+)
Sint Antonius Ziekenhuis	58	R. Koelemij	S.O. Breukink (Maastricht UMC+)
Sint Elisabeth Ziekenhuis	37	P. Jansen	S.O. Breukink (Maastricht UMC+)
Streekziekenhuis Koningin Beatrix	19	A. Jonk	S.O. Breukink (Maastricht UMC+)
Tergooiziekenhuizen locatie Blaricum	42	E.J.C. Vriens	S.O. Breukink (Maastricht UMC+)
Universitair Medisch Centrum Groningen	17	L. Jansen	S.O. Breukink (Maastricht UMC+)
Universitair Medisch Centrum Utrecht	23	A.J. Witkamp	S.O. Breukink (Maastricht UMC+)
Viecuri Medisch Centrum voor Noord-Limburg	39	C.L.H. van Berlo	S.O. Breukink (Maastricht UMC+)
Westfries Gasthuis	27	J.W.D. de Waard	S.O. Breukink (Maastricht UMC+)
Waterland Ziekenhuis	22	L. de Widt	S.O. Breukink (Maastricht UMC+)
Zaans Medisch Centrum	15	S. Muller	S.O. Breukink (Maastricht UMC+)
Ziekenhuisvoorzieningen Gelderse Vallei	32	W.K. de Roos	S.O. Breukink (Maastricht UMC+)
Ziekenhuisgroep Twente	31	D. de Leeuw	S.O. Breukink (Maastricht UMC+)

Rivierenland Ziekenhuis	23	M.A.J. de Roos	S.O. Breukink (Maastricht UMC+)
Medisch Centrum Alkmaar	54	W.H. Schreurs	S.O. Breukink (Maastricht UMC+)
Medisch Centrum Haaglanden	31	A. Marinelli	S.O. Breukink (Maastricht UMC+)
Medisch Spectrum Twente	46	P. Steenvoorde	S.O. Breukink (Maastricht UMC+)
Rijnstate Ziekenhuis	62	R. van Eekeren	S.O. Breukink (Maastricht UMC+)
Flevoziekenhuis	50	S. van der Meij	S.O. Breukink (Maastricht UMC+)
Zuwehofpoort	15	R.C. van Doorn	S.O. Breukink (Maastricht UMC+)
Jeroen Bosch Ziekenhuis	20	M. Bessem	B. Biesma (Jeroen Bosch ziekenhuis)
Alrijne ziekenhuis	50	P. Neijenhuis	S.O. Breukink (Maastricht UMC+)

C10. Betreft het onderzoek met:

- [x] mensen
- [ ] geslachtscellen
- [ ] (rest-)embryo's
- [ ] foetussen in utero

C11. Beoogd totaal aantal proefpersonen/(rest)embryo's/foetussen in utero:
C11a. In Nederland
1644

C13. Onderzoeksgebied

- [ ] etiologie
- [ ] organisatorisch/zorgonderzoek
- [ ] diagnostiek
- [ ] preventie
- [x] therapie
- [ ] veiligheid
- [ ] werkzaamheid
- [ ] farmacokinetiek
- [ ] farmacodynamiek
- [ ] bio-equivalentie
- [ ] dosis-respons
- [ ] farmacogenomics
- [ ] farmaco-economie
- [ ] anders

C14. Type onderzoek

- ○ observationeel onderzoek zonder invasieve metingen
- ○ observationeel onderzoek met invasieve metingen
- ◉ interventie-onderzoek

C15. In welke fase kan het onderzoek worden ingedeeld?

- ○ fase I (a)
- ○ fase II (b)
- ◉ fase III (c)
- ○ fase IV (d)
- ○ overige onderzoeken waarbij geneesmiddelen worden toegepast (e)
- ○ niet van toepassing

C17. Is er sprake van een andere interventie dan met geneesmiddelen zoals bedoeld in de Wet medisch-wetenschappelijk onderzoek met mensen (WMO) (zie toelichting)?

- ◉ Ja
- ○ Nee

C17a. ☐ Medisch hulpmiddel, namelijk
C17b. ☐ Operatie, namelijk
C17c. ☐ Psychosociale interventie, namelijk
C17d. ☐ Voeding(stoffen), namelijk
C17e. ☐ Bewegingstherapie, namelijk
C17f. ☐ interventie met radioactieve straling, namelijk
C17g. ☐ Blootstellingsonderzoek (bijv pesticidenonderzoek), namelijk
C17h.
C17i. ☑ andere interventie, namelijk

In het Engels:	No sentinel lymph node procedure
In het Nederlands:	Géén schildwachtklierprocedure

C18. Worden de onderzoeksproducten voor deze studie door de verrichter gratis verstrekt?

○ ja
○ nee
○ gedeeltelijk namelijk
◉ niet van toepassing

C19. Is/zijn er (een) controlegroep (en)?

◉ ja
○ nee

☐ ja, geneesmiddel
☐ ja, placebo
☐ ja, geen interventie
☑ ja, anders

namelijkDe standaard behandeling: schildwachtklierprocedure

C20. Betreft het een gerandomiseerd onderzoek?

◉ ja
○ nee

namelijk

☑ open
☐ enkelblind
☐ dubbelblind
☐ parallel
☐ cross-over
☐ anders namelijk

C21. Op welke klasse(n) van aandoeningen heeft het onderzoek betrekking (maximaal 3)

☐ hartaandoeningen

☐ congenitale, familiaire en genetische aandoeningen
☐ bloed- en lymfestelsel aandoeningen
☐ zenuwstelsel aandoeningen
☐ oogaandoeningen
☐ evenwichtsorgaan- en ooraandoeningen
☐ ademhalingsstelsel-, thorax- en mediastinumaandoeningen
☐ maagdarmstelselaandoeningen
☐ nier- en urinewegaandoeningen
☐ huid- en onderhuidaandoeningen
☐ skeletspierstelsel- en bindweefselaandoeningen
☐ endocriene aandoeningen
☐ voedingsstoornissen en metabole ziekten

- ☐ infecties en parasitaire aandoeningen
- ☐ letsels, intoxicaties en verrichtingscomplicaties
- ☑ neoplasmata, benigne, maligne en niet-gespecificeerd (incl cysten en poliepen)
  borstneoplasmata maligne en niet-gespecificeerd (incl. tepel)
- ☑ chirurgische en medische verrichtingen
  borst therapeutische verrichtingen
- ☐ bloedvataandoeningen
- ☐ algemene aandoeningen en aandoeningen op de plek van toediening
- ☐ zwangerschap, perinatale periode en puerperium
- ☐ sociale omstandigheden
- ☐ immuunsysteemaandoeningen
- ☐ lever- en galaandoeningen
- ☐ voortplantingsstelsel- en borstaandoeningen
- ☐ psychische stoornissen
- ☐ overig, namelijk

C22.Geef twee synoniemen voor de aandoening die bestudeerd wordt, waarvan tenminste één lekenterm.

In het Engels:	breast cancer, sentinel lymph nodes, sentinel lymph node metastases
In het Nederlands:	borstkanker, schildwachtklieren, schildwachtkliermetastasen

C23. Beoogde start- en einddatum van het onderzoek

C23a. Start Datum (dd-mm-jjjj)	01-01-2015
C23b. Eind Datum (dd-mm-jjjj)	01-01-2027

C24. Zijn patiënten betrokken bij het ontwikkelen van het onderzoek? (Voor patiënten lees ook: patiëntenorganisaties, naasten of nabestaanden van patiënten en gezonde vrijwilligers)?

C30. Wordt de ervaring van proefpersonen na deelname aan het onderzoek geëvalueerd?

D. Sectie - Proefpersonen

D1. Is er een proefpersonenverzekering conform de WMO-eisen afgesloten of wordt aan de oordelende toetsingscommissie ontheffing gevraagd?

- ◉ proefpersonenverzekering is afgesloten bij verzekeringsmaatschappij
- ○ ontheffing van de verzekering wordt gevraagd
- ○ niet van toepassing - onderzoek valt onder de Embryowet en niet onder de WMO

Verzekeringsmaatschappij	Marketform (onderdeel van Lloyd's)

D2. Gezonde proefpersonen en/of patiënten

- ☐ Gezonde proefpersonen

- ☑ Patiënten

Aantal	1644

D4. Voornaamste inclusiecriteria

D4a. In het Engels

- Female
- Aged 18 years or older
- Pathologically confirmed invasive unilateral breast carcinoma
- A clinical T1-2 tumour (≤ 5cm)
- Will be treated with lumpectomy followed by whole breast radiotherapy
- Clinically node negative status: no signs of axillary lymph node metastases at physical examination and preoperative axillary ultrasound (or negative cyto-/histopathology)
- Written informed consent

D4b. In het Nederlands

- Vrouw
- Leeftijd 18 jaar of ouder
- Pathologisch bewezen invasief unilaterale borstkanker
- Klinisch T1-2 tumor (≤ 5cm)
- Zal worden behandeld met lumpectomie gevolgd door gehele borstbestraling
- Klinisch negatieve okselklierstatus: negatief lichamelijk onderzoek en echografie van de oksel (of negatieve cyto-/histologie)
- Schriftelijke informed consent

D5. Voornaamste exclusiecriteria

D5a. In het Engels

- Clinically node positive pre-operative
- Bilateral breast cancer
- Evidence of metastatic disease
- History of invasive breast cancer
- Previous treatment of the ipsilateral axilla with surgery or radiotherapy, except surgery for hidradenitis suppurativa or for other superficially located skin lesions, such as naevi
- Pregnant or nursing
- Other prior malignancies within the past 5 years, except successfully treated malignancies that occurred more than five years before randomization, and except successfully treated basal cell and squamous cell skin cancer, carcinoma in situ of the cervix or carcinoma in situ of the ipsilateral or contralateral breast
- Unable or unwilling to give informed consent

D5b. In het Nederlands

- Klinisch positieve okselklierstatus (preoperatief)
- Bilaterale borstkanker
- Primair (afstands-)gemetastaseerde borstkanker
- Invasieve borstkanker in de voorgeschiedenis
- Chirurgie of radiotherapie van ipsilaterale oksel in de voorgeschiedenis, behalve chirurgie voor hidradenitis suppurativa of andere oppervlakkig gelegen huidlaesies, zoals naevi
- Zwanger of lacterend
- Andere maligniteit in de afgelopen 5 jaar, behalve succesvol behandelde maligniteit die meer dan vijf jaar voor randomisatie optrad, en behalve succesvol behandelde huidkanker van type basaalcel of plaveiselcel, carcinoma in situ van de cervix, carcinoma in situ van de ipsilaterale of contralaterale mamma
- Niet in staat of niet bereid tot informed consent

D6. Bij welke categorie proefpersonen wordt het onderzoek uitgevoerd (meerdere antwoorden mogelijk)

- [x] 18 jaar of ouder en wilsbekwaam
- [ ] 18 jaar of ouder en wilsonbekwaam
- [ ] 12 t/m 17 jaar en in staat tot het geven van geïnformeerde toestemming
- [ ] 12 t/m 17 jaar en niet in staat tot het geven van geïnformeerde toestemming (wilsonbekwaam)
- [ ] jonger dan 12 jaar

D10. Verkeren (sommige) proefpersonen in een afhankelijkheidssituatie ten opzichte van de onderzoeker of degene die de deelnemers werft? (lees de toelichting voor voorbeelden wanneer er sprake kan zijn van een afhankelijkheidssituatie)

- (●) ja
- ( ) nee

D10a. Zo ja, waardoor?

Soms is de lokale onderzoeker ook de behandelend arts die de patiënt vraagt om deel te nemen aan de studie.

D10b. Waarom wordt het onderzoek juist met deze proefpersonen uitgevoerd en hoe worden de belangen van de proefpersonen gewaarborgd?

Het onderzoek moet met proefpersonen worden uitgevoerd die voldoen aan de beschreven in/exclusie criteria zodat de resultaten van de studie uiteindelijk ook naar de kliniek vertaald kunnen worden binnen deze patiëntenpopulatie. De belangen van de proefpersonen worden gewaarborgd door de geldende medisch ethische normen en richtlijn zoals vermeld in het protocol (o.a. onafhankelijk arts, uitgebreide patiëntinformatiefolder).

D11. Waaruit bestaat de vergoeding voor de proefpersonen?

- [x] geen vergoeding
- [ ] reiskosten
- [ ] financiële vergoeding (in Euro's)
- [ ] andere vergoeding

D12. Is deze vergoeding afhankelijk van bepaalde voorwaarden, bijvoorbeeld het voltooien van (een deel van) het onderzoek?

- ( ) ja (motiveer)
- ( ) nee
- (●) niet van toepassing

E. Sectie - Voor- en nadelen

E1. Wordt er bij dit onderzoek een rechtstreeks therapeutisch effect beoogd bij de proefpersonen / patiënten?

- (●) ja (therapeutisch onderzoek)
- ( ) nee (niet-therapeutisch onderzoek)

E1a. Zo ja, geef kort aan waaruit dit therapeutisch effect bestaat.

Het therapeutische effect bestaat uit:
- Het verlagen van het risico op okselbehandeling-gerelateerde korte en lange termijn complicaties;
- Het verbeteren van de kwaliteit van leven;
- Met een even goede ziektecontrole en algehele overleving.

E2. Waaruit bestaat de belasting van het onderzoek (en een eventueel daaraan voorafgaande keuring) voor de proefpersonen?

Tijdbeslag	per bezoek	20 min per keer voor invullen kwaliteit van leven vragenlijst
	totaal	20 min x 6 = 120 min
	totale duur van de studie voor de individuele proefpersoon	Tav de behandeling minder belasting dan normaal voor de studie-arm (geen schildwachtklierprocedure). Voor beide armen is extra het invullen van de kwaliteit van leven vragenlijst prerandomisatie en postrandomisatie op 5 momenten.

E3. Worden de proefpersonen in verband met het onderzoek in het ziekenhuis opgenomen of wordt een opname verlengd?

○ ja - het verblijf in het ziekenhuis/instituut wordt in verband met het onderzoek verlengd
○ ja - ze worden voor het onderzoek in het ziekenhuis/instituut opgenomen
◉ nee

E4. Beschrijf in hoeverre proefpersonen worden onderworpen aan handelingen dan wel een gedragswijze krijgen opgelegd, zoals vragenlijst, interviews, lichamelijk/psychologisch onderzoek, ontzegging, dieet (voor invasieve ingrepen: zie vraag E6)

In totaal zullen 700 proefpersonen (350 in de experimentele en 350 in de controlegroep) deelnemen aan het kwaliteit van leven onderzoek, bestaande uit een vragenlijst voor de randomisatie en op 5 tijdstippen na de randomisatie (na een half jaar, één, twee, vijf en 10 jaar).

E5. Worden de proefpersonen getest op bepaalde aandoeningen/condities?

○ ja (motiveer)
◉ nee

E6. Welke extra (invasieve) ingrepen (anders dan bij de standaard behandeling) moeten de proefpersonen in het kader van het onderzoek ondergaan:

☑ Niet van toepassing

E9. Geef aan welke risico's er voor proefpersonen zijn verbonden aan deelname aan het onderzoek.

Hoewel de hypothese luidt dat het achterwege laten van de schildwachtklierprocedure geen nadelige invloed heeft op de lokale controle van de ziekte en de overlevingskans, bestaat er toch een kans dat proefpersonen in de experimentele arm een hoger risico hebben op een recidief in de oksel (regionaal recidief) en daardoor tijdens follow-up alsnog een vorm van okselklierbehandeling moeten ondergaan. Uit onderzoek blijkt dat het uitgesteld uitvoeren van okselklierbehandeling (bijvoorbeeld met okselklierdissectie) géén nadelige gevolgen heeft voor de overleving.

E9a. Geef op grond van uw eigen afweging aan waarom het uitvoeren van het onderzoek, in het licht van de belasting en/of risico's die voor proefpersonen aan deelname verbonden zijn, gerechtvaardigd is?

Het mogelijk hogere risico op een recidief in de oksel (regionaal recidief) weegt op tegen de lagere kans op ernstige okselklierbehandeling-gerelateerde morbiditeit (lymfoedeem, bewegingsbeperking in de schouder, arm- en schouderpijn, verminderd gevoel of zenuwpijn) en de verbetering van kwaliteit van leven.

E10. Indien het onderzoek bij minderjarige en/of wilsonbekwame proefpersonen wordt uitgevoerd en geen direct therapeutisch effect wordt beoogd: waarom kunnen belasting en risico's als minimaal worden beschouwd? (Verwijs eventueel naar de relevante pagina's in het protocol.

☑ niet van toepassing

E11. Kan de eventuele therapie na beëindiging van het onderzoek worden voortgezet?

○ ja (motiveer)
○ nee (motiveer)
◉ niet van toepassing

E12. Heeft deelname aan het onderzoek voor de proefpersoon tot gevolg dat van de standaardbehandeling of -diagnostiek kan worden afgeweken of deze kan worden uitgesteld?

◉ ja
○ nee
○ niet van toepassing

E12a. Zo ja, waaruit bestaat de afwijking of het uitstel en waarom is afwijking/uitstel

Proefpersonen in de controlegroep ondergaan de standaard behandeling, namelijk schildwachtklierprocedure volgens de Nederlandse borstkankerrichtlijn. Proefpersonen in de experimentele arm ondergaan primair géén schildwachtklierprocedure. Beide groepen ondergaan dezelfde follow-up middels lichamelijk onderzoek en aanvullend onderzoek op indicatie. Indien een proefpersoon zich in de follow-up periode presenteert met lymfekliermetastasen, kan alsnog besloten worden om een vorm van okselklierbehandeling uit te voeren. Deze beslissing wordt gemaakt tijdens het multidisciplinair overleg in het desbetreffende centrum waar de patiënt onder behandeling staat.

F. Sectie - Informatie en privacy

F1. Hoe worden de proefpersonen geworven en door wie (onderzoeker, behandelend arts, andere persoon) wordt de proefpersoon/wettelijke vertegenwoordiger geïnformeerd en om toestemming gevraagd?

Op het moment in de behandeling van borstkanker dat de patiënt voldoet aan de in/exclusiecriteria zal de behandeld arts informatie geven over de mogelijkheid tot deelname aan deze studie. Een schriftelijke patiëntinformatiebrief zal worden meegegeven aan de patiënt. De patiënt krijgt ruim de tijd (ten minste één week) om deze informatie thuis na te lezen en een beslissing te nemen. De patiënt kan haar interesse in deelname kenbaar maken aan haar behandelend arts bij een volgend polibezoek. De behandelend arts zal haar dan om toestemming vragen voor deelname aan de studie.

F2. Hoeveel bedenktijd krijgen de proefpersonen/wettelijke vertegenwoordigers om te beslissen over deelname?

Zo lang als nodig, met ten minste één week

F3. Wordt de huisarts, behandelend specialist en/of apotheker van de proefpersoon geïnformeerd over diens deelname aan het onderzoek?

- (●) ja (de proefpersoon dient hiervoor toestemming te geven)
- ( ) nee

F4. Worden persoonsgegevens gecodeerd?

- (●) ja
- ( ) nee

F4a. Zo ja, hoe is deze codering opgebouwd?

Alle proefpersonen krijgen een uniek studienummer. Het unieke studienummer betreft een opvolgnummer zonder verdere specifieke codering voor bijvoorbeeld centrum.

F4b. Wie heeft toegang tot de sleutel van deze code?

De behandelend arts en de onderzoeker

F4c. Wie hebben toegang tot de brondocumenten en eventuele andere tot de persoon herleidbare gegevens?

De lokaal datamanagers en de onderzoeker

F5. Hoe wordt het lichaamsmateriaal gedurende het onderzoek bewaard?

- ( ) in tot de proefpersoon herleidbare vorm (gecodeerd)
- ( ) in niet tot de proefpersoon herleidbare vorm (volledig geanonimiseerd)
- (●) niet van toepassing

F6. Wordt afgenomen lichaamsmateriaal na afloop van het onderzoek vernietigd?

- ( ) ja
- ( ) nee (motiveer)
- (●) niet van toepassing

F7. Kunnen proefpersonen na afloop van het onderzoek opnieuw benaderd worden (bijvoorbeeld voor nader onderzoek of follow-up)?

- ( ) ja
- (●) nee

G. Sectie - Financieel

G1. Door welke geldstroom wordt het onderzoek gefinancierd?

- [ ] eerste geldstroom (Geld van Ministerie van OC&W aan universiteiten)
- [ ] tweede geldstroom (NWO of KNAW), namelijk
- [x] derde geldstroom (anders dan 1e of 2e geldstroom, zoals collectebusfondsen, Europese Unie, vakministeries of bedrijven), namelijk

KWF Kankerbestrijding

G2. Wordt het onderzoek (mede) gefinancierd door de industrie/bedrijven?

- ○ ja - door de industrie/bedrijf zoals is opgegeven bij vraag B6/B7 (opdrachtgever van het onderzoek)
- ○ ja - (ook) door andere industrie/bedrijven dan de opdrachtgever
- ◉ nee

G3. Wat is de hoogte van de vergoeding die de arts/onderzoeker cq onderzoeksafdeling/maatschap ontvangt voor de uitvoering van het onderzoek?

- ○ Per patiënt of proefpersoon
- ◉ Per deelnemend centrum

bedrag afgerond in hele euro's: € 100,00

G3a. Hoe is de vergoeding opgebouwd?

De vergoeding betreft een financiële compensatie van € 100 excl. BTW als opstartvergoeding voor ieder deelnemend centrum.

G4. Heeft/hebben de onderzoeker(s) gedurende de afgelopen vijf jaar op een of andere wijze een persoonlijke financiële relatie (gehad) met de verrichter/sponsor van het huidige onderzoek?

- ○ ja (licht toe)
- ◉ nee

I. Sectie - Indiening en beoordeling

I1. Sla het formulier eerst op en selecteer vervolgens een METC of de CCMO

Commissie
METC NedMec

J. Sectie - Aanvullende opmerkingen

Aanvullende opmerkingen

K. Sectie - Samenvatting

Achtergrond van het onderzoek:

De schildwachtklierprocedure is de standaard methode voor lymfeklierstadiëring in borstkankerpatiënten met een klinisch negatieve okselklierstatus. In de algemene bevolking bevat de schildwachtklier in ongeveer 25% een uitzaaiing. Dit percentage is lager in patiënten met een kleine borsttumor en in borstsparend behandelde patiënten. De ACOSOG-Z0011 studie en de IBCSG 23-01 studie hebben recent aangetoond dat een completerende okselklierdissectie veilig achterwege gelaten kan worden bij borstsparend en met systemische therapie behandelde patiënten met een beperkt aantal schildwachtkliermetastasen. Dit roept de vraag op of de schildwachtklierprocedure nog wel noodzakelijk is voor borstsparend behandelde patiënten met een klinisch negatieve okselklierstatus. De klinisch negatieve okselklierstatus van de patiënten in de beschreven studies werd gesteld op basis van lichamelijk onderzoek. Het toevoegen van een preoperatieve echografie van de oksel zal zorgen voor een betere voorselectie van patiënten zonder lymfekliermetastasen, ten opzichte van lichamelijk onderzoek alleen. Daarbij sluit een negatieve echografie uitgevoerd in Nederland bijna alle patiënten uit met uitgebreide lymfekliermetastasen (≥4 lymfekliermetastasen). Bovendien omvat gehele borstbestraling na de borstsparende operatie in het algemeen het lagere deel van de oksel en behandelt daarmee eventueel achtergebleven uitzaaiingen. Hoewel de schildwachtklierprocedure minder invasief is vergeleken met een okselklierdissectie, komen complicaties nog steeds voor. Zo lijdt 6% van de patiënten die alleen een schildwachtklierprocedure ondergingen aan ernstige lymfoedeem na slechts 3 jaar. Deze studie gaat onderzoeken of de schildwachtklierprocedure veilig achterwege gelaten kan worden bij borstsparend behandelde patiënten, met een negatieve echografie van de oksel.

Doel van het onderzoek:

Het doel van deze studie is het verminderen van overbehandeling van de oksel bij patiënten met borstkanker en een klinisch negatieve okselklierstatus die behandeld worden met borstsparende therapie, door het achterwege laten van de schildwachtklierprocedure, om zodoende de kans op korte en lange termijn complicaties te verminderen en te zorgen voor een verbetering van de kwaliteit van leven met een even goede ziektecontrole en algehele overleving.

Onderzoeksopzet:

Het betreft een gerandomiseerde non-inferiority multicenter onderzoek. Patienten die voldoen aan de in- en exclusiecriteria worden na toestemming middels loting behandeld volgens één van de volgende behandelmethoden:

- Behandelmethode A (controle groep): schildwachtklierprocedure volgens de Nederlandse borstkankerrichtlijn.
- Behandelmethode B (studie groep): geen verdere okselklierstadiëring.

Stratificatiefactoren: leeftijd (≤50, 50≤75, >75 jaar), oestrogeen receptor status (positief vs. negatief), HER2neu status (geamplificeerd vs. niet geamplificeerd), klinisch tumor stadium (<3 cm vs. ≥3 cm), tumorgraad volgens gemodificeerde Bloom-Richardson (graad I-II vs. graad III), is of zal worden behandeld met primaire systemische therapie (ja/nee), participerend centrum.
Overige behandelingen (o.a. hormonale therapie, chemotherapie) worden op indicatie gegeven volgens de Nederlandse borstkankerrichtlijn.
Tijdens de follow-up periode van 10 jaar wordt de oksel nauwkeurig onderzocht middels lichamelijk onderzoek en echografie op indicatie. Indien sprake is van een cyto-/histologisch bewezen lymfekliermetastase tijdens follow-up, dan kan in het multidisciplinair overleg van de desbetreffende patiënt worden besloten tot het uitvoeren van een vorm van okselklierbehandeling.
Gevalideerde vragenlijsten worden verstrekt voor het onderzoeken van de invloed op het aantal complicaties en kwaliteit van leven (Lymph-ICF, QLQ-C30, QLQ-BR23) en om te meten of angst en persoonlijkheidstrekken invloed hebben op de uitkomst van

kwaliteit van leven (STAI-trait, NEO-FFI). Deze vragenlijsten worden verstrekt aan 700 van de 1644 patiënten (350 per arm), éénmaal voor de randomisatie en vijfmaal na de randomisatie (6 maanden, 1 jaar, 2 jaar, 5 jaar en 10 jaar).

Onderzoekspopulatie:

Vrouwen van 18 jaar of ouder met pathologisch bewezen invasief mammacarcinoom die borstsparende therapie ondergaan.
cT1-2: primaire tumor is bij klinisch onderzoek kleiner dan 5 cm.
cN0: bij lichamelijk onderzoek en echografie van de oksel worden geen aanwijzingen voor lymfekliermetastasen gevonden of de cytologie/histologie na punctie/biopt van een lymfeklier is negatief.
Neoadjuvante systemische therapie is toegestaan indien de patiënt voor de start van de neoadjuvante systemische therapie een klinische T1-2N0 status heeft en waarvoor borstsparende therapie op dat moment al geschikt is.
De patiënt heeft informed consent persoonlijk ondertekend.

Interventie (indien van toepassing):

De controlegroep ondergaat wél de schildwachtklierprocedure volgens de Nederlands borstkankerrichtlijn. De experimentele arm ondergaan géén schildwachtklierprocedure.

Primaire onderzoeksvariabelen/uitkomstmaten:

Primaire uitkomstmaat:
- Regionaal recidief percentage

Secundaire onderzoeksvariabelen/uitkomstmaten (indien van toepassing):
Secundaire uitkomstmaten:
- Distant-disease free survival
- Overall survival
- Later uitgevoerde okselbehandeling
- Lokaal recidief percentage
- Overig-regionaal recidief percentage
- Contralaterale borstkanker
- Percentage (neo)adjuvante systemische therapie
- Kwaliteit van leven en okselbehandeling-gerelateerde morbiditeit

Omschrijving en inschatting van belasting en risico (indien van toepassing):

Wat betreft belasting is er in deze studie géén sprake van extra diagnostiek of interventies (juist minder) en hoeft de patiënt niet vaker dan normaal voor controle naar het ziekenhuis. De eventuele extra belasting voor de patiënt zal alleen bestaan uit het invullen van kwaliteit van leven vragenlijsten (bij 700 van 1644 patiënten) éénmaal voor de randomisatie en vijfmaal na randomisatie (6 maanden, 1 jaar, 2 jaar, 5 jaar en 10 jaar). De hypothese luidt dat het achterwege laten van de schildwachtklierprocedure even veilig is als het wél uitvoeren van deze procedure. Desondanks is er een risico dat in de experimentele arm meer regionale recidieven optreden en dat bij patiënten op een later tijdstip alsnog een vorm van okselklierbehandeling moet worden uitgevoerd. Daartegenover staat dat er in de experimentele arm door het niet uitvoeren van de schildwachtklierprocedure minder kans is op morbiditeit, minder operaties en opnames in het ziekenhuis en een betere kwaliteit van leven. Patiënten zullen op geen enkele manier vertraging ondervinden als gevolg van deelname aan de studie.

K2.Engelse Samenvatting

Background of the study:
The NSABP B-04 trial revealed that omitting primary axillary treatment of occult positive lymph nodes in clinically node negative breast cancer patients does not affect survival, even after 25 years of follow-up and without the use of adjuvant systemic or radiation therapy. A delayed axillary dissection in case lymph nodes become clinically positive does not affect survival and prevents axillary overtreatment in the majority of patients. The ACOSOG Z0011 and IBCSG 23-01 trials revealed that completion axillary dissection can be safely omitted in clinically node negative patients with metastatic sentinel nodes. Patients randomized for watchful waiting were likely to have residual nodal disease (13-27%) that was not surgically removed. Nevertheless, survival rates were not affected and regional recurrence rates low. Use of axillary ultrasound will improve the preoperative selection of node negative patients, as it selects patients with a more favourable tumour load and accurately excludes advanced nodal disease (≥4 metastatic nodes). Biology and adjuvant systemic and radiation therapy are all factors that most likely diminish the risk that possible metastases left in situ develop into clinically detectable lymph nodes. Patients treated with breast conserving therapy are significantly more often diagnosed with pathologic node negative disease or with micrometastatic disease, compared to patients treated with mastectomy. The risk for occult (macro)metastases in cT1-2N0 breast cancer patients treated with breast conserving therapy is low: about 88% will be node negative and about 95% of node positive patients will have no lymph node metastases beyond the sentinel node. Reflected by the low regional recurrence rate after a (false) negative sentinel node, only a small amount of patients (0.8%) with node positive disease is expected to develop clinically detectable nodal disease.

Objective of the study:
The objective of this study is to decrease the number of breast cancer patients receiving overtreatment of the axilla, in order to positively influence the axillary morbidity rate and quality of life. Therefore, we investigate whether the sentinel lymph node procedure can be safely omitted in clinically node negative breast cancer patients undergoing breast conserving therapy, in terms of non-inferior regional control, distant-disease free and overall survival. All objectives are measured during a follow-up of 10 years.

Study design:
A randomized controlled non-inferiority multicenter study. Patients who meet the in- and exclusioncriteria will be randomized after informed consent for one of the two treatment methods:
- Arm A (control arm): lumpectomy with sentinel lymph node procedure, followed by radiotherapy of the breast with or without completion axillary treatment according to the Dutch breast cancer guideline.

- Arm B (experimental arm): lumpectomy without further axillary staging, followed by radiotherapy of the breast.
Stratification: age (≤50, 50≤75, >75 years old), oestrogen receptor status (positive vs. negative), HER2neu status (amplified vs. not amplified), clinical tumour stage prior to any treatment (<3 cm vs. ≥3 cm), grading (grade I-II vs. III - according to Bloom-Richardson grading system), is or will be treated with primary systemic therapy (yes/no) and participating centre.
Adjuvant systemic treatment is administered if indicated according to the Dutch breast cancer guideline.
Primary systemic therapy is allowed if the patient has a clinical T1-2N0 status (initial stage) that is amenable to lumpectomy pre-systemic therapy.
Yearly follow-up with physical examination of the axilla for 10 years. Axillary ultrasound (+/- tissue sampling) on indication. Staging for distant metastases and/or a delayed axillary lymph node dissection if indicated by the multidisciplinary team. Validated questionnaires are used to assess axillary morbidity rate and quality of life (Lymph-ICF, QLQ-C30, QLQ-BR23) and to measure if anxiety and personality traits influence the outcome of quality of life (STAI-trait, NEO-FFI) in 700 of 878 patients. These questionnaires are provided before randomization and sequentially post randomization at 6 months, 1, 2, 5 and 10 years.

Study population:
Women aged 18 years or older with pathologically confirmed invasive breast carcinoma about to undergo breast conserving surgery followed by radiotherapy of the breast.
cT1-2: primary tumour is smaller than 5 cm.
cN0: no signs of axillary lymph node metastases at physical examination and axillary ultrasound (+/- tissue sampling).
Primary systemic treatment is allowed.
Patient is able to and signed the informed consent form.

Intervention (if applicable):
Patients in the control arm will be treated with the sentinel lymph node procedure according to the Dutch breast cancer guideline.
Patients in the experimental arm will not be treated with the sentinel lymph node procedure.

Primary study parameters/outcome of the study:
Primary endpoint:
- Regional recurrence rate

Secundary study parameters/outcome of the study (if applicable):
Secondary endpoints:
- Distant-disease free survival
- Overall survival
- Delayed axillary treatment
- Local recurrence rate
- Other-regional recurrence rate
- Contralateral breast cancer rate
- Percentage difference in the administration of (neo)adjuvant systemic therapy
- Quality of life and axillary morbidity rate

Nature and extent of the burden and risks associated with participation, benefit and group relatedness (if applicable):
There is no extra burden for participating patients as regard to additional diagnostic tests/interventions and follow-up. Any additional burden for the patient will only consist of completing quality of life questionnaires in 700 of 1644 study patients. These questionnaires are provided before randomization and sequentially post randomization at 6 months, 1, 2, 5 and 10 years.
We hypothesize that omitting the sentinel lymph node procedure is not inferior to the standard treatment. Nevertheless, possible disadvantages are a worse regional recurrence rate, and delayed axillary treatment if indicated. Patients in the experimental arm have the possible advantages of less morbidity, fewer operations and hospitalizations and an improved quality of life, compared to patients treated with the sentinel lymph node procedure.
No patient will encounter any delay in their treatment as a result of inclusion.

ONDERTEKENING

De verrichter en indiener verklaren hierbij:
a. het formulier (en samenvatting) volledig en naar waarheid te hebben ingevuld;
b. de antwoorden op de vragen uit het ABR-formulier niet in strijd zijn met het bijbehorende onderzoeksdossier en onderzoekscontract

Naar waarheid getekend, door de verrichter (=opdrachtgever)		door de indiener	
datum	....	datum	....
Handtekening		Handtekening	
naam	U.P. Neumann	naam	J.A. van der Hage
functie	Hoofd afdeling Chirurgie, MUMC+	functie	Chirurg, NKI-AVL

Sluiten Print

Sluiten Print

Formulier voor medisch-ethische beoordeling en registratie

ABR-formulier, versie december 2015

Onderzoeksdossiernummer

ABR Nummer	**49315**	Status	**Definitief**
Versie	**06**	Status per	**04-10-2016**
Jaar	**16**		
Dossiernummer	**NL49315.031.14**		
Reden voor Amendement	**Toevoeging kosteneffectiviteit en mantelzorger**		

A. Sectie - Openbaar maken gegevens medisch wetenschappelijk onderzoek

A1. Het CCMO-register is een voor ieder toegankelijk openbaar trial register. De antwoorden op de vragen gemarkeerd met een wereldbol en de samenvatting bij dit formulier worden openbaar gemaakt in het CCMO register.

B. Sectie - Administratief

B1. Betreft het onderzoek met geneesmiddelen (inclusief gentherapie, somatische celtherapie, vaccinonderzoek, GGO's, zie verdere toelichting) als bedoeld in de Wet medisch-wetenschappelijk onderzoek met mensen (WMO)?

○ ja
◉ nee

B2. Houdt het onderzoek verband met een eerder door een erkende METC of door de CCMO beoordeelde studie of is het onderzoek reeds eerder bij een erkende METC ter beoordeling voorgelegd?

○ ja, het onderzoek houdt verband met – of is het vervolg op – een eerder beoordeelde studie
○ ja, het onderzoek is eerder ter beoordeling aan een erkende METC of de CCMO voorgelegd (stuur kopie besluit mee)
◉ nee

B4. Is het protocol (nog) in een ander openbaar trial register geregistreerd?

○ ja
◉ nee

B5. Naam indiener/contactpersoon voor de oordelende toetsingscommissie

**B5a.**	Achternaam indiener/contactpersoon	Hage
	Titel en voorletters	dr. J.A.
	Tussenvoegsels	van der
**B5b.**	Type organisatie/bedrijf	Overige Ziekenhuizen
	Organisatie/bedrijf	Antoni van Leeuwenhoek Ziekenhuis
	Naam organisatie/bedrijf	IKNL trialbureau
	Afdeling	Chirurgie
	Adres	Plesmanlaan 121
	Postcode en plaats	1066 CX Amsterdam
	Land	NL
**B5c.**	Intern adres	afdeling Heelkunde
	Telefoon	020 512 9111
	Fax	020 512 2554
	E-mail	j.vd.hage@nki.nl

B6. Is de indiener werkzaam bij de opdrachtgever/sponsor (verrichter) van het onderzoek?

○ Ja ◉ Nee

B7. Opdrachtgever/sponsor van het onderzoek (verrichter volgens de WMO)

Type Organisatie/Bedrijf	Universitair Medisch Centrum
Organisatie/Bedrijf	Medisch Universitair Ziekenhuis Maastricht

Adres	P. Debyelaan 25
Postcode en plaats	6229 HX Maastricht
Land	NL
Telefoon	0433877477
Fax	0433875473
E-mail	m.smidt@mumc.nl

C. Sectie - Onderzoek

C1. Volledige titel van het onderzoek

C1a. In het Engels

Clinically node negative breast cancer patients undergoing breast conserving therapy: Sentinel lymph node procedure versus follow-up. A Dutch randomized controlled multicentre trial.

C1b. In het Nederlands

Patiënten met borstkanker en een klinisch negatieve okselklierstatus die een borstsparende behandeling ondergaan: Schildwachtklierprocedure versus follow-up. Een Nederlandse gerandomiseerde multicenter studie.

C2. Verkorte titel van het onderzoek/acroniem

C2a. In het Engels

BOOG 2013-08

C2b. In het Nederlands (Let op: deze korte titel wordt vermeld binnen ToetsingOnline)

BOOG 2013-08

C3. Trefwoorden (maximaal 4, plaats elk trefwoord op een aparte regel)

C3a. In het Engels

Breast neoplasms
Breast conserving therapy
Sentinel lymph node biopsy
Axilla

C3b. In het Nederlands

Borstkanker
Borstsparende behandeling
Schildwachtklierprocedure
Oksel

C4. Beschrijf het belang van het onderzoek en de beoogde toepassing van de resultaten (verwijs eventueel naar de relevante pagina's in het protocol).

Het doel van dit onderzoek is om:
1) Overbehandeling van de oksel te verminderen;
2) Het aantal korte en lange termijn complicaties te verminderen;
3) De kwaliteit van leven te verbeteren;
4) Met een even goede ziektecontrole en algehele overleving

Dit willen wij bereiken door te onderzoeken of de schildwachtklierprocedure veilig achterwege gelaten kan worden bij patiënten met borstkanker en een klinisch negatieve okselklierstatus behandeld met borstsparende therapie.

C6. Betreft het onderzoek een multicenter-onderzoek?

- ○ nee
- ◉ ja - alleen in Nederland
- ○ ja - internationaal binnen de Europese Unie
- ○ ja - internationaal ook buiten de Europese Unie

C7. Is er bij multicenter-onderzoek sprake van een coördinerend onderzoeker?

- ◉ ja namelijk — Naam onderzoeker: M.L. Smidt
- ○ nee

C8. Wie is/zijn medisch verantwoordelijk voor de proefpersonen die deelnemen aan het onderzoek

Behandelend arts en lokale hoofdonderzoeker

C9. In welk centrum/welke centra (incl. huisartsenpraktijken) in Nederland wordt het onderzoek uitgevoerd?

Centrum	Proefpersonen	Hoofdonderzoeker	Onafhankelijk arts
Albert Schweitzer Ziekenhuis	49	M.B.E. Menke-Pluijmers	S.O. Breukink (Maastricht UMC+)
Amphia Ziekenhuis	69	E.J.T. Luiten	S.O. Breukink (Maastricht UMC+)

Antonius Ziekenhuis, Sneek	56	C.G.J.M. van Riel	S.O. Breukink (Maastricht UMC+)
Atrium Medisch Centrum	39	E.R.M. van Haaren	S.O. Breukink (Maastricht UMC+)
Bronovo Ziekenhuis	23	O.R. Guicherit	S.O. Breukink (Maastricht UMC+)
Canisius Wilhelmina Ziekenhuis	46	L.J.A. Strobbe	S.O. Breukink (Maastricht UMC+)
Catharina-ziekenhuis	46	G.A.P. Nieuwenhuijzen	S.O. Breukink (Maastricht UMC+)
Deventer Ziekenhuis	39	H. de Vries	S.O. Breukink (Maastricht UMC+)
Zorggroep De Sionsberg	15	K. Blaauwendraat	S.O. Breukink (Maastricht UMC+)
Diakonessenhuis Utrecht	39	T. van Dalen	S.O. Breukink (Maastricht UMC+)
Gelre Ziekenhuizen	37	W.B. Lastdrager	S.O. Breukink (Maastricht UMC+)
Groene Hart Ziekenhuis	31	E. Linthorst	S.O. Breukink (Maastricht UMC+)
HagaZiekenhuis	41	I. Jannink	S.O. Breukink (Maastricht UMC+)
Ikazia Ziekenhuis	27	B.R. Toorenvliet	S.O. Breukink (Maastricht UMC+)
Isala Klinieken	54	A.B. Francken	S.O. Breukink (Maastricht UMC+)
Kennemer Gasthuis	43	R.J.C.L.M. Vuylsteke	S.O. Breukink (Maastricht UMC+)
LangeLand Ziekenhuis	14	B. Jas	S.O. Breukink (Maastricht UMC+)
Laurentius Ziekenhuis	23	A.V.R.J. Bell	S.O. Breukink (Maastricht UMC+)
Academisch Ziekenhuis Maastricht	39	M.L. Smidt	S.O. Breukink (Maastricht UMC+)
Martini Ziekenhuis	43	W. Kelder	S.O. Breukink (Maastricht UMC+)
Maxima Medisch Centrum	31	K. Schenk	S.O. Breukink (Maastricht UMC+)
Meander Medisch Centrum	43	A.J. van Overbeeke	S.O. Breukink (Maastricht UMC+)
Antoni van Leeuwenhoek Ziekenhuis	93	J.A. van der Hage	S.O. Breukink (Maastricht UMC+)
Onze Lieve Vrouwe Gasthuis	28	P.J. Borgstein	S.O. Breukink (Maastricht UMC+)
Orbis Medisch Centrum	23	Y. Vissers	S.O. Breukink (Maastricht UMC+)
Universitair Medisch Centrum Sint Radboud	31	J.H.W. de Wilt	S.O. Breukink (Maastricht UMC+)
Reinier de Graaf Groep	46	M. van 't Riet	S.O. Breukink (Maastricht UMC+)
Sint Antonius Ziekenhuis	58	R. Koelemij	S.O. Breukink (Maastricht UMC+)
Sint Elisabeth Ziekenhuis	37	P. Jansen	S.O. Breukink (Maastricht UMC+)
Streekziekenhuis Koningin Beatrix	19	A. Jonk	S.O. Breukink (Maastricht UMC+)
Tergooiziekenhuizen locatie Blaricum	42	E.J.C. Vriens	S.O. Breukink (Maastricht UMC+)
Universitair Medisch Centrum Groningen	17	L. Jansen	S.O. Breukink (Maastricht UMC+)
Universitair Medisch Centrum Utrecht	23	A.J. Witkamp	S.O. Breukink (Maastricht UMC+)
Viecuri Medisch Centrum voor Noord-Limburg	39	C.L.H. van Berlo	S.O. Breukink (Maastricht UMC+)
Westfries Gasthuis	27	J.W.D. de Waard	S.O. Breukink (Maastricht UMC+)
Waterland Ziekenhuis	22	L. de Widt	S.O. Breukink (Maastricht UMC+)
Zaans Medisch Centrum	15	S. Muller	S.O. Breukink (Maastricht UMC+)
Ziekenhuisvoorzieningen Gelderse Vallei	32	W.K. de Roos	S.O. Breukink (Maastricht UMC+)
Ziekenhuisgroep Twente	31	D. de Leeuw	S.O. Breukink (Maastricht UMC+)

Rivierenland Ziekenhuis	23	M.A.J. de Roos	S.O. Breukink (Maastricht UMC+)
Medisch Centrum Alkmaar	54	W.H. Schreurs	S.O. Breukink (Maastricht UMC+)
Medisch Centrum Haaglanden	31	A. Marinelli	S.O. Breukink (Maastricht UMC+)
Medisch Spectrum Twente	46	P. Steenvoorde	S.O. Breukink (Maastricht UMC+)
Rijnstate Ziekenhuis	62	R. van Eekeren	S.O. Breukink (Maastricht UMC+)
Flevoziekenhuis	50	S. van der Meij	S.O. Breukink (Maastricht UMC+)
Zuwehofpoort	15	R.C. van Doorn	S.O. Breukink (Maastricht UMC+)
Jeroen Bosch Ziekenhuis	20	M. Bessem	B. Biesma (Jeroen Bosch ziekenhuis)
Alrijne ziekenhuis	50	P. Neijenhuis	S.O. Breukink (Maastricht UMC+)
Kennemer Gasthuis	30	B. Kortmann	S.O. Breukink (Maastricht UMC+)

C10. Betreft het onderzoek met:

- ☑ mensen
- ☐ geslachtscellen
- ☐ (rest-)embryo's
- ☐ foetussen in utero

C11. Beoogd totaal aantal proefpersonen/(rest)embryo's/foetussen in utero:

C11a. In Nederland

1644

C13. Onderzoeksgebied

- ☐ etiologie
- ☐ organisatorisch/zorgonderzoek
- ☐ diagnostiek
- ☐ preventie
- ☑ therapie
- ☐ veiligheid
- ☐ werkzaamheid
- ☐ farmacokinetiek
- ☐ farmacodynamiek
- ☐ bio-equivalentie
- ☐ dosis-respons
- ☐ farmacogenomics
- ☐ farmaco-economie
- ☐ anders

C14. Type onderzoek

- ○ observationeel onderzoek zonder invasieve metingen
- ○ observationeel onderzoek met invasieve metingen
- ◉ interventie-onderzoek

C15. In welke fase kan het onderzoek worden ingedeeld?

- ○ fase I (a)
- ○ fase II (b)
- ◉ fase III (c)
- ○ fase IV (d)
- ○ overige onderzoeken waarbij geneesmiddelen worden toegepast (e)
- ○ niet van toepassing

C17. Is er sprake van een andere interventie dan met geneesmiddelen zoals bedoeld in de Wet medisch-wetenschappelijk onderzoek met mensen (WMO) (zie toelichting)?

- [x] Ja
- [ ] Nee

C17a. ☐ Medisch hulpmiddel, namelijk
C17b. ☐ Operatie, namelijk
C17c. ☐ Psychosociale interventie, namelijk
C17d. ☐ Voeding(stoffen), namelijk
C17e. ☐ Bewegingstherapie, namelijk
C17f. ☐ interventie met radioactieve straling, namelijk
C17g. ☐ Blootstellingsonderzoek (bijv pesticidenonderzoek), namelijk
C17h.
C17i. ☑ andere interventie, namelijk

In het Engels: No sentinel lymph node procedure
In het Nederlands: Géén schildwachtklierprocedure

C18. Worden de onderzoeksproducten voor deze studie door de verrichter gratis verstrekt?

- [ ] ja
- [ ] nee
- [ ] gedeeltelijk namelijk
- [x] niet van toepassing

C19. Is/zijn er (een) controlegroep (en)?

- [x] ja
- [ ] nee

- [ ] ja, geneesmiddel
- [ ] ja, placebo
- [ ] ja, geen interventie
- [x] ja, anders

namelijkDe standaard behandeling: schildwachtklierprocedure

C20. Betreft het een gerandomiseerd onderzoek?

- [x] ja
- [ ] nee

namelijk

- [x] open
- [ ] enkelblind
- [ ] dubbelblind
- [ ] parallel
- [ ] cross-over
- [ ] anders namelijk

C21. Op welke klasse(n) van aandoeningen heeft het onderzoek betrekking (maximaal 3)

- [ ] hartaandoeningen
- [ ] congenitale, familiaire en genetische aandoeningen
- [ ] bloed- en lymfestelsel aandoeningen
- [ ] zenuwstelsel aandoeningen
- [ ] oogaandoeningen
- [ ] evenwichtsorgaan- en ooraandoeningen
- [ ] ademhalingsstelsel-, thorax- en mediastinumaandoeningen
- [ ] maagdarmstelselaandoeningen
- [ ] nier- en urinewegaandoeningen
- [ ] huid- en onderhuidaandoeningen

- ☐ skeletspierstelsel- en bindweefselaandoeningen
- ☐ endocriene aandoeningen
- ☐ voedingsstoornissen en metabole ziekten
- ☐ infecties en parasitaire aandoeningen
- ☐ letsels, intoxicaties en verrichtingscomplicaties
- ☑ neoplasmata, benigne, maligne en niet-gespecificeerd (incl cysten en poliepen)
  borstneoplasmata maligne en niet-gespecificeerd (incl. tepel)
- ☑ chirurgische en medische verrichtingen
  borst therapeutische verrichtingen
- ☐ bloedvataandoeningen
- ☐ algemene aandoeningen en aandoeningen op de plek van toediening
- ☐ zwangerschap, perinatale periode en puerperium
- ☐ sociale omstandigheden
- ☐ immuunsysteemaandoeningen
- ☐ lever- en galaandoeningen
- ☐ voortplantingsstelsel- en borstaandoeningen
- ☐ psychische stoornissen
- ☐ overig, namelijk

C22.Geef twee synoniemen voor de aandoening die bestudeerd wordt, waarvan tenminste één lekenterm.

In het Engels:	breast cancer, sentinel lymph nodes, sentinel lymph node metastases
In het Nederlands:	borstkanker, schildwachtklieren, schildwachtkliermetastasen

C23. Beoogde start- en einddatum van het onderzoek

C23a. Start Datum (dd-mm-jjjj)	01-01-2015
C23b. Eind Datum (dd-mm-jjjj)	01-01-2027

C24. Zijn patiënten betrokken bij het ontwikkelen van het onderzoek? (Voor patiënten lees ook: patiëntenorganisaties, naasten of nabestaanden van patiënten en gezonde vrijwilligers)?

C30. Wordt de ervaring van proefpersonen na deelname aan het onderzoek geëvalueerd?

D. Sectie - Proefpersonen

D1. Is er een proefpersonenverzekering conform de WMO-eisen afgesloten of wordt aan de oordelende toetsingscommissie ontheffing gevraagd?

- ◉ proefpersonenverzekering is afgesloten bij verzekeringsmaatschappij
- ○ ontheffing van de verzekering wordt gevraagd
- ○ niet van toepassing - onderzoek valt onder de Embryowet en niet onder de WMO

Verzekeringsmaatschappij	Marketform (onderdeel van Lloyd's)

D2. Gezonde proefpersonen en/of patiënten

- ☐ Gezonde proefpersonen

- ☑ Patiënten

Aantal	1644

D4. Voornaamste inclusiecriteria

D4a. In het Engels
- Female
- Aged 18 years or older
- Pathologically confirmed invasive unilateral breast carcinoma
- A clinical T1-2 tumour (≤ 5cm)
- Will be treated with lumpectomy followed by whole breast radiotherapy
- Clinically node negative status: no signs of axillary lymph node metastases at physical examination and preoperative axillary ultrasound (or negative cyto-/histopathology)
- Written informed consent

D4b. In het Nederlands
- Vrouw
- Leeftijd 18 jaar of ouder
- Pathologisch bewezen invasief unilaterale borstkanker
- Klinisch T1-2 tumor (≤ 5cm)
- Zal worden behandeld met lumpectomie gevolgd door gehele borstbestraling

- Klinisch negatieve okselklierstatus: negatief lichamelijk onderzoek en echografie van de oksel (of negatieve cyto-/histologie)
- Schriftelijke informed consent

D5. Voornaamste exclusiecriteria

D5a. In het Engels

- Clinically node positive pre-operative
- Bilateral breast cancer
- Evidence of metastatic disease
- History of invasive breast cancer
- Previous treatment of the ipsilateral axilla with surgery or radiotherapy, except surgery for hidradenitis suppurativa or for other superficially located skin lesions, such as naevi
- Pregnant or nursing
- Other prior malignancies within the past 5 years, except successfully treated malignancies that occurred more than five years before randomization, and except successfully treated basal cell and squamous cell skin cancer, carcinoma in situ of the cervix or carcinoma in situ of the ipsilateral or contralateral breast
- Unable or unwilling to give informed consent

D5b. In het Nederlands

- Klinisch positieve okselklierstatus (preoperatief)
- Bilaterale borstkanker
- Primair (afstands-)gemetastaseerde borstkanker
- Invasieve borstkanker in de voorgeschiedenis
- Chirurgie of radiotherapie van ipsilaterale oksel in de voorgeschiedenis, behalve chirurgie voor hidradenitis suppurativa of andere oppervlakkig gelegen huidlaesies, zoals naevi
- Zwanger of lacterend
- Andere maligniteit in de afgelopen 5 jaar, behalve succesvol behandelde maligniteit die meer dan vijf jaar voor randomisatie optrad, en behalve succesvol behandelde huidkanker van type basaalcel of plaveiselcel, carcinoma in situ van de cervix, carcinoma in situ van de ipsilaterale of contralaterale mamma
- Niet in staat of niet bereid tot informed consent

D6. Bij welke categorie proefpersonen wordt het onderzoek uitgevoerd (meerdere antwoorden mogelijk)

☑ 18 jaar of ouder en wilsbekwaam
☐ 18 jaar of ouder en wilsonbekwaam
☐ 12 t/m 17 jaar en in staat tot het geven van geïnformeerde toestemming
☐ 12 t/m 17 jaar en niet in staat tot het geven van geïnformeerde toestemming (wilsonbekwaam)
☐ jonger dan 12 jaar

D10. Verkeren (sommige) proefpersonen in een afhankelijkheidssituatie ten opzichte van de onderzoeker of degene die de deelnemers werft? (lees de toelichting voor voorbeelden wanneer er sprake kan zijn van een afhankelijkheidssituatie)

◉ ja
○ nee

D10a. Zo ja, waardoor?

Soms is de lokale onderzoeker ook de behandelend arts die de patiënt vraagt om deel te nemen aan de studie.

D10b. Waarom wordt het onderzoek juist met deze proefpersonen uitgevoerd en hoe worden de belangen van de proefpersonen gewaarborgd?

Het onderzoek moet met proefpersonen worden uitgevoerd die voldoen aan de beschreven in/exclusie criteria zodat de resultaten van de studie uiteindelijk ook naar de kliniek vertaald kunnen worden binnen deze patiëntenpopulatie. De belangen van de proefpersonen worden gewaarborgd door de geldende medisch ethische normen en richtlijn zoals vermeld in het protocol (o.a. onafhankelijk arts, uitgebreide patiëntinformatiefolder).

D11. Waaruit bestaat de vergoeding voor de proefpersonen?

☑ geen vergoeding
☐ reiskosten
☐ financiële vergoeding (in Euro's)
☐ andere vergoeding

D12. Is deze vergoeding afhankelijk van bepaalde voorwaarden, bijvoorbeeld het voltooien van (een deel van) het onderzoek?

○ ja (motiveer)
○ nee
◉ niet van toepassing

E. Sectie - Voor- en nadelen

E1. Wordt er bij dit onderzoek een rechtstreeks therapeutisch effect beoogd bij de proefpersonen / patiënten?

◉ ja (therapeutisch onderzoek)

○ nee (niet-therapeutisch onderzoek)

E1a. Zo ja, geef kort aan waaruit dit therapeutisch effect bestaat.

Het therapeutische effect bestaat uit:
- Het verlagen van het risico op okselbehandeling-gerelateerde korte en lange termijn complicaties;
- Het verbeteren van de kwaliteit van leven;
- Met een even goede ziektecontrole en algehele overleving.

E2. Waaruit bestaat de belasting van het onderzoek (en een eventueel daaraan voorafgaande keuring) voor de proefpersonen?

Tijdbeslag	per bezoek	20 min per keer voor invullen kwaliteit van leven vragenlijst
	totaal	20 min x 6 = 120 min
	totale duur van de studie voor de individuele proefpersoon	Tav de behandeling minder belasting dan normaal voor de studie-arm (geen schildwachtklierprocedure). Voor beide armen is extra het invullen van de kwaliteit van leven vragenlijst prerandomisatie en postrandomisatie op 5 momenten.

E3. Worden de proefpersonen in verband met het onderzoek in het ziekenhuis opgenomen of wordt een opname verlengd?

○ ja - het verblijf in het ziekenhuis/instituut wordt in verband met het onderzoek verlengd

○ ja - ze worden voor het onderzoek in het ziekenhuis/instituut opgenomen

◉ nee

E4. Beschrijf in hoeverre proefpersonen worden onderworpen aan handelingen dan wel een gedragswijze krijgen opgelegd, zoals vragenlijst, interviews, lichamelijk/psychologisch onderzoek, ontzegging, dieet (voor invasieve ingrepen: zie vraag E6)

In totaal zullen 700 proefpersonen (350 in de experimentele en 350 in de controlegroep) deelnemen aan het kwaliteit van leven onderzoek, bestaande uit een vragenlijst voor de randomisatie en op 5 tijdstippen na de randomisatie (na een half jaar, één, twee, vijf en 10 jaar).

E5. Worden de proefpersonen getest op bepaalde aandoeningen/condities?

○ ja (motiveer)

◉ nee

E6. Welke extra (invasieve) ingrepen (anders dan bij de standaard behandeling) moeten de proefpersonen in het kader van het onderzoek ondergaan:

☑ Niet van toepassing

E9. Geef aan welke risico's er voor proefpersonen zijn verbonden aan deelname aan het onderzoek.

Hoewel de hypothese luidt dat het achterwege laten van de schildwachtklierprocedure geen nadelige invloed heeft op de lokale controle van de ziekte en de overlevingskans, bestaat er toch een kans dat proefpersonen in de experimentele arm een hoger risico hebben op een recidief in de oksel (regionaal recidief) en daardoor tijdens follow-up alsnog een vorm van okselklierbehandeling moeten ondergaan. Uit onderzoek blijkt dat het uitgesteld uitvoeren van okselklierbehandeling (bijvoorbeeld met okselklierdissectie) géén nadelige gevolgen heeft voor de overleving.

E9a. Geef op grond van uw eigen afweging aan waarom het uitvoeren van het onderzoek, in het licht van de belasting en/of risico's die voor proefpersonen aan deelname verbonden zijn, gerechtvaardigd is?

Het mogelijk hogere risico op een recidief in de oksel (regionaal recidief) weegt op tegen de lagere kans op ernstige okselklierbehandeling-gerelateerde morbiditeit (lymfoedeem, bewegingsbeperking in de schouder, arm- en schouderpijn, verminderd gevoel of zenuwpijn) en de verbetering van kwaliteit van leven.

E10. Indien het onderzoek bij minderjarige en/of wilsonbekwame proefpersonen wordt uitgevoerd en geen direct therapeutisch effect wordt beoogd: waarom kunnen belasting en risico's als minimaal worden beschouwd? (Verwijs eventueel naar de relevante pagina's in het protocol.

☑ niet van toepassing

E11. Kan de eventuele therapie na beëindiging van het onderzoek worden voortgezet?

○ ja (motiveer)

○ nee (motiveer)

◉ niet van toepassing

E12. Heeft deelname aan het onderzoek voor de proefpersoon tot gevolg dat van de standaardbehandeling of -diagnostiek kan worden afgeweken of deze kan worden uitgesteld?

◉ ja
○ nee
○ niet van toepassing

E12a. Zo ja, waaruit bestaat de afwijking of het uitstel en waarom is afwijking/uitstel
Proefpersonen in de controlegroep ondergaan de standaard behandeling, namelijk schildwachtklierprocedure volgens de Nederlandse borstkankerrichtlijn. Proefpersonen in de experimentele arm ondergaan primair géén schildwachtklierprocedure. Beide groepen ondergaan dezelfde follow-up middels lichamelijk onderzoek en aanvullend onderzoek op indicatie. Indien een proefpersoon zich in de follow-up periode presenteert met lymfekliermetastasen, kan alsnog besloten worden om een vorm van okselklierbehandeling uit te voeren. Deze beslissing wordt gemaakt tijdens het multidisciplinair overleg in het desbetreffende centrum waar de patiënt onder behandeling staat.

F. Sectie - Informatie en privacy

F1. Hoe worden de proefpersonen geworven en door wie (onderzoeker, behandelend arts, andere persoon) wordt de proefpersoon/wettelijke vertegenwoordiger geïnformeerd en om toestemming gevraagd?
Op het moment in de behandeling van borstkanker dat de patiënt voldoet aan de in/exclusiecriteria zal de behandeld arts informatie geven over de mogelijkheid tot deelname aan deze studie. Een schriftelijke patiëntinformatiebrief zal worden meegegeven aan de patiënt. De patiënt krijgt ruim de tijd (ten minste één week) om deze informatie thuis na te lezen en een beslissing te nemen. De patiënt kan haar interesse in deelname kenbaar maken aan haar behandelend arts bij een volgend polibezoek. De behandelend arts zal haar dan om toestemming vragen voor deelname aan de studie.

F2. Hoeveel bedenktijd krijgen de proefpersonen/wettelijke vertegenwoordigers om te beslissen over deelname?
Zo lang als nodig, met ten minste één week

F3. Wordt de huisarts, behandelend specialist en/of apotheker van de proefpersoon geïnformeerd over diens deelname aan het onderzoek?

◉ ja (de proefpersoon dient hiervoor toestemming te geven)
○ nee

F4. Worden persoonsgegevens gecodeerd?

◉ ja
○ nee

F4a. Zo ja, hoe is deze codering opgebouwd?
Alle proefpersonen krijgen een uniek studienummer. Het unieke studienummer betreft een opvolgnummer zonder verdere specifieke codering voor bijvoorbeeld centrum.

F4b. Wie heeft toegang tot de sleutel van deze code?
De behandelend arts en de onderzoeker

F4c. Wie hebben toegang tot de brondocumenten en eventuele andere tot de persoon herleidbare gegevens?
De lokaal datamanagers en de onderzoeker

F5. Hoe wordt het lichaamsmateriaal <u>gedurende</u> het onderzoek bewaard?

○ in tot de proefpersoon herleidbare vorm (gecodeerd)
○ in niet tot de proefpersoon herleidbare vorm (volledig geanonimiseerd)
◉ niet van toepassing

F6. Wordt afgenomen lichaamsmateriaal na afloop van het onderzoek vernietigd?

○ ja
○ nee (motiveer)
◉ niet van toepassing

F7. Kunnen proefpersonen na afloop van het onderzoek opnieuw benaderd worden (bijvoorbeeld voor nader onderzoek of follow-up)?

○ ja
◉ nee

G. Sectie - Financieel

G1. Door welke geldstroom wordt het onderzoek gefinancierd?

☐ eerste geldstroom (Geld van Ministerie van OC&W aan universiteiten)
☐ tweede geldstroom (NWO of KNAW), namelijk

☑ derde geldstroom (anders dan 1e of 2e geldstroom, zoals collectebusfondsen, Europese Unie, vakministeries of bedrijven), namelijk
KWF Kankerbestrijding

G2. Wordt het onderzoek (mede) gefinancierd door de industrie/bedrijven?

- ○ ja - door de industrie/bedrijf zoals is opgegeven bij vraag B6/B7 (opdrachtgever van het onderzoek)
- ○ ja - (ook) door andere industrie/bedrijven dan de opdrachtgever
- ◉ nee

G3. Wat is de hoogte van de vergoeding die de arts/onderzoeker cq onderzoeksafdeling/maatschap ontvangt voor de uitvoering van het onderzoek?

- ○ Per patiënt of proefpersoon
- ◉ Per deelnemend centrum

bedrag afgerond in hele euro's: € 100,00

G3a. Hoe is de vergoeding opgebouwd?

De vergoeding betreft een financiële compensatie van € 100 excl. BTW als opstartvergoeding voor ieder deelnemend centrum.

G4. Heeft/hebben de onderzoeker(s) gedurende de afgelopen vijf jaar op een of andere wijze een persoonlijke financiële relatie (gehad) met de verrichter/sponsor van het huidige onderzoek?

- ○ ja (licht toe)
- ◉ nee

I. Sectie - Indiening en beoordeling

I1. Sla het formulier eerst op en selecteer vervolgens een METC of de CCMO

Commissie
METC NedMec

J. Sectie - Aanvullende opmerkingen

Aanvullende opmerkingen

K. Sectie - Samenvatting

Achtergrond van het onderzoek:

De schildwachtklierprocedure is de standaard methode voor lymfeklierstadiëring in borstkankerpatiënten met een klinisch negatieve okselklierstatus. In de algemene bevolking bevat de schildwachtklier in ongeveer 25% een uitzaaiing. Dit percentage is lager in patiënten met een kleine borsttumor en in borstsparend behandelde patiënten. De ACOSOG-Z0011 studie en de IBCSG 23-01 studie hebben recent aangetoond dat een completerende okselklierdissectie veilig achterwege gelaten kan worden bij borstsparend en met systemische therapie behandelde patiënten met een beperkt aantal schildwachtkliermetastasen. Dit roept de vraag op of de schildwachtklierprocedure nog wel noodzakelijk is voor borstsparend behandelde patiënten met een klinisch negatieve okselklierstatus. De klinisch negatieve okselklierstatus van de patiënten in de beschreven studies werd gesteld op basis van lichamelijk onderzoek. Het toevoegen van een preoperatieve echografie van de oksel zal zorgen voor een betere voorselectie van patiënten zonder lymfekliermetastasen, ten opzichte van lichamelijk onderzoek alleen. Daarbij sluit een negatieve echografie uitgevoerd in Nederland bijna alle patiënten uit met uitgebreide lymfekliermetastasen (≥4 lymfekliermetastasen). Bovendien omvat gehele borstbestraling na de borstsparende operatie in het algemeen het lagere deel van de oksel en behandelt daarmee eventueel achtergebleven uitzaaiingen. Hoewel de schildwachtklierprocedure minder invasief is vergeleken met een okselklierdissectie, komen complicaties nog steeds voor. Zo lijdt 6% van de patiënten die alleen een schildwachtklierprocedure ondergingen aan ernstige lymfoedeem na slechts 3 jaar. Deze studie gaat onderzoeken of de schildwachtklierprocedure veilig achterwege gelaten kan worden bij borstsparend behandelde patiënten, met een negatieve echografie van de oksel.

Doel van het onderzoek:

Het doel van deze studie is het verminderen van overbehandeling van de oksel bij patiënten met borstkanker en een klinisch negatieve okselklierstatus die behandeld worden met borstsparende therapie, door het achterwege laten van de schildwachtklierprocedure, om zodoende de kans op korte en lange termijn complicaties te verminderen en te zorgen voor een verbetering van de kwaliteit van leven met een even goede ziektecontrole en algehele overleving.

Onderzoeksopzet:

Het betreft een gerandomiseerde non-inferiority multicenter onderzoek. Patienten die voldoen aan de in- en exclusiecriteria worden na toestemming middels loting behandeld volgens één van de volgende behandelmethoden:

- Behandelmethode A (controle groep): schildwachtklierprocedure volgens de Nederlandse borstkankerrichtlijn.
- Behandelmethode B (studie groep): geen verdere okselklierstadiëring.

Stratificatiefactoren: leeftijd (≤50, 50≤75, >75 jaar), oestrogeen receptor status (positief vs. negatief), HER2neu status (geamplificeerd vs. niet geamplificeerd), klinisch tumor stadium (<3 cm vs. ≥3 cm), tumorgraad volgens gemodificeerde Bloom-Richardson (graad I-II vs. graad III), is of zal worden behandeld met primaire systemische therapie (ja/nee), participerend centrum.
Overige behandelingen (o.a. hormonale therapie, chemotherapie) worden op indicatie gegeven volgens de Nederlandse borstkankerrichtlijn.
Tijdens de follow-up periode van 10 jaar wordt de oksel nauwkeurig onderzocht middels lichamelijk onderzoek en echografie op

indicatie. Indien sprake is van een cyto-/histologisch bewezen lymfekliermetastase tijdens follow-up, dan kan in het multidisciplinair overleg van de desbetreffende patiënt worden besloten tot het uitvoeren van een vorm van okselklierbehandeling.
Gevalideerde vragenlijsten worden verstrekt voor het onderzoeken van de invloed op het aantal complicaties en kwaliteit van leven (Lymph-ICF, QLQ-C30, QLQ-BR23) en om te meten of angst en persoonlijkheidstrekken invloed hebben op de uitkomst van kwaliteit van leven (STAI-trait, NEO-FFI). Deze vragenlijsten worden verstrekt aan 700 van de 1644 patiënten (350 per arm), éénmaal voor de randomisatie en vijfmaal na de randomisatie (6 maanden, 1 jaar, 2 jaar, 5 jaar en 10 jaar).

Onderzoekspopulatie:

Vrouwen van 18 jaar of ouder met pathologisch bewezen invasief mammacarcinoom die borstsparende therapie ondergaan.
cT1-2: primaire tumor is bij klinisch onderzoek kleiner dan 5 cm.
cN0: bij lichamelijk onderzoek en echografie van de oksel worden geen aanwijzingen voor lymfekliermetastasen gevonden of de cytologie/histologie na punctie/biopt van een lymfeklier is negatief.
Neoadjuvante systemische therapie is toegestaan indien de patiënt voor de start van de neoadjuvante systemische therapie een klinische T1-2N0 status heeft en waarvoor borstsparende therapie op dat moment al geschikt is.
De patiënt heeft informed consent persoonlijk ondertekend.

Interventie (indien van toepassing):

De controlegroep ondergaat wél de schildwachtklierprocedure volgens de Nederlands borstkankerrichtlijn. De experimentele arm ondergaan géén schildwachtklierprocedure.

Primaire onderzoeksvariabelen/uitkomstmaten:

Primaire uitkomstmaat:
- Regionaal recidief percentage

Secundaire onderzoeksvariabelen/uitkomstmaten (indien van toepassing):
Secundaire uitkomstmaten:
- Distant-disease free survival
- Overall survival
- Later uitgevoerde okselbehandeling
- Lokaal recidief percentage
- Overig-regionaal recidief percentage
- Contralaterale borstkanker
- Percentage (neo)adjuvante systemische therapie
- Kwaliteit van leven en okselbehandeling-gerelateerde morbiditeit

Omschrijving en inschatting van belasting en risico (indien van toepassing):

Wat betreft belasting is er in deze studie géén sprake van extra diagnostiek of interventies (juist minder) en hoeft de patiënt niet vaker dan normaal voor controle naar het ziekenhuis. De eventuele extra belasting voor de patiënt zal alleen bestaan uit het invullen van kwaliteit van leven vragenlijsten (bij 700 van 1644 patiënten) éénmaal voor de randomisatie en vijfmaal na randomisatie (6 maanden, 1 jaar, 2 jaar, 5 jaar en 10 jaar). De hypothese luidt dat het achterwege laten van de schildwachtklierprocedure even veilig is als het wél uitvoeren van deze procedure. Desondanks is er een risico dat in de experimentele arm meer regionale recidieven optreden en dat bij patiënten op een later tijdstip alsnog een vorm van okselklierbehandeling moet worden uitgevoerd. Daartegenover staat dat er in de experimentele arm door het niet uitvoeren van de schildwachtklierprocedure minder kans is op morbiditeit, minder operaties en opnames in het ziekenhuis en een betere kwaliteit van leven. Patiënten zullen op geen enkele manier vertraging ondervinden als gevolg van deelname aan de studie.

K2.Engelse Samenvatting

Background of the study:
The NSABP B-04 trial revealed that omitting primary axillary treatment of occult positive lymph nodes in clinically node negative breast cancer patients does not affect survival, even after 25 years of follow-up and without the use of adjuvant systemic or radiation therapy. A delayed axillary dissection in case lymph nodes become clinically positive does not affect survival and prevents axillary overtreatment in the majority of patients. The ACOSOG Z0011 and IBCSG 23-01 trials revealed that completion axillary dissection can be safely omitted in clinically node negative patients with metastatic sentinel nodes. Patients randomized for watchful waiting were likely to have residual nodal disease (13-27%) that was not surgically removed. Nevertheless, survival rates were not affected and regional recurrence rates low. Use of axillary ultrasound will improve the preoperative selection of node negative patients, as it selects patients with a more favourable tumour load and accurately excludes advanced nodal disease (≥4 metastatic nodes). Biology and adjuvant systemic and radiation therapy are all factors that most likely diminish the risk that possible metastases left in situ develop into clinically detectable lymph nodes. Patients treated with breast conserving therapy are significantly more often diagnosed with pathologic node negative disease or with micrometastatic disease, compared to patients treated with mastectomy. The risk for occult (macro)metastases in cT1-2N0 breast cancer patients treated with breast conserving therapy is low: about 88% will be node negative and about 95% of node positive patients will have no lymph node metastases beyond the sentinel node. Reflected by the low regional recurrence rate after a (false) negative sentinel node, only a small amount of patients (0.8%) with node positive disease is expected to develop clinically detectable nodal disease.

Objective of the study:
The objective of this study is to decrease the number of breast cancer patients receiving overtreatment of the axilla, in order to positively influence the axillary morbidity rate and quality of life. Therefore, we investigate whether the sentinel lymph node procedure can be safely omitted in clinically node negative breast cancer patients undergoing breast conserving therapy, in terms of non-inferior regional control, distant-disease free and overall survival. All objectives are measured during a follow-up of 10 years.

Study design:
A randomized controlled non-inferiority multicenter study. Patients who meet the in- and exclusioncriteria will be randomized after informed consent for one of the two treatment methods:
- Arm A (control arm): lumpectomy with sentinel lymph node procedure, followed by radiotherapy of the breast with or without completion axillary treatment according to the Dutch breast cancer guideline.
- Arm B (experimental arm): lumpectomy without further axillary staging, followed by radiotherapy of the breast.
Stratification: age (≤50, 50≤75, >75 years old), oestrogen receptor status (positive vs. negative), HER2neu status (amplified vs. not amplified), clinical tumour stage prior to any treatment (<3 cm vs. ≥3 cm), grading (grade I-II vs. III - according to Bloom-Richardson grading system), is or will be treated with primary systemic therapy (yes/no) and participating centre.
Adjuvant systemic treatment is administered if indicated according to the Dutch breast cancer guideline.
Primary systemic therapy is allowed if the patient has a clinical T1-2N0 status (initial stage) that is amenable to lumpectomy pre-systemic therapy.
Yearly follow-up with physical examination of the axilla for 10 years. Axillary ultrasound (+/- tissue sampling) on indication. Staging for distant metastases and/or a delayed axillary lymph node dissection if indicated by the multidisciplinary team. Validated questionnaires are used to assess axillary morbidity rate and quality of life (Lymph-ICF, QLQ-C30, QLQ-BR23) and to measure if anxiety and personality traits influence the outcome of quality of life (STAI-trait, NEO-FFI) in 700 of 878 patients. These questionnaires are provided before randomization and sequentially post randomization at 6 months, 1, 2, 5 and 10 years.

Study population:
Women aged 18 years or older with pathologically confirmed invasive breast carcinoma about to undergo breast conserving surgery followed by radiotherapy of the breast.
cT1-2: primary tumour is smaller than 5 cm.
cN0: no signs of axillary lymph node metastases at physical examination and axillary ultrasound (+/- tissue sampling).
Primary systemic treatment is allowed.
Patient is able to and signed the informed consent form.

Intervention (if applicable):
Patients in the control arm will be treated with the sentinel lymph node procedure according to the Dutch breast cancer guideline.
Patients in the experimental arm will not be treated with the sentinel lymph node procedure.

Primary study parameters/outcome of the study:
Primary endpoint:
- Regional recurrence rate

Secundary study parameters/outcome of the study (if applicable):
Secondary endpoints:
- Distant-disease free survival
- Overall survival
- Delayed axillary treatment
- Local recurrence rate
- Other-regional recurrence rate
- Contralateral breast cancer rate
- Percentage difference in the administration of (neo)adjuvant systemic therapy
- Quality of life and axillary morbidity rate

Nature and extent of the burden and risks associated with participation, benefit and group relatedness (if applicable):
There is no extra burden for participating patients as regard to additional diagnostic tests/interventions and follow-up. Any additional burden for the patient will only consist of completing quality of life questionnaires in 700 of 1644 study patients. These questionnaires are provided before randomization and sequentially post randomization at 6 months, 1, 2, 5 and 10 years.
We hypothesize that omitting the sentinel lymph node procedure is not inferior to the standard treatment. Nevertheless, possible disadvantages are a worse regional recurrence rate, and delayed axillary treatment if indicated. Patients in the experimental arm have the possible advantages of less morbidity, fewer operations and hospitalizations and an improved quality of life, compared to patients treated with the sentinel lymph node procedure.
No patient will encounter any delay in their treatment as a result of inclusion.

ONDERTEKENING

De verrichter en indiener verklaren hierbij:
a. het formulier (en samenvatting) volledig en naar waarheid te hebben ingevuld;
b. de antwoorden op de vragen uit het ABR-formulier niet in strijd zijn met het bijbehorende onderzoeksdossier en onderzoekscontract

Naar waarheid getekend, door de verrichter (=opdrachtgever)	door de indiener
datum	datum ....

Handtekening		Handtekening	
naam	U.P. Neumann	naam	J.A. van der Hage
functie	Hoofd afdeling Chirurgie, MUMC+	functie	Chirurg, NKI-AVL

Sluiten Print

Sluiten Print

Formulier voor medisch-ethische beoordeling en registratie

ABR-formulier, versie december 2015

Onderzoeksdossiernummer

ABR Nummer	**49315**	Status	**Beoordeeld - Positief**
Versie	**07**	Status per	**30-11-2016**
Jaar	**16**		
Dossiernummer	**NL49315.031.14**		
Reden voor	**Aanpassing ABR**		

A. Sectie - Openbaar maken gegevens medisch wetenschappelijk onderzoek

A1. Het CCMO-register is een voor ieder toegankelijk openbaar trial register. De antwoorden op de vragen gemarkeerd met een wereldbol en de samenvatting bij dit formulier worden openbaar gemaakt in het CCMO register.

B. Sectie - Administratief

B1. Betreft het onderzoek met geneesmiddelen (inclusief gentherapie, somatische celtherapie, vaccinonderzoek, GGO's, zie verdere toelichting) als bedoeld in de Wet medisch-wetenschappelijk onderzoek met mensen (WMO)?

○ ja
◉ nee

B2. Houdt het onderzoek verband met een eerder door een erkende METC of door de CCMO beoordeelde studie of is het onderzoek reeds eerder bij een erkende METC ter beoordeling voorgelegd?

○ ja, het onderzoek houdt verband met – of is het vervolg op – een eerder beoordeelde studie
○ ja, het onderzoek is eerder ter beoordeling aan een erkende METC of de CCMO voorgelegd (stuur kopie besluit mee)
◉ nee

B4. Is het protocol (nog) in een ander openbaar trial register geregistreerd?

○ ja
◉ nee

B5. Naam indiener/contactpersoon voor de oordelende toetsingscommissie

B5a.

Achternaam indiener/contactpersoon	Hage
Titel en voorletters	dr. J.A.
Tussenvoegsels	van der

B5b.

Type organisatie/bedrijf	Overige Ziekenhuizen
Organisatie/bedrijf	Antoni van Leeuwenhoek Ziekenhuis
Naam organisatie/bedrijf	IKNL trialbureau
Afdeling	Chirurgie
Adres	Plesmanlaan 121
Postcode en plaats	1066 CX Amsterdam
Land	NL

B5c.

Intern adres	afdeling Heelkunde
Telefoon	020 512 9111
Fax	020 512 2554
E-mail	j.vd.hage@nki.nl

B6. Is de indiener werkzaam bij de opdrachtgever/sponsor (verrichter) van het onderzoek?

○ Ja ◉ Nee

B7. Opdrachtgever/sponsor van het onderzoek (verrichter volgens de WMO)

Type Organisatie/Bedrijf	Universitair Medisch Centrum
Organisatie/Bedrijf	Medisch Universitair Ziekenhuis Maastricht

Adres	P. Debyelaan 25
Postcode en plaats	6229 HX Maastricht
Land	NL
Telefoon	0433877477
Fax	0433875473
E-mail	m.smidt@mumc.nl

C. Sectie - Onderzoek

C1. Volledige titel van het onderzoek
C1a. In het Engels
Clinically node negative breast cancer patients undergoing breast conserving therapy: Sentinel lymph node procedure versus follow-up. A Dutch randomized controlled multicentre trial.

C1b. In het Nederlands
Patiënten met borstkanker en een klinisch negatieve okselklierstatus die een borstsparende behandeling ondergaan: Schildwachtklierprocedure versus follow-up. Een Nederlandse gerandomiseerde multicenter studie.

C2. Verkorte titel van het onderzoek/acroniem
C2a. In het Engels
BOOG 2013-08

C2b. In het Nederlands (Let op: deze korte titel wordt vermeld binnen ToetsingOnline)
BOOG 2013-08

C3. Trefwoorden (maximaal 4, plaats elk trefwoord op een aparte regel)
C3a. In het Engels
Breast neoplasms
Breast conserving therapy
Sentinel lymph node biopsy
Axilla

C3b. In het Nederlands
Borstkanker
Borstsparende behandeling
Schildwachtklierprocedure
Oksel

C4. Beschrijf het belang van het onderzoek en de beoogde toepassing van de resultaten (verwijs eventueel naar de relevante pagina's in het protocol).
Het doel van dit onderzoek is om:
1) Overbehandeling van de oksel te verminderen;
2) Het aantal korte en lange termijn complicaties te verminderen;
3) De kwaliteit van leven te verbeteren;
4) Met een even goede ziektecontrole en algehele overleving

Dit willen wij bereiken door te onderzoeken of de schildwachtklierprocedure veilig achterwege gelaten kan worden bij patiënten met borstkanker en een klinisch negatieve okselklierstatus behandeld met borstsparende therapie.

C6. Betreft het onderzoek een multicenter-onderzoek?
- ○ nee
- ◉ ja - alleen in Nederland
- ○ ja - internationaal binnen de Europese Unie
- ○ ja - internationaal ook buiten de Europese Unie

C7. Is er bij multicenter-onderzoek sprake van een coördinerend onderzoeker?
- ◉ ja namelijk
- ○ nee

Naam onderzoeker:
M.L. Smidt

C8. Wie is/zijn medisch verantwoordelijk voor de proefpersonen die deelnemen aan het onderzoek
Behandelend arts en lokale hoofdonderzoeker

C9. In welk centrum/welke centra (incl. huisartsenpraktijken) in Nederland wordt het onderzoek uitgevoerd?

Centrum	Proefpersonen	Hoofdonderzoeker	Onafhankelijk arts
Albert Schweitzer Ziekenhuis	49	M.B.E. Menke-Pluijmers	S.O. Breukink (Maastricht UMC+)
Amphia Ziekenhuis	69	E.J.T. Luiten	S.O. Breukink (Maastricht UMC+)

Antonius Ziekenhuis, Sneek	56	C.G.J.M. van Riel	S.O. Breukink (Maastricht UMC+)
Atrium Medisch Centrum	39	E.R.M. van Haaren	S.O. Breukink (Maastricht UMC+)
Bronovo Ziekenhuis	23	O.R. Guicherit	S.O. Breukink (Maastricht UMC+)
Canisius Wilhelmina Ziekenhuis	46	L.J.A. Strobbe	S.O. Breukink (Maastricht UMC+)
Catharina-ziekenhuis	46	G.A.P. Nieuwenhuijzen	S.O. Breukink (Maastricht UMC+)
Deventer Ziekenhuis	39	H. de Vries	S.O. Breukink (Maastricht UMC+)
Zorggroep De Sionsberg	15	K. Blaauwendraat	S.O. Breukink (Maastricht UMC+)
Diakonessenhuis Utrecht	39	T. van Dalen	S.O. Breukink (Maastricht UMC+)
Gelre Ziekenhuizen	37	W.B. Lastdrager	S.O. Breukink (Maastricht UMC+)
Groene Hart Ziekenhuis	31	E. Linthorst	S.O. Breukink (Maastricht UMC+)
HagaZiekenhuis	41	I. Jannink	S.O. Breukink (Maastricht UMC+)
Ikazia Ziekenhuis	27	B.R. Toorenvliet	S.O. Breukink (Maastricht UMC+)
Isala Klinieken	54	A.B. Francken	S.O. Breukink (Maastricht UMC+)
LangeLand Ziekenhuis	14	B. Jas	S.O. Breukink (Maastricht UMC+)
Laurentius Ziekenhuis	23	A.V.R.J. Bell	S.O. Breukink (Maastricht UMC+)
Academisch Ziekenhuis Maastricht	39	M.L. Smidt	S.O. Breukink (Maastricht UMC+)
Martini Ziekenhuis	43	W. Kelder	S.O. Breukink (Maastricht UMC+)
Maxima Medisch Centrum	31	K. Schenk	S.O. Breukink (Maastricht UMC+)
Meander Medisch Centrum	43	A.J. van Overbeeke	S.O. Breukink (Maastricht UMC+)
Antoni van Leeuwenhoek Ziekenhuis	93	J.A. van der Hage	S.O. Breukink (Maastricht UMC+)
Onze Lieve Vrouwe Gasthuis	28	P.J. Borgstein	S.O. Breukink (Maastricht UMC+)
Orbis Medisch Centrum	23	Y. Vissers	S.O. Breukink (Maastricht UMC+)
Universitair Medisch Centrum Sint Radboud	31	J.H.W. de Wilt	S.O. Breukink (Maastricht UMC+)
Reinier de Graaf Groep	46	M. van 't Riet	S.O. Breukink (Maastricht UMC+)
Sint Antonius Ziekenhuis	58	R. Koelemij	S.O. Breukink (Maastricht UMC+)
Sint Elisabeth Ziekenhuis	37	P. Jansen	S.O. Breukink (Maastricht UMC+)
Streekziekenhuis Koningin Beatrix	19	A. Jonk	S.O. Breukink (Maastricht UMC+)
Tergooiziekenhuizen locatie Blaricum	42	E.J.C. Vriens	S.O. Breukink (Maastricht UMC+)
Universitair Medisch Centrum Groningen	17	J. de Vries	S.O. Breukink (Maastricht UMC+)
Universitair Medisch Centrum Utrecht	23	C. van der Pol	S.O. Breukink (Maastricht UMC+)
Viecuri Medisch Centrum voor Noord-Limburg	39	C.L.H. van Berlo	S.O. Breukink (Maastricht UMC+)
Westfries Gasthuis	27	J.W.D. de Waard	S.O. Breukink (Maastricht UMC+)
Waterland Ziekenhuis	22	L. de Widt	S.O. Breukink (Maastricht UMC+)
Zaans Medisch Centrum	15	S. Muller	S.O. Breukink (Maastricht UMC+)
Ziekenhuisvoorzieningen Gelderse Vallei	32	W.K. de Roos	S.O. Breukink (Maastricht UMC+)
Ziekenhuisgroep Twente	31	D. de Leeuw	S.O. Breukink (Maastricht UMC+)
Rivierenland Ziekenhuis	23	M.A.J. de Roos	S.O. Breukink (Maastricht UMC+)

Medisch Centrum Alkmaar	54	W.H. Schreurs	S.O. Breukink (Maastricht UMC+)
Medisch Centrum Haaglanden	31	A. Marinelli	S.O. Breukink (Maastricht UMC+)
Medisch Spectrum Twente	46	P. Steenvoorde	S.O. Breukink (Maastricht UMC+)
Rijnstate Ziekenhuis	62	R. van Eekeren	S.O. Breukink (Maastricht UMC+)
Flevoziekenhuis	50	S. van der Meij	S.O. Breukink (Maastricht UMC+)
Zuwehofpoort	15	R.C. van Doorn	S.O. Breukink (Maastricht UMC+)
Jeroen Bosch Ziekenhuis	20	M. Bessem	B. Biesma (Jeroen Bosch ziekenhuis)
Alrijne ziekenhuis	50	P. Neijenhuis	S.O. Breukink (Maastricht UMC+)
Kennemer Gasthuis	30	B. Kortmann	S.O. Breukink (Maastricht UMC+)

C10. Betreft het onderzoek met:

- [x] mensen
- [ ] geslachtscellen
- [ ] (rest-)embryo's
- [ ] foetussen in utero

C11. Beoogd totaal aantal proefpersonen/(rest)embryo's/foetussen in utero:
C11a. In Nederland
1644

C13. Onderzoeksgebied

- [ ] etiologie
- [ ] organisatorisch/zorgonderzoek
- [ ] diagnostiek
- [ ] preventie
- [x] therapie
- [ ] veiligheid
- [ ] werkzaamheid
- [ ] farmacokinetiek
- [ ] farmacodynamiek
- [ ] bio-equivalentie
- [ ] dosis-respons
- [ ] farmacogenomics
- [ ] farmaco-economie
- [ ] anders

C14. Type onderzoek

- ( ) observationeel onderzoek zonder invasieve metingen
- ( ) observationeel onderzoek met invasieve metingen
- (•) interventie-onderzoek

C15. In welke fase kan het onderzoek worden ingedeeld?

- ( ) fase I (a)
- ( ) fase II (b)
- (•) fase III (c)
- ( ) fase IV (d)
- ( ) overige onderzoeken waarbij geneesmiddelen worden toegepast (e)
- ( ) niet van toepassing

C17. Is er sprake van een andere interventie dan met geneesmiddelen zoals bedoeld in de Wet medisch-wetenschappelijk onderzoek met mensen (WMO) (zie toelichting)?

- (•) Ja
- ( ) Nee

C17a. ☐ Medisch hulpmiddel, namelijk
C17b. ☐ Operatie, namelijk
C17c. ☐ Psychosociale interventie, namelijk
C17d. ☐ Voeding(stoffen), namelijk
C17e. ☐ Bewegingstherapie, namelijk
C17f. ☐ interventie met radioactieve straling, namelijk
C17g. ☐ Blootstellingsonderzoek (bijv pesticidenonderzoek), namelijk
C17h.
C17i. ☑ andere interventie, namelijk

In het Engels: No sentinel lymph node procedure
In het Nederlands: Géén schildwachtklierprocedure

C18. Worden de onderzoeksproducten voor deze studie door de verrichter gratis verstrekt?

○ ja
○ nee
○ gedeeltelijk namelijk
◉ niet van toepassing

C19. Is/zijn er (een) controlegroep (en)?

◉ ja
○ nee

☐ ja, geneesmiddel
☐ ja, placebo
☐ ja, geen interventie
☑ ja, anders

namelijk De standaard behandeling: schildwachtklierprocedure

C20. Betreft het een gerandomiseerd onderzoek?

◉ ja
○ nee

namelijk

☑ open
☐ enkelblind
☐ dubbelblind
☐ parallel
☐ cross-over
☐ anders namelijk

C21. Op welke klasse(n) van aandoeningen heeft het onderzoek betrekking (maximaal 3)

☐ hartaandoeningen

☐ congenitale, familiaire en genetische aandoeningen
☐ bloed- en lymfestelsel aandoeningen
☐ zenuwstelsel aandoeningen
☐ oogaandoeningen
☐ evenwichtsorgaan- en ooraandoeningen
☐ ademhalingsstelsel-, thorax- en mediastinumaandoeningen
☐ maagdarmstelselaandoeningen
☐ nier- en urinewegaandoeningen
☐ huid- en onderhuidaandoeningen
☐ skeletspierstelsel- en bindweefselaandoeningen
☐ endocriene aandoeningen
☐ voedingsstoornissen en metabole ziekten

- ☐ infecties en parasitaire aandoeningen
- ☐ letsels, intoxicaties en verrichtingscomplicaties
- ☑ neoplasmata, benigne, maligne en niet-gespecificeerd (incl cysten en poliepen)
  borstneoplasmata maligne en niet-gespecificeerd (incl. tepel)
- ☑ chirurgische en medische verrichtingen
  borst therapeutische verrichtingen
- ☐ bloedvataandoeningen
- ☐ algemene aandoeningen en aandoeningen op de plek van toediening
- ☐ zwangerschap, perinatale periode en puerperium
- ☐ sociale omstandigheden
- ☐ immuunsysteemaandoeningen
- ☐ lever- en galaandoeningen
- ☐ voortplantingsstelsel- en borstaandoeningen
- ☐ psychische stoornissen
- ☐ overig, namelijk

C22.Geef twee synoniemen voor de aandoening die bestudeerd wordt, waarvan tenminste één lekenterm.

In het Engels:	breast cancer, sentinel lymph nodes, sentinel lymph node metastases
In het Nederlands:	borstkanker, schildwachtklieren, schildwachtkliermetastasen

C23. Beoogde start- en einddatum van het onderzoek

C23a. Start Datum (dd-mm-jjjj)	01-01-2015
C23b. Eind Datum (dd-mm-jjjj)	01-01-2027

C24. Zijn patiënten betrokken bij het ontwikkelen van het onderzoek? (Voor patiënten lees ook: patiëntenorganisaties, naasten of nabestaanden van patiënten en gezonde vrijwilligers)?

C30. Wordt de ervaring van proefpersonen na deelname aan het onderzoek geëvalueerd?

D. Sectie - Proefpersonen

D1. Is er een proefpersonenverzekering conform de WMO-eisen afgesloten of wordt aan de oordelende toetsingscommissie ontheffing gevraagd?

- ◉ proefpersonenverzekering is afgesloten bij verzekeringsmaatschappij
- ○ ontheffing van de verzekering wordt gevraagd
- ○ niet van toepassing - onderzoek valt onder de Embryowet en niet onder de WMO

Verzekeringsmaatschappij	Marketform (onderdeel van Lloyd's)

D2. Gezonde proefpersonen en/of patiënten

- ☐ Gezonde proefpersonen
- ☑ Patiënten

Aantal 1644

D4. Voornaamste inclusiecriteria

D4a. In het Engels

- Female
- Aged 18 years or older
- Pathologically confirmed invasive unilateral breast carcinoma
- A clinical T1-2 tumour (≤ 5cm)
- Will be treated with lumpectomy followed by whole breast radiotherapy
- Clinically node negative status: no signs of axillary lymph node metastases at physical examination and preoperative axillary ultrasound (or negative cyto-/histopathology)
- Written informed consent

D4b. In het Nederlands

- Vrouw
- Leeftijd 18 jaar of ouder
- Pathologisch bewezen invasief unilaterale borstkanker
- Klinisch T1-2 tumor (≤ 5cm)
- Zal worden behandeld met lumpectomie gevolgd door gehele borstbestraling
- Klinisch negatieve okselklierstatus: negatief lichamelijk onderzoek en echografie van de oksel (of negatieve cyto-/histologie)
- Schriftelijke informed consent

D5. Voornaamste exclusiecriteria

D5a. In het Engels

- Clinically node positive pre-operative
- Bilateral breast cancer
- Evidence of metastatic disease
- History of invasive breast cancer
- Previous treatment of the ipsilateral axilla with surgery or radiotherapy, except surgery for hidradenitis suppurativa or for other superficially located skin lesions, such as naevi
- Pregnant or nursing
- Other prior malignancies within the past 5 years, except successfully treated malignancies that occurred more than five years before randomization, and except successfully treated basal cell and squamous cell skin cancer, carcinoma in situ of the cervix or carcinoma in situ of the ipsilateral or contralateral breast
- Unable or unwilling to give informed consent

D5b. In het Nederlands

- Klinisch positieve okselklierstatus (preoperatief)
- Bilaterale borstkanker
- Primair (afstands-)gemetastaseerde borstkanker
- Invasieve borstkanker in de voorgeschiedenis
- Chirurgie of radiotherapie van ipsilaterale oksel in de voorgeschiedenis, behalve chirurgie voor hidradenitis suppurativa of andere oppervlakkig gelegen huidlaesies, zoals naevi
- Zwanger of lacterend
- Andere maligniteit in de afgelopen 5 jaar, behalve succesvol behandelde maligniteit die meer dan vijf jaar voor randomisatie optrad, en behalve succesvol behandelde huidkanker van type basaalcel of plaveiselcel, carcinoma in situ van de cervix, carcinoma in situ van de ipsilaterale of contralaterale mamma
- Niet in staat of niet bereid tot informed consent

D6. Bij welke categorie proefpersonen wordt het onderzoek uitgevoerd (meerdere antwoorden mogelijk)

☑ 18 jaar of ouder en wilsbekwaam
☐ 18 jaar of ouder en wilsonbekwaam
☐ 12 t/m 17 jaar en in staat tot het geven van geïnformeerde toestemming
☐ 12 t/m 17 jaar en niet in staat tot het geven van geïnformeerde toestemming (wilsonbekwaam)
☐ jonger dan 12 jaar

D10. Verkeren (sommige) proefpersonen in een afhankelijkheidssituatie ten opzichte van de onderzoeker of degene die de deelnemers werft? (lees de toelichting voor voorbeelden wanneer er sprake kan zijn van een afhankelijkheidssituatie)

◉ ja
○ nee

D10a. Zo ja, waardoor?

Soms is de lokale onderzoeker ook de behandelend arts die de patiënt vraagt om deel te nemen aan de studie.

D10b. Waarom wordt het onderzoek juist met deze proefpersonen uitgevoerd en hoe worden de belangen van de proefpersonen gewaarborgd?

Het onderzoek moet met proefpersonen worden uitgevoerd die voldoen aan de beschreven in/exclusie criteria zodat de resultaten van de studie uiteindelijk ook naar de kliniek vertaald kunnen worden binnen deze patiëntenpopulatie. De belangen van de proefpersonen worden gewaarborgd door de geldende medisch ethische normen en richtlijn zoals vermeld in het protocol (o.a. onafhankelijk arts, uitgebreide patiëntinformatiefolder).

D11. Waaruit bestaat de vergoeding voor de proefpersonen?

☑ geen vergoeding
☐ reiskosten
☐ financiële vergoeding (in Euro's)
☐ andere vergoeding

D12. Is deze vergoeding afhankelijk van bepaalde voorwaarden, bijvoorbeeld het voltooien van (een deel van) het onderzoek?

○ ja (motiveer)
○ nee
◉ niet van toepassing

E. Sectie - Voor- en nadelen

E1. Wordt er bij dit onderzoek een rechtstreeks therapeutisch effect beoogd bij de proefpersonen / patiënten?

◉ ja (therapeutisch onderzoek)
○ nee (niet-therapeutisch onderzoek)

E1a. Zo ja, geef kort aan waaruit dit therapeutisch effect bestaat.

Het therapeutische effect bestaat uit:
- Het verlagen van het risico op okselbehandeling-gerelateerde korte en lange termijn complicaties;
- Het verbeteren van de kwaliteit van leven;
- Met een even goede ziektecontrole en algehele overleving.

E2. Waaruit bestaat de belasting van het onderzoek (en een eventueel daaraan voorafgaande keuring) voor de proefpersonen?

Tijdbeslag	per bezoek	20 min per keer voor invullen kwaliteit van leven vragenlijst
	totaal	20 min x 6 = 120 min
	totale duur van de studie voor de individuele proefpersoon	Tav de behandeling minder belasting dan normaal voor de studie-arm (geen schildwachtklierprocedure). Voor beide armen is extra het invullen van de kwaliteit van leven vragenlijst prerandomisatie en postrandomisatie op 5 momenten.

E3. Worden de proefpersonen in verband met het onderzoek in het ziekenhuis opgenomen of wordt een opname verlengd?

- ○ ja - het verblijf in het ziekenhuis/instituut wordt in verband met het onderzoek verlengd
- ○ ja - ze worden voor het onderzoek in het ziekenhuis/instituut opgenomen
- ◉ nee

E4. Beschrijf in hoeverre proefpersonen worden onderworpen aan handelingen dan wel een gedragswijze krijgen opgelegd, zoals vragenlijst, interviews, lichamelijk/psychologisch onderzoek, ontzegging, dieet (voor invasieve ingrepen: zie vraag E6)

In totaal zullen 700 proefpersonen (350 in de experimentele en 350 in de controlegroep) deelnemen aan het kwaliteit van leven onderzoek, bestaande uit een vragenlijst voor de randomisatie en op 5 tijdstippen na de randomisatie (na een half jaar, één, twee, vijf en 10 jaar).

E5. Worden de proefpersonen getest op bepaalde aandoeningen/condities?

- ○ ja (motiveer)
- ◉ nee

E6. Welke extra (invasieve) ingrepen (anders dan bij de standaard behandeling) moeten de proefpersonen in het kader van het onderzoek ondergaan:

- ☑ Niet van toepassing

E9. Geef aan welke risico's er voor proefpersonen zijn verbonden aan deelname aan het onderzoek.

Hoewel de hypothese luidt dat het achterwege laten van de schildwachtklierprocedure geen nadelige invloed heeft op de lokale controle van de ziekte en de overlevingskans, bestaat er toch een kans dat proefpersonen in de experimentele arm een hoger risico hebben op een recidief in de oksel (regionaal recidief) en daardoor tijdens follow-up alsnog een vorm van okselklierbehandeling moeten ondergaan. Uit onderzoek blijkt dat het uitgesteld uitvoeren van okselklierbehandeling (bijvoorbeeld met okselklierdissectie) géén nadelige gevolgen heeft voor de overleving.

E9a. Geef op grond van uw eigen afweging aan waarom het uitvoeren van het onderzoek, in het licht van de belasting en/of risico's die voor proefpersonen aan deelname verbonden zijn, gerechtvaardigd is?

Het mogelijk hogere risico op een recidief in de oksel (regionaal recidief) weegt op tegen de lagere kans op ernstige okselklierbehandeling-gerelateerde morbiditeit (lymfoedeem, bewegingsbeperking in de schouder, arm- en schouderpijn, verminderd gevoel of zenuwpijn) en de verbetering van kwaliteit van leven.

E10. Indien het onderzoek bij minderjarige en/of wilsonbekwame proefpersonen wordt uitgevoerd en geen direct therapeutisch effect wordt beoogd: waarom kunnen belasting en risico's als minimaal worden beschouwd? (Verwijs eventueel naar de relevante pagina's in het protocol.

- ☑ niet van toepassing

E11. Kan de eventuele therapie na beëindiging van het onderzoek worden voortgezet?

- ○ ja (motiveer)
- ○ nee (motiveer)
- ◉ niet van toepassing

E12. Heeft deelname aan het onderzoek voor de proefpersoon tot gevolg dat van de standaardbehandeling of -diagnostiek kan worden afgeweken of deze kan worden uitgesteld?

- ◉ ja
- ○ nee
- ○ niet van toepassing

E12a. Zo ja, waaruit bestaat de afwijking of het uitstel en waarom is afwijking/uitstel

Proefpersonen in de controlegroep ondergaan de standaard behandeling, namelijk schildwachtklierprocedure volgens de Nederlandse borstkankerrichtlijn. Proefpersonen in de experimentele arm ondergaan primair géén schildwachtklierprocedure. Beide groepen ondergaan dezelfde follow-up middels lichamelijk onderzoek en aanvullend onderzoek op indicatie. Indien een proefpersoon zich in de follow-up periode presenteert met lymfekliermetastasen, kan alsnog besloten worden om een vorm van okselklierbehandeling uit te voeren. Deze beslissing wordt gemaakt tijdens het multidisciplinair overleg in het desbetreffende centrum waar de patiënt onder behandeling staat.

F. Sectie - Informatie en privacy

F1. Hoe worden de proefpersonen geworven en door wie (onderzoeker, behandelend arts, andere persoon) wordt de proefpersoon/wettelijke vertegenwoordiger geïnformeerd en om toestemming gevraagd?

Op het moment in de behandeling van borstkanker dat de patiënt voldoet aan de in/exclusiecriteria zal de behandeld arts informatie geven over de mogelijkheid tot deelname aan deze studie. Een schriftelijke patiëntinformatiebrief zal worden meegegeven aan de patiënt. De patiënt krijgt ruim de tijd (ten minste één week) om deze informatie thuis na te lezen en een beslissing te nemen. De patiënt kan haar interesse in deelname kenbaar maken aan haar behandelend arts bij een volgend polibezoek. De behandelend arts zal haar dan om toestemming vragen voor deelname aan de studie.

F2. Hoeveel bedenktijd krijgen de proefpersonen/wettelijke vertegenwoordigers om te beslissen over deelname?

Zo lang als nodig, met ten minste één week

F3. Wordt de huisarts, behandelend specialist en/of apotheker van de proefpersoon geïnformeerd over diens deelname aan het onderzoek?

- (●) ja (de proefpersoon dient hiervoor toestemming te geven)
- ( ) nee

F4. Worden persoonsgegevens gecodeerd?

- (●) ja
- ( ) nee

F4a. Zo ja, hoe is deze codering opgebouwd?

Alle proefpersonen krijgen een uniek studienummer. Het unieke studienummer betreft een opvolgnummer zonder verdere specifieke codering voor bijvoorbeeld centrum.

F4b. Wie heeft toegang tot de sleutel van deze code?

De behandelend arts en de onderzoeker

F4c. Wie hebben toegang tot de brondocumenten en eventuele andere tot de persoon herleidbare gegevens?

De lokaal datamanagers en de onderzoeker

F5. Hoe wordt het lichaamsmateriaal gedurende het onderzoek bewaard?

- ( ) in tot de proefpersoon herleidbare vorm (gecodeerd)
- ( ) in niet tot de proefpersoon herleidbare vorm (volledig geanonimiseerd)
- (●) niet van toepassing

F6. Wordt afgenomen lichaamsmateriaal na afloop van het onderzoek vernietigd?

- ( ) ja
- ( ) nee (motiveer)
- (●) niet van toepassing

F7. Kunnen proefpersonen na afloop van het onderzoek opnieuw benaderd worden (bijvoorbeeld voor nader onderzoek of follow-up)?

- ( ) ja
- (●) nee

G. Sectie - Financieel

G1. Door welke geldstroom wordt het onderzoek gefinancierd?

- [ ] eerste geldstroom (Geld van Ministerie van OC&W aan universiteiten)
- [ ] tweede geldstroom (NWO of KNAW), namelijk
- [x] derde geldstroom (anders dan 1e of 2e geldstroom, zoals collectebusfondsen, Europese Unie, vakministeries of bedrijven), namelijk

KWF Kankerbestrijding

G2. Wordt het onderzoek (mede) gefinancierd door de industrie/bedrijven?

- ○ ja - door de industrie/bedrijf zoals is opgegeven bij vraag B6/B7 (opdrachtgever van het onderzoek)
- ○ ja - (ook) door andere industrie/bedrijven dan de opdrachtgever
- ◉ nee

G3. Wat is de hoogte van de vergoeding die de arts/onderzoeker cq onderzoeksafdeling/maatschap ontvangt voor de uitvoering van het onderzoek?

- ○ Per patiënt of proefpersoon
- ◉ Per deelnemend centrum

bedrag afgerond in hele euro's: € 100,00

G3a. Hoe is de vergoeding opgebouwd?

De vergoeding betreft een financiële compensatie van € 100 excl. BTW als opstartvergoeding voor ieder deelnemend centrum.

G4. Heeft/hebben de onderzoeker(s) gedurende de afgelopen vijf jaar op een of andere wijze een persoonlijke financiële relatie (gehad) met de verrichter/sponsor van het huidige onderzoek?

- ○ ja (licht toe)
- ◉ nee

I. Sectie - Indiening en beoordeling

I1. Sla het formulier eerst op en selecteer vervolgens een METC of de CCMO

Commissie
METC NedMec

J. Sectie - Aanvullende opmerkingen

Aanvullende opmerkingen

K. Sectie - Samenvatting

Achtergrond van het onderzoek:

De schildwachtklierprocedure is de standaard methode voor lymfeklierstadiëring in borstkankerpatiënten met een klinisch negatieve okselklierstatus. In de algemene bevolking bevat de schildwachtklier in ongeveer 25% een uitzaaiing. Dit percentage is lager in patiënten met een kleine borsttumor en in borstsparend behandelde patiënten. De ACOSOG-Z0011 studie en de IBCSG 23-01 studie hebben recent aangetoond dat een completerende okselklierdissectie veilig achterwege gelaten kan worden bij borstsparend en met systemische therapie behandelde patiënten met een beperkt aantal schildwachtkliermetastasen. Dit roept de vraag op of de schildwachtklierprocedure nog wel noodzakelijk is voor borstsparend behandelde patiënten met een klinisch negatieve okselklierstatus. De klinisch negatieve okselklierstatus van de patiënten in de beschreven studies werd gesteld op basis van lichamelijk onderzoek. Het toevoegen van een preoperatieve echografie van de oksel zal zorgen voor een betere voorselectie van patiënten zonder lymfekliermetastasen, ten opzichte van lichamelijk onderzoek alleen. Daarbij sluit een negatieve echografie uitgevoerd in Nederland bijna alle patiënten uit met uitgebreide lymfekliermetastasen (≥4 lymfekliermetastasen). Bovendien omvat gehele borstbestraling na de borstsparende operatie in het algemeen het lagere deel van de oksel en behandelt daarmee eventueel achtergebleven uitzaaiingen. Hoewel de schildwachtklierprocedure minder invasief is vergeleken met een okselklierdissectie, komen complicaties nog steeds voor. Zo lijdt 6% van de patiënten die alleen een schildwachtklierprocedure ondergingen aan ernstige lymfoedeem na slechts 3 jaar. Deze studie gaat onderzoeken of de schildwachtklierprocedure veilig achterwege gelaten kan worden bij borstsparend behandelde patiënten, met een negatieve echografie van de oksel.

Doel van het onderzoek:

Het doel van deze studie is het verminderen van overbehandeling van de oksel bij patiënten met borstkanker en een klinisch negatieve okselklierstatus die behandeld worden met borstsparende therapie, door het achterwege laten van de schildwachtklierprocedure, om zodoende de kans op korte en lange termijn complicaties te verminderen en te zorgen voor een verbetering van de kwaliteit van leven met een even goede ziektecontrole en algehele overleving.

Onderzoeksopzet:

Het betreft een gerandomiseerde non-inferiority multicenter onderzoek. Patienten die voldoen aan de in- en exclusiecriteria worden na toestemming middels loting behandeld volgens één van de volgende behandelmethoden:

- Behandelmethode A (controle groep): schildwachtklierprocedure volgens de Nederlandse borstkankerrichtlijn.
- Behandelmethode B (studie groep): geen verdere okselklierstadiëring.

Stratificatiefactoren: leeftijd (≤50, 50≤75, >75 jaar), oestrogeen receptor status (positief vs. negatief), HER2neu status (geamplificeerd vs. niet geamplificeerd), klinisch tumor stadium (<3 cm vs. ≥3 cm), tumorgraad volgens gemodificeerde Bloom-Richardson (graad I-II vs. graad III), is of zal worden behandeld met primaire systemische therapie (ja/nee), participerend centrum.
Overige behandelingen (o.a. hormonale therapie, chemotherapie) worden op indicatie gegeven volgens de Nederlandse borstkankerrichtlijn.
Tijdens de follow-up periode van 10 jaar wordt de oksel nauwkeurig onderzocht middels lichamelijk onderzoek en echografie op indicatie. Indien sprake is van een cyto-/histologisch bewezen lymfekliermetastase tijdens follow-up, dan kan in het multidisciplinair overleg van de desbetreffende patiënt worden besloten tot het uitvoeren van een vorm van okselklierbehandeling.
Gevalideerde vragenlijsten worden verstrekt voor het onderzoeken van de invloed op het aantal complicaties en kwaliteit van leven (Lymph-ICF, QLQ-C30, QLQ-BR23) en om te meten of angst en persoonlijkheidstrekken invloed hebben op de uitkomst van

kwaliteit van leven (STAI-trait, NEO-FFI). Deze vragenlijsten worden verstrekt aan 700 van de 1644 patiënten (350 per arm), éénmaal voor de randomisatie en vijfmaal na de randomisatie (6 maanden, 1 jaar, 2 jaar, 5 jaar en 10 jaar). Vragenlijsten met betrekking to kosten-effectiviteit worden éénmaal voor de randomisatie en viermaal na de randomisatie (6 maanden, 1,2 en 3 jaar) afgenomen.

Onderzoekspopulatie:

Vrouwen van 18 jaar of ouder met pathologisch bewezen invasief mammacarcinoom die borstsparende therapie ondergaan.
cT1-2: primaire tumor is bij klinisch onderzoek kleiner dan 5 cm.
cN0: bij lichamelijk onderzoek en echografie van de oksel worden geen aanwijzingen voor lymfekliermetastasen gevonden of de cytologie/histologie na punctie/biopt van een lymfeklier is negatief.
Neoadjuvante systemische therapie is toegestaan indien de patiënt voor de start van de neoadjuvante systemische therapie een klinische T1-2N0 status heeft en waarvoor borstsparende therapie op dat moment al geschikt is.
De patiënt heeft informed consent persoonlijk ondertekend.

Interventie (indien van toepassing):

De controlegroep ondergaat wél de schildwachtklierprocedure volgens de Nederlands borstkankerrichtlijn. De experimentele arm ondergaan géén schildwachtklierprocedure.

Primaire onderzoeksvariabelen/uitkomstmaten:

Primaire uitkomstmaat:
- Regionaal recidief percentage

Secundaire onderzoeksvariabelen/uitkomstmaten (indien van toepassing):
Secundaire uitkomstmaten:
- Distant-disease free survival
- Overall survival
- Later uitgevoerde okselbehandeling
- Lokaal recidief percentage
- Overig-regionaal recidief percentage
- Contralaterale borstkanker
- Percentage (neo)adjuvante systemische therapie
- Kwaliteit van leven en okselbehandeling-gerelateerde morbiditeit
- Kosten-effectiviteit

Omschrijving en inschatting van belasting en risico (indien van toepassing):

Wat betreft belasting is er in deze studie géén sprake van extra diagnostiek of interventies (juist minder) en hoeft de patiënt niet vaker dan normaal voor controle naar het ziekenhuis. De eventuele extra belasting voor de patiënt zal alleen bestaan uit het invullen van kwaliteit van leven vragenlijsten (bij 700 van 1644 patiënten) éénmaal voor de randomisatie en vijfmaal na randomisatie (6 maanden, 1 jaar, 2 jaar, 5 jaar en 10 jaar). De hypothese luidt dat het achterwege laten van de schildwachtklierprocedure even veilig is als het wél uitvoeren van deze procedure. Desondanks is er een risico dat in de experimentele arm meer regionale recidieven optreden en dat bij patiënten op een later tijdstip alsnog een vorm van okselklierbehandeling moet worden uitgevoerd. Daartegenover staat dat er in de experimentele arm door het niet uitvoeren van de schildwachtklierprocedure minder kans is op morbiditeit, minder operaties en opnames in het ziekenhuis en een betere kwaliteit van leven. Patiënten zullen op geen enkele manier vertraging ondervinden als gevolg van deelname aan de studie.

K2.Engelse Samenvatting

Background of the study:
The NSABP B-04 trial revealed that omitting primary axillary treatment of occult positive lymph nodes in clinically node negative breast cancer patients does not affect survival, even after 25 years of follow-up and without the use of adjuvant systemic or radiation therapy. A delayed axillary dissection in case lymph nodes become clinically positive does not affect survival and prevents axillary overtreatment in the majority of patients. The ACOSOG Z0011 and IBCSG 23-01 trials revealed that completion axillary dissection can be safely omitted in clinically node negative patients with metastatic sentinel nodes. Patients randomized for watchful waiting were likely to have residual nodal disease (13-27%) that was not surgically removed. Nevertheless, survival rates were not affected and regional recurrence rates low. Use of axillary ultrasound will improve the preoperative selection of node negative patients, as it selects patients with a more favourable tumour load and accurately excludes advanced nodal disease (≥4 metastatic nodes). Biology and adjuvant systemic and radiation therapy are all factors that most likely diminish the risk that possible metastases left in situ develop into clinically detectable lymph nodes. Patients treated with breast conserving therapy are significantly more often diagnosed with pathologic node negative disease or with micrometastatic disease, compared to patients treated with mastectomy. The risk for occult (macro)metastases in cT1-2N0 breast cancer patients treated with breast conserving therapy is low: about 88% will be node negative and about 95% of node positive patients will have no lymph node metastases beyond the sentinel node. Reflected by the low regional recurrence rate after a (false) negative sentinel node, only a small amount of patients (0.8%) with node positive disease is expected to develop clinically detectable nodal disease.

Objective of the study:
The objective of this study is to decrease the number of breast cancer patients receiving overtreatment of the axilla, in order to positively influence the axillary morbidity rate and quality of life. Therefore, we investigate whether the sentinel lymph node procedure can be safely omitted in clinically node negative breast cancer patients undergoing breast conserving therapy, in terms of non-inferior regional control, distant-disease free and overall survival. All objectives are measured during a follow-up of 10 years.

Study design:
A randomized controlled non-inferiority multicenter study. Patients who meet the in- and exclusioncriteria will be randomized after

informed consent for one of the two treatment methods:
- Arm A (control arm): lumpectomy with sentinel lymph node procedure, followed by radiotherapy of the breast with or without completion axillary treatment according to the Dutch breast cancer guideline.
- Arm B (experimental arm): lumpectomy without further axillary staging, followed by radiotherapy of the breast.
Stratification: age (≤50, 50≤75, >75 years old), oestrogen receptor status (positive vs. negative), HER2neu status (amplified vs. not amplified), clinical tumour stage prior to any treatment (<3 cm vs. ≥3 cm), grading (grade I-II vs. III - according to Bloom-Richardson grading system), is or will be treated with primary systemic therapy (yes/no) and participating centre.
Adjuvant systemic treatment is administered if indicated according to the Dutch breast cancer guideline.
Primary systemic therapy is allowed if the patient has a clinical T1-2N0 status (initial stage) that is amenable to lumpectomy pre-systemic therapy.
Yearly follow-up with physical examination of the axilla for 10 years. Axillary ultrasound (+/- tissue sampling) on indication. Staging for distant metastases and/or a delayed axillary lymph node dissection if indicated by the multidisciplinary team. Validated questionnaires are used to assess axillary morbidity rate and quality of life (Lymph-ICF, QLQ-C30, QLQ-BR23) and to measure if anxiety and personality traits influence the outcome of quality of life (STAI-trait, NEO-FFI) in 700 of 878 patients. These questionnaires are provided before randomization and sequentially post randomization at 6 months, 1, 2, 5 and 10 years. Cost-effectiveness questionnaires are provided before randomization and sequentially post randomization at 6 months, 1, 2 and 3 years.

Study population:
Women aged 18 years or older with pathologically confirmed invasive breast carcinoma about to undergo breast conserving surgery followed by radiotherapy of the breast.
cT1-2: primary tumour is smaller than 5 cm.
cN0: no signs of axillary lymph node metastases at physical examination and axillary ultrasound (+/- tissue sampling).
Primary systemic treatment is allowed.
Patient is able to and signed the informed consent form.

Intervention (if applicable):
Patients in the control arm will be treated with the sentinel lymph node procedure according to the Dutch breast cancer guideline.
Patients in the experimental arm will not be treated with the sentinel lymph node procedure.

Primary study parameters/outcome of the study:
Primary endpoint:
- Regional recurrence rate

Secundary study parameters/outcome of the study (if applicable):
Secondary endpoints:
- Distant-disease free survival
- Overall survival
- Delayed axillary treatment
- Local recurrence rate
- Other-regional recurrence rate
- Contralateral breast cancer rate
- Percentage difference in the administration of (neo)adjuvant systemic therapy
- Quality of life and axillary morbidity rate
- Cost-effectiveness

Nature and extent of the burden and risks associated with participation, benefit and group relatedness (if applicable):
There is no extra burden for participating patients as regard to additional diagnostic tests/interventions and follow-up. Any additional burden for the patient will only consist of completing quality of life questionnaires in 700 of 1644 study patients. These questionnaires are provided before randomization and sequentially post randomization at 6 months, 1, 2, 5 and 10 years.
We hypothesize that omitting the sentinel lymph node procedure is not inferior to the standard treatment. Nevertheless, possible disadvantages are a worse regional recurrence rate, and delayed axillary treatment if indicated. Patients in the experimental arm have the possible advantages of less morbidity, fewer operations and hospitalizations and an improved quality of life, compared to patients treated with the sentinel lymph node procedure.
No patient will encounter any delay in their treatment as a result of inclusion.

ONDERTEKENING

De verrichter en indiener verklaren hierbij:
a. het formulier (en samenvatting) volledig en naar waarheid te hebben ingevuld;
b. de antwoorden op de vragen uit het ABR-formulier niet in strijd zijn met het bijbehorende onderzoeksdossier en onderzoekscontract

Naar waarheid getekend, door de verrichter (=opdrachtgever) | door de indiener

datum .... | datum ....

Handtekening		Handtekening	
naam	U.P. Neumann	naam	J.A. van der Hage
functie	Hoofd afdeling Chirurgie, MUMC+	functie	Chirurg, NKI-AVL

Sluiten Print

Sluiten Print

Formulier voor medisch-ethische beoordeling en registratie

ABR-formulier, versie december 2015

Onderzoeksdossiernummer

ABR Nummer	**49315**	Status	**Beoordeeld - Positief**
Versie	**08**	Status per	**30-06-2017**
Jaar	**17**		
Dossiernummer	**NL49315.031.14**		
Reden voor	**Toevoegen extra meetmoment t=3 jaar in PIF**		

A. Sectie - Openbaar maken gegevens medisch wetenschappelijk onderzoek

A1. Het CCMO-register is een voor ieder toegankelijk openbaar trial register. De antwoorden op de vragen gemarkeerd met een wereldbol en de samenvatting bij dit formulier worden openbaar gemaakt in het CCMO register.

B. Sectie - Administratief

B1. Betreft het onderzoek met geneesmiddelen (inclusief gentherapie, somatische celtherapie, vaccinonderzoek, GGO's, zie verdere toelichting) als bedoeld in de Wet medisch-wetenschappelijk onderzoek met mensen (WMO)?

- ○ ja
- ◉ nee

B2. Houdt het onderzoek verband met een eerder door een erkende METC of door de CCMO beoordeelde studie of is het onderzoek reeds eerder bij een erkende METC ter beoordeling voorgelegd?

- ○ ja, het onderzoek houdt verband met – of is het vervolg op – een eerder beoordeelde studie
- ○ ja, het onderzoek is eerder ter beoordeling aan een erkende METC of de CCMO voorgelegd (stuur kopie besluit mee)
- ◉ nee

B4. Is het protocol (nog) in een ander openbaar trial register geregistreerd?

- ○ ja
- ◉ nee

B5. Naam indiener/contactpersoon voor de oordelende toetsingscommissie

B5a.

Achternaam indiener/contactpersoon	Hage
Titel en voorletters	dr. J.A.
Tussenvoegsels	van der

B5b.

Type organisatie/bedrijf	Overige Ziekenhuizen
Organisatie/bedrijf	Antoni van Leeuwenhoek Ziekenhuis
Naam organisatie/bedrijf	IKNL trialbureau
Afdeling	Chirurgie
Adres	Plesmanlaan 121
Postcode en plaats	1066 CX Amsterdam
Land	NL

B5c.

Intern adres	afdeling Heelkunde
Telefoon	020 512 9111
Fax	020 512 2554
E-mail	j.vd.hage@nki.nl

B6. Is de indiener werkzaam bij de opdrachtgever/sponsor (verrichter) van het onderzoek?

○ Ja ◉ Nee

B7. Opdrachtgever/sponsor van het onderzoek (verrichter volgens de WMO)

Type Organisatie/Bedrijf	Universitair Medisch Centrum
Organisatie/Bedrijf	Medisch Universitair Ziekenhuis Maastricht

Adres P. Debyelaan 25
Postcode en plaats 6229 HX Maastricht
Land NL
Telefoon 0433877477
Fax 0433875473
E-mail m.smidt@mumc.nl

C. Sectie - Onderzoek

C1. Volledige titel van het onderzoek
C1a. In het Engels
Clinically node negative breast cancer patients undergoing breast conserving therapy: Sentinel lymph node procedure versus follow-up. A Dutch randomized controlled multicentre trial.

C1b. In het Nederlands
Patiënten met borstkanker en een klinisch negatieve okselklierstatus die een borstsparende behandeling ondergaan: Schildwachtklierprocedure versus follow-up. Een Nederlandse gerandomiseerde multicenter studie.

C2. Verkorte titel van het onderzoek/acroniem
C2a. In het Engels
BOOG 2013-08

C2b. In het Nederlands (Let op: deze korte titel wordt vermeld binnen ToetsingOnline)
BOOG 2013-08

C3. Trefwoorden (maximaal 4, plaats elk trefwoord op een aparte regel)
C3a. In het Engels
Breast neoplasms
Breast conserving therapy
Sentinel lymph node biopsy
Axilla

C3b. In het Nederlands
Borstkanker
Borstsparende behandeling
Schildwachtklierprocedure
Oksel

C4. Beschrijf het belang van het onderzoek en de beoogde toepassing van de resultaten (verwijs eventueel naar de relevante pagina's in het protocol).
Het doel van dit onderzoek is om:
1) Overbehandeling van de oksel te verminderen;
2) Het aantal korte en lange termijn complicaties te verminderen;
3) De kwaliteit van leven te verbeteren;
4) Met een even goede ziektecontrole en algehele overleving

Dit willen wij bereiken door te onderzoeken of de schildwachtklierprocedure veilig achterwege gelaten kan worden bij patiënten met borstkanker en een klinisch negatieve okselklierstatus behandeld met borstsparende therapie.

C6. Betreft het onderzoek een multicenter-onderzoek?
- ○ nee
- ◉ ja - alleen in Nederland
- ○ ja - internationaal binnen de Europese Unie
- ○ ja - internationaal ook buiten de Europese Unie

C7. Is er bij multicenter-onderzoek sprake van een coördinerend onderzoeker?
- ◉ ja namelijk — Naam onderzoeker: M.L. Smidt
- ○ nee

C8. Wie is/zijn medisch verantwoordelijk voor de proefpersonen die deelnemen aan het onderzoek
Behandelend arts en lokale hoofdonderzoeker

C9. In welk centrum/welke centra (incl. huisartsenpraktijken) in Nederland wordt het onderzoek uitgevoerd?

Centrum	Proefpersonen	Hoofdonderzoeker	Onafhankelijk arts
Albert Schweitzer Ziekenhuis	49	M.B.E. Menke-Pluijmers	S.O. Breukink (Maastricht UMC+)
Amphia Ziekenhuis	69	E.J.T. Luiten	S.O. Breukink (Maastricht UMC+)

Antonius Ziekenhuis, Sneek	56	C.G.J.M. van Riel	S.O. Breukink (Maastricht UMC+)
Atrium Medisch Centrum	39	E.R.M. van Haaren	S.O. Breukink (Maastricht UMC+)
Bronovo Ziekenhuis	23	O.R. Guicherit	S.O. Breukink (Maastricht UMC+)
Canisius Wilhelmina Ziekenhuis	46	L.J.A. Strobbe	S.O. Breukink (Maastricht UMC+)
Catharina-ziekenhuis	46	G.A.P. Nieuwenhuijzen	S.O. Breukink (Maastricht UMC+)
Deventer Ziekenhuis	39	H. de Vries	S.O. Breukink (Maastricht UMC+)
Zorggroep De Sionsberg	15	K. Blaauwendraat	S.O. Breukink (Maastricht UMC+)
Diakonessenhuis Utrecht	39	T. van Dalen	S.O. Breukink (Maastricht UMC+)
Gelre Ziekenhuizen	37	W.B. Lastdrager	S.O. Breukink (Maastricht UMC+)
Groene Hart Ziekenhuis	31	E. Linthorst	S.O. Breukink (Maastricht UMC+)
HagaZiekenhuis	41	I. Jannink	S.O. Breukink (Maastricht UMC+)
Ikazia Ziekenhuis	27	B.R. Toorenvliet	S.O. Breukink (Maastricht UMC+)
Isala Klinieken	54	A.B. Francken	S.O. Breukink (Maastricht UMC+)
LangeLand Ziekenhuis	14	B. Jas	S.O. Breukink (Maastricht UMC+)
Laurentius Ziekenhuis	23	A.V.R.J. Bell	S.O. Breukink (Maastricht UMC+)
Academisch Ziekenhuis Maastricht	39	M.L. Smidt	S.O. Breukink (Maastricht UMC+)
Martini Ziekenhuis	43	W. Kelder	S.O. Breukink (Maastricht UMC+)
Maxima Medisch Centrum	31	K. Schenk	S.O. Breukink (Maastricht UMC+)
Meander Medisch Centrum	43	A.J. van Overbeeke	S.O. Breukink (Maastricht UMC+)
Antoni van Leeuwenhoek Ziekenhuis	93	J.A. van der Hage	S.O. Breukink (Maastricht UMC+)
Onze Lieve Vrouwe Gasthuis	28	P.J. Borgstein	S.O. Breukink (Maastricht UMC+)
Orbis Medisch Centrum	23	Y. Vissers	S.O. Breukink (Maastricht UMC+)
Universitair Medisch Centrum Sint Radboud	31	J.H.W. de Wilt	S.O. Breukink (Maastricht UMC+)
Reinier de Graaf Groep	46	M. van 't Riet	S.O. Breukink (Maastricht UMC+)
Sint Antonius Ziekenhuis	58	R. Koelemij	S.O. Breukink (Maastricht UMC+)
Sint Elisabeth Ziekenhuis	37	P. Jansen	S.O. Breukink (Maastricht UMC+)
Streekziekenhuis Koningin Beatrix	19	A. Jonk	S.O. Breukink (Maastricht UMC+)
Tergooiziekenhuizen locatie Blaricum	42	E.J.C. Vriens	S.O. Breukink (Maastricht UMC+)
Universitair Medisch Centrum Groningen	17	J. de Vries	S.O. Breukink (Maastricht UMC+)
Universitair Medisch Centrum Utrecht	23	C. van der Pol	S.O. Breukink (Maastricht UMC+)
Viecuri Medisch Centrum voor Noord-Limburg	39	F. Aarts	S.O. Breukink (Maastricht UMC+)
Westfries Gasthuis	27	J.W.D. de Waard	S.O. Breukink (Maastricht UMC+)
Waterland Ziekenhuis	22	L. de Widt	S.O. Breukink (Maastricht UMC+)
Zaans Medisch Centrum	15	S. Muller	S.O. Breukink (Maastricht UMC+)
Ziekenhuisvoorzieningen Gelderse Vallei	32	W.K. de Roos	S.O. Breukink (Maastricht UMC+)
Ziekenhuisgroep Twente	31	D. de Leeuw	S.O. Breukink (Maastricht UMC+)
Rivierenland Ziekenhuis	23	M.A.J. de Roos	S.O. Breukink (Maastricht UMC+)

Noordwest Ziekenhuisgroep	54	W.H. Schreurs	S.O. Breukink (Maastricht UMC+)
Medisch Centrum Haaglanden	31	A. Marinelli	S.O. Breukink (Maastricht UMC+)
Medisch Spectrum Twente	46	P. Steenvoorde	S.O. Breukink (Maastricht UMC+)
Rijnstate Ziekenhuis	62	R. van Eekeren	S.O. Breukink (Maastricht UMC+)
Flevoziekenhuis	50	S. van der Meij	S.O. Breukink (Maastricht UMC+)
Zuwehofpoort	15	R.C. van Doorn	S.O. Breukink (Maastricht UMC+)
Jeroen Bosch Ziekenhuis	20	M. Bessem	B. Biesma (Jeroen Bosch ziekenhuis)
Alrijne ziekenhuis	50	P. Neijenhuis	S.O. Breukink (Maastricht UMC+)
Spaarne Gasthuis	30	B. Kortmann	S.O. Breukink (Maastricht UMC+)

C10. Betreft het onderzoek met:

- ☑ mensen
- ☐ geslachtscellen
- ☐ (rest-)embryo's
- ☐ foetussen in utero

C11. Beoogd totaal aantal proefpersonen/(rest)embryo's/foetussen in utero:
C11a. In Nederland
1644

C13. Onderzoeksgebied

- ☐ etiologie
- ☐ organisatorisch/zorgonderzoek
- ☐ diagnostiek
- ☐ preventie
- ☑ therapie
- ☐ veiligheid
- ☐ werkzaamheid
- ☐ farmacokinetiek
- ☐ farmacodynamiek
- ☐ bio-equivalentie
- ☐ dosis-respons
- ☐ farmacogenomics
- ☐ farmaco-economie
- ☐ anders

C14. Type onderzoek

- ○ observationeel onderzoek zonder invasieve metingen
- ○ observationeel onderzoek met invasieve metingen
- ◉ interventie-onderzoek

C15. In welke fase kan het onderzoek worden ingedeeld?

- ○ fase I (a)
- ○ fase II (b)
- ◉ fase III (c)
- ○ fase IV (d)
- ○ overige onderzoeken waarbij geneesmiddelen worden toegepast (e)
- ○ niet van toepassing

C17. Is er sprake van een andere interventie dan met geneesmiddelen zoals bedoeld in de Wet medisch-wetenschappelijk onderzoek met mensen (WMO) (zie toelichting)?

- ◉ Ja
- ○ Nee

C17a. ☐ Medisch hulpmiddel, namelijk
C17b. ☐ Operatie, namelijk
C17c. ☐ Psychosociale interventie, namelijk
C17d. ☐ Voeding(stoffen), namelijk
C17e. ☐ Bewegingstherapie, namelijk
C17f. ☐ interventie met radioactieve straling, namelijk
C17g. ☐ Blootstellingsonderzoek (bijv pesticidenonderzoek), namelijk
C17h.
C17i. ☑ andere interventie, namelijk

In het Engels: No sentinel lymph node procedure
In het Nederlands: Géén schildwachtklierprocedure

C18. Worden de onderzoeksproducten voor deze studie door de verrichter gratis verstrekt?

○ ja
○ nee
○ gedeeltelijk namelijk
◉ niet van toepassing

C19. Is/zijn er (een) controlegroep (en)?

◉ ja
○ nee

☐ ja, geneesmiddel
☐ ja, placebo
☐ ja, geen interventie
☑ ja, anders

namelijk De standaard behandeling: schildwachtklierprocedure

C20. Betreft het een gerandomiseerd onderzoek?

◉ ja
○ nee

namelijk

☑ open
☐ enkelblind
☐ dubbelblind
☐ parallel
☐ cross-over
☐ anders namelijk

C21. Op welke klasse(n) van aandoeningen heeft het onderzoek betrekking (maximaal 3)

☐ hartaandoeningen

☐ congenitale, familiaire en genetische aandoeningen
☐ bloed- en lymfestelsel aandoeningen
☐ zenuwstelsel aandoeningen
☐ oogaandoeningen
☐ evenwichtsorgaan- en ooraandoeningen
☐ ademhalingsstelsel-, thorax- en mediastinumaandoeningen
☐ maagdarmstelselaandoeningen
☐ nier- en urinewegaandoeningen
☐ huid- en onderhuidaandoeningen
☐ skeletspierstelsel- en bindweefselaandoeningen
☐ endocriene aandoeningen
☐ voedingsstoornissen en metabole ziekten

☐ infecties en parasitaire aandoeningen
☐ letsels, intoxicaties en verrichtingscomplicaties
☑ neoplasmata, benigne, maligne en niet-gespecificeerd (incl cysten en poliepen)
borstneoplasmata maligne en niet-gespecificeerd (incl. tepel)
☑ chirurgische en medische verrichtingen
borst therapeutische verrichtingen
☐ bloedvataandoeningen
☐ algemene aandoeningen en aandoeningen op de plek van toediening
☐ zwangerschap, perinatale periode en puerperium
☐ sociale omstandigheden
☐ immuunsysteemaandoeningen
☐ lever- en galaandoeningen
☐ voortplantingsstelsel- en borstaandoeningen
☐ psychische stoornissen
☐ overig, namelijk

C22.Geef twee synoniemen voor de aandoening die bestudeerd wordt, waarvan tenminste één lekenterm.

In het Engels:	breast cancer, sentinel lymph nodes, sentinel lymph node metastases
In het Nederlands:	borstkanker, schildwachtklieren, schildwachtkliermetastasen

C23. Beoogde start- en einddatum van het onderzoek

C23a. Start Datum (dd-mm-jjjj)	01-01-2015
C23b. Eind Datum (dd-mm-jjjj)	01-01-2027

C24. Zijn patiënten betrokken bij het ontwikkelen van het onderzoek? (Voor patiënten lees ook: patiëntenorganisaties, naasten of nabestaanden van patiënten en gezonde vrijwilligers)?

C30. Wordt de ervaring van proefpersonen na deelname aan het onderzoek geëvalueerd?

D. Sectie - Proefpersonen

D1. Is er een proefpersonenverzekering conform de WMO-eisen afgesloten of wordt aan de oordelende toetsingscommissie ontheffing gevraagd?

◉ proefpersonenverzekering is afgesloten bij verzekeringsmaatschappij
○ ontheffing van de verzekering wordt gevraagd
○ niet van toepassing - onderzoek valt onder de Embryowet en niet onder de WMO

Verzekeringsmaatschappij	Marketform (onderdeel van Lloyd's)

D2. Gezonde proefpersonen en/of patiënten

☐ Gezonde proefpersonen

☑ Patiënten

Aantal	1644

D4. Voornaamste inclusiecriteria

D4a. In het Engels
- Female
- Aged 18 years or older
- Pathologically confirmed invasive unilateral breast carcinoma
- A clinical T1-2 tumour (≤ 5cm)
- Will be treated with lumpectomy followed by whole breast radiotherapy
- Clinically node negative status: no signs of axillary lymph node metastases at physical examination and preoperative axillary ultrasound (or negative cyto-/histopathology)
- Written informed consent

D4b. In het Nederlands
- Vrouw
- Leeftijd 18 jaar of ouder
- Pathologisch bewezen invasief unilaterale borstkanker
- Klinisch T1-2 tumor (≤ 5cm)
- Zal worden behandeld met lumpectomie gevolgd door gehele borstbestraling
- Klinisch negatieve okselklierstatus: negatief lichamelijk onderzoek en echografie van de oksel (of negatieve cyto-/histologie)
- Schriftelijke informed consent

D5. Voornaamste exclusiecriteria

D5a. In het Engels

- Clinically node positive pre-operative
- Bilateral breast cancer
- Evidence of metastatic disease
- History of invasive breast cancer
- Previous treatment of the ipsilateral axilla with surgery or radiotherapy, except surgery for hidradenitis suppurativa or for other superficially located skin lesions, such as naevi
- Pregnant or nursing
- Other prior malignancies within the past 5 years, except successfully treated malignancies that occurred more than five years before randomization, and except successfully treated basal cell and squamous cell skin cancer, carcinoma in situ of the cervix or carcinoma in situ of the ipsilateral or contralateral breast
- Unable or unwilling to give informed consent

D5b. In het Nederlands

- Klinisch positieve okselklierstatus (preoperatief)
- Bilaterale borstkanker
- Primair (afstands-)gemetastaseerde borstkanker
- Invasieve borstkanker in de voorgeschiedenis
- Chirurgie of radiotherapie van ipsilaterale oksel in de voorgeschiedenis, behalve chirurgie voor hidradenitis suppurativa of andere oppervlakkig gelegen huidlaesies, zoals naevi
- Zwanger of lacterend
- Andere maligniteit in de afgelopen 5 jaar, behalve succesvol behandelde maligniteit die meer dan vijf jaar voor randomisatie optrad, en behalve succesvol behandelde huidkanker van type basaalcel of plaveiselcel, carcinoma in situ van de cervix, carcinoma in situ van de ipsilaterale of contralaterale mamma
- Niet in staat of niet bereid tot informed consent

D6. Bij welke categorie proefpersonen wordt het onderzoek uitgevoerd (meerdere antwoorden mogelijk)

☑ 18 jaar of ouder en wilsbekwaam
☐ 18 jaar of ouder en wilsonbekwaam
☐ 12 t/m 17 jaar en in staat tot het geven van geïnformeerde toestemming
☐ 12 t/m 17 jaar en niet in staat tot het geven van geïnformeerde toestemming (wilsonbekwaam)
☐ jonger dan 12 jaar

D10. Verkeren (sommige) proefpersonen in een afhankelijkheidssituatie ten opzichte van de onderzoeker of degene die de deelnemers werft? (lees de toelichting voor voorbeelden wanneer er sprake kan zijn van een afhankelijkheidssituatie)

◉ ja
○ nee

D10a. Zo ja, waardoor?

Soms is de lokale onderzoeker ook de behandelend arts die de patiënt vraagt om deel te nemen aan de studie.

D10b. Waarom wordt het onderzoek juist met deze proefpersonen uitgevoerd en hoe worden de belangen van de proefpersonen gewaarborgd?

Het onderzoek moet met proefpersonen worden uitgevoerd die voldoen aan de beschreven in/exclusie criteria zodat de resultaten van de studie uiteindelijk ook naar de kliniek vertaald kunnen worden binnen deze patiëntenpopulatie. De belangen van de proefpersonen worden gewaarborgd door de geldende medisch ethische normen en richtlijn zoals vermeld in het protocol (o.a. onafhankelijk arts, uitgebreide patiëntinformatiefolder).

D11. Waaruit bestaat de vergoeding voor de proefpersonen?

☑ geen vergoeding
☐ reiskosten
☐ financiële vergoeding (in Euro's)
☐ andere vergoeding

D12. Is deze vergoeding afhankelijk van bepaalde voorwaarden, bijvoorbeeld het voltooien van (een deel van) het onderzoek?

○ ja (motiveer)
○ nee
◉ niet van toepassing

E. Sectie - Voor- en nadelen

E1. Wordt er bij dit onderzoek een rechtstreeks therapeutisch effect beoogd bij de proefpersonen / patiënten?

◉ ja (therapeutisch onderzoek)
○ nee (niet-therapeutisch onderzoek)

E1a. Zo ja, geef kort aan waaruit dit therapeutisch effect bestaat.

Het therapeutische effect bestaat uit:
- Het verlagen van het risico op okselbehandeling-gerelateerde korte en lange termijn complicaties;
- Het verbeteren van de kwaliteit van leven;
- Met een even goede ziektecontrole en algehele overleving.

E2. Waaruit bestaat de belasting van het onderzoek (en een eventueel daaraan voorafgaande keuring) voor de proefpersonen?

Tijdbeslag	per bezoek	20 min per keer voor invullen kwaliteit van leven vragenlijst
	totaal	20 min x 6 = 120 min
	totale duur van de studie voor de individuele proefpersoon	Tav de behandeling minder belasting dan normaal voor de studie-arm (geen schildwachtklierprocedure). Voor beide armen is extra het invullen van de kwaliteit van leven vragenlijst prerandomisatie en postrandomisatie op 5 momenten.

E3. Worden de proefpersonen in verband met het onderzoek in het ziekenhuis opgenomen of wordt een opname verlengd?

- ○ ja - het verblijf in het ziekenhuis/instituut wordt in verband met het onderzoek verlengd
- ○ ja - ze worden voor het onderzoek in het ziekenhuis/instituut opgenomen
- ◉ nee

E4. Beschrijf in hoeverre proefpersonen worden onderworpen aan handelingen dan wel een gedragswijze krijgen opgelegd, zoals vragenlijst, interviews, lichamelijk/psychologisch onderzoek, ontzegging, dieet (voor invasieve ingrepen: zie vraag E6)

In totaal zullen 700 proefpersonen (350 in de experimentele en 350 in de controlegroep) deelnemen aan het kwaliteit van leven onderzoek, bestaande uit een vragenlijst voor de randomisatie en op 5 tijdstippen na de randomisatie (na een half jaar, één, twee, vijf en 10 jaar).

E5. Worden de proefpersonen getest op bepaalde aandoeningen/condities?

- ○ ja (motiveer)
- ◉ nee

E6. Welke extra (invasieve) ingrepen (anders dan bij de standaard behandeling) moeten de proefpersonen in het kader van het onderzoek ondergaan:

- ☑ Niet van toepassing

E9. Geef aan welke risico's er voor proefpersonen zijn verbonden aan deelname aan het onderzoek.

Hoewel de hypothese luidt dat het achterwege laten van de schildwachtklierprocedure geen nadelige invloed heeft op de lokale controle van de ziekte en de overlevingskans, bestaat er toch een kans dat proefpersonen in de experimentele arm een hoger risico hebben op een recidief in de oksel (regionaal recidief) en daardoor tijdens follow-up alsnog een vorm van okselklierbehandeling moeten ondergaan. Uit onderzoek blijkt dat het uitgesteld uitvoeren van okselklierbehandeling (bijvoorbeeld met okselklierdissectie) géén nadelige gevolgen heeft voor de overleving.

E9a. Geef op grond van uw eigen afweging aan waarom het uitvoeren van het onderzoek, in het licht van de belasting en/of risico's die voor proefpersonen aan deelname verbonden zijn, gerechtvaardigd is?

Het mogelijk hogere risico op een recidief in de oksel (regionaal recidief) weegt op tegen de lagere kans op ernstige okselklierbehandeling-gerelateerde morbiditeit (lymfoedeem, bewegingsbeperking in de schouder, arm- en schouderpijn, verminderd gevoel of zenuwpijn) en de verbetering van kwaliteit van leven.

E10. Indien het onderzoek bij minderjarige en/of wilsonbekwame proefpersonen wordt uitgevoerd en geen direct therapeutisch effect wordt beoogd: waarom kunnen belasting en risico's als minimaal worden beschouwd? (Verwijs eventueel naar de relevante pagina's in het protocol.

- ☑ niet van toepassing

E11. Kan de eventuele therapie na beëindiging van het onderzoek worden voortgezet?

- ○ ja (motiveer)
- ○ nee (motiveer)
- ◉ niet van toepassing

E12. Heeft deelname aan het onderzoek voor de proefpersoon tot gevolg dat van de standaardbehandeling of -diagnostiek kan worden afgeweken of deze kan worden uitgesteld?

- ◉ ja
- ○ nee
- ○ niet van toepassing

E12a. Zo ja, waaruit bestaat de afwijking of het uitstel en waarom is afwijking/uitstel

Proefpersonen in de controlegroep ondergaan de standaard behandeling, namelijk schildwachtklierprocedure volgens de Nederlandse borstkankerrichtlijn. Proefpersonen in de experimentele arm ondergaan primair géén schildwachtklierprocedure. Beide groepen ondergaan dezelfde follow-up middels lichamelijk onderzoek en aanvullend onderzoek op indicatie. Indien een proefpersoon zich in de follow-up periode presenteert met lymfekliermetastasen, kan alsnog besloten worden om een vorm van okselklierbehandeling uit te voeren. Deze beslissing wordt gemaakt tijdens het multidisciplinair overleg in het desbetreffende centrum waar de patiënt onder behandeling staat.

F. Sectie - Informatie en privacy

F1. Hoe worden de proefpersonen geworven en door wie (onderzoeker, behandelend arts, andere persoon) wordt de proefpersoon/wettelijke vertegenwoordiger geïnformeerd en om toestemming gevraagd?

Op het moment in de behandeling van borstkanker dat de patiënt voldoet aan de in/exclusiecriteria zal de behandeld arts informatie geven over de mogelijkheid tot deelname aan deze studie. Een schriftelijke patiëntinformatiebrief zal worden meegegeven aan de patiënt. De patiënt krijgt ruim de tijd (ten minste één week) om deze informatie thuis na te lezen en een beslissing te nemen. De patiënt kan haar interesse in deelname kenbaar maken aan haar behandelend arts bij een volgend polibezoek. De behandelend arts zal haar dan om toestemming vragen voor deelname aan de studie.

F2. Hoeveel bedenktijd krijgen de proefpersonen/wettelijke vertegenwoordigers om te beslissen over deelname?

Zo lang als nodig, met ten minste één week

F3. Wordt de huisarts, behandelend specialist en/of apotheker van de proefpersoon geïnformeerd over diens deelname aan het onderzoek?

- (●) ja (de proefpersoon dient hiervoor toestemming te geven)
- ( ) nee

F4. Worden persoonsgegevens gecodeerd?

- (●) ja
- ( ) nee

F4a. Zo ja, hoe is deze codering opgebouwd?

Alle proefpersonen krijgen een uniek studienummer. Het unieke studienummer betreft een opvolgnummer zonder verdere specifieke codering voor bijvoorbeeld centrum.

F4b. Wie heeft toegang tot de sleutel van deze code?

De behandelend arts en de onderzoeker

F4c. Wie hebben toegang tot de brondocumenten en eventuele andere tot de persoon herleidbare gegevens?

De lokaal datamanagers en de onderzoeker

F5. Hoe wordt het lichaamsmateriaal <u>gedurende</u> het onderzoek bewaard?

- ( ) in tot de proefpersoon herleidbare vorm (gecodeerd)
- ( ) in niet tot de proefpersoon herleidbare vorm (volledig geanonimiseerd)
- (●) niet van toepassing

F6. Wordt afgenomen lichaamsmateriaal na afloop van het onderzoek vernietigd?

- ( ) ja
- ( ) nee (motiveer)
- (●) niet van toepassing

F7. Kunnen proefpersonen na afloop van het onderzoek opnieuw benaderd worden (bijvoorbeeld voor nader onderzoek of follow-up)?

- ( ) ja
- (●) nee

G. Sectie - Financieel

G1. Door welke geldstroom wordt het onderzoek gefinancierd?

- [ ] eerste geldstroom (Geld van Ministerie van OC&W aan universiteiten)
- [ ] tweede geldstroom (NWO of KNAW), namelijk
- [x] derde geldstroom (anders dan 1e of 2e geldstroom, zoals collectebusfondsen, Europese Unie, vakministeries of bedrijven), namelijk

KWF Kankerbestrijding

G2. Wordt het onderzoek (mede) gefinancierd door de industrie/bedrijven?

- ○ ja - door de industrie/bedrijf zoals is opgegeven bij vraag B6/B7 (opdrachtgever van het onderzoek)
- ○ ja - (ook) door andere industrie/bedrijven dan de opdrachtgever
- ◉ nee

G3. Wat is de hoogte van de vergoeding die de arts/onderzoeker cq onderzoeksafdeling/maatschap ontvangt voor de uitvoering van het onderzoek?

- ○ Per patiënt of proefpersoon
- ◉ Per deelnemend centrum

bedrag afgerond in hele euro's: € 100,00

G3a. Hoe is de vergoeding opgebouwd?

De vergoeding betreft een financiële compensatie van € 100 excl. BTW als opstartvergoeding voor ieder deelnemend centrum.

G4. Heeft/hebben de onderzoeker(s) gedurende de afgelopen vijf jaar op een of andere wijze een persoonlijke financiële relatie (gehad) met de verrichter/sponsor van het huidige onderzoek?

- ○ ja (licht toe)
- ◉ nee

I. Sectie - Indiening en beoordeling

I1. Sla het formulier eerst op en selecteer vervolgens een METC of de CCMO

Commissie
METC NedMec

J. Sectie - Aanvullende opmerkingen

Aanvullende opmerkingen

K. Sectie - Samenvatting

Achtergrond van het onderzoek:

De schildwachtklierprocedure is de standaard methode voor lymfeklierstadiëring in borstkankerpatiënten met een klinisch negatieve okselklierstatus. In de algemene bevolking bevat de schildwachtklier in ongeveer 25% een uitzaaiing. Dit percentage is lager in patiënten met een kleine borsttumor en in borstsparend behandelde patiënten. De ACOSOG-Z0011 studie en de IBCSG 23-01 studie hebben recent aangetoond dat een completerende okselklierdissectie veilig achterwege gelaten kan worden bij borstsparend en met systemische therapie behandelde patiënten met een beperkt aantal schildwachtkliermetastasen. Dit roept de vraag op of de schildwachtklierprocedure nog wel noodzakelijk is voor borstsparend behandelde patiënten met een klinisch negatieve okselklierstatus. De klinisch negatieve okselklierstatus van de patiënten in de beschreven studies werd gesteld op basis van lichamelijk onderzoek. Het toevoegen van een preoperatieve echografie van de oksel zal zorgen voor een betere voorselectie van patiënten zonder lymfekliermetastasen, ten opzichte van lichamelijk onderzoek alleen. Daarbij sluit een negatieve echografie uitgevoerd in Nederland bijna alle patiënten uit met uitgebreide lymfekliermetastasen (≥4 lymfekliermetastasen). Bovendien omvat gehele borstbestraling na de borstsparende operatie in het algemeen het lagere deel van de oksel en behandelt daarmee eventueel achtergebleven uitzaaiingen. Hoewel de schildwachtklierprocedure minder invasief is vergeleken met een okselklierdissectie, komen complicaties nog steeds voor. Zo lijdt 6% van de patiënten die alleen een schildwachtklierprocedure ondergingen aan ernstige lymfoedeem na slechts 3 jaar. Deze studie gaat onderzoeken of de schildwachtklierprocedure veilig achterwege gelaten kan worden bij borstsparend behandelde patiënten, met een negatieve echografie van de oksel.

Doel van het onderzoek:

Het doel van deze studie is het verminderen van overbehandeling van de oksel bij patiënten met borstkanker en een klinisch negatieve okselklierstatus die behandeld worden met borstsparende therapie, door het achterwege laten van de schildwachtklierprocedure, om zodoende de kans op korte en lange termijn complicaties te verminderen en te zorgen voor een verbetering van de kwaliteit van leven met een even goede ziektecontrole en algehele overleving.

Onderzoeksopzet:

Het betreft een gerandomiseerde non-inferiority multicenter onderzoek. Patienten die voldoen aan de in- en exclusiecriteria worden na toestemming middels loting behandeld volgens één van de volgende behandelmethoden:

- Behandelmethode A (controle groep): schildwachtklierprocedure volgens de Nederlandse borstkankerrichtlijn.
- Behandelmethode B (studie groep): geen verdere okselklierstadiëring.

Stratificatiefactoren: leeftijd (≤50, 50≤75, >75 jaar), oestrogeen receptor status (positief vs. negatief), HER2neu status (geamplificeerd vs. niet geamplificeerd), klinisch tumor stadium (<3 cm vs. ≥3 cm), tumorgraad volgens gemodificeerde Bloom-Richardson (graad I-II vs. graad III), is of zal worden behandeld met primaire systemische therapie (ja/nee), participerend centrum.
Overige behandelingen (o.a. hormonale therapie, chemotherapie) worden op indicatie gegeven volgens de Nederlandse borstkankerrichtlijn.
Tijdens de follow-up periode van 10 jaar wordt de oksel nauwkeurig onderzocht middels lichamelijk onderzoek en echografie op indicatie. Indien sprake is van een cyto-/histologisch bewezen lymfekliermetastase tijdens follow-up, dan kan in het multidisciplinair overleg van de desbetreffende patiënt worden besloten tot het uitvoeren van een vorm van okselklierbehandeling.
Gevalideerde vragenlijsten worden verstrekt voor het onderzoeken van de invloed op het aantal complicaties en kwaliteit van leven (Lymph-ICF, QLQ-C30, QLQ-BR23) en om te meten of angst en persoonlijkheidstrekken invloed hebben op de uitkomst van

kwaliteit van leven (STAI-trait, NEO-FFI). Deze vragenlijsten worden verstrekt aan 700 van de 1644 patiënten (350 per arm), éénmaal voor de randomisatie en vijfmaal na de randomisatie (6 maanden, 1 jaar, 2 jaar, 5 jaar en 10 jaar). Vragenlijsten met betrekking to kosten-effectiviteit worden éénmaal voor de randomisatie en viermaal na de randomisatie (6 maanden, 1,2 en 3 jaar) afgenomen.

Onderzoekspopulatie:

Vrouwen van 18 jaar of ouder met pathologisch bewezen invasief mammacarcinoom die borstsparende therapie ondergaan.
cT1-2: primaire tumor is bij klinisch onderzoek kleiner dan 5 cm.
cN0: bij lichamelijk onderzoek en echografie van de oksel worden geen aanwijzingen voor lymfekliermetastasen gevonden of de cytologie/histologie na punctie/biopt van een lymfeklier is negatief.
Neoadjuvante systemische therapie is toegestaan indien de patiënt voor de start van de neoadjuvante systemische therapie een klinische T1-2N0 status heeft en waarvoor borstsparende therapie op dat moment al geschikt is.
De patiënt heeft informed consent persoonlijk ondertekend.

Interventie (indien van toepassing):

De controlegroep ondergaat wél de schildwachtklierprocedure volgens de Nederlands borstkankerrichtlijn. De experimentele arm ondergaan géén schildwachtklierprocedure.

Primaire onderzoeksvariabelen/uitkomstmaten:

Primaire uitkomstmaat:
- Regionaal recidief percentage

Secundaire onderzoeksvariabelen/uitkomstmaten (indien van toepassing):
Secundaire uitkomstmaten:
- Distant-disease free survival
- Overall survival
- Later uitgevoerde okselbehandeling
- Lokaal recidief percentage
- Overig-regionaal recidief percentage
- Contralaterale borstkanker
- Percentage (neo)adjuvante systemische therapie
- Kwaliteit van leven en okselbehandeling-gerelateerde morbiditeit
- Kosten-effectiviteit

Omschrijving en inschatting van belasting en risico (indien van toepassing):

Wat betreft belasting is er in deze studie géén sprake van extra diagnostiek of interventies (juist minder) en hoeft de patiënt niet vaker dan normaal voor controle naar het ziekenhuis. De eventuele extra belasting voor de patiënt zal alleen bestaan uit het invullen van kwaliteit van leven vragenlijsten (bij 700 van 1644 patiënten) éénmaal voor de randomisatie en vijfmaal na randomisatie (6 maanden, 1 jaar, 2 jaar, 5 jaar en 10 jaar). De hypothese luidt dat het achterwege laten van de schildwachtklierprocedure even veilig is als het wél uitvoeren van deze procedure. Desondanks is er een risico dat in de experimentele arm meer regionale recidieven optreden en dat bij patiënten op een later tijdstip alsnog een vorm van okselklierbehandeling moet worden uitgevoerd. Daartegenover staat dat er in de experimentele arm door het niet uitvoeren van de schildwachtklierprocedure minder kans is op morbiditeit, minder operaties en opnames in het ziekenhuis en een betere kwaliteit van leven. Patiënten zullen op geen enkele manier vertraging ondervinden als gevolg van deelname aan de studie.

K2.Engelse Samenvatting

Background of the study:
The NSABP B-04 trial revealed that omitting primary axillary treatment of occult positive lymph nodes in clinically node negative breast cancer patients does not affect survival, even after 25 years of follow-up and without the use of adjuvant systemic or radiation therapy. A delayed axillary dissection in case lymph nodes become clinically positive does not affect survival and prevents axillary overtreatment in the majority of patients. The ACOSOG Z0011 and IBCSG 23-01 trials revealed that completion axillary dissection can be safely omitted in clinically node negative patients with metastatic sentinel nodes. Patients randomized for watchful waiting were likely to have residual nodal disease (13-27%) that was not surgically removed. Nevertheless, survival rates were not affected and regional recurrence rates low. Use of axillary ultrasound will improve the preoperative selection of node negative patients, as it selects patients with a more favourable tumour load and accurately excludes advanced nodal disease (≥4 metastatic nodes). Biology and adjuvant systemic and radiation therapy are all factors that most likely diminish the risk that possible metastases left in situ develop into clinically detectable lymph nodes. Patients treated with breast conserving therapy are significantly more often diagnosed with pathologic node negative disease or with micrometastatic disease, compared to patients treated with mastectomy. The risk for occult (macro)metastases in cT1-2N0 breast cancer patients treated with breast conserving therapy is low: about 88% will be node negative and about 95% of node positive patients will have no lymph node metastases beyond the sentinel node. Reflected by the low regional recurrence rate after a (false) negative sentinel node, only a small amount of patients (0.8%) with node positive disease is expected to develop clinically detectable nodal disease.

Objective of the study:
The objective of this study is to decrease the number of breast cancer patients receiving overtreatment of the axilla, in order to positively influence the axillary morbidity rate and quality of life. Therefore, we investigate whether the sentinel lymph node procedure can be safely omitted in clinically node negative breast cancer patients undergoing breast conserving therapy, in terms of non-inferior regional control, distant-disease free and overall survival. All objectives are measured during a follow-up of 10 years.

Study design:
A randomized controlled non-inferiority multicenter study. Patients who meet the in- and exclusioncriteria will be randomized after

informed consent for one of the two treatment methods:
- Arm A (control arm): lumpectomy with sentinel lymph node procedure, followed by radiotherapy of the breast with or without completion axillary treatment according to the Dutch breast cancer guideline.
- Arm B (experimental arm): lumpectomy without further axillary staging, followed by radiotherapy of the breast.
Stratification: age (≤50, 50≤75, >75 years old), oestrogen receptor status (positive vs. negative), HER2neu status (amplified vs. not amplified), clinical tumour stage prior to any treatment (<3 cm vs. ≥3 cm), grading (grade I-II vs. III - according to Bloom-Richardson grading system), is or will be treated with primary systemic therapy (yes/no) and participating centre.
Adjuvant systemic treatment is administered if indicated according to the Dutch breast cancer guideline.
Primary systemic therapy is allowed if the patient has a clinical T1-2N0 status (initial stage) that is amenable to lumpectomy pre-systemic therapy.
Yearly follow-up with physical examination of the axilla for 10 years. Axillary ultrasound (+/- tissue sampling) on indication. Staging for distant metastases and/or a delayed axillary lymph node dissection if indicated by the multidisciplinary team. Validated questionnaires are used to assess axillary morbidity rate and quality of life (Lymph-ICF, QLQ-C30, QLQ-BR23) and to measure if anxiety and personality traits influence the outcome of quality of life (STAI-trait, NEO-FFI) in 700 of 878 patients. These questionnaires are provided before randomization and sequentially post randomization at 6 months, 1, 2, 5 and 10 years. Cost-effectiveness questionnaires are provided before randomization and sequentially post randomization at 6 months, 1, 2 and 3 years.

Study population:
Women aged 18 years or older with pathologically confirmed invasive breast carcinoma about to undergo breast conserving surgery followed by radiotherapy of the breast.
cT1-2: primary tumour is smaller than 5 cm.
cN0: no signs of axillary lymph node metastases at physical examination and axillary ultrasound (+/- tissue sampling).
Primary systemic treatment is allowed.
Patient is able to and signed the informed consent form.

Intervention (if applicable):
Patients in the control arm will be treated with the sentinel lymph node procedure according to the Dutch breast cancer guideline.
Patients in the experimental arm will not be treated with the sentinel lymph node procedure.

Primary study parameters/outcome of the study:
Primary endpoint:
- Regional recurrence rate

Secundary study parameters/outcome of the study (if applicable):
Secondary endpoints:
- Distant-disease free survival
- Overall survival
- Delayed axillary treatment
- Local recurrence rate
- Other-regional recurrence rate
- Contralateral breast cancer rate
- Percentage difference in the administration of (neo)adjuvant systemic therapy
- Quality of life and axillary morbidity rate
- Cost-effectiveness

Nature and extent of the burden and risks associated with participation, benefit and group relatedness (if applicable):
There is no extra burden for participating patients as regard to additional diagnostic tests/interventions and follow-up. Any additional burden for the patient will only consist of completing quality of life questionnaires in 700 of 1644 study patients. These questionnaires are provided before randomization and sequentially post randomization at 6 months, 1, 2, 5 and 10 years.
We hypothesize that omitting the sentinel lymph node procedure is not inferior to the standard treatment. Nevertheless, possible disadvantages are a worse regional recurrence rate, and delayed axillary treatment if indicated. Patients in the experimental arm have the possible advantages of less morbidity, fewer operations and hospitalizations and an improved quality of life, compared to patients treated with the sentinel lymph node procedure.
No patient will encounter any delay in their treatment as a result of inclusion.

ONDERTEKENING

De verrichter en indiener verklaren hierbij:
a. het formulier (en samenvatting) volledig en naar waarheid te hebben ingevuld;
b. de antwoorden op de vragen uit het ABR-formulier niet in strijd zijn met het bijbehorende onderzoeksdossier en onderzoekscontract

Naar waarheid getekend, door de verrichter (=opdrachtgever)		door de indiener	
datum	....	datum	....

Handtekening		Handtekening	
naam	U.P. Neumann	naam	J.A. van der Hage
functie	Hoofd afdeling Chirurgie, MUMC+	functie	Chirurg, NKI-AVL

Sluiten Print

Sluiten Print

Formulier voor medisch-ethische beoordeling en registratie

ABR-formulier, versie december 2015

Onderzoeksdossiernummer

ABR Nummer	**49315**	Status	**Definitief**
Versie	**09**	Status per	**27-10-2017**
Jaar	**17**		
Dossiernummer	**NL49315.031.14**		
Reden voor Amendement	**Toevoegen centra**		

A. Sectie - Openbaar maken gegevens medisch wetenschappelijk onderzoek

A1. Het CCMO-register is een voor ieder toegankelijk openbaar trial register. De antwoorden op de vragen gemarkeerd met een wereldbol en de samenvatting bij dit formulier worden openbaar gemaakt in het CCMO register.

B. Sectie - Administratief

B1. Betreft het onderzoek met geneesmiddelen (inclusief gentherapie, somatische celtherapie, vaccinonderzoek, GGO's, zie verdere toelichting) als bedoeld in de Wet medisch-wetenschappelijk onderzoek met mensen (WMO)?

- ○ ja
- ◉ nee

B2. Houdt het onderzoek verband met een eerder door een erkende METC of door de CCMO beoordeelde studie of is het onderzoek reeds eerder bij een erkende METC ter beoordeling voorgelegd?

- ○ ja, het onderzoek houdt verband met – of is het vervolg op – een eerder beoordeelde studie
- ○ ja, het onderzoek is eerder ter beoordeling aan een erkende METC of de CCMO voorgelegd (stuur kopie besluit mee)
- ◉ nee

B4. Is het protocol (nog) in een ander openbaar trial register geregistreerd?

- ○ ja
- ◉ nee

B5. Naam indiener/contactpersoon voor de oordelende toetsingscommissie

**B5a.**	Achternaam indiener/contactpersoon	Duijnhoven
	Titel en voorletters	dr. F.
	Tussenvoegsels	van
**B5b.**	Type organisatie/bedrijf	Overige Ziekenhuizen
	Organisatie/bedrijf	Antoni van Leeuwenhoek Ziekenhuis
	Naam organisatie/bedrijf	IKNL trialbureau
	Afdeling	Chirurgie
	Adres	Plesmanlaan 121
	Postcode en plaats	1066 CX Amsterdam
	Land	NL
**B5c.**	Intern adres	afdeling Heelkunde
	Telefoon	020 512 9111
	Fax	020 512 2554
	E-mail	f.v.duijnhoven@nki.nl

B6. Is de indiener werkzaam bij de opdrachtgever/sponsor (verrichter) van het onderzoek?

○ Ja ◉ Nee

B7. Opdrachtgever/sponsor van het onderzoek (verrichter volgens de WMO)

Type Organisatie/Bedrijf	Universitair Medisch Centrum
Organisatie/Bedrijf	Medisch Universitair Ziekenhuis Maastricht

Adres	P. Debyelaan 25
Postcode en plaats	6229 HX Maastricht
Land	NL
Telefoon	0433877477
Fax	0433875473
E-mail	m.smidt@mumc.nl

C. Sectie - Onderzoek

C1. Volledige titel van het onderzoek

C1a. In het Engels

Clinically node negative breast cancer patients undergoing breast conserving therapy: Sentinel lymph node procedure versus follow-up. A Dutch randomized controlled multicentre trial.

C1b. In het Nederlands

Patiënten met borstkanker en een klinisch negatieve okselklierstatus die een borstsparende behandeling ondergaan: Schildwachtklierprocedure versus follow-up. Een Nederlandse gerandomiseerde multicenter studie.

C2. Verkorte titel van het onderzoek/acroniem

C2a. In het Engels

BOOG 2013-08

C2b. In het Nederlands (Let op: deze korte titel wordt vermeld binnen ToetsingOnline)

BOOG 2013-08

C3. Trefwoorden (maximaal 4, plaats elk trefwoord op een aparte regel)

C3a. In het Engels

Breast neoplasms
Breast conserving therapy
Sentinel lymph node biopsy
Axilla

C3b. In het Nederlands

Borstkanker
Borstsparende behandeling
Schildwachtklierprocedure
Oksel

C4. Beschrijf het belang van het onderzoek en de beoogde toepassing van de resultaten (verwijs eventueel naar de relevante pagina's in het protocol).

Het doel van dit onderzoek is om:
1) Overbehandeling van de oksel te verminderen;
2) Het aantal korte en lange termijn complicaties te verminderen;
3) De kwaliteit van leven te verbeteren;
4) Met een even goede ziektecontrole en algehele overleving

Dit willen wij bereiken door te onderzoeken of de schildwachtklierprocedure veilig achterwege gelaten kan worden bij patiënten met borstkanker en een klinisch negatieve okselklierstatus behandeld met borstsparende therapie.

C6. Betreft het onderzoek een multicenter-onderzoek?

○ nee
◉ ja - alleen in Nederland
○ ja - internationaal binnen de Europese Unie
○ ja - internationaal ook buiten de Europese Unie

C7. Is er bij multicenter-onderzoek sprake van een coördinerend onderzoeker?

◉ ja namelijk — Naam onderzoeker: M.L. Smidt
○ nee

C8. Wie is/zijn medisch verantwoordelijk voor de proefpersonen die deelnemen aan het onderzoek

Behandelend arts en lokale hoofdonderzoeker

C9. In welk centrum/welke centra (incl. huisartsenpraktijken) in Nederland wordt het onderzoek uitgevoerd?

Centrum	Proefpersonen	Hoofdonderzoeker	Onafhankelijk arts
Albert Schweitzer Ziekenhuis	49	M.B.E. Menke-Pluijmers	S.O. Breukink (Maastricht UMC+)
Amphia Ziekenhuis	69	E.J.T. Luiten	S.O. Breukink (Maastricht UMC+)

Antonius Ziekenhuis, Sneek	56	C.G.J.M. van Riel	S.O. Breukink (Maastricht UMC+)
Atrium Medisch Centrum	39	E.R.M. van Haaren	S.O. Breukink (Maastricht UMC+)
Bronovo Ziekenhuis	23	O.R. Guicherit	S.O. Breukink (Maastricht UMC+)
Canisius Wilhelmina Ziekenhuis	46	L.J.A. Strobbe	S.O. Breukink (Maastricht UMC+)
Catharina-ziekenhuis	46	G.A.P. Nieuwenhuijzen	S.O. Breukink (Maastricht UMC+)
Deventer Ziekenhuis	39	H. de Vries	S.O. Breukink (Maastricht UMC+)
Diakonessenhuis Utrecht	39	T. van Dalen	S.O. Breukink (Maastricht UMC+)
Gelre Ziekenhuizen	37	W.B. Lastdrager	S.O. Breukink (Maastricht UMC+)
Groene Hart Ziekenhuis	31	E. Linthorst	S.O. Breukink (Maastricht UMC+)
HagaZiekenhuis	41	I. Jannink	S.O. Breukink (Maastricht UMC+)
Isala Klinieken	54	A.B. Francken	S.O. Breukink (Maastricht UMC+)
Laurentius Ziekenhuis	23	A.V.R.J. Bell	S.O. Breukink (Maastricht UMC+)
Academisch Ziekenhuis Maastricht	39	M.L. Smidt	S.O. Breukink (Maastricht UMC+)
Martini Ziekenhuis	43	W. Kelder	S.O. Breukink (Maastricht UMC+)
Maxima Medisch Centrum	31	K. Schenk	S.O. Breukink (Maastricht UMC+)
Meander Medisch Centrum	43	A.J. van Overbeeke	S.O. Breukink (Maastricht UMC+)
Antoni van Leeuwenhoek Ziekenhuis	93	J.A. van der Hage	S.O. Breukink (Maastricht UMC+)
Onze Lieve Vrouwe Gasthuis	28	S. Donkervoort	S.O. Breukink (Maastricht UMC+)
Orbis Medisch Centrum	23	Y. Vissers	S.O. Breukink (Maastricht UMC+)
Universitair Medisch Centrum Sint Radboud	31	J.H.W. de Wilt	S.O. Breukink (Maastricht UMC+)
Reinier de Graaf Groep	46	M. van 't Riet	S.O. Breukink (Maastricht UMC+)
Sint Antonius Ziekenhuis	58	R. Koelemij	S.O. Breukink (Maastricht UMC+)
Sint Elisabeth Ziekenhuis	37	P. Jansen	S.O. Breukink (Maastricht UMC+)
Streekziekenhuis Koningin Beatrix	19	A. Jonk	S.O. Breukink (Maastricht UMC+)
Tergooiziekenhuizen locatie Blaricum	42	E.J.C. Vriens	S.O. Breukink (Maastricht UMC+)
Universitair Medisch Centrum Groningen	17	J. de Vries	S.O. Breukink (Maastricht UMC+)
Universitair Medisch Centrum Utrecht	23	C. van der Pol	S.O. Breukink (Maastricht UMC+)
Viecuri Medisch Centrum voor Noord-Limburg	39	F. Aarts	S.O. Breukink (Maastricht UMC+)
Westfries Gasthuis	27	J.W.D. de Waard	S.O. Breukink (Maastricht UMC+)
Waterland Ziekenhuis	22	L. de Widt	S.O. Breukink (Maastricht UMC+)
Zaans Medisch Centrum	15	S. Muller	S.O. Breukink (Maastricht UMC+)
Ziekenhuisvoorzieningen Gelderse Vallei	32	W.K. de Roos	S.O. Breukink (Maastricht UMC+)
Ziekenhuisgroep Twente	31	D. de Leeuw	S.O. Breukink (Maastricht UMC+)
Rivierenland Ziekenhuis	23	P.I. Welvaart	S.O. Breukink (Maastricht UMC+)
Noordwest Ziekenhuisgroep	54	W.H. Schreurs	S.O. Breukink (Maastricht UMC+)
Medisch Centrum Haaglanden	31	A. Marinelli	S.O. Breukink (Maastricht UMC+)
Medisch Spectrum Twente	46	P. Steenvoorde	S.O. Breukink (Maastricht UMC+)

Rijnstate Ziekenhuis	62	R. van Eekeren	S.O. Breukink (Maastricht UMC+)
Flevoziekenhuis	50	S. van der Meij	S.O. Breukink (Maastricht UMC+)
Zuwehofpoort	15	R.C. van Doorn	S.O. Breukink (Maastricht UMC+)
Jeroen Bosch Ziekenhuis	20	M. Bessem	B. Biesma (Jeroen Bosch ziekenhuis)
Alrijne ziekenhuis	50	P. Neijenhuis	S.O. Breukink (Maastricht UMC+)
Spaarne Gasthuis	30	B. Kortmann	S.O. Breukink (Maastricht UMC+)

C10. Betreft het onderzoek met:

- [x] mensen
- [ ] geslachtscellen
- [ ] (rest-)embryo's
- [ ] foetussen in utero

C11. Beoogd totaal aantal proefpersonen/(rest)embryo's/foetussen in utero:
C11a. In Nederland
1644

C13. Onderzoeksgebied

- [ ] etiologie
- [ ] organisatorisch/zorgonderzoek
- [ ] diagnostiek
- [ ] preventie
- [x] therapie
- [ ] veiligheid
- [ ] werkzaamheid
- [ ] farmacokinetiek
- [ ] farmacodynamiek
- [ ] bio-equivalentie
- [ ] dosis-respons
- [ ] farmacogenomics
- [ ] farmaco-economie
- [ ] anders

C14. Type onderzoek

- ○ observationeel onderzoek zonder invasieve metingen
- ○ observationeel onderzoek met invasieve metingen
- ◉ interventie-onderzoek

C15. In welke fase kan het onderzoek worden ingedeeld?

- ○ fase I (a)
- ○ fase II (b)
- ◉ fase III (c)
- ○ fase IV (d)
- ○ overige onderzoeken waarbij geneesmiddelen worden toegepast (e)
- ○ niet van toepassing

C17. Is er sprake van een andere interventie dan met geneesmiddelen zoals bedoeld in de Wet medisch-wetenschappelijk onderzoek met mensen (WMO) (zie toelichting)?

- ◉ Ja
- ○ Nee

C17a. ☐ Medisch hulpmiddel, namelijk
C17b. ☐ Operatie, namelijk
C17c. ☐ Psychosociale interventie, namelijk

C17d. ☐ Voeding(stoffen), namelijk
C17e. ☐ Bewegingstherapie, namelijk
C17f. ☐ interventie met radioactieve straling, namelijk
C17g. ☐ Blootstellingsonderzoek (bijv pesticidenonderzoek), namelijk
C17h.
C17i. ☑ andere interventie, namelijk

In het Engels:	No sentinel lymph node procedure
In het Nederlands:	Géén schildwachtklierprocedure

C18. Worden de onderzoeksproducten voor deze studie door de verrichter gratis verstrekt?

- ○ ja
- ○ nee
- ○ gedeeltelijk namelijk
- ◉ niet van toepassing

C19. Is/zijn er (een) controlegroep (en)?

- ◉ ja
- ○ nee

- ☐ ja, geneesmiddel
- ☐ ja, placebo
- ☐ ja, geen interventie
- ☑ ja, anders

namelijk De standaard behandeling: schildwachtklierprocedure

C20. Betreft het een gerandomiseerd onderzoek?

- ◉ ja
- ○ nee

namelijk

- ☑ open
- ☐ enkelblind
- ☐ dubbelblind
- ☐ parallel
- ☐ cross-over
- ☐ anders namelijk

C21. Op welke klasse(n) van aandoeningen heeft het onderzoek betrekking (maximaal 3)

- ☐ hartaandoeningen
- ☐ congenitale, familiaire en genetische aandoeningen
- ☐ bloed- en lymfestelsel aandoeningen
- ☐ zenuwstelsel aandoeningen
- ☐ oogaandoeningen
- ☐ evenwichtsorgaan- en ooraandoeningen
- ☐ ademhalingsstelsel-, thorax- en mediastinumaandoeningen
- ☐ maagdarmstelselaandoeningen
- ☐ nier- en urinewegaandoeningen
- ☐ huid- en onderhuidaandoeningen
- ☐ skeletspierstelsel- en bindweefselaandoeningen
- ☐ endocriene aandoeningen
- ☐ voedingsstoornissen en metabole ziekten
- ☐ infecties en parasitaire aandoeningen
- ☐ letsels, intoxicaties en verrichtingscomplicaties
- ☑ neoplasmata, benigne, maligne en niet-gespecificeerd (incl cysten en poliepen)
  borstneoplasmata maligne en niet-gespecificeerd (incl. tepel)

- [x] chirurgische en medische verrichtingen
  borst therapeutische verrichtingen
- [ ] bloedvataandoeningen
- [ ] algemene aandoeningen en aandoeningen op de plek van toediening
- [ ] zwangerschap, perinatale periode en puerperium
- [ ] sociale omstandigheden
- [ ] immuunsysteemaandoeningen
- [ ] lever- en galaandoeningen
- [ ] voortplantingsstelsel- en borstaandoeningen
- [ ] psychische stoornissen
- [ ] overig, namelijk

C22.Geef twee synoniemen voor de aandoening die bestudeerd wordt, waarvan tenminste één lekenterm.

In het Engels:	breast cancer, sentinel lymph nodes, sentinel lymph node metastases
In het Nederlands:	borstkanker, schildwachtklieren, schildwachtkliermetastasen

C23. Beoogde start- en einddatum van het onderzoek

C23a. Start Datum (dd-mm-jjjj)	01-01-2015
C23b. Eind Datum (dd-mm-jjjj)	01-01-2027

C24. Zijn patiënten betrokken bij het ontwikkelen van het onderzoek? (Voor patiënten lees ook: patiëntenorganisaties, naasten of nabestaanden van patiënten en gezonde vrijwilligers)?

C30. Wordt de ervaring van proefpersonen na deelname aan het onderzoek geëvalueerd?

D. Sectie - Proefpersonen

D1. Is er een proefpersonenverzekering conform de WMO-eisen afgesloten of wordt aan de oordelende toetsingscommissie ontheffing gevraagd?

- (●) proefpersonenverzekering is afgesloten bij verzekeringsmaatschappij
- ( ) ontheffing van de verzekering wordt gevraagd
- ( ) niet van toepassing - onderzoek valt onder de Embryowet en niet onder de WMO

Verzekeringsmaatschappij	Marketform (onderdeel van Lloyd's)

D2. Gezonde proefpersonen en/of patiënten

- [ ] Gezonde proefpersonen

- [x] Patiënten

Aantal	1644

D4. Voornaamste inclusiecriteria

D4a. In het Engels

- Female
- Aged 18 years or older
- Pathologically confirmed invasive unilateral breast carcinoma
- A clinical T1-2 tumour (≤ 5cm)
- Will be treated with lumpectomy followed by whole breast radiotherapy
- Clinically node negative status: no signs of axillary lymph node metastases at physical examination and preoperative axillary ultrasound (or negative cyto-/histopathology)
- Written informed consent

D4b. In het Nederlands

- Vrouw
- Leeftijd 18 jaar of ouder
- Pathologisch bewezen invasief unilaterale borstkanker
- Klinisch T1-2 tumor (≤ 5cm)
- Zal worden behandeld met lumpectomie gevolgd door gehele borstbestraling
- Klinisch negatieve okselklierstatus: negatief lichamelijk onderzoek en echografie van de oksel (of negatieve cyto-/histologie)
- Schriftelijke informed consent

D5. Voornaamste exclusiecriteria

D5a. In het Engels

- Clinically node positive pre-operative
- Bilateral breast cancer
- Evidence of metastatic disease

- History of invasive breast cancer
- Previous treatment of the ipsilateral axilla with surgery or radiotherapy, except surgery for hidradenitis suppurativa or for other superficially located skin lesions, such as naevi
- Pregnant or nursing
- Other prior malignancies within the past 5 years, except successfully treated malignancies that occurred more than five years before randomization, and except successfully treated basal cell and squamous cell skin cancer, carcinoma in situ of the cervix or carcinoma in situ of the ipsilateral or contralateral breast
- Unable or unwilling to give informed consent

D5b. In het Nederlands

- Klinisch positieve okselklierstatus (preoperatief)
- Bilaterale borstkanker
- Primair (afstands-)gemetastaseerde borstkanker
- Invasieve borstkanker in de voorgeschiedenis
- Chirurgie of radiotherapie van ipsilaterale oksel in de voorgeschiedenis, behalve chirurgie voor hidradenitis suppurativa of andere oppervlakkig gelegen huidlaesies, zoals naevi
- Zwanger of lacterend
- Andere maligniteit in de afgelopen 5 jaar, behalve succesvol behandelde maligniteit die meer dan vijf jaar voor randomisatie optrad, en behalve succesvol behandelde huidkanker van type basaalcel of plaveiselcel, carcinoma in situ van de cervix, carcinoma in situ van de ipsilaterale of contralaterale mamma
- Niet in staat of niet bereid tot informed consent

D6. Bij welke categorie proefpersonen wordt het onderzoek uitgevoerd (meerdere antwoorden mogelijk)

- [x] 18 jaar of ouder en wilsbekwaam
- [ ] 18 jaar of ouder en wilsonbekwaam
- [ ] 12 t/m 17 jaar en in staat tot het geven van geïnformeerde toestemming
- [ ] 12 t/m 17 jaar en niet in staat tot het geven van geïnformeerde toestemming (wilsonbekwaam)
- [ ] jonger dan 12 jaar

D10. Verkeren (sommige) proefpersonen in een afhankelijkheidssituatie ten opzichte van de onderzoeker of degene die de deelnemers werft? (lees de toelichting voor voorbeelden wanneer er sprake kan zijn van een afhankelijkheidssituatie)

- (•) ja
- ( ) nee

D10a. Zo ja, waardoor?

Soms is de lokale onderzoeker ook de behandelend arts die de patiënt vraagt om deel te nemen aan de studie.

D10b. Waarom wordt het onderzoek juist met deze proefpersonen uitgevoerd en hoe worden de belangen van de proefpersonen gewaarborgd?

Het onderzoek moet met proefpersonen worden uitgevoerd die voldoen aan de beschreven in/exclusie criteria zodat de resultaten van de studie uiteindelijk ook naar de kliniek vertaald kunnen worden binnen deze patiëntenpopulatie. De belangen van de proefpersonen worden gewaarborgd door de geldende medisch ethische normen en richtlijn zoals vermeld in het protocol (o.a. onafhankelijk arts, uitgebreide patiëntinformatiefolder).

D11. Waaruit bestaat de vergoeding voor de proefpersonen?

- [x] geen vergoeding
- [ ] reiskosten
- [ ] financiële vergoeding (in Euro's)
- [ ] andere vergoeding

D12. Is deze vergoeding afhankelijk van bepaalde voorwaarden, bijvoorbeeld het voltooien van (een deel van) het onderzoek?

- ( ) ja (motiveer)
- ( ) nee
- (•) niet van toepassing

E. Sectie - Voor- en nadelen

E1. Wordt er bij dit onderzoek een rechtstreeks therapeutisch effect beoogd bij de proefpersonen / patiënten?

- (•) ja (therapeutisch onderzoek)
- ( ) nee (niet-therapeutisch onderzoek)

E1a. Zo ja, geef kort aan waaruit dit therapeutisch effect bestaat.

Het therapeutische effect bestaat uit:
- Het verlagen van het risico op okselbehandeling-gerelateerde korte en lange termijn complicaties;
- Het verbeteren van de kwaliteit van leven;
- Met een even goede ziektecontrole en algehele overleving.

E2. Waaruit bestaat de belasting van het onderzoek (en een eventueel daaraan voorafgaande keuring) voor de proefpersonen?

Tijdbeslag	per bezoek	20 min per keer voor invullen kwaliteit van leven vragenlijst
	totaal	20 min x 6 = 120 min
	totale duur van de studie voor de individuele proefpersoon	Tav de behandeling minder belasting dan normaal voor de studie-arm (geen schildwachtklierprocedure). Voor beide armen is extra het invullen van de kwaliteit van leven vragenlijst prerandomisatie en postrandomisatie op 5 momenten.

E3. Worden de proefpersonen in verband met het onderzoek in het ziekenhuis opgenomen of wordt een opname verlengd?

○ ja - het verblijf in het ziekenhuis/instituut wordt in verband met het onderzoek verlengd
○ ja - ze worden voor het onderzoek in het ziekenhuis/instituut opgenomen
◉ nee

E4. Beschrijf in hoeverre proefpersonen worden onderworpen aan handelingen dan wel een gedragswijze krijgen opgelegd, zoals vragenlijst, interviews, lichamelijk/psychologisch onderzoek, ontzegging, dieet (voor invasieve ingrepen: zie vraag E6)

In totaal zullen 700 proefpersonen (350 in de experimentele en 350 in de controlegroep) deelnemen aan het kwaliteit van leven onderzoek, bestaande uit een vragenlijst voor de randomisatie en op 5 tijdstippen na de randomisatie (na een half jaar, één, twee, vijf en 10 jaar).

E5. Worden de proefpersonen getest op bepaalde aandoeningen/condities?

○ ja (motiveer)
◉ nee

E6. Welke extra (invasieve) ingrepen (anders dan bij de standaard behandeling) moeten de proefpersonen in het kader van het onderzoek ondergaan:

☑ Niet van toepassing

E9. Geef aan welke risico's er voor proefpersonen zijn verbonden aan deelname aan het onderzoek.

Hoewel de hypothese luidt dat het achterwege laten van de schildwachtklierprocedure geen nadelige invloed heeft op de lokale controle van de ziekte en de overlevingskans, bestaat er toch een kans dat proefpersonen in de experimentele arm een hoger risico hebben op een recidief in de oksel (regionaal recidief) en daardoor tijdens follow-up alsnog een vorm van okselklierbehandeling moeten ondergaan. Uit onderzoek blijkt dat het uitgesteld uitvoeren van okselklierbehandeling (bijvoorbeeld met okselklierdissectie) géén nadelige gevolgen heeft voor de overleving.

E9a. Geef op grond van uw eigen afweging aan waarom het uitvoeren van het onderzoek, in het licht van de belasting en/of risico's die voor proefpersonen aan deelname verbonden zijn, gerechtvaardigd is?

Het mogelijk hogere risico op een recidief in de oksel (regionaal recidief) weegt op tegen de lagere kans op ernstige okselklierbehandeling-gerelateerde morbiditeit (lymfoedeem, bewegingsbeperking in de schouder, arm- en schouderpijn, verminderd gevoel of zenuwpijn) en de verbetering van kwaliteit van leven.

E10. Indien het onderzoek bij minderjarige en/of wilsonbekwame proefpersonen wordt uitgevoerd en geen direct therapeutisch effect wordt beoogd: waarom kunnen belasting en risico's als minimaal worden beschouwd? (Verwijs eventueel naar de relevante pagina's in het protocol.

☑ niet van toepassing

E11. Kan de eventuele therapie na beëindiging van het onderzoek worden voortgezet?

○ ja (motiveer)
○ nee (motiveer)
◉ niet van toepassing

E12. Heeft deelname aan het onderzoek voor de proefpersoon tot gevolg dat van de standaardbehandeling of -diagnostiek kan worden afgeweken of deze kan worden uitgesteld?

◉ ja
○ nee
○ niet van toepassing

E12a. Zo ja, waaruit bestaat de afwijking of het uitstel en waarom is afwijking/uitstel

Proefpersonen in de controlegroep ondergaan de standaard behandeling, namelijk schildwachtklierprocedure volgens de Nederlandse borstkankerrichtlijn. Proefpersonen in de experimentele arm ondergaan primair géén schildwachtklierprocedure. Beide groepen ondergaan dezelfde follow-up middels lichamelijk onderzoek en aanvullend onderzoek op indicatie. Indien een proefpersoon zich in de follow-up periode presenteert met

lymfekliermetastasen, kan alsnog besloten worden om een vorm van okselklierbehandeling uit te voeren. Deze beslissing wordt gemaakt tijdens het multidisciplinair overleg in het desbetreffende centrum waar de patiënt onder behandeling staat.

F. Sectie - Informatie en privacy

F1. Hoe worden de proefpersonen geworven en door wie (onderzoeker, behandelend arts, andere persoon) wordt de proefpersoon/wettelijke vertegenwoordiger geïnformeerd en om toestemming gevraagd?

Op het moment in de behandeling van borstkanker dat de patiënt voldoet aan de in/exclusiecriteria zal de behandeld arts informatie geven over de mogelijkheid tot deelname aan deze studie. Een schriftelijke patiëntinformatiebrief zal worden meegegeven aan de patiënt. De patiënt krijgt ruim de tijd (ten minste één week) om deze informatie thuis na te lezen en een beslissing te nemen. De patiënt kan haar interesse in deelname kenbaar maken aan haar behandelend arts bij een volgend polibezoek. De behandelend arts zal haar dan om toestemming vragen voor deelname aan de studie.

F2. Hoeveel bedenktijd krijgen de proefpersonen/wettelijke vertegenwoordigers om te beslissen over deelname?

Zo lang als nodig, met ten minste één week

F3. Wordt de huisarts, behandelend specialist en/of apotheker van de proefpersoon geïnformeerd over diens deelname aan het onderzoek?

- (●) ja (de proefpersoon dient hiervoor toestemming te geven)
- ( ) nee

F4. Worden persoonsgegevens gecodeerd?

- (●) ja
- ( ) nee

F4a. Zo ja, hoe is deze codering opgebouwd?

Alle proefpersonen krijgen een uniek studienummer. Het unieke studienummer betreft een opvolgnummer zonder verdere specifieke codering voor bijvoorbeeld centrum.

F4b. Wie heeft toegang tot de sleutel van deze code?

De behandelend arts en de onderzoeker

F4c. Wie hebben toegang tot de brondocumenten en eventuele andere tot de persoon herleidbare gegevens?

De lokaal datamanagers en de onderzoeker

F5. Hoe wordt het lichaamsmateriaal gedurende het onderzoek bewaard?

- ( ) in tot de proefpersoon herleidbare vorm (gecodeerd)
- ( ) in niet tot de proefpersoon herleidbare vorm (volledig geanonimiseerd)
- (●) niet van toepassing

F6. Wordt afgenomen lichaamsmateriaal na afloop van het onderzoek vernietigd?

- ( ) ja
- ( ) nee (motiveer)
- (●) niet van toepassing

F7. Kunnen proefpersonen na afloop van het onderzoek opnieuw benaderd worden (bijvoorbeeld voor nader onderzoek of follow-up)?

- ( ) ja
- (●) nee

G. Sectie - Financieel

G1. Door welke geldstroom wordt het onderzoek gefinancierd?

- [ ] eerste geldstroom (Geld van Ministerie van OC&W aan universiteiten)
- [ ] tweede geldstroom (NWO of KNAW), namelijk
- [x] derde geldstroom (anders dan 1e of 2e geldstroom, zoals collectebusfondsen, Europese Unie, vakministeries of bedrijven), namelijk
  KWF Kankerbestrijding

G2. Wordt het onderzoek (mede) gefinancierd door de industrie/bedrijven?

- ( ) ja - door de industrie/bedrijf zoals is opgegeven bij vraag B6/B7 (opdrachtgever van het onderzoek)
- ( ) ja - (ook) door andere industrie/bedrijven dan de opdrachtgever
- (●) nee

G3. Wat is de hoogte van de vergoeding die de arts/onderzoeker cq onderzoeksafdeling/maatschap ontvangt voor de uitvoering van het onderzoek?

○ Per patiënt of proefpersoon
◉ Per deelnemend centrum

bedrag afgerond in hele euro's: € 100,00

G3a. Hoe is de vergoeding opgebouwd?

De vergoeding betreft een financiële compensatie van € 100 excl. BTW als opstartvergoeding voor ieder deelnemend centrum.

G4. Heeft/hebben de onderzoeker(s) gedurende de afgelopen vijf jaar op een of andere wijze een persoonlijke financiële relatie (gehad) met de verrichter/sponsor van het huidige onderzoek?

○ ja (licht toe)
◉ nee

I. Sectie - Indiening en beoordeling

I1. Sla het formulier eerst op en selecteer vervolgens een METC of de CCMO

Commissie
METC NedMec

J. Sectie - Aanvullende opmerkingen

Aanvullende opmerkingen

K. Sectie - Samenvatting

Achtergrond van het onderzoek:

De schildwachtklierprocedure is de standaard methode voor lymfeklierstadiëring in borstkankerpatiënten met een klinisch negatieve okselklierstatus. In de algemene bevolking bevat de schildwachtklier in ongeveer 25% een uitzaaiing. Dit percentage is lager in patiënten met een kleine borsttumor en in borstsparend behandelde patiënten. De ACOSOG-Z0011 studie en de IBCSG 23-01 studie hebben recent aangetoond dat een completerende okselklierdissectie veilig achterwege gelaten kan worden bij borstsparend en met systemische therapie behandelde patiënten met een beperkt aantal schildwachtkliermetastasen. Dit roept de vraag op of de schildwachtklierprocedure nog wel noodzakelijk is voor borstsparend behandelde patiënten met een klinisch negatieve okselklierstatus. De klinisch negatieve okselklierstatus van de patiënten in de beschreven studies werd gesteld op basis van lichamelijk onderzoek. Het toevoegen van een preoperatieve echografie van de oksel zal zorgen voor een betere voorselectie van patiënten zonder lymfekliermetastasen, ten opzichte van lichamelijk onderzoek alleen. Daarbij sluit een negatieve echografie uitgevoerd in Nederland bijna alle patiënten uit met uitgebreide lymfekliermetastasen (≥4 lymfekliermetastasen). Bovendien omvat gehele borstbestraling na de borstsparende operatie in het algemeen het lagere deel van de oksel en behandelt daarmee eventueel achtergebleven uitzaaiingen. Hoewel de schildwachtklierprocedure minder invasief is vergeleken met een okselklierdissectie, komen complicaties nog steeds voor. Zo lijdt 6% van de patiënten die alleen een schildwachtklierprocedure ondergingen aan ernstige lymfoedeem na slechts 3 jaar. Deze studie gaat onderzoeken of de schildwachtklierprocedure veilig achterwege gelaten kan worden bij borstsparend behandelde patiënten, met een negatieve echografie van de oksel.

Doel van het onderzoek:

Het doel van deze studie is het verminderen van overbehandeling van de oksel bij patiënten met borstkanker en een klinisch negatieve okselklierstatus die behandeld worden met borstsparende therapie, door het achterwege laten van de schildwachtklierprocedure, om zodoende de kans op korte en lange termijn complicaties te verminderen en te zorgen voor een verbetering van de kwaliteit van leven met een even goede ziektecontrole en algehele overleving.

Onderzoeksopzet:

Het betreft een gerandomiseerde non-inferiority multicenter onderzoek. Patienten die voldoen aan de in- en exclusiecriteria worden na toestemming middels loting behandeld volgens één van de volgende behandelmethoden:

- Behandelmethode A (controle groep): schildwachtklierprocedure volgens de Nederlandse borstkankerrichtlijn.
- Behandelmethode B (studie groep): geen verdere okselklierstadiëring.

Stratificatiefactoren: leeftijd (≤50, 50≤75, >75 jaar), oestrogeen receptor status (positief vs. negatief), HER2neu status (geamplificeerd vs. niet geamplificeerd), klinisch tumor stadium (<3 cm vs. ≥3 cm), tumorgraad volgens gemodificeerde Bloom-Richardson (graad I-II vs. graad III), is of zal worden behandeld met primaire systemische therapie (ja/nee), participerend centrum.
Overige behandelingen (o.a. hormonale therapie, chemotherapie) worden op indicatie gegeven volgens de Nederlandse borstkankerrichtlijn.
Tijdens de follow-up periode van 10 jaar wordt de oksel nauwkeurig onderzocht middels lichamelijk onderzoek en echografie op indicatie. Indien sprake is van een cyto-/histologisch bewezen lymfekliermetastase tijdens follow-up, dan kan in het multidisciplinair overleg van de desbetreffende patiënt worden besloten tot het uitvoeren van een vorm van okselklierbehandeling.
Gevalideerde vragenlijsten worden verstrekt voor het onderzoeken van de invloed op het aantal complicaties en kwaliteit van leven (Lymph-ICF, QLQ-C30, QLQ-BR23) en om te meten of angst en persoonlijkheidstrekken invloed hebben op de uitkomst van kwaliteit van leven (STAI-trait, NEO-FFI). Deze vragenlijsten worden verstrekt aan 700 van de 1644 patiënten (350 per arm), éénmaal voor de randomisatie en vijfmaal na de randomisatie (6 maanden, 1 jaar, 2 jaar, 5 jaar en 10 jaar). Vragenlijsten met betrekking to kosten-effectiviteit worden éénmaal voor de randomisatie en viermaal na de randomisatie (6 maanden, 1,2 en 3 jaar) afgenomen.

Onderzoekspopulatie:

Vrouwen van 18 jaar of ouder met pathologisch bewezen invasief mammacarcinoom die borstsparende therapie ondergaan.
cT1-2: primaire tumor is bij klinisch onderzoek kleiner dan 5 cm.
cN0: bij lichamelijk onderzoek en echografie van de oksel worden geen aanwijzingen voor lymfekliermetastasen gevonden of de cytologie/histologie na punctie/biopt van een lymfeklier is negatief.
Neoadjuvante systemische therapie is toegestaan indien de patiënt voor de start van de neoadjuvante systemische therapie een klinische T1-2N0 status heeft en waarvoor borstsparende therapie op dat moment al geschikt is.
De patiënt heeft informed consent persoonlijk ondertekend.

Interventie (indien van toepassing):

De controlegroep ondergaat wél de schildwachtklierprocedure volgens de Nederlands borstkankerrichtlijn. De experimentele arm ondergaan géén schildwachtklierprocedure.

Primaire onderzoeksvariabelen/uitkomstmaten:

Primaire uitkomstmaat:
- Regionaal recidief percentage

Secundaire onderzoeksvariabelen/uitkomstmaten (indien van toepassing):
Secundaire uitkomstmaten:
- Distant-disease free survival
- Overall survival
- Later uitgevoerde okselbehandeling
- Lokaal recidief percentage
- Overig-regionaal recidief percentage
- Contralaterale borstkanker
- Percentage (neo)adjuvante systemische therapie
- Kwaliteit van leven en okselbehandeling-gerelateerde morbiditeit
- Kosten-effectiviteit

Omschrijving en inschatting van belasting en risico (indien van toepassing):

Wat betreft belasting is er in deze studie géén sprake van extra diagnostiek of interventies (juist minder) en hoeft de patiënt niet vaker dan normaal voor controle naar het ziekenhuis. De eventuele extra belasting voor de patiënt zal alleen bestaan uit het invullen van kwaliteit van leven vragenlijsten (bij 700 van 1644 patiënten) éénmaal voor de randomisatie en vijfmaal na randomisatie (6 maanden, 1 jaar, 2 jaar, 5 jaar en 10 jaar). De hypothese luidt dat het achterwege laten van de schildwachtklierprocedure even veilig is als het wél uitvoeren van deze procedure. Desondanks is er een risico dat in de experimentele arm meer regionale recidieven optreden en dat bij patiënten op een later tijdstip alsnog een vorm van okselklierbehandeling moet worden uitgevoerd. Daartegenover staat dat er in de experimentele arm door het niet uitvoeren van de schildwachtklierprocedure minder kans is op morbiditeit, minder operaties en opnames in het ziekenhuis en een betere kwaliteit van leven. Patiënten zullen op geen enkele manier vertraging ondervinden als gevolg van deelname aan de studie.

K2.Engelse Samenvatting

Background of the study:
The NSABP B-04 trial revealed that omitting primary axillary treatment of occult positive lymph nodes in clinically node negative breast cancer patients does not affect survival, even after 25 years of follow-up and without the use of adjuvant systemic or radiation therapy. A delayed axillary dissection in case lymph nodes become clinically positive does not affect survival and prevents axillary overtreatment in the majority of patients. The ACOSOG Z0011 and IBCSG 23-01 trials revealed that completion axillary dissection can be safely omitted in clinically node negative patients with metastatic sentinel nodes. Patients randomized for watchful waiting were likely to have residual nodal disease (13-27%) that was not surgically removed. Nevertheless, survival rates were not affected and regional recurrence rates low. Use of axillary ultrasound will improve the preoperative selection of node negative patients, as it selects patients with a more favourable tumour load and accurately excludes advanced nodal disease (≥4 metastatic nodes). Biology and adjuvant systemic and radiation therapy are all factors that most likely diminish the risk that possible metastases left in situ develop into clinically detectable lymph nodes. Patients treated with breast conserving therapy are significantly more often diagnosed with pathologic node negative disease or with micrometastatic disease, compared to patients treated with mastectomy. The risk for occult (macro)metastases in cT1-2N0 breast cancer patients treated with breast conserving therapy is low: about 88% will be node negative and about 95% of node positive patients will have no lymph node metastases beyond the sentinel node. Reflected by the low regional recurrence rate after a (false) negative sentinel node, only a small amount of patients (0.8%) with node positive disease is expected to develop clinically detectable nodal disease.

Objective of the study:
The objective of this study is to decrease the number of breast cancer patients receiving overtreatment of the axilla, in order to positively influence the axillary morbidity rate and quality of life. Therefore, we investigate whether the sentinel lymph node procedure can be safely omitted in clinically node negative breast cancer patients undergoing breast conserving therapy, in terms of non-inferior regional control, distant-disease free and overall survival. All objectives are measured during a follow-up of 10 years.

Study design:
A randomized controlled non-inferiority multicenter study. Patients who meet the in- and exclusioncriteria will be randomized after informed consent for one of the two treatment methods:
- Arm A (control arm): lumpectomy with sentinel lymph node procedure, followed by radiotherapy of the breast with or without completion axillary treatment according to the Dutch breast cancer guideline.
- Arm B (experimental arm): lumpectomy without further axillary staging, followed by radiotherapy of the breast.

Stratification: age (≤50, 50≤75, >75 years old), oestrogen receptor status (positive vs. negative), HER2neu status (amplified vs. not amplified), clinical tumour stage prior to any treatment (<3 cm vs. ≥3 cm), grading (grade I-II vs. III - according to Bloom-Richardson grading system), is or will be treated with primary systemic therapy (yes/no) and participating centre.
Adjuvant systemic treatment is administered if indicated according to the Dutch breast cancer guideline.
Primary systemic therapy is allowed if the patient has a clinical T1-2N0 status (initial stage) that is amenable to lumpectomy pre-systemic therapy.
Yearly follow-up with physical examination of the axilla for 10 years. Axillary ultrasound (+/- tissue sampling) on indication. Staging for distant metastases and/or a delayed axillary lymph node dissection if indicated by the multidisciplinary team. Validated questionnaires are used to assess axillary morbidity rate and quality of life (Lymph-ICF, QLQ-C30, QLQ-BR23) and to measure if anxiety and personality traits influence the outcome of quality of life (STAI-trait, NEO-FFI) in 700 of 878 patients. These questionnaires are provided before randomization and sequentially post randomization at 6 months, 1, 2, 5 and 10 years. Cost-effectiveness questionnaires are provided before randomization and sequentially post randomization at 6 months, 1, 2 and 3 years.

Study population:
Women aged 18 years or older with pathologically confirmed invasive breast carcinoma about to undergo breast conserving surgery followed by radiotherapy of the breast.
cT1-2: primary tumour is smaller than 5 cm.
cN0: no signs of axillary lymph node metastases at physical examination and axillary ultrasound (+/- tissue sampling).
Primary systemic treatment is allowed.
Patient is able to and signed the informed consent form.

Intervention (if applicable):
Patients in the control arm will be treated with the sentinel lymph node procedure according to the Dutch breast cancer guideline.
Patients in the experimental arm will not be treated with the sentinel lymph node procedure.

Primary study parameters/outcome of the study:
Primary endpoint:
- Regional recurrence rate

Secundary study parameters/outcome of the study (if applicable):
Secondary endpoints:
- Distant-disease free survival
- Overall survival
- Delayed axillary treatment
- Local recurrence rate
- Other-regional recurrence rate
- Contralateral breast cancer rate
- Percentage difference in the administration of (neo)adjuvant systemic therapy
- Quality of life and axillary morbidity rate
- Cost-effectiveness

Nature and extent of the burden and risks associated with participation, benefit and group relatedness (if applicable):
There is no extra burden for participating patients as regard to additional diagnostic tests/interventions and follow-up. Any additional burden for the patient will only consist of completing quality of life questionnaires in 700 of 1644 study patients. These questionnaires are provided before randomization and sequentially post randomization at 6 months, 1, 2, 5 and 10 years.
We hypothesize that omitting the sentinel lymph node procedure is not inferior to the standard treatment. Nevertheless, possible disadvantages are a worse regional recurrence rate, and delayed axillary treatment if indicated. Patients in the experimental arm have the possible advantages of less morbidity, fewer operations and hospitalizations and an improved quality of life, compared to patients treated with the sentinel lymph node procedure.
No patient will encounter any delay in their treatment as a result of inclusion.

ONDERTEKENING

De verrichter en indiener verklaren hierbij:

a. het formulier (en samenvatting) volledig en naar waarheid te hebben ingevuld;
b. de antwoorden op de vragen uit het ABR-formulier niet in strijd zijn met het bijbehorende onderzoeksdossier en onderzoekscontract

Naar waarheid getekend, door de verrichter (=opdrachtgever)		door de indiener	
datum	....	datum	....
Handtekening		Handtekening	
naam	U.P. Neumann	naam	J.A. van der Hage
functie	Hoofd afdeling Chirurgie, MUMC+	functie	Chirurg, NKI-AVL

Sluiten Print

Formulier voor medisch-ethische beoordeling en registratie

ABR-formulier, versie december 2015

Onderzoeksdossiernummer

ABR Nummer	**49315**	Status	**Beoordeeld - Positief**
Versie	**10**	Status per	**16-02-2018**
Jaar	**18**		
Dossiernummer	**NL49315.031.14**		
Reden voor	**Wijziging PIs**		

A. Sectie - Openbaar maken gegevens medisch wetenschappelijk onderzoek

A1. Het CCMO-register is een voor ieder toegankelijk openbaar trial register. De antwoorden op de vragen gemarkeerd met een wereldbol en de samenvatting bij dit formulier worden openbaar gemaakt in het CCMO register.

B. Sectie - Administratief

B1. Betreft het onderzoek met geneesmiddelen (inclusief gentherapie, somatische celtherapie, vaccinonderzoek, GGO's, zie verdere toelichting) als bedoeld in de Wet medisch-wetenschappelijk onderzoek met mensen (WMO)?

○ ja

◉ nee

B2. Houdt het onderzoek verband met een eerder door een erkende METC of door de CCMO beoordeelde studie of is het onderzoek reeds eerder bij een erkende METC ter beoordeling voorgelegd?

○ ja, het onderzoek houdt verband met – of is het vervolg op – een eerder beoordeelde studie

○ ja, het onderzoek is eerder ter beoordeling aan een erkende METC of de CCMO voorgelegd (stuur kopie besluit mee)

◉ nee

B4. Is het protocol (nog) in een ander openbaar trial register geregistreerd?

○ ja

◉ nee

B5. Naam indiener/contactpersoon voor de oordelende toetsingscommissie

**B5a.**	Achternaam indiener/contactpersoon	Duijnhoven
	Titel en voorletters	dr. F.
	Tussenvoegsels	van
**B5b.**	Type organisatie/bedrijf	Overige Ziekenhuizen
	Organisatie/bedrijf	Antoni van Leeuwenhoek Ziekenhuis
	Naam organisatie/bedrijf	IKNL trialbureau
	Afdeling	Chirurgie
	Adres	Plesmanlaan 121
	Postcode en plaats	1066 CX Amsterdam
	Land	NL
**B5c.**	Intern adres	afdeling Heelkunde
	Telefoon	020 512 9111
	Fax	020 512 2554
	E-mail	f.v.duijnhoven@nki.nl

B6. Is de indiener werkzaam bij de opdrachtgever/sponsor (verrichter) van het onderzoek?

○ Ja ◉ Nee

B7. Opdrachtgever/sponsor van het onderzoek (verrichter volgens de WMO)

Type Organisatie/Bedrijf	Universitair Medisch Centrum
Organisatie/Bedrijf	Medisch Universitair Ziekenhuis Maastricht

Adres	P. Debyelaan 25
Postcode en plaats	6229 HX Maastricht
Land	NL
Telefoon	0433877477
Fax	0433875473
E-mail	m.smidt@mumc.nl

C. Sectie - Onderzoek

C1. Volledige titel van het onderzoek
C1a. In het Engels
Clinically node negative breast cancer patients undergoing breast conserving therapy: Sentinel lymph node procedure versus follow-up. A Dutch randomized controlled multicentre trial.

C1b. In het Nederlands
Patiënten met borstkanker en een klinisch negatieve okselklierstatus die een borstsparende behandeling ondergaan: Schildwachtklierprocedure versus follow-up. Een Nederlandse gerandomiseerde multicenter studie.

C2. Verkorte titel van het onderzoek/acroniem
C2a. In het Engels
BOOG 2013-08

C2b. In het Nederlands (Let op: deze korte titel wordt vermeld binnen ToetsingOnline)
BOOG 2013-08

C3. Trefwoorden (maximaal 4, plaats elk trefwoord op een aparte regel)
C3a. In het Engels
Breast neoplasms
Breast conserving therapy
Sentinel lymph node biopsy
Axilla

C3b. In het Nederlands
Borstkanker
Borstsparende behandeling
Schildwachtklierprocedure
Oksel

C4. Beschrijf het belang van het onderzoek en de beoogde toepassing van de resultaten (verwijs eventueel naar de relevante pagina's in het protocol).
Het doel van dit onderzoek is om:
1) Overbehandeling van de oksel te verminderen;
2) Het aantal korte en lange termijn complicaties te verminderen;
3) De kwaliteit van leven te verbeteren;
4) Met een even goede ziektecontrole en algehele overleving

Dit willen wij bereiken door te onderzoeken of de schildwachtklierprocedure veilig achterwege gelaten kan worden bij patiënten met borstkanker en een klinisch negatieve okselklierstatus behandeld met borstsparende therapie.

C6. Betreft het onderzoek een multicenter-onderzoek?
- ○ nee
- ◉ ja - alleen in Nederland
- ○ ja - internationaal binnen de Europese Unie
- ○ ja - internationaal ook buiten de Europese Unie

C7. Is er bij multicenter-onderzoek sprake van een coördinerend onderzoeker?
- ◉ ja namelijk
- ○ nee

Naam onderzoeker:
M.L. Smidt

C8. Wie is/zijn medisch verantwoordelijk voor de proefpersonen die deelnemen aan het onderzoek
Behandelend arts en lokale hoofdonderzoeker

C9. In welk centrum/welke centra (incl. huisartsenpraktijken) in Nederland wordt het onderzoek uitgevoerd?

Centrum	Proefpersonen	Hoofdonderzoeker	Onafhankelijk arts
Albert Schweitzer Ziekenhuis	49	M.B.E. Menke-Pluijmers	S.O. Breukink (Maastricht UMC+)
Amphia Ziekenhuis	69	E.J.T. Luiten	S.O. Breukink (Maastricht UMC+)

Antonius Ziekenhuis, Sneek	56	C.G.J.M. van Riel	S.O. Breukink (Maastricht UMC+)
Atrium Medisch Centrum	39	E.R.M. van Haaren	S.O. Breukink (Maastricht UMC+)
Bronovo Ziekenhuis	23	O.R. Guicherit	S.O. Breukink (Maastricht UMC+)
Canisius Wilhelmina Ziekenhuis	46	L.J.A. Strobbe	S.O. Breukink (Maastricht UMC+)
Catharina-ziekenhuis	46	G.A.P. Nieuwenhuijzen	S.O. Breukink (Maastricht UMC+)
Deventer Ziekenhuis	39	H. de Vries	S.O. Breukink (Maastricht UMC+)
Diakonessenhuis Utrecht	39	T. van Dalen	S.O. Breukink (Maastricht UMC+)
Gelre Ziekenhuizen	37	W.B. Lastdrager	S.O. Breukink (Maastricht UMC+)
Groene Hart Ziekenhuis	31	E. Linthorst	S.O. Breukink (Maastricht UMC+)
HagaZiekenhuis	41	I. Jannink	S.O. Breukink (Maastricht UMC+)
Isala Klinieken	54	A.B. Francken	S.O. Breukink (Maastricht UMC+)
Laurentius Ziekenhuis	23	A.V.R.J. Bell	S.O. Breukink (Maastricht UMC+)
Academisch Ziekenhuis Maastricht	39	M.L. Smidt	S.O. Breukink (Maastricht UMC+)
Martini Ziekenhuis	43	W. Kelder	S.O. Breukink (Maastricht UMC+)
Maxima Medisch Centrum	31	K. Schenk	S.O. Breukink (Maastricht UMC+)
Meander Medisch Centrum	43	A.J. van Overbeeke	S.O. Breukink (Maastricht UMC+)
Antoni van Leeuwenhoek Ziekenhuis	93	F. van Duijnhoven	S.O. Breukink (Maastricht UMC+)
Onze Lieve Vrouwe Gasthuis	28	S. Donkervoort	S.O. Breukink (Maastricht UMC+)
Orbis Medisch Centrum	23	Y. Vissers	S.O. Breukink (Maastricht UMC+)
Universitair Medisch Centrum Sint Radboud	31	J.H.W. de Wilt	S.O. Breukink (Maastricht UMC+)
Reinier de Graaf Groep	46	M. van 't Riet	S.O. Breukink (Maastricht UMC+)
Sint Antonius Ziekenhuis	58	R. Koelemij	S.O. Breukink (Maastricht UMC+)
Sint Elisabeth Ziekenhuis	37	P. Jansen	S.O. Breukink (Maastricht UMC+)
Streekziekenhuis Koningin Beatrix	19	A. Jonk	S.O. Breukink (Maastricht UMC+)
Tergooiziekenhuizen locatie Blaricum	42	E.J.C. Vriens	S.O. Breukink (Maastricht UMC+)
Universitair Medisch Centrum Groningen	17	J. de Vries	S.O. Breukink (Maastricht UMC+)
Universitair Medisch Centrum Utrecht	23	A.J. Witkamp	S.O. Breukink (Maastricht UMC+)
Viecuri Medisch Centrum voor Noord-Limburg	39	P.H.A Nijhuis	S.O. Breukink (Maastricht UMC+)
Westfries Gasthuis	27	J.W.D. de Waard	S.O. Breukink (Maastricht UMC+)
Waterland Ziekenhuis	22	L. de Widt	S.O. Breukink (Maastricht UMC+)
Zaans Medisch Centrum	15	S. Muller	S.O. Breukink (Maastricht UMC+)
Ziekenhuisvoorzieningen Gelderse Vallei	32	W.K. de Roos	S.O. Breukink (Maastricht UMC+)
Ziekenhuisgroep Twente	31	D. de Leeuw	S.O. Breukink (Maastricht UMC+)
Rivierenland Ziekenhuis	23	M.F. Sier	S.O. Breukink (Maastricht UMC+)
Noordwest Ziekenhuisgroep	54	K.M. Blaauwendraat	S.O. Breukink (Maastricht UMC+)
Medisch Centrum Haaglanden	31	A. Marinelli	S.O. Breukink (Maastricht UMC+)
Medisch Spectrum Twente	46	P. Steenvoorde	S.O. Breukink (Maastricht UMC+)

Rijnstate Ziekenhuis	62	R. van Eekeren	S.O. Breukink (Maastricht UMC+)
Flevoziekenhuis	50	S. van der Meij	S.O. Breukink (Maastricht UMC+)
Zuwehofpoort	15	R.C. van Doorn	S.O. Breukink (Maastricht UMC+)
Jeroen Bosch Ziekenhuis	20	M. Bessem	B. Biesma (Jeroen Bosch ziekenhuis)
Alrijne ziekenhuis	50	P. Neijenhuis	S.O. Breukink (Maastricht UMC+)
Spaarne Gasthuis	30	B. Kortmann	S.O. Breukink (Maastricht UMC+)
St. Jansdal	40	Schouten van der Velden	S.O. Breukink (Maastricht UMC+)
Treant Zorggroep	50	L. Smit	S.O. Breukink (Maastricht UMC+

C10. Betreft het onderzoek met:

- [x] mensen
- [ ] geslachtscellen
- [ ] (rest-)embryo's
- [ ] foetussen in utero

C11. Beoogd totaal aantal proefpersonen/(rest)embryo's/foetussen in utero:
C11a. In Nederland
1644

C13. Onderzoeksgebied

- [ ] etiologie
- [ ] organisatorisch/zorgonderzoek
- [ ] diagnostiek
- [ ] preventie
- [x] therapie
- [ ] veiligheid
- [ ] werkzaamheid
- [ ] farmacokinetiek
- [ ] farmacodynamiek
- [ ] bio-equivalentie
- [ ] dosis-respons
- [ ] farmacogenomics
- [ ] farmaco-economie
- [ ] anders

C14. Type onderzoek

- ( ) observationeel onderzoek zonder invasieve metingen
- ( ) observationeel onderzoek met invasieve metingen
- (•) interventie-onderzoek

C15. In welke fase kan het onderzoek worden ingedeeld?

- ( ) fase I (a)
- ( ) fase II (b)
- (•) fase III (c)
- ( ) fase IV (d)
- ( ) overige onderzoeken waarbij geneesmiddelen worden toegepast (e)
- ( ) niet van toepassing

C17. Is er sprake van een andere interventie dan met geneesmiddelen zoals bedoeld in de Wet medisch-wetenschappelijk onderzoek met mensen (WMO) (zie toelichting)?

- (•) Ja
- ( ) Nee

C17a. [ ] Medisch hulpmiddel, namelijk

C17b. ☐ Operatie, namelijk
C17c. ☐ Psychosociale interventie, namelijk
C17d. ☐ Voeding(stoffen), namelijk
C17e. ☐ Bewegingstherapie, namelijk
C17f. ☐ interventie met radioactieve straling, namelijk
C17g. ☐ Blootstellingsonderzoek (bijv pesticidenonderzoek), namelijk
C17h.
C17i. ☑ andere interventie, namelijk

In het Engels:	No sentinel lymph node procedure
In het Nederlands:	Géén schildwachtklierprocedure

C18. Worden de onderzoeksproducten voor deze studie door de verrichter gratis verstrekt?

- ○ ja
- ○ nee
- ○ gedeeltelijk namelijk
- ◉ niet van toepassing

C19. Is/zijn er (een) controlegroep (en)?

- ◉ ja
- ○ nee

- ☐ ja, geneesmiddel
- ☐ ja, placebo
- ☐ ja, geen interventie
- ☑ ja, anders

namelijkDe standaard behandeling: schildwachtklierprocedure

C20. Betreft het een gerandomiseerd onderzoek?

- ◉ ja
- ○ nee

namelijk

- ☑ open
- ☐ enkelblind
- ☐ dubbelblind
- ☐ parallel
- ☐ cross-over
- ☐ anders namelijk

C21. Op welke klasse(n) van aandoeningen heeft het onderzoek betrekking (maximaal 3)

- ☐ hartaandoeningen
- ☐ congenitale, familiaire en genetische aandoeningen
- ☐ bloed- en lymfestelsel aandoeningen
- ☐ zenuwstelsel aandoeningen
- ☐ oogaandoeningen
- ☐ evenwichtsorgaan- en ooraandoeningen
- ☐ ademhalingsstelsel-, thorax- en mediastinumaandoeningen
- ☐ maagdarmstelselaandoeningen
- ☐ nier- en urinewegaandoeningen
- ☐ huid- en onderhuidaandoeningen
- ☐ skeletspierstelsel- en bindweefselaandoeningen
- ☐ endocriene aandoeningen
- ☐ voedingsstoomissen en metabole ziekten
- ☐ infecties en parasitaire aandoeningen
- ☐ letsels, intoxicaties en verrichtingscomplicaties

- [x] neoplasmata, benigne, maligne en niet-gespecificeerd (incl cysten en poliepen)
  borstneoplasmata maligne en niet-gespecificeerd (incl. tepel)
- [x] chirurgische en medische verrichtingen
  borst therapeutische verrichtingen
- [ ] bloedvataandoeningen
- [ ] algemene aandoeningen en aandoeningen op de plek van toediening
- [ ] zwangerschap, perinatale periode en puerperium
- [ ] sociale omstandigheden
- [ ] immuunsysteemaandoeningen
- [ ] lever- en galaandoeningen
- [ ] voortplantingsstelsel- en borstaandoeningen
- [ ] psychische stoornissen
- [ ] overig, namelijk

C22.Geef twee synoniemen voor de aandoening die bestudeerd wordt, waarvan tenminste één lekenterm.

In het Engels:	breast cancer, sentinel lymph nodes, sentinel lymph node metastases
In het Nederlands:	borstkanker, schildwachtklieren, schildwachtkliermetastasen

C23. Beoogde start- en einddatum van het onderzoek

C23a. Start Datum (dd-mm-jjjj)	01-01-2015
C23b. Eind Datum (dd-mm-jjjj)	01-01-2027

C24. Zijn patiënten betrokken bij het ontwikkelen van het onderzoek? (Voor patiënten lees ook: patiëntenorganisaties, naasten of nabestaanden van patiënten en gezonde vrijwilligers)?

C30. Wordt de ervaring van proefpersonen na deelname aan het onderzoek geëvalueerd?

D. Sectie - Proefpersonen

D1. Is er een proefpersonenverzekering conform de WMO-eisen afgesloten of wordt aan de oordelende toetsingscommissie ontheffing gevraagd?

- (●) proefpersonenverzekering is afgesloten bij verzekeringsmaatschappij
- ( ) ontheffing van de verzekering wordt gevraagd
- ( ) niet van toepassing - onderzoek valt onder de Embryowet en niet onder de WMO

Verzekeringsmaatschappij	Marketform (onderdeel van Lloyd's)

D2. Gezonde proefpersonen en/of patiënten

- [ ] Gezonde proefpersonen

- [x] Patiënten

Aantal	1644

D4. Voornaamste inclusiecriteria

D4a. In het Engels
- Female
- Aged 18 years or older
- Pathologically confirmed invasive unilateral breast carcinoma
- A clinical T1-2 tumour (≤ 5cm)
- Will be treated with lumpectomy followed by whole breast radiotherapy
- Clinically node negative status: no signs of axillary lymph node metastases at physical examination and preoperative axillary ultrasound (or negative cyto-/histopathology)
- Written informed consent

D4b. In het Nederlands
- Vrouw
- Leeftijd 18 jaar of ouder
- Pathologisch bewezen invasief unilaterale borstkanker
- Klinisch T1-2 tumor (≤ 5cm)
- Zal worden behandeld met lumpectomie gevolgd door gehele borstbestraling
- Klinisch negatieve okselklierstatus: negatief lichamelijk onderzoek en echografie van de oksel (of negatieve cyto-/histologie)
- Schriftelijke informed consent

D5. Voornaamste exclusiecriteria

D5a. In het Engels

- Clinically node positive pre-operative
- Bilateral breast cancer
- Evidence of metastatic disease
- History of invasive breast cancer
- Previous treatment of the ipsilateral axilla with surgery or radiotherapy, except surgery for hidradenitis suppurativa or for other superficially located skin lesions, such as naevi
- Pregnant or nursing
- Other prior malignancies within the past 5 years, except successfully treated malignancies that occurred more than five years before randomization, and except successfully treated basal cell and squamous cell skin cancer, carcinoma in situ of the cervix or carcinoma in situ of the ipsilateral or contralateral breast
- Unable or unwilling to give informed consent

D5b. In het Nederlands

- Klinisch positieve okselklierstatus (preoperatief)
- Bilaterale borstkanker
- Primair (afstands-)gemetastaseerde borstkanker
- Invasieve borstkanker in de voorgeschiedenis
- Chirurgie of radiotherapie van ipsilaterale oksel in de voorgeschiedenis, behalve chirurgie voor hidradenitis suppurativa of andere oppervlakkig gelegen huidlaesies, zoals naevi
- Zwanger of lacterend
- Andere maligniteit in de afgelopen 5 jaar, behalve succesvol behandelde maligniteit die meer dan vijf jaar voor randomisatie optrad, en behalve succesvol behandelde huidkanker van type basaalcel of plaveiselcel, carcinoma in situ van de cervix, carcinoma in situ van de ipsilaterale of contralaterale mamma
- Niet in staat of niet bereid tot informed consent

D6. Bij welke categorie proefpersonen wordt het onderzoek uitgevoerd (meerdere antwoorden mogelijk)

☑ 18 jaar of ouder en wilsbekwaam
☐ 18 jaar of ouder en wilsonbekwaam
☐ 12 t/m 17 jaar en in staat tot het geven van geïnformeerde toestemming
☐ 12 t/m 17 jaar en niet in staat tot het geven van geïnformeerde toestemming (wilsonbekwaam)
☐ jonger dan 12 jaar

D10. Verkeren (sommige) proefpersonen in een afhankelijkheidssituatie ten opzichte van de onderzoeker of degene die de deelnemers werft? (lees de toelichting voor voorbeelden wanneer er sprake kan zijn van een afhankelijkheidssituatie)

◉ ja
○ nee

D10a. Zo ja, waardoor?

Soms is de lokale onderzoeker ook de behandelend arts die de patiënt vraagt om deel te nemen aan de studie.

D10b. Waarom wordt het onderzoek juist met deze proefpersonen uitgevoerd en hoe worden de belangen van de proefpersonen gewaarborgd?

Het onderzoek moet met proefpersonen worden uitgevoerd die voldoen aan de beschreven in/exclusie criteria zodat de resultaten van de studie uiteindelijk ook naar de kliniek vertaald kunnen worden binnen deze patiëntenpopulatie. De belangen van de proefpersonen worden gewaarborgd door de geldende medisch ethische normen en richtlijn zoals vermeld in het protocol (o.a. onafhankelijk arts, uitgebreide patiëntinformatiefolder).

D11. Waaruit bestaat de vergoeding voor de proefpersonen?

☑ geen vergoeding
☐ reiskosten
☐ financiële vergoeding (in Euro's)
☐ andere vergoeding

D12. Is deze vergoeding afhankelijk van bepaalde voorwaarden, bijvoorbeeld het voltooien van (een deel van) het onderzoek?

○ ja (motiveer)
○ nee
◉ niet van toepassing

E. Sectie - Voor- en nadelen

E1. Wordt er bij dit onderzoek een rechtstreeks therapeutisch effect beoogd bij de proefpersonen / patiënten?

◉ ja (therapeutisch onderzoek)
○ nee (niet-therapeutisch onderzoek)

E1a. Zo ja, geef kort aan waaruit dit therapeutisch effect bestaat.

Het therapeutische effect bestaat uit:
- Het verlagen van het risico op okselbehandeling-gerelateerde korte en lange termijn complicaties;

- Het verbeteren van de kwaliteit van leven;
- Met een even goede ziektecontrole en algehele overleving.

E2. Waaruit bestaat de belasting van het onderzoek (en een eventueel daaraan voorafgaande keuring) voor de proefpersonen?

Tijdbeslag	per bezoek	20 min per keer voor invullen kwaliteit van leven vragenlijst
	totaal	20 min x 6 = 120 min
	totale duur van de studie voor de individuele proefpersoon	Tav de behandeling minder belasting dan normaal voor de studie-arm (geen schildwachtklierprocedure). Voor beide armen is extra het invullen van de kwaliteit van leven vragenlijst prerandomisatie en postrandomisatie op 5 momenten.

E3. Worden de proefpersonen in verband met het onderzoek in het ziekenhuis opgenomen of wordt een opname verlengd?

○ ja - het verblijf in het ziekenhuis/instituut wordt in verband met het onderzoek verlengd
○ ja - ze worden voor het onderzoek in het ziekenhuis/instituut opgenomen
◉ nee

E4. Beschrijf in hoeverre proefpersonen worden onderworpen aan handelingen dan wel een gedragswijze krijgen opgelegd, zoals vragenlijst, interviews, lichamelijk/psychologisch onderzoek, ontzegging, dieet (voor invasieve ingrepen: zie vraag E6)

In totaal zullen 700 proefpersonen (350 in de experimentele en 350 in de controlegroep) deelnemen aan het kwaliteit van leven onderzoek, bestaande uit een vragenlijst voor de randomisatie en op 5 tijdstippen na de randomisatie (na een half jaar, één, twee, vijf en 10 jaar).

E5. Worden de proefpersonen getest op bepaalde aandoeningen/condities?

○ ja (motiveer)
◉ nee

E6. Welke extra (invasieve) ingrepen (anders dan bij de standaard behandeling) moeten de proefpersonen in het kader van het onderzoek ondergaan:

☑ Niet van toepassing

E9. Geef aan welke risico's er voor proefpersonen zijn verbonden aan deelname aan het onderzoek.

Hoewel de hypothese luidt dat het achterwege laten van de schildwachtklierprocedure geen nadelige invloed heeft op de lokale controle van de ziekte en de overlevingskans, bestaat er toch een kans dat proefpersonen in de experimentele arm een hoger risico hebben op een recidief in de oksel (regionaal recidief) en daardoor tijdens follow-up alsnog een vorm van okselklierbehandeling moeten ondergaan. Uit onderzoek blijkt dat het uitgesteld uitvoeren van okselklierbehandeling (bijvoorbeeld met okselklierdissectie) géén nadelige gevolgen heeft voor de overleving.

E9a. Geef op grond van uw eigen afweging aan waarom het uitvoeren van het onderzoek, in het licht van de belasting en/of risico's die voor proefpersonen aan deelname verbonden zijn, gerechtvaardigd is?

Het mogelijk hogere risico op een recidief in de oksel (regionaal recidief) weegt op tegen de lagere kans op ernstige okselklierbehandeling-gerelateerde morbiditeit (lymfoedeem, bewegingsbeperking in de schouder, arm- en schouderpijn, verminderd gevoel of zenuwpijn) en de verbetering van kwaliteit van leven.

E10. Indien het onderzoek bij minderjarige en/of wilsonbekwame proefpersonen wordt uitgevoerd en geen direct therapeutisch effect wordt beoogd: waarom kunnen belasting en risico's als minimaal worden beschouwd? (Verwijs eventueel naar de relevante pagina's in het protocol.

☑ niet van toepassing

E11. Kan de eventuele therapie na beëindiging van het onderzoek worden voortgezet?

○ ja (motiveer)
○ nee (motiveer)
◉ niet van toepassing

E12. Heeft deelname aan het onderzoek voor de proefpersoon tot gevolg dat van de standaardbehandeling of -diagnostiek kan worden afgeweken of deze kan worden uitgesteld?

◉ ja
○ nee
○ niet van toepassing

E12a. Zo ja, waaruit bestaat de afwijking of het uitstel en waarom is afwijking/uitstel

Proefpersonen in de controlegroep ondergaan de standaard behandeling, namelijk schildwachtklierprocedure volgens de Nederlandse borstkankerrichtlijn. Proefpersonen in de experimentele arm ondergaan primair géén schildwachtklierprocedure. Beide groepen ondergaan dezelfde follow-up middels lichamelijk onderzoek en aanvullend onderzoek op indicatie. Indien een proefpersoon zich in de follow-up periode presenteert met lymfekliermetastasen, kan alsnog besloten worden om een vorm van okselklierbehandeling uit te voeren. Deze beslissing wordt gemaakt tijdens het multidisciplinair overleg in het desbetreffende centrum waar de patiënt onder behandeling staat.

F. Sectie - Informatie en privacy

F1. Hoe worden de proefpersonen geworven en door wie (onderzoeker, behandelend arts, andere persoon) wordt de proefpersoon/wettelijke vertegenwoordiger geïnformeerd en om toestemming gevraagd?

Op het moment in de behandeling van borstkanker dat de patiënt voldoet aan de in/exclusiecriteria zal de behandeld arts informatie geven over de mogelijkheid tot deelname aan deze studie. Een schriftelijke patiëntinformatiebrief zal worden meegegeven aan de patiënt. De patiënt krijgt ruim de tijd (ten minste één week) om deze informatie thuis na te lezen en een beslissing te nemen. De patiënt kan haar interesse in deelname kenbaar maken aan haar behandelend arts bij een volgend polibezoek. De behandelend arts zal haar dan om toestemming vragen voor deelname aan de studie.

F2. Hoeveel bedenktijd krijgen de proefpersonen/wettelijke vertegenwoordigers om te beslissen over deelname?

Zo lang als nodig, met ten minste één week

F3. Wordt de huisarts, behandelend specialist en/of apotheker van de proefpersoon geïnformeerd over diens deelname aan het onderzoek?

- (●) ja (de proefpersoon dient hiervoor toestemming te geven)
- ( ) nee

F4. Worden persoonsgegevens gecodeerd?

- (●) ja
- ( ) nee

F4a. Zo ja, hoe is deze codering opgebouwd?

Alle proefpersonen krijgen een uniek studienummer. Het unieke studienummer betreft een opvolgnummer zonder verdere specifieke codering voor bijvoorbeeld centrum.

F4b. Wie heeft toegang tot de sleutel van deze code?

De behandelend arts en de onderzoeker

F4c. Wie hebben toegang tot de brondocumenten en eventuele andere tot de persoon herleidbare gegevens?

De lokaal datamanagers en de onderzoeker

F5. Hoe wordt het lichaamsmateriaal <u>gedurende</u> het onderzoek bewaard?

- ( ) in tot de proefpersoon herleidbare vorm (gecodeerd)
- ( ) in niet tot de proefpersoon herleidbare vorm (volledig geanonimiseerd)
- (●) niet van toepassing

F6. Wordt afgenomen lichaamsmateriaal na afloop van het onderzoek vernietigd?

- ( ) ja
- ( ) nee (motiveer)
- (●) niet van toepassing

F7. Kunnen proefpersonen na afloop van het onderzoek opnieuw benaderd worden (bijvoorbeeld voor nader onderzoek of follow-up)?

- ( ) ja
- (●) nee

G. Sectie - Financieel

G1. Door welke geldstroom wordt het onderzoek gefinancierd?

- [ ] eerste geldstroom (Geld van Ministerie van OC&W aan universiteiten)
- [ ] tweede geldstroom (NWO of KNAW), namelijk

- [x] derde geldstroom (anders dan 1e of 2e geldstroom, zoals collectebusfondsen, Europese Unie, vakministeries of bedrijven), namelijk
KWF Kankerbestrijding

G2. Wordt het onderzoek (mede) gefinancierd door de industrie/bedrijven?

○ ja - door de industrie/bedrijf zoals is opgegeven bij vraag B6/B7 (opdrachtgever van het onderzoek)
○ ja - (ook) door andere industrie/bedrijven dan de opdrachtgever
◉ nee

G3. Wat is de hoogte van de vergoeding die de arts/onderzoeker cq onderzoeksafdeling/maatschap ontvangt voor de uitvoering van het onderzoek?

○ Per patiënt of proefpersoon
◉ Per deelnemend centrum

bedrag afgerond in hele euro's: € 100,00

G3a. Hoe is de vergoeding opgebouwd?

De vergoeding betreft een financiële compensatie van € 100 excl. BTW als opstartvergoeding voor ieder deelnemend centrum.

G4. Heeft/hebben de onderzoeker(s) gedurende de afgelopen vijf jaar op een of andere wijze een persoonlijke financiële relatie (gehad) met de verrichter/sponsor van het huidige onderzoek?

○ ja (licht toe)
◉ nee

I. Sectie - Indiening en beoordeling

I1. Sla het formulier eerst op en selecteer vervolgens een METC of de CCMO

Commissie
METC NedMec

J. Sectie - Aanvullende opmerkingen

Aanvullende opmerkingen

K. Sectie - Samenvatting

Achtergrond van het onderzoek:

De schildwachtklierprocedure is de standaard methode voor lymfeklierstadiëring in borstkankerpatiënten met een klinisch negatieve okselklierstatus. In de algemene bevolking bevat de schildwachtklier in ongeveer 25% een uitzaaiing. Dit percentage is lager in patiënten met een kleine borsttumor en in borstsparend behandelde patiënten. De ACOSOG-Z0011 studie en de IBCSG 23-01 studie hebben recent aangetoond dat een completerende okselklierdissectie veilig achterwege gelaten kan worden bij borstsparend en met systemische therapie behandelde patiënten met een beperkt aantal schildwachtkliermetastasen. Dit roept de vraag op of de schildwachtklierprocedure nog wel noodzakelijk is voor borstsparend behandelde patiënten met een klinisch negatieve okselklierstatus. De klinisch negatieve okselklierstatus van de patiënten in de beschreven studies werd gesteld op basis van lichamelijk onderzoek. Het toevoegen van een preoperatieve echografie van de oksel zal zorgen voor een betere voorselectie van patiënten zonder lymfekliermetastasen, ten opzichte van lichamelijk onderzoek alleen. Daarbij sluit een negatieve echografie uitgevoerd in Nederland bijna alle patiënten uit met uitgebreide lymfekliermetastasen (≥4 lymfekliermetastasen). Bovendien omvat gehele borstbestraling na de borstsparende operatie in het algemeen het lagere deel van de oksel en behandelt daarmee eventueel achtergebleven uitzaaiingen. Hoewel de schildwachtklierprocedure minder invasief is vergeleken met een okselklierdissectie, komen complicaties nog steeds voor. Zo lijdt 6% van de patiënten die alleen een schildwachtklierprocedure ondergingen aan ernstige lymfoedeem na slechts 3 jaar. Deze studie gaat onderzoeken of de schildwachtklierprocedure veilig achterwege gelaten kan worden bij borstsparend behandelde patiënten, met een negatieve echografie van de oksel.

Doel van het onderzoek:

Het doel van deze studie is het verminderen van overbehandeling van de oksel bij patiënten met borstkanker en een klinisch negatieve okselklierstatus die behandeld worden met borstsparende therapie, door het achterwege laten van de schildwachtklierprocedure, om zodoende de kans op korte en lange termijn complicaties te verminderen en te zorgen voor een verbetering van de kwaliteit van leven met een even goede ziektecontrole en algehele overleving.

Onderzoeksopzet:

Het betreft een gerandomiseerde non-inferiority multicenter onderzoek. Patienten die voldoen aan de in- en exclusiecriteria worden na toestemming middels loting behandeld volgens één van de volgende behandelmethoden:

- Behandelmethode A (controle groep): schildwachtklierprocedure volgens de Nederlandse borstkankerrichtlijn.
- Behandelmethode B (studie groep): geen verdere okselklierstadiëring.

Stratificatiefactoren: leeftijd (≤50, 50≤75, >75 jaar), oestrogeen receptor status (positief vs. negatief), HER2neu status (geamplificeerd vs. niet geamplificeerd), klinisch tumor stadium (<3 cm vs. ≥3 cm), tumorgraad volgens gemodificeerde Bloom-Richardson (graad I-II vs. graad III), is of zal worden behandeld met primaire systemische therapie (ja/nee), participerend centrum. Overige behandelingen (o.a. hormonale therapie, chemotherapie) worden op indicatie gegeven volgens de Nederlandse borstkankerrichtlijn.
Tijdens de follow-up periode van 10 jaar wordt de oksel nauwkeurig onderzocht middels lichamelijk onderzoek en echografie op indicatie. Indien sprake is van een cyto-/histologisch bewezen lymfekliermetastase tijdens follow-up, dan kan in het multidisciplinair overleg van de desbetreffende patiënt worden besloten tot het uitvoeren van een vorm van okselklierbehandeling.
Gevalideerde vragenlijsten worden verstrekt voor het onderzoeken van de invloed op het aantal complicaties en kwaliteit van leven (Lymph-ICF, QLQ-C30, QLQ-BR23) en om te meten of angst en persoonlijkheidstrekken invloed hebben op de uitkomst van kwaliteit van leven (STAI-trait, NEO-FFI). Deze vragenlijsten worden verstrekt aan 700 van de 1644 patiënten (350 per arm),

éénmaal voor de randomisatie en vijfmaal na de randomisatie (6 maanden, 1 jaar, 2 jaar, 5 jaar en 10 jaar). Vragenlijsten met betrekking to kosten-effectiviteit worden éénmaal voor de randomisatie en viermaal na de randomisatie (6 maanden, 1,2 en 3 jaar) afgenomen.

Onderzoekspopulatie:

Vrouwen van 18 jaar of ouder met pathologisch bewezen invasief mammacarcinoom die borstsparende therapie ondergaan.
cT1-2: primaire tumor is bij klinisch onderzoek kleiner dan 5 cm.
cN0: bij lichamelijk onderzoek en echografie van de oksel worden geen aanwijzingen voor lymfekliermetastasen gevonden of de cytologie/histologie na punctie/biopt van een lymfeklier is negatief.
Neoadjuvante systemische therapie is toegestaan indien de patiënt voor de start van de neoadjuvante systemische therapie een klinische T1-2N0 status heeft en waarvoor borstsparende therapie op dat moment al geschikt is.
De patiënt heeft informed consent persoonlijk ondertekend.

Interventie (indien van toepassing):

De controlegroep ondergaat wél de schildwachtklierprocedure volgens de Nederlands borstkankerrichtlijn. De experimentele arm ondergaan géén schildwachtklierprocedure.

Primaire onderzoeksvariabelen/uitkomstmaten:

Primaire uitkomstmaat:
- Regionaal recidief percentage

Secundaire onderzoeksvariabelen/uitkomstmaten (indien van toepassing):
Secundaire uitkomstmaten:
- Distant-disease free survival
- Overall survival
- Later uitgevoerde okselbehandeling
- Lokaal recidief percentage
- Overig-regionaal recidief percentage
- Contralaterale borstkanker
- Percentage (neo)adjuvante systemische therapie
- Kwaliteit van leven en okselbehandeling-gerelateerde morbiditeit
- Kosten-effectiviteit

Omschrijving en inschatting van belasting en risico (indien van toepassing):

Wat betreft belasting is er in deze studie géén sprake van extra diagnostiek of interventies (juist minder) en hoeft de patiënt niet vaker dan normaal voor controle naar het ziekenhuis. De eventuele extra belasting voor de patiënt zal alleen bestaan uit het invullen van kwaliteit van leven vragenlijsten (bij 700 van 1644 patiënten) éénmaal voor de randomisatie en vijfmaal na randomisatie (6 maanden, 1 jaar, 2 jaar, 5 jaar en 10 jaar). De hypothese luidt dat het achterwege laten van de schildwachtklierprocedure even veilig is als het wél uitvoeren van deze procedure. Desondanks is er een risico dat in de experimentele arm meer regionale recidieven optreden en dat bij patiënten op een later tijdstip alsnog een vorm van okselklierbehandeling moet worden uitgevoerd. Daartegenover staat dat er in de experimentele arm door het niet uitvoeren van de schildwachtklierprocedure minder kans is op morbiditeit, minder operaties en opnames in het ziekenhuis en een betere kwaliteit van leven. Patiënten zullen op geen enkele manier vertraging ondervinden als gevolg van deelname aan de studie.

K2.Engelse Samenvatting

Background of the study:
The NSABP B-04 trial revealed that omitting primary axillary treatment of occult positive lymph nodes in clinically node negative breast cancer patients does not affect survival, even after 25 years of follow-up and without the use of adjuvant systemic or radiation therapy. A delayed axillary dissection in case lymph nodes become clinically positive does not affect survival and prevents axillary overtreatment in the majority of patients. The ACOSOG Z0011 and IBCSG 23-01 trials revealed that completion axillary dissection can be safely omitted in clinically node negative patients with metastatic sentinel nodes. Patients randomized for watchful waiting were likely to have residual nodal disease (13-27%) that was not surgically removed. Nevertheless, survival rates were not affected and regional recurrence rates low. Use of axillary ultrasound will improve the preoperative selection of node negative patients, as it selects patients with a more favourable tumour load and accurately excludes advanced nodal disease (≥4 metastatic nodes). Biology and adjuvant systemic and radiation therapy are all factors that most likely diminish the risk that possible metastases left in situ develop into clinically detectable lymph nodes. Patients treated with breast conserving therapy are significantly more often diagnosed with pathologic node negative disease or with micrometastatic disease, compared to patients treated with mastectomy. The risk for occult (macro)metastases in cT1-2N0 breast cancer patients treated with breast conserving therapy is low: about 88% will be node negative and about 95% of node positive patients will have no lymph node metastases beyond the sentinel node. Reflected by the low regional recurrence rate after a (false) negative sentinel node, only a small amount of patients (0.8%) with node positive disease is expected to develop clinically detectable nodal disease.

Objective of the study:
The objective of this study is to decrease the number of breast cancer patients receiving overtreatment of the axilla, in order to positively influence the axillary morbidity rate and quality of life. Therefore, we investigate whether the sentinel lymph node procedure can be safely omitted in clinically node negative breast cancer patients undergoing breast conserving therapy, in terms of non-inferior regional control, distant-disease free and overall survival. All objectives are measured during a follow-up of 10 years.

Study design:
A randomized controlled non-inferiority multicenter study. Patients who meet the in- and exclusioncriteria will be randomized after informed consent for one of the two treatment methods:

- Arm A (control arm): lumpectomy with sentinel lymph node procedure, followed by radiotherapy of the breast with or without completion axillary treatment according to the Dutch breast cancer guideline.
- Arm B (experimental arm): lumpectomy without further axillary staging, followed by radiotherapy of the breast.
Stratification: age (≤50, 50≤75, >75 years old), oestrogen receptor status (positive vs. negative), HER2neu status (amplified vs. not amplified), clinical tumour stage prior to any treatment (<3 cm vs. ≥3 cm), grading (grade I-II vs. III - according to Bloom-Richardson grading system), is or will be treated with primary systemic therapy (yes/no) and participating centre.
Adjuvant systemic treatment is administered if indicated according to the Dutch breast cancer guideline.
Primary systemic therapy is allowed if the patient has a clinical T1-2N0 status (initial stage) that is amenable to lumpectomy pre-systemic therapy.
Yearly follow-up with physical examination of the axilla for 10 years. Axillary ultrasound (+/- tissue sampling) on indication. Staging for distant metastases and/or a delayed axillary lymph node dissection if indicated by the multidisciplinary team. Validated questionnaires are used to assess axillary morbidity rate and quality of life (Lymph-ICF, QLQ-C30, QLQ-BR23) and to measure if anxiety and personality traits influence the outcome of quality of life (STAI-trait, NEO-FFI) in 700 of 878 patients. These questionnaires are provided before randomization and sequentially post randomization at 6 months, 1, 2, 5 and 10 years. Cost-effectiveness questionnaires are provided before randomization and sequentially post randomization at 6 months, 1, 2 and 3 years.

Study population:
Women aged 18 years or older with pathologically confirmed invasive breast carcinoma about to undergo breast conserving surgery followed by radiotherapy of the breast.
cT1-2: primary tumour is smaller than 5 cm.
cN0: no signs of axillary lymph node metastases at physical examination and axillary ultrasound (+/- tissue sampling).
Primary systemic treatment is allowed.
Patient is able to and signed the informed consent form.

Intervention (if applicable):
Patients in the control arm will be treated with the sentinel lymph node procedure according to the Dutch breast cancer guideline.
Patients in the experimental arm will not be treated with the sentinel lymph node procedure.

Primary study parameters/outcome of the study:
Primary endpoint:
- Regional recurrence rate

Secundary study parameters/outcome of the study (if applicable):
Secondary endpoints:
- Distant-disease free survival
- Overall survival
- Delayed axillary treatment
- Local recurrence rate
- Other-regional recurrence rate
- Contralateral breast cancer rate
- Percentage difference in the administration of (neo)adjuvant systemic therapy
- Quality of life and axillary morbidity rate
- Cost-effectiveness

Nature and extent of the burden and risks associated with participation, benefit and group relatedness (if applicable):
There is no extra burden for participating patients as regard to additional diagnostic tests/interventions and follow-up. Any additional burden for the patient will only consist of completing quality of life questionnaires in 700 of 1644 study patients. These questionnaires are provided before randomization and sequentially post randomization at 6 months, 1, 2, 5 and 10 years.
We hypothesize that omitting the sentinel lymph node procedure is not inferior to the standard treatment. Nevertheless, possible disadvantages are a worse regional recurrence rate, and delayed axillary treatment if indicated. Patients in the experimental arm have the possible advantages of less morbidity, fewer operations and hospitalizations and an improved quality of life, compared to patients treated with the sentinel lymph node procedure.
No patient will encounter any delay in their treatment as a result of inclusion.

ONDERTEKENING

De verrichter en indiener verklaren hierbij:
a. het formulier (en samenvatting) volledig en naar waarheid te hebben ingevuld;
b. de antwoorden op de vragen uit het ABR-formulier niet in strijd zijn met het bijbehorende onderzoeksdossier en onderzoekscontract

Naar waarheid getekend, door de verrichter (=opdrachtgever)	door de indiener
datum	datum ....
Handtekening	Handtekening

naam	U.P. Neumann	naam	F. van Duijnhoven
functie	Hoofd afdeling Chirurgie, MUMC+	functie	Chirurg, NKI-AVL

Sluiten Print

Sluiten Print

Formulier voor medisch-ethische beoordeling en registratie

ABR-formulier, versie december 2015

Onderzoeksdossiernummer

ABR Nummer	**49315**	Status	**Beoordeeld - Positief**
Versie	**11**	Status per	**29-10-2018**
Jaar	**18**		
Dossiernummer	**NL49315.031.14**		
Reden voor	**Toevoeging 2 centra en tekstuele wijzigingen.**		

A. Sectie - Openbaar maken gegevens medisch wetenschappelijk onderzoek

A1. Het CCMO-register is een voor ieder toegankelijk openbaar trial register. De antwoorden op de vragen gemarkeerd met een wereldbol en de samenvatting bij dit formulier worden openbaar gemaakt in het CCMO register.

B. Sectie - Administratief

B1. Betreft het onderzoek met geneesmiddelen (inclusief gentherapie, somatische celtherapie, vaccinonderzoek, GGO's, zie verdere toelichting) als bedoeld in de Wet medisch-wetenschappelijk onderzoek met mensen (WMO)?

○ ja
◉ nee

B2. Houdt het onderzoek verband met een eerder door een erkende METC of door de CCMO beoordeelde studie of is het onderzoek reeds eerder bij een erkende METC ter beoordeling voorgelegd?

○ ja, het onderzoek houdt verband met – of is het vervolg op – een eerder beoordeelde studie
○ ja, het onderzoek is eerder ter beoordeling aan een erkende METC of de CCMO voorgelegd (stuur kopie besluit mee)
◉ nee

B4. Is het protocol (nog) in een ander openbaar trial register geregistreerd?

○ ja
◉ nee

B5. Naam indiener/contactpersoon voor de oordelende toetsingscommissie

**B5a.**	Achternaam indiener/contactpersoon	Duijnhoven
	Titel en voorletters	dr. F.
	Tussenvoegsels	van
**B5b.**	Type organisatie/bedrijf	Overige Ziekenhuizen
	Organisatie/bedrijf	Antoni van Leeuwenhoek Ziekenhuis
	Naam organisatie/bedrijf	IKNL trialbureau
	Afdeling	Chirurgie
	Adres	Plesmanlaan 121
	Postcode en plaats	1066 CX Amsterdam
	Land	NL
**B5c.**	Intern adres	afdeling Heelkunde
	Telefoon	020 512 9111
	Fax	020 512 2554
	E-mail	f.v.duijnhoven@nki.nl

B6. Is de indiener werkzaam bij de opdrachtgever/sponsor (verrichter) van het onderzoek?

○ Ja ◉ Nee

B7. Opdrachtgever/sponsor van het onderzoek (verrichter volgens de WMO)

Type Organisatie/Bedrijf	Universitair Medisch Centrum
Organisatie/Bedrijf	Medisch Universitair Ziekenhuis Maastricht

Adres	P. Debyelaan 25
Postcode en plaats	6229 HX Maastricht
Land	NL
Telefoon	0433877477
Fax	0433875473
E-mail	m.smidt@mumc.nl

C. Sectie - Onderzoek

C1. Volledige titel van het onderzoek

C1a. In het Engels

Clinically node negative breast cancer patients undergoing breast conserving therapy: Sentinel lymph node procedure versus follow-up. A Dutch randomized controlled multicentre trial.

C1b. In het Nederlands

Patiënten met borstkanker en een klinisch negatieve okselklierstatus die een borstsparende behandeling ondergaan: Schildwachtklierprocedure versus follow-up. Een Nederlandse gerandomiseerde multicenter studie.

C2. Verkorte titel van het onderzoek/acroniem

C2a. In het Engels

BOOG 2013-08

C2b. In het Nederlands (Let op: deze korte titel wordt vermeld binnen ToetsingOnline)

BOOG 2013-08

C3. Trefwoorden (maximaal 4, plaats elk trefwoord op een aparte regel)

C3a. In het Engels

Breast neoplasms
Breast conserving therapy
Sentinel lymph node biopsy
Axilla

C3b. In het Nederlands

Borstkanker
Borstsparende behandeling
Schildwachtklierprocedure
Oksel

C4. Beschrijf het belang van het onderzoek en de beoogde toepassing van de resultaten (verwijs eventueel naar de relevante pagina's in het protocol).

Het doel van dit onderzoek is om:
1) Overbehandeling van de oksel te verminderen;
2) Het aantal korte en lange termijn complicaties te verminderen;
3) De kwaliteit van leven te verbeteren;
4) Met een even goede ziektecontrole en algehele overleving

Dit willen wij bereiken door te onderzoeken of de schildwachtklierprocedure veilig achterwege gelaten kan worden bij patiënten met borstkanker en een klinisch negatieve okselklierstatus behandeld met borstsparende therapie.

C6. Betreft het onderzoek een multicenter-onderzoek?

○ nee
◉ ja - alleen in Nederland
○ ja - internationaal binnen de Europese Unie
○ ja - internationaal ook buiten de Europese Unie

C7. Is er bij multicenter-onderzoek sprake van een coördinerend onderzoeker?

◉ ja namelijk — Naam onderzoeker: M.L. Smidt
○ nee

C8. Wie is/zijn medisch verantwoordelijk voor de proefpersonen die deelnemen aan het onderzoek

Behandelend arts en lokale hoofdonderzoeker

C9. In welk centrum/welke centra (incl. huisartsenpraktijken) in Nederland wordt het onderzoek uitgevoerd?

Centrum	Proefpersonen	Hoofdonderzoeker	Onafhankelijk arts
Albert Schweitzer Ziekenhuis	49	M.B.E. Menke-Pluijmers	S.O. Breukink (Maastricht UMC+)
Amphia Ziekenhuis	69	E.J.T. Luiten	S.O. Breukink (Maastricht UMC+)

Antonius Ziekenhuis, Sneek	56	C.G.J.M. van Riel	S.O. Breukink (Maastricht UMC+)
Atrium Medisch Centrum	39	E.R.M. van Haaren	S.O. Breukink (Maastricht UMC+)
Bronovo Ziekenhuis	23	O.R. Guicherit	S.O. Breukink (Maastricht UMC+)
Canisius Wilhelmina Ziekenhuis	46	L.J.A. Strobbe	S.O. Breukink (Maastricht UMC+)
Catharina-ziekenhuis	46	G.A.P. Nieuwenhuijzen	S.O. Breukink (Maastricht UMC+)
Deventer Ziekenhuis	39	H. de Vries	S.O. Breukink (Maastricht UMC+)
Diakonessenhuis Utrecht	39	T. van Dalen	S.O. Breukink (Maastricht UMC+)
Gelre Ziekenhuizen	37	W.B. Lastdrager	S.O. Breukink (Maastricht UMC+)
Groene Hart Ziekenhuis	31	E. Linthorst	S.O. Breukink (Maastricht UMC+)
HagaZiekenhuis	41	I. Jannink	S.O. Breukink (Maastricht UMC+)
Isala Klinieken	54	A.B. Francken	S.O. Breukink (Maastricht UMC+)
Laurentius Ziekenhuis	23	A.V.R.J. Bell	S.O. Breukink (Maastricht UMC+)
Academisch Ziekenhuis Maastricht	39	M.L. Smidt	S.O. Breukink (Maastricht UMC+)
Martini Ziekenhuis	43	W. Kelder	S.O. Breukink (Maastricht UMC+)
Maxima Medisch Centrum	31	K. Schenk	S.O. Breukink (Maastricht UMC+)
Meander Medisch Centrum	43	A.J. van Overbeeke	S.O. Breukink (Maastricht UMC+)
Antoni van Leeuwenhoek Ziekenhuis	93	F. van Duijnhoven	S.O. Breukink (Maastricht UMC+)
Onze Lieve Vrouwe Gasthuis	28	S. Donkervoort	S.O. Breukink (Maastricht UMC+)
Orbis Medisch Centrum	23	Y. Vissers	S.O. Breukink (Maastricht UMC+)
Universitair Medisch Centrum Sint Radboud	31	J.H.W. de Wilt	S.O. Breukink (Maastricht UMC+)
Reinier de Graaf Groep	46	M. van 't Riet	S.O. Breukink (Maastricht UMC+)
Sint Antonius Ziekenhuis	58	R. Koelemij	S.O. Breukink (Maastricht UMC+)
Sint Elisabeth Ziekenhuis	37	P. Jansen	S.O. Breukink (Maastricht UMC+)
Streekziekenhuis Koningin Beatrix	19	A. Jonk	S.O. Breukink (Maastricht UMC+)
Tergooiziekenhuizen locatie Blaricum	42	E.J.C. Vriens	S.O. Breukink (Maastricht UMC+)
Universitair Medisch Centrum Groningen	17	J. de Vries	S.O. Breukink (Maastricht UMC+)
Universitair Medisch Centrum Utrecht	23	A.J. Witkamp	S.O. Breukink (Maastricht UMC+)
Viecuri Medisch Centrum voor Noord-Limburg	39	P.H.A Nijhuis	S.O. Breukink (Maastricht UMC+)
Westfries Gasthuis	27	J.W.D. de Waard	S.O. Breukink (Maastricht UMC+)
Waterland Ziekenhuis	22	L. de Widt	S.O. Breukink (Maastricht UMC+)
Zaans Medisch Centrum	15	S. Muller	S.O. Breukink (Maastricht UMC+)
Ziekenhuisvoorzieningen Gelderse Vallei	32	W.K. de Roos	S.O. Breukink (Maastricht UMC+)
Ziekenhuisgroep Twente	31	D. de Leeuw	S.O. Breukink (Maastricht UMC+)
Rivierenland Ziekenhuis	23	M.F. Sier	S.O. Breukink (Maastricht UMC+)
Noordwest Ziekenhuisgroep	54	K.M. Blaauwendraat	S.O. Breukink (Maastricht UMC+)
Medisch Centrum Haaglanden	31	A. Marinelli	S.O. Breukink (Maastricht UMC+)
Medisch Spectrum Twente	46	P. Steenvoorde	S.O. Breukink (Maastricht UMC+)

Rijnstate Ziekenhuis	62	R. van Eekeren	S.O. Breukink (Maastricht UMC+)
Flevoziekenhuis	50	S. van der Meij	S.O. Breukink (Maastricht UMC+)
Zuwehofpoort	15	R.C. van Doorn	S.O. Breukink (Maastricht UMC+)
Jeroen Bosch Ziekenhuis	20	M. Bessem	B. Biesma (Jeroen Bosch ziekenhuis)
Alrijne ziekenhuis	50	P. Neijenhuis	S.O. Breukink (Maastricht UMC+)
Spaarne Gasthuis	30	B. Kortmann	S.O. Breukink (Maastricht UMC+)
St. Jansdal	40	Schouten van der Velden	S.O. Breukink (Maastricht UMC+)
Treant Zorggroep	50	L. Smit	S.O. Breukink (Maastricht UMC+
Ommelander Zorggroep	20	Drs. T. van Sprundel	Dr. S. Breukink
Zuyderland Medisch Centrum	30	Drs. E.R.M. van Haaren	Dr. S. Breukink

C10. Betreft het onderzoek met:

- ☑ mensen
- ☐ geslachtscellen
- ☐ (rest-)embryo's
- ☐ foetussen in utero

C11. Beoogd totaal aantal proefpersonen/(rest)embryo's/foetussen in utero:
C11a. In Nederland
1644

C13. Onderzoeksgebied

- ☐ etiologie
- ☐ organisatorisch/zorgonderzoek
- ☐ diagnostiek
- ☐ preventie
- ☑ therapie
- ☐ veiligheid
- ☐ werkzaamheid
- ☐ farmacokinetiek
- ☐ farmacodynamiek
- ☐ bio-equivalentie
- ☐ dosis-respons
- ☐ farmacogenomics
- ☐ farmaco-economie
- ☐ anders

C14. Type onderzoek

- ○ observationeel onderzoek zonder invasieve metingen
- ○ observationeel onderzoek met invasieve metingen
- ◉ interventie-onderzoek

C15. In welke fase kan het onderzoek worden ingedeeld?

- ○ fase I (a)
- ○ fase II (b)
- ◉ fase III (c)
- ○ fase IV (d)
- ○ overige onderzoeken waarbij geneesmiddelen worden toegepast (e)
- ○ niet van toepassing

C17. Is er sprake van een andere interventie dan met geneesmiddelen zoals bedoeld in de Wet medisch-wetenschappelijk onderzoek met mensen (WMO) (zie toelichting)?

- ◉ Ja
- ○ Nee

C17a. ☐ Medisch hulpmiddel, namelijk
C17b. ☐ Operatie, namelijk
C17c. ☐ Psychosociale interventie, namelijk
C17d. ☐ Voeding(stoffen), namelijk
C17e. ☐ Bewegingstherapie, namelijk
C17f. ☐ interventie met radioactieve straling, namelijk
C17g. ☐ Blootstellingsonderzoek (bijv pesticidenonderzoek), namelijk
C17h.
C17i. ☑ andere interventie, namelijk

In het Engels: No sentinel lymph node procedure
In het Nederlands: Géén schildwachtklierprocedure

C18. Worden de onderzoeksproducten voor deze studie door de verrichter gratis verstrekt?

○ ja
○ nee
○ gedeeltelijk namelijk
◉ niet van toepassing

C19. Is/zijn er (een) controlegroep (en)?

◉ ja
○ nee

☐ ja, geneesmiddel
☐ ja, placebo
☐ ja, geen interventie
☑ ja, anders

namelijk De standaard behandeling: schildwachtklierprocedure

C20. Betreft het een gerandomiseerd onderzoek?

◉ ja
○ nee

namelijk

☑ open
☐ enkelblind
☐ dubbelblind
☐ parallel
☐ cross-over
☐ anders namelijk

C21. Op welke klasse(n) van aandoeningen heeft het onderzoek betrekking (maximaal 3)

☐ hartaandoeningen

☐ congenitale, familiaire en genetische aandoeningen
☐ bloed- en lymfestelsel aandoeningen
☐ zenuwstelsel aandoeningen
☐ oogaandoeningen
☐ evenwichtsorgaan- en ooraandoeningen
☐ ademhalingsstelsel-, thorax- en mediastinumaandoeningen
☐ maagdarmstelselaandoeningen
☐ nier- en urinewegaandoeningen
☐ huid- en onderhuidaandoeningen
☐ skeletspierstelsel- en bindweefselaandoeningen
☐ endocriene aandoeningen
☐ voedingsstoornissen en metabole ziekten

- ☐ infecties en parasitaire aandoeningen
- ☐ letsels, intoxicaties en verrichtingscomplicaties
- ☑ neoplasmata, benigne, maligne en niet-gespecificeerd (incl cysten en poliepen)
  borstneoplasmata maligne en niet-gespecificeerd (incl. tepel)
- ☑ chirurgische en medische verrichtingen
  borst therapeutische verrichtingen
- ☐ bloedvataandoeningen
- ☐ algemene aandoeningen en aandoeningen op de plek van toediening
- ☐ zwangerschap, perinatale periode en puerperium
- ☐ sociale omstandigheden
- ☐ immuunsysteemaandoeningen
- ☐ lever- en galaandoeningen
- ☐ voortplantingsstelsel- en borstaandoeningen
- ☐ psychische stoornissen
- ☐ overig, namelijk

C22.Geef twee synoniemen voor de aandoening die bestudeerd wordt, waarvan tenminste één lekenterm.

In het Engels:	breast cancer, sentinel lymph nodes, sentinel lymph node metastases
In het Nederlands:	borstkanker, schildwachtklieren, schildwachtkliermetastasen

C23. Beoogde start- en einddatum van het onderzoek

C23a. Start Datum (dd-mm-jjjj)	01-01-2015
C23b. Eind Datum (dd-mm-jjjj)	01-01-2027

C24. Zijn patiënten betrokken bij het ontwikkelen van het onderzoek? (Voor patiënten lees ook: patiëntenorganisaties, naasten of nabestaanden van patiënten en gezonde vrijwilligers)?

C30. Wordt de ervaring van proefpersonen na deelname aan het onderzoek geëvalueerd?

D. Sectie - Proefpersonen

D1. Is er een proefpersonenverzekering conform de WMO-eisen afgesloten of wordt aan de oordelende toetsingscommissie ontheffing gevraagd?

- ◉ proefpersonenverzekering is afgesloten bij verzekeringsmaatschappij
- ○ ontheffing van de verzekering wordt gevraagd
- ○ niet van toepassing - onderzoek valt onder de Embryowet en niet onder de WMO

Verzekeringsmaatschappij	Marketform (onderdeel van Lloyd's)

D2. Gezonde proefpersonen en/of patiënten

- ☐ Gezonde proefpersonen

- ☑ Patiënten

Aantal	1644

D4. Voornaamste inclusiecriteria

D4a. In het Engels
- Female
- Aged 18 years or older
- Pathologically confirmed invasive unilateral breast carcinoma
- A clinical T1-2 tumour (≤ 5cm)
- Will be treated with lumpectomy followed by whole breast radiotherapy
- Clinically node negative status: no signs of axillary lymph node metastases at physical examination and preoperative axillary ultrasound (or negative cyto-/histopathology)
- Written informed consent

D4b. In het Nederlands
- Vrouw
- Leeftijd 18 jaar of ouder
- Pathologisch bewezen invasief unilaterale borstkanker
- Klinisch T1-2 tumor (≤ 5cm)
- Zal worden behandeld met lumpectomie gevolgd door gehele borstbestraling
- Klinisch negatieve okselklierstatus: negatief lichamelijk onderzoek en echografie van de oksel (of negatieve cyto-/histologie)
- Schriftelijke informed consent

D5. Voornaamste exclusiecriteria

D5a. In het Engels

- Clinically node positive pre-operative
- Bilateral breast cancer
- Evidence of metastatic disease
- History of invasive breast cancer
- Previous treatment of the ipsilateral axilla with surgery or radiotherapy, except surgery for hidradenitis suppurativa or for other superficially located skin lesions, such as naevi
- Pregnant or nursing
- Other prior malignancies within the past 5 years, except successfully treated malignancies that occurred more than five years before randomization, and except successfully treated basal cell and squamous cell skin cancer, carcinoma in situ of the cervix or carcinoma in situ of the ipsilateral or contralateral breast
- Unable or unwilling to give informed consent

D5b. In het Nederlands

- Klinisch positieve okselklierstatus (preoperatief)
- Bilaterale borstkanker
- Primair (afstands-)gemetastaseerde borstkanker
- Invasieve borstkanker in de voorgeschiedenis
- Chirurgie of radiotherapie van ipsilaterale oksel in de voorgeschiedenis, behalve chirurgie voor hidradenitis suppurativa of andere oppervlakkig gelegen huidlaesies, zoals naevi
- Zwanger of lacterend
- Andere maligniteit in de afgelopen 5 jaar, behalve succesvol behandelde maligniteit die meer dan vijf jaar voor randomisatie optrad, en behalve succesvol behandelde huidkanker van type basaalcel of plaveiselcel, carcinoma in situ van de cervix, carcinoma in situ van de ipsilaterale of contralaterale mamma
- Niet in staat of niet bereid tot informed consent

D6. Bij welke categorie proefpersonen wordt het onderzoek uitgevoerd (meerdere antwoorden mogelijk)

- [x] 18 jaar of ouder en wilsbekwaam
- [ ] 18 jaar of ouder en wilsonbekwaam
- [ ] 12 t/m 17 jaar en in staat tot het geven van geïnformeerde toestemming
- [ ] 12 t/m 17 jaar en niet in staat tot het geven van geïnformeerde toestemming (wilsonbekwaam)
- [ ] jonger dan 12 jaar

D10. Verkeren (sommige) proefpersonen in een afhankelijkheidssituatie ten opzichte van de onderzoeker of degene die de deelnemers werft? (lees de toelichting voor voorbeelden wanneer er sprake kan zijn van een afhankelijkheidssituatie)

- (●) ja
- ( ) nee

D10a. Zo ja, waardoor?

Soms is de lokale onderzoeker ook de behandelend arts die de patiënt vraagt om deel te nemen aan de studie.

D10b. Waarom wordt het onderzoek juist met deze proefpersonen uitgevoerd en hoe worden de belangen van de proefpersonen gewaarborgd?

Het onderzoek moet met proefpersonen worden uitgevoerd die voldoen aan de beschreven in/exclusie criteria zodat de resultaten van de studie uiteindelijk ook naar de kliniek vertaald kunnen worden binnen deze patiëntenpopulatie. De belangen van de proefpersonen worden gewaarborgd door de geldende medisch ethische normen en richtlijn zoals vermeld in het protocol (o.a. onafhankelijk arts, uitgebreide patiëntinformatiefolder).

D11. Waaruit bestaat de vergoeding voor de proefpersonen?

- [x] geen vergoeding
- [ ] reiskosten
- [ ] financiële vergoeding (in Euro's)
- [ ] andere vergoeding

D12. Is deze vergoeding afhankelijk van bepaalde voorwaarden, bijvoorbeeld het voltooien van (een deel van) het onderzoek?

- ( ) ja (motiveer)
- ( ) nee
- (●) niet van toepassing

E. Sectie - Voor- en nadelen

E1. Wordt er bij dit onderzoek een rechtstreeks therapeutisch effect beoogd bij de proefpersonen / patiënten?

- (●) ja (therapeutisch onderzoek)
- ( ) nee (niet-therapeutisch onderzoek)

E1a. Zo ja, geef kort aan waaruit dit therapeutisch effect bestaat.

Het therapeutische effect bestaat uit:
- Het verlagen van het risico op okselbehandeling-gerelateerde korte en lange termijn complicaties;
- Het verbeteren van de kwaliteit van leven;
- Met een even goede ziektecontrole en algehele overleving.

E2. Waaruit bestaat de belasting van het onderzoek (en een eventueel daaraan voorafgaande keuring) voor de proefpersonen?

Tijdbeslag	per bezoek	20 min per keer voor invullen kwaliteit van leven vragenlijst
	totaal	20 min x 6 = 120 min
	totale duur van de studie voor de individuele proefpersoon	Tav de behandeling minder belasting dan normaal voor de studie-arm (geen schildwachtklierprocedure). Voor beide armen is extra het invullen van de kwaliteit van leven vragenlijst prerandomisatie en postrandomisatie op 5 momenten.

E3. Worden de proefpersonen in verband met het onderzoek in het ziekenhuis opgenomen of wordt een opname verlengd?

○ ja - het verblijf in het ziekenhuis/instituut wordt in verband met het onderzoek verlengd
○ ja - ze worden voor het onderzoek in het ziekenhuis/instituut opgenomen
◉ nee

E4. Beschrijf in hoeverre proefpersonen worden onderworpen aan handelingen dan wel een gedragswijze krijgen opgelegd, zoals vragenlijst, interviews, lichamelijk/psychologisch onderzoek, ontzegging, dieet (voor invasieve ingrepen: zie vraag E6)

In totaal zullen 700 proefpersonen (350 in de experimentele en 350 in de controlegroep) deelnemen aan het kwaliteit van leven onderzoek, bestaande uit een vragenlijst voor de randomisatie en op 5 tijdstippen na de randomisatie (na een half jaar, één, twee, drie, vijf en 10 jaar).

E5. Worden de proefpersonen getest op bepaalde aandoeningen/condities?

○ ja (motiveer)
◉ nee

E6. Welke extra (invasieve) ingrepen (anders dan bij de standaard behandeling) moeten de proefpersonen in het kader van het onderzoek ondergaan:

☑ Niet van toepassing

E9. Geef aan welke risico's er voor proefpersonen zijn verbonden aan deelname aan het onderzoek.

Hoewel de hypothese luidt dat het achterwege laten van de schildwachtklierprocedure geen nadelige invloed heeft op de lokale controle van de ziekte en de overlevingskans, bestaat er toch een kans dat proefpersonen in de experimentele arm een hoger risico hebben op een recidief in de oksel (regionaal recidief) en daardoor tijdens follow-up alsnog een vorm van okselklierbehandeling moeten ondergaan. Uit onderzoek blijkt dat het uitgesteld uitvoeren van okselklierbehandeling (bijvoorbeeld met okselklierdissectie) géén nadelige gevolgen heeft voor de overleving.

E9a. Geef op grond van uw eigen afweging aan waarom het uitvoeren van het onderzoek, in het licht van de belasting en/of risico's die voor proefpersonen aan deelname verbonden zijn, gerechtvaardigd is?

Het mogelijk hogere risico op een recidief in de oksel (regionaal recidief) weegt op tegen de lagere kans op ernstige okselklierbehandeling-gerelateerde morbiditeit (lymfoedeem, bewegingsbeperking in de schouder, arm- en schouderpijn, verminderd gevoel of zenuwpijn) en de verbetering van kwaliteit van leven.

E10. Indien het onderzoek bij minderjarige en/of wilsonbekwame proefpersonen wordt uitgevoerd en geen direct therapeutisch effect wordt beoogd: waarom kunnen belasting en risico's als minimaal worden beschouwd? (Verwijs eventueel naar de relevante pagina's in het protocol.

☑ niet van toepassing

E11. Kan de eventuele therapie na beëindiging van het onderzoek worden voortgezet?

○ ja (motiveer)
○ nee (motiveer)
◉ niet van toepassing

E12. Heeft deelname aan het onderzoek voor de proefpersoon tot gevolg dat van de standaardbehandeling of -diagnostiek kan worden afgeweken of deze kan worden uitgesteld?

◉ ja
○ nee
○ niet van toepassing

E12a. Zo ja, waaruit bestaat de afwijking of het uitstel en waarom is afwijking/uitstel

Proefpersonen in de controlegroep ondergaan de standaard behandeling, namelijk schildwachtklierprocedure volgens de Nederlandse borstkankerrichtlijn. Proefpersonen in de experimentele arm ondergaan primair géén schildwachtklierprocedure. Beide groepen ondergaan dezelfde follow-up middels lichamelijk onderzoek en aanvullend onderzoek op indicatie. Indien een proefpersoon zich in de follow-up periode presenteert met lymfekliermetastasen, kan alsnog besloten worden om een vorm van okselklierbehandeling uit te voeren. Deze beslissing wordt gemaakt tijdens het multidisciplinair overleg in het desbetreffende centrum waar de patiënt onder behandeling staat.

F. Sectie - Informatie en privacy

F1. Hoe worden de proefpersonen geworven en door wie (onderzoeker, behandelend arts, andere persoon) wordt de proefpersoon/wettelijke vertegenwoordiger geïnformeerd en om toestemming gevraagd?

Op het moment in de behandeling van borstkanker dat de patiënt voldoet aan de in/exclusiecriteria zal de behandeld arts informatie geven over de mogelijkheid tot deelname aan deze studie. Een schriftelijke patiëntinformatiebrief zal worden meegegeven aan de patiënt. De patiënt krijgt ruim de tijd (ten minste één week) om deze informatie thuis na te lezen en een beslissing te nemen. De patiënt kan haar interesse in deelname kenbaar maken aan haar behandelend arts bij een volgend polibezoek. De behandelend arts zal haar dan om toestemming vragen voor deelname aan de studie.

F2. Hoeveel bedenktijd krijgen de proefpersonen/wettelijke vertegenwoordigers om te beslissen over deelname?

Zo lang als nodig, met ten minste één week

F3. Wordt de huisarts, behandelend specialist en/of apotheker van de proefpersoon geïnformeerd over diens deelname aan het onderzoek?

- (•) ja (de proefpersoon dient hiervoor toestemming te geven)
- ( ) nee

F4. Worden persoonsgegevens gecodeerd?

- (•) ja
- ( ) nee

F4a. Zo ja, hoe is deze codering opgebouwd?

Alle proefpersonen krijgen een uniek studienummer. Het unieke studienummer betreft een opvolgnummer zonder verdere specifieke codering voor bijvoorbeeld centrum.

F4b. Wie heeft toegang tot de sleutel van deze code?

De behandelend arts en de onderzoeker

F4c. Wie hebben toegang tot de brondocumenten en eventuele andere tot de persoon herleidbare gegevens?

De lokaal datamanagers en de onderzoeker

F5. Hoe wordt het lichaamsmateriaal gedurende het onderzoek bewaard?

- ( ) in tot de proefpersoon herleidbare vorm (gecodeerd)
- ( ) in niet tot de proefpersoon herleidbare vorm (volledig geanonimiseerd)
- (•) niet van toepassing

F6. Wordt afgenomen lichaamsmateriaal na afloop van het onderzoek vernietigd?

- ( ) ja
- ( ) nee (motiveer)
- (•) niet van toepassing

F7. Kunnen proefpersonen na afloop van het onderzoek opnieuw benaderd worden (bijvoorbeeld voor nader onderzoek of follow-up)?

- ( ) ja
- (•) nee

G. Sectie - Financieel

G1. Door welke geldstroom wordt het onderzoek gefinancierd?

- [ ] eerste geldstroom (Geld van Ministerie van OC&W aan universiteiten)
- [ ] tweede geldstroom (NWO of KNAW), namelijk
- [x] derde geldstroom (anders dan 1e of 2e geldstroom, zoals collectebusfondsen, Europese Unie, vakministeries of bedrijven), namelijk
KWF Kankerbestrijding

G2. Wordt het onderzoek (mede) gefinancierd door de industrie/bedrijven?

- ○ ja - door de industrie/bedrijf zoals is opgegeven bij vraag B6/B7 (opdrachtgever van het onderzoek)
- ○ ja - (ook) door andere industrie/bedrijven dan de opdrachtgever
- ◉ nee

G3. Wat is de hoogte van de vergoeding die de arts/onderzoeker cq onderzoeksafdeling/maatschap ontvangt voor de uitvoering van het onderzoek?

- ○ Per patiënt of proefpersoon
- ◉ Per deelnemend centrum

bedrag afgerond in hele euro's: € 100,00

G3a. Hoe is de vergoeding opgebouwd?

De vergoeding betreft een financiële compensatie van € 100 excl. BTW als opstartvergoeding voor ieder deelnemend centrum.

G4. Heeft/hebben de onderzoeker(s) gedurende de afgelopen vijf jaar op een of andere wijze een persoonlijke financiële relatie (gehad) met de verrichter/sponsor van het huidige onderzoek?

- ○ ja (licht toe)
- ◉ nee

I. Sectie - Indiening en beoordeling

I1. Sla het formulier eerst op en selecteer vervolgens een METC of de CCMO

Commissie
METC NedMec

J. Sectie - Aanvullende opmerkingen

Aanvullende opmerkingen

K. Sectie - Samenvatting

Achtergrond van het onderzoek:

De schildwachtklierprocedure is de standaard methode voor lymfeklierstadiëring in borstkankerpatiënten met een klinisch negatieve okselklierstatus. In de algemene bevolking bevat de schildwachtklier in ongeveer 25% een uitzaaiing. Dit percentage is lager in patiënten met een kleine borsttumor en in borstsparend behandelde patiënten. De ACOSOG-Z0011 studie en de IBCSG 23-01 studie hebben recent aangetoond dat een completerende okselklierdissectie veilig achterwege gelaten kan worden bij borstsparend en met systemische therapie behandelde patiënten met een beperkt aantal schildwachtkliermetastasen. Dit roept de vraag op of de schildwachtklierprocedure nog wel noodzakelijk is voor borstsparend behandelde patiënten met een klinisch negatieve okselklierstatus. De klinisch negatieve okselklierstatus van de patiënten in de beschreven studies werd gesteld op basis van lichamelijk onderzoek. Het toevoegen van een preoperatieve echografie van de oksel zal zorgen voor een betere voorselectie van patiënten zonder lymfekliermetastasen, ten opzichte van lichamelijk onderzoek alleen. Daarbij sluit een negatieve echografie uitgevoerd in Nederland bijna alle patiënten uit met uitgebreide lymfekliermetastasen (≥4 lymfekliermetastasen). Bovendien omvat gehele borstbestraling na de borstsparende operatie in het algemeen het lagere deel van de oksel en behandelt daarmee eventueel achtergebleven uitzaaiingen. Hoewel de schildwachtklierprocedure minder invasief is vergeleken met een okselklierdissectie, komen complicaties nog steeds voor. Zo lijdt 6% van de patiënten die alleen een schildwachtklierprocedure ondergingen aan ernstige lymfoedeem na slechts 3 jaar. Deze studie gaat onderzoeken of de schildwachtklierprocedure veilig achterwege gelaten kan worden bij borstsparend behandelde patiënten, met een negatieve echografie van de oksel.

Doel van het onderzoek:

Het doel van deze studie is het verminderen van overbehandeling van de oksel bij patiënten met borstkanker en een klinisch negatieve okselklierstatus die behandeld worden met borstsparende therapie, door het achterwege laten van de schildwachtklierprocedure, om zodoende de kans op korte en lange termijn complicaties te verminderen en te zorgen voor een verbetering van de kwaliteit van leven met een even goede ziektecontrole en algehele overleving.

Onderzoeksopzet:

Het betreft een gerandomiseerde non-inferiority multicenter onderzoek. Patienten die voldoen aan de in- en exclusiecriteria worden na toestemming middels loting behandeld volgens één van de volgende behandelmethoden:

- Behandelmethode A (controle groep): schildwachtklierprocedure volgens de Nederlandse borstkankerrichtlijn.
- Behandelmethode B (studie groep): geen verdere okselklierstadiëring.

Stratificatiefactoren: leeftijd (≤50, 50≤75, >75 jaar), oestrogeen receptor status (positief vs. negatief), HER2neu status (geamplificeerd vs. niet geamplificeerd), klinisch tumor stadium (<3 cm vs. ≥3 cm), tumorgraad volgens gemodificeerde Bloom-Richardson (graad I-II vs. graad III), is of zal worden behandeld met primaire systemische therapie (ja/nee), participerend centrum.
Overige behandelingen (o.a. hormonale therapie, chemotherapie) worden op indicatie gegeven volgens de Nederlandse borstkankerrichtlijn.
Tijdens de follow-up periode van 10 jaar wordt de oksel nauwkeurig onderzocht middels lichamelijk onderzoek en echografie op indicatie. Indien sprake is van een cyto-/histologisch bewezen lymfekliermetastase tijdens follow-up, dan kan in het multidisciplinair overleg van de desbetreffende patiënt worden besloten tot het uitvoeren van een vorm van okselklierbehandeling.
Gevalideerde vragenlijsten worden verstrekt voor het onderzoeken van de invloed op het aantal complicaties en kwaliteit van leven (Lymph-ICF, QLQ-C30, QLQ-BR23) en om te meten of angst en persoonlijkheidstrekken invloed hebben op de uitkomst van

kwaliteit van leven (STAI-trait, NEO-FFI). Deze vragenlijsten worden verstrekt aan 700 van de 1644 patiënten (350 per arm), éénmaal voor de randomisatie en vijfmaal na de randomisatie (6 maanden, 1 jaar, 2 jaar, 5 jaar en 10 jaar). Vragenlijsten met betrekking to kosten-effectiviteit worden éénmaal voor de randomisatie en viermaal na de randomisatie (6 maanden, 1,2 en 3 jaar) afgenomen.

Onderzoekspopulatie:

Vrouwen van 18 jaar of ouder met pathologisch bewezen invasief mammacarcinoom die borstsparende therapie ondergaan.
cT1-2: primaire tumor is bij klinisch onderzoek kleiner dan 5 cm.
cN0: bij lichamelijk onderzoek en echografie van de oksel worden geen aanwijzingen voor lymfekliermetastasen gevonden of de cytologie/histologie na punctie/biopt van een lymfeklier is negatief.
Neoadjuvante systemische therapie is toegestaan indien de patiënt voor de start van de neoadjuvante systemische therapie een klinische T1-2N0 status heeft en waarvoor borstsparende therapie op dat moment al geschikt is.
De patiënt heeft informed consent persoonlijk ondertekend.

Interventie (indien van toepassing):

De controlegroep ondergaat wél de schildwachtklierprocedure volgens de Nederlands borstkankerrichtlijn. De experimentele arm ondergaan géén schildwachtklierprocedure.

Primaire onderzoeksvariabelen/uitkomstmaten:

Primaire uitkomstmaat:
- Regionaal recidief percentage

Secundaire onderzoeksvariabelen/uitkomstmaten (indien van toepassing):
Secundaire uitkomstmaten:
- Distant-disease free survival
- Overall survival
- Later uitgevoerde okselbehandeling
- Lokaal recidief percentage
- Overig-regionaal recidief percentage
- Contralaterale borstkanker
- Percentage (neo)adjuvante systemische therapie
- Kwaliteit van leven en okselbehandeling-gerelateerde morbiditeit
- Kosten-effectiviteit

Omschrijving en inschatting van belasting en risico (indien van toepassing):

Wat betreft belasting is er in deze studie géén sprake van extra diagnostiek of interventies (juist minder) en hoeft de patiënt niet vaker dan normaal voor controle naar het ziekenhuis. De eventuele extra belasting voor de patiënt zal alleen bestaan uit het invullen van kwaliteit van leven vragenlijsten (bij 700 van 1644 patiënten) éénmaal voor de randomisatie en vijfmaal na randomisatie (6 maanden, 1 jaar, 2 jaar, 5 jaar en 10 jaar). De hypothese luidt dat het achterwege laten van de schildwachtklierprocedure even veilig is als het wél uitvoeren van deze procedure. Desondanks is er een risico dat in de experimentele arm meer regionale recidieven optreden en dat bij patiënten op een later tijdstip alsnog een vorm van okselklierbehandeling moet worden uitgevoerd. Daartegenover staat dat er in de experimentele arm door het niet uitvoeren van de schildwachtklierprocedure minder kans is op morbiditeit, minder operaties en opnames in het ziekenhuis en een betere kwaliteit van leven. Patiënten zullen op geen enkele manier vertraging ondervinden als gevolg van deelname aan de studie.

K2.Engelse Samenvatting

Background of the study:
The NSABP B-04 trial revealed that omitting primary axillary treatment of occult positive lymph nodes in clinically node negative breast cancer patients does not affect survival, even after 25 years of follow-up and without the use of adjuvant systemic or radiation therapy. A delayed axillary dissection in case lymph nodes become clinically positive does not affect survival and prevents axillary overtreatment in the majority of patients. The ACOSOG Z0011 and IBCSG 23-01 trials revealed that completion axillary dissection can be safely omitted in clinically node negative patients with metastatic sentinel nodes. Patients randomized for watchful waiting were likely to have residual nodal disease (13-27%) that was not surgically removed. Nevertheless, survival rates were not affected and regional recurrence rates low. Use of axillary ultrasound will improve the preoperative selection of node negative patients, as it selects patients with a more favourable tumour load and accurately excludes advanced nodal disease (≥4 metastatic nodes). Biology and adjuvant systemic and radiation therapy are all factors that most likely diminish the risk that possible metastases left in situ develop into clinically detectable lymph nodes. Patients treated with breast conserving therapy are significantly more often diagnosed with pathologic node negative disease or with micrometastatic disease, compared to patients treated with mastectomy. The risk for occult (macro)metastases in cT1-2N0 breast cancer patients treated with breast conserving therapy is low: about 88% will be node negative and about 95% of node positive patients will have no lymph node metastases beyond the sentinel node. Reflected by the low regional recurrence rate after a (false) negative sentinel node, only a small amount of patients (0.8%) with node positive disease is expected to develop clinically detectable nodal disease.

Objective of the study:
The objective of this study is to decrease the number of breast cancer patients receiving overtreatment of the axilla, in order to positively influence the axillary morbidity rate and quality of life. Therefore, we investigate whether the sentinel lymph node procedure can be safely omitted in clinically node negative breast cancer patients undergoing breast conserving therapy, in terms of non-inferior regional control, distant-disease free and overall survival. All objectives are measured during a follow-up of 10 years.

Study design:
A randomized controlled non-inferiority multicenter study. Patients who meet the in- and exclusioncriteria will be randomized after

informed consent for one of the two treatment methods:
- Arm A (control arm): lumpectomy with sentinel lymph node procedure, followed by radiotherapy of the breast with or without completion axillary treatment according to the Dutch breast cancer guideline.
- Arm B (experimental arm): lumpectomy without further axillary staging, followed by radiotherapy of the breast.
Stratification: age (≤50, 50≤75, >75 years old), oestrogen receptor status (positive vs. negative), HER2neu status (amplified vs. not amplified), clinical tumour stage prior to any treatment (<3 cm vs. ≥3 cm), grading (grade I-II vs. III - according to Bloom-Richardson grading system), is or will be treated with primary systemic therapy (yes/no) and participating centre.
Adjuvant systemic treatment is administered if indicated according to the Dutch breast cancer guideline.
Primary systemic therapy is allowed if the patient has a clinical T1-2N0 status (initial stage) that is amenable to lumpectomy pre-systemic therapy.
Yearly follow-up with physical examination of the axilla for 10 years. Axillary ultrasound (+/- tissue sampling) on indication. Staging for distant metastases and/or a delayed axillary lymph node dissection if indicated by the multidisciplinary team. Validated questionnaires are used to assess axillary morbidity rate and quality of life (Lymph-ICF, QLQ-C30, QLQ-BR23) and to measure if anxiety and personality traits influence the outcome of quality of life (STAI-trait, NEO-FFI) in 700 of 878 patients. These questionnaires are provided before randomization and sequentially post randomization at 6 months, 1, 2, 5 and 10 years. Cost-effectiveness questionnaires are provided before randomization and sequentially post randomization at 6 months, 1, 2 and 3 years.

Study population:
Women aged 18 years or older with pathologically confirmed invasive breast carcinoma about to undergo breast conserving surgery followed by radiotherapy of the breast.
cT1-2: primary tumour is smaller than 5 cm.
cN0: no signs of axillary lymph node metastases at physical examination and axillary ultrasound (+/- tissue sampling).
Primary systemic treatment is allowed.
Patient is able to and signed the informed consent form.

Intervention (if applicable):
Patients in the control arm will be treated with the sentinel lymph node procedure according to the Dutch breast cancer guideline.
Patients in the experimental arm will not be treated with the sentinel lymph node procedure.

Primary study parameters/outcome of the study:
Primary endpoint:
- Regional recurrence rate

Secundary study parameters/outcome of the study (if applicable):
Secondary endpoints:
- Distant-disease free survival
- Overall survival
- Delayed axillary treatment
- Local recurrence rate
- Other-regional recurrence rate
- Contralateral breast cancer rate
- Percentage difference in the administration of (neo)adjuvant systemic therapy
- Quality of life and axillary morbidity rate
- Cost-effectiveness

Nature and extent of the burden and risks associated with participation, benefit and group relatedness (if applicable):
There is no extra burden for participating patients as regard to additional diagnostic tests/interventions and follow-up. Any additional burden for the patient will only consist of completing quality of life questionnaires in 700 of 1644 study patients. These questionnaires are provided before randomization and sequentially post randomization at 6 months, 1, 2, 5 and 10 years.
We hypothesize that omitting the sentinel lymph node procedure is not inferior to the standard treatment. Nevertheless, possible disadvantages are a worse regional recurrence rate, and delayed axillary treatment if indicated. Patients in the experimental arm have the possible advantages of less morbidity, fewer operations and hospitalizations and an improved quality of life, compared to patients treated with the sentinel lymph node procedure.
No patient will encounter any delay in their treatment as a result of inclusion.

ONDERTEKENING

De verrichter en indiener verklaren hierbij:
a. het formulier (en samenvatting) volledig en naar waarheid te hebben ingevuld;
b. de antwoorden op de vragen uit het ABR-formulier niet in strijd zijn met het bijbehorende onderzoeksdossier en onderzoekscontract

Naar waarheid getekend, door de verrichter (=opdrachtgever) — door de indiener

datum .... datum ....

Handtekening		Handtekening	
naam	U.P. Neumann	naam	F. van Duijnhoven
functie	Hoofd afdeling Chirurgie, MUMC+	functie	Chirurg, NKI-AVL

Sluiten Print

Sluiten Print

Formulier voor medisch-ethische beoordeling en registratie

ABR-formulier, versie december 2015

Onderzoeksdossiernummer

ABR Nummer	**49315**	Status	**Beoordeeld - Positief**
Versie	**12**	Status per	**27-02-2020**
Jaar	**20**		
Dossiernummer	**NL49315.031.14**		
Reden voor	**toevoegen centrum**		

A. Sectie - Openbaar maken gegevens medisch wetenschappelijk onderzoek

A1. Het CCMO-register is een voor ieder toegankelijk openbaar trial register. De antwoorden op de vragen gemarkeerd met een wereldbol en de samenvatting bij dit formulier worden openbaar gemaakt in het CCMO register.

B. Sectie - Administratief

B1. Betreft het onderzoek met geneesmiddelen (inclusief gentherapie, somatische celtherapie, vaccinonderzoek, GGO's, zie verdere toelichting) als bedoeld in de Wet medisch-wetenschappelijk onderzoek met mensen (WMO)?

○ ja
◉ nee

B2. Houdt het onderzoek verband met een eerder door een erkende METC of door de CCMO beoordeelde studie of is het onderzoek reeds eerder bij een erkende METC ter beoordeling voorgelegd?

○ ja, het onderzoek houdt verband met – of is het vervolg op – een eerder beoordeelde studie
○ ja, het onderzoek is eerder ter beoordeling aan een erkende METC of de CCMO voorgelegd (stuur kopie besluit mee)
◉ nee

B4. Is het protocol (nog) in een ander openbaar trial register geregistreerd?

○ ja
◉ nee

B5. Naam indiener/contactpersoon voor de oordelende toetsingscommissie

**B5a.**	Achternaam indiener/contactpersoon	Duijnhoven
	Titel en voorletters	dr. F.
	Tussenvoegsels	van
**B5b.**	Type organisatie/bedrijf	Overige Ziekenhuizen
	Organisatie/bedrijf	Antoni van Leeuwenhoek Ziekenhuis
	Naam organisatie/bedrijf	IKNL trialbureau
	Afdeling	Chirurgie
	Adres	Plesmanlaan 121
	Postcode en plaats	1066 CX Amsterdam
	Land	NL
**B5c.**	Intern adres	afdeling Heelkunde
	Telefoon	020 512 9111
	Fax	020 512 2554
	E-mail	f.v.duijnhoven@nki.nl

B6. Is de indiener werkzaam bij de opdrachtgever/sponsor (verrichter) van het onderzoek?

○ Ja ◉ Nee

B7. Opdrachtgever/sponsor van het onderzoek (verrichter volgens de WMO)

Type Organisatie/Bedrijf	Universitair Medisch Centrum
Organisatie/Bedrijf	Medisch Universitair Ziekenhuis Maastricht

Adres	P. Debyelaan 25
Postcode en plaats	6229 HX Maastricht
Land	NL
Telefoon	0433877477
Fax	0433875473
E-mail	m.smidt@mumc.nl

C. Sectie - Onderzoek

C1. Volledige titel van het onderzoek
C1a. In het Engels
Clinically node negative breast cancer patients undergoing breast conserving therapy: Sentinel lymph node procedure versus follow-up. A Dutch randomized controlled multicentre trial.

C1b. In het Nederlands
Patiënten met borstkanker en een klinisch negatieve okselklierstatus die een borstsparende behandeling ondergaan: Schildwachtklierprocedure versus follow-up. Een Nederlandse gerandomiseerde multicenter studie.

C2. Verkorte titel van het onderzoek/acroniem
C2a. In het Engels
BOOG 2013-08

C2b. In het Nederlands (Let op: deze korte titel wordt vermeld binnen ToetsingOnline)
BOOG 2013-08

C3. Trefwoorden (maximaal 4, plaats elk trefwoord op een aparte regel)
C3a. In het Engels
Breast neoplasms
Breast conserving therapy
Sentinel lymph node biopsy
Axilla

C3b. In het Nederlands
Borstkanker
Borstsparende behandeling
Schildwachtklierprocedure
Oksel

C4. Beschrijf het belang van het onderzoek en de beoogde toepassing van de resultaten (verwijs eventueel naar de relevante pagina's in het protocol).
Het doel van dit onderzoek is om:
1) Overbehandeling van de oksel te verminderen;
2) Het aantal korte en lange termijn complicaties te verminderen;
3) De kwaliteit van leven te verbeteren;
4) Met een even goede ziektecontrole en algehele overleving

Dit willen wij bereiken door te onderzoeken of de schildwachtklierprocedure veilig achterwege gelaten kan worden bij patiënten met borstkanker en een klinisch negatieve okselklierstatus behandeld met borstsparende therapie.

C6. Betreft het onderzoek een multicenter-onderzoek?
- ○ nee
- ◉ ja - alleen in Nederland
- ○ ja - internationaal binnen de Europese Unie
- ○ ja - internationaal ook buiten de Europese Unie

C7. Is er bij multicenter-onderzoek sprake van een coördinerend onderzoeker?
- ◉ ja namelijk
- ○ nee

Naam onderzoeker:
M.L. Smidt

C8. Wie is/zijn medisch verantwoordelijk voor de proefpersonen die deelnemen aan het onderzoek
Behandelend arts en lokale hoofdonderzoeker

C9. In welk centrum/welke centra (incl. huisartsenpraktijken) in Nederland wordt het onderzoek uitgevoerd?

Centrum	Proefpersonen	Hoofdonderzoeker	Onafhankelijk arts
Amphia Ziekenhuis	69	E.J.T. Luiten	S.O. Breukink (Maastricht UMC+)
Zuyderland Medisch Centrum	39	E.R.M. van Haaren	S.O. Breukink (Maastricht UMC+)

Canisius Wilhelmina Ziekenhuis	46	L.J.A. Strobbe	S.O. Breukink (Maastricht UMC+)
Catharina-ziekenhuis	46	G.A.P. Nieuwenhuijzen	S.O. Breukink (Maastricht UMC+)
Deventer Ziekenhuis	39	H. de Vries	S.O. Breukink (Maastricht UMC+)
Diakonessenhuis Utrecht	39	T. van Dalen	S.O. Breukink (Maastricht UMC+)
Gelre Ziekenhuizen	37	J.H.G. Klinkenberg	S.O. Breukink (Maastricht UMC+)
HagaZiekenhuis	41	I. Jannink	S.O. Breukink (Maastricht UMC+)
Isala Klinieken	54	A.B. Francken	S.O. Breukink (Maastricht UMC+)
Laurentius Ziekenhuis	23	A.V.R.J. Bell	S.O. Breukink (Maastricht UMC+)
Academisch Ziekenhuis Maastricht	39	M.L. Smidt	S.O. Breukink (Maastricht UMC+)
Maxima Medisch Centrum	31	K. Schenk	S.O. Breukink (Maastricht UMC+)
Antoni van Leeuwenhoek Ziekenhuis	93	F. van Duijnhoven	S.O. Breukink (Maastricht UMC+)
Universitair Medisch Centrum Sint Radboud	31	J.H.W. de Wilt	S.O. Breukink (Maastricht UMC+)
Sint Antonius Ziekenhuis	58	R. Koelemij	S.O. Breukink (Maastricht UMC+)
Tergooiziekenhuizen locatie Hilversum	42	E.J.C. Vriens	S.O. Breukink (Maastricht UMC+)
Universitair Medisch Centrum Groningen	17	J. de Vries	S.O. Breukink (Maastricht UMC+)
Universitair Medisch Centrum Utrecht	23	A.J. Witkamp	S.O. Breukink (Maastricht UMC+)
Viecuri Medisch Centrum voor Noord-Limburg	39	P.H.A Nijhuis	S.O. Breukink (Maastricht UMC+)
Ziekenhuisvoorzieningen Gelderse Vallei	32	W.K. de Roos	S.O. Breukink (Maastricht UMC+)
Rivierenland Ziekenhuis	23	M.F. Sier	S.O. Breukink (Maastricht UMC+)
Noordwest Ziekenhuisgroep	54	K.M. Blaauwendraat	S.O. Breukink (Maastricht UMC+)
Rijnstate Ziekenhuis	62	R. van Eekeren	S.O. Breukink (Maastricht UMC+)
Jeroen Bosch Ziekenhuis	20	M. Bessems	B. Biesma (Jeroen Bosch ziekenhuis)
Alrijne ziekenhuis	50	P. Neijenhuis	S.O. Breukink (Maastricht UMC+)
Spaarne Gasthuis	30	B. Kortmann	S.O. Breukink (Maastricht UMC+)
St. Jansdal	40	Schouten van der Velden	S.O. Breukink (Maastricht UMC+)
Treant Zorggroep	50	L. Smit	S.O. Breukink (Maastricht UMC+

C10. Betreft het onderzoek met:

- ☑ mensen
- ☐ geslachtscellen
- ☐ (rest-)embryo's
- ☐ foetussen in utero

C11. Beoogd totaal aantal proefpersonen/(rest)embryo's/foetussen in utero:
C11a. In Nederland
1644

C13. Onderzoeksgebied

- ☐ etiologie
- ☐ organisatorisch/zorgonderzoek
- ☐ diagnostiek
- ☐ preventie
- ☑ therapie
- ☐ veiligheid
- ☐ werkzaamheid

- ☐ farmacokinetiek
- ☐ farmacodynamiek
- ☐ bio-equivalentie
- ☐ dosis-respons
- ☐ farmacogenomics
- ☐ farmaco-economie
- ☐ anders

C14. Type onderzoek

- ○ observationeel onderzoek zonder invasieve metingen
- ○ observationeel onderzoek met invasieve metingen
- ◉ interventie-onderzoek

C15. In welke fase kan het onderzoek worden ingedeeld?

- ○ fase I (a)
- ○ fase II (b)
- ◉ fase III (c)
- ○ fase IV (d)
- ○ overige onderzoeken waarbij geneesmiddelen worden toegepast (e)
- ○ niet van toepassing

C17. Is er sprake van een andere interventie dan met geneesmiddelen zoals bedoeld in de Wet medisch-wetenschappelijk onderzoek met mensen (WMO) (zie toelichting)?

- ◉ Ja
- ○ Nee

C17a. ☐ Medisch hulpmiddel, namelijk
C17b. ☐ Operatie, namelijk
C17c. ☐ Psychosociale interventie, namelijk
C17d. ☐ Voeding(stoffen), namelijk
C17e. ☐ Bewegingstherapie, namelijk
C17f. ☐ interventie met radioactieve straling, namelijk
C17g. ☐ Blootstellingsonderzoek (bijv pesticidenonderzoek), namelijk
C17h.
C17i. ☑ andere interventie, namelijk

In het Engels: No sentinel lymph node procedure
In het Nederlands: Géén schildwachtklierprocedure

C18. Worden de onderzoeksproducten voor deze studie door de verrichter gratis verstrekt?

- ○ ja
- ○ nee
- ○ gedeeltelijk namelijk
- ◉ niet van toepassing

C19. Is/zijn er (een) controlegroep (en)?

- ◉ ja
- ○ nee

- ☐ ja, geneesmiddel
- ☐ ja, placebo
- ☐ ja, geen interventie
- ☑ ja, anders

namelijkDe standaard behandeling: schildwachtklierprocedure

C20. Betreft het een gerandomiseerd onderzoek?

- (●) ja
- ( ) nee

namelijk

- [x] open
- [ ] enkelblind
- [ ] dubbelblind
- [ ] parallel
- [ ] cross-over
- [ ] anders namelijk

C21. Op welke klasse(n) van aandoeningen heeft het onderzoek betrekking (maximaal 3)

- [ ] hartaandoeningen
- [ ] congenitale, familiaire en genetische aandoeningen
- [ ] bloed- en lymfestelsel aandoeningen
- [ ] zenuwstelsel aandoeningen
- [ ] oogaandoeningen
- [ ] evenwichtsorgaan- en ooraandoeningen
- [ ] ademhalingsstelsel-, thorax- en mediastinumaandoeningen
- [ ] maagdarmstelselaandoeningen
- [ ] nier- en urinewegaandoeningen
- [ ] huid- en onderhuidaandoeningen
- [ ] skeletspierstelsel- en bindweefselaandoeningen
- [ ] endocriene aandoeningen
- [ ] voedingsstoornissen en metabole ziekten
- [ ] infecties en parasitaire aandoeningen
- [ ] letsels, intoxicaties en verrichtingscomplicaties
- [x] neoplasmata, benigne, maligne en niet-gespecificeerd (incl cysten en poliepen)
  borstneoplasmata maligne en niet-gespecificeerd (incl. tepel)
- [x] chirurgische en medische verrichtingen
  borst therapeutische verrichtingen
- [ ] bloedvataandoeningen
- [ ] algemene aandoeningen en aandoeningen op de plek van toediening
- [ ] zwangerschap, perinatale periode en puerperium
- [ ] sociale omstandigheden
- [ ] immuunsysteemaandoeningen
- [ ] lever- en galaandoeningen
- [ ] voortplantingsstelsel- en borstaandoeningen
- [ ] psychische stoornissen
- [ ] overig, namelijk

C22.Geef twee synoniemen voor de aandoening die bestudeerd wordt, waarvan tenminste één lekenterm.

In het Engels: breast cancer, sentinel lymph nodes, sentinel lymph node metastases

In het Nederlands: borstkanker, schildwachtklieren, schildwachtkliermetastasen

C23. Beoogde start- en einddatum van het onderzoek

C23a. Start Datum (dd-mm-jjjj) 01-01-2015

C23b. Eind Datum (dd-mm-jjjj) 01-01-2027

C24. Zijn patiënten betrokken bij het ontwikkelen van het onderzoek? (Voor patiënten lees ook: patiëntenorganisaties, naasten of nabestaanden van patiënten en gezonde vrijwilligers)?

C30. Wordt de ervaring van proefpersonen na deelname aan het onderzoek geëvalueerd?

D. Sectie - Proefpersonen

D1. Is er een proefpersonenverzekering conform de WMO-eisen afgesloten of wordt aan de oordelende toetsingscommissie ontheffing gevraagd?

- (●) proefpersonenverzekering is afgesloten bij verzekeringsmaatschappij
- ( ) ontheffing van de verzekering wordt gevraagd
- ( ) niet van toepassing - onderzoek valt onder de Embryowet en niet onder de WMO

Verzekeringsmaatschappij Marketform (onderdeel van Lloyd's)

D2. Gezonde proefpersonen en/of patiënten

- [ ] Gezonde proefpersonen

- [x] Patiënten

Aantal 1644

D4. Voornaamste inclusiecriteria
D4a. In het Engels
- Female
- Aged 18 years or older
- Pathologically confirmed invasive unilateral breast carcinoma
- A clinical T1-2 tumour (≤ 5cm)
- Will be treated with lumpectomy followed by whole breast radiotherapy
- Clinically node negative status: no signs of axillary lymph node metastases at physical examination and preoperative axillary ultrasound (or negative cyto-/histopathology)
- Written informed consent

D4b. In het Nederlands
- Vrouw
- Leeftijd 18 jaar of ouder
- Pathologisch bewezen invasief unilaterale borstkanker
- Klinisch T1-2 tumor (≤ 5cm)
- Zal worden behandeld met lumpectomie gevolgd door gehele borstbestraling
- Klinisch negatieve okselklierstatus: negatief lichamelijk onderzoek en echografie van de oksel (of negatieve cyto-/histologie)
- Schriftelijke informed consent

D5. Voornaamste exclusiecriteria
D5a. In het Engels
- Clinically node positive pre-operative
- Bilateral breast cancer
- Evidence of metastatic disease
- History of invasive breast cancer
- Previous treatment of the ipsilateral axilla with surgery or radiotherapy, except surgery for hidradenitis suppurativa or for other superficially located skin lesions, such as naevi
- Pregnant or nursing
- Other prior malignancies within the past 5 years, except successfully treated malignancies that occurred more than five years before randomization, and except successfully treated basal cell and squamous cell skin cancer, carcinoma in situ of the cervix or carcinoma in situ of the ipsilateral or contralateral breast
- Unable or unwilling to give informed consent

D5b. In het Nederlands
- Klinisch positieve okselklierstatus (preoperatief)
- Bilaterale borstkanker
- Primair (afstands-)gemetastaseerde borstkanker
- Invasieve borstkanker in de voorgeschiedenis
- Chirurgie of radiotherapie van ipsilaterale oksel in de voorgeschiedenis, behalve chirurgie voor hidradenitis suppurativa of andere oppervlakkig gelegen huidlaesies, zoals naevi
- Zwanger of lacterend
- Andere maligniteit in de afgelopen 5 jaar, behalve succesvol behandelde maligniteit die meer dan vijf jaar voor randomisatie optrad, en behalve succesvol behandelde huidkanker van type basaalcel of plaveiselcel, carcinoma in situ van de cervix, carcinoma in situ van de ipsilaterale of contralaterale mamma
- Niet in staat of niet bereid tot informed consent

D6. Bij welke categorie proefpersonen wordt het onderzoek uitgevoerd (meerdere antwoorden mogelijk)

- [x] 18 jaar of ouder en wilsbekwaam
- [ ] 18 jaar of ouder en wilsonbekwaam
- [ ] 12 t/m 17 jaar en in staat tot het geven van geïnformeerde toestemming
- [ ] 12 t/m 17 jaar en niet in staat tot het geven van geïnformeerde toestemming (wilsonbekwaam)
- [ ] jonger dan 12 jaar

D10. Verkeren (sommige) proefpersonen in een afhankelijkheidssituatie ten opzichte van de onderzoeker of degene die de deelnemers werft? (lees de toelichting voor voorbeelden wanneer er sprake kan zijn van een afhankelijkheidssituatie)

- (●) ja
- ( ) nee

D10a. Zo ja, waardoor?

Soms is de lokale onderzoeker ook de behandelend arts die de patiënt vraagt om deel te nemen aan de studie.

D10b. Waarom wordt het onderzoek juist met deze proefpersonen uitgevoerd en hoe worden de belangen van de proefpersonen gewaarborgd?

Het onderzoek moet met proefpersonen worden uitgevoerd die voldoen aan de beschreven in/exclusie criteria zodat de resultaten van de studie uiteindelijk ook naar de kliniek vertaald kunnen worden binnen deze patiëntenpopulatie. De belangen van de proefpersonen worden gewaarborgd door de geldende medisch ethische normen en richtlijn zoals vermeld in het protocol (o.a. onafhankelijk arts, uitgebreide patiëntinformatiefolder).

D11. Waaruit bestaat de vergoeding voor de proefpersonen?

- [x] geen vergoeding
- [ ] reiskosten
- [ ] financiële vergoeding (in Euro's)
- [ ] andere vergoeding

D12. Is deze vergoeding afhankelijk van bepaalde voorwaarden, bijvoorbeeld het voltooien van (een deel van) het onderzoek?

- ○ ja (motiveer)
- ○ nee
- ● niet van toepassing

E. Sectie - Voor- en nadelen

E1. Wordt er bij dit onderzoek een rechtstreeks therapeutisch effect beoogd bij de proefpersonen / patiënten?

- ● ja (therapeutisch onderzoek)
- ○ nee (niet-therapeutisch onderzoek)

E1a. Zo ja, geef kort aan waaruit dit therapeutisch effect bestaat.

Het therapeutische effect bestaat uit:
- Het verlagen van het risico op okselbehandeling-gerelateerde korte en lange termijn complicaties;
- Het verbeteren van de kwaliteit van leven;
- Met een even goede ziektecontrole en algehele overleving.

E2. Waaruit bestaat de belasting van het onderzoek (en een eventueel daaraan voorafgaande keuring) voor de proefpersonen?

Tijdbeslag	per bezoek	20 min per keer voor invullen kwaliteit van leven vragenlijst
	totaal	20 min x 6 = 120 min
	totale duur van de studie voor de individuele proefpersoon	Tav de behandeling minder belasting dan normaal voor de studie-arm (geen schildwachtklierprocedure). Voor beide armen is extra het invullen van de kwaliteit van leven vragenlijst prerandomisatie en postrandomisatie op 5 momenten.

E3. Worden de proefpersonen in verband met het onderzoek in het ziekenhuis opgenomen of wordt een opname verlengd?

- ○ ja - het verblijf in het ziekenhuis/instituut wordt in verband met het onderzoek verlengd
- ○ ja - ze worden voor het onderzoek in het ziekenhuis/instituut opgenomen
- ● nee

E4. Beschrijf in hoeverre proefpersonen worden onderworpen aan handelingen dan wel een gedragswijze krijgen opgelegd, zoals vragenlijst, interviews, lichamelijk/psychologisch onderzoek, ontzegging, dieet (voor invasieve ingrepen: zie vraag E6)

Initieel zouden 700 proefpersonen (350 in de experimentele en 350 in de controlegroep) deelnemen aan het kwaliteit van leven onderzoek, bestaande uit een vragenlijst voor de randomisatie en op 5 tijdstippen na de randomisatie (na een half jaar, één, twee, drie, vijf en 10 jaar).
Later werd een kosteneffectiviteitsstudie (KEA) gestart en gekoppeld aan de kwaliteit van leven studie. Het inclusiepercentage van de KEA bedraagt 66%. Voor deze sub-studie dienen ten minste 474 patiënten geïncludeerd te worden. Bij dit inclusiepercentage, dienen er nu nog 74 patiënten benaderd te worden (toelichting: 1056 nodig voor de substudie, inmiddels 983 geïncludeerd.)
Gezien het kwaliteit van leven -onderzoek van groot belang is voor de kosteneffectiviteitsstudie, wordt de kwaliteit van leven-vragenlijst dus verlengd uitgestuurd tot de inclusie van de kosteneffectiviteitsstudie volledig is.
Indien patiënten geen of slechts een van de eerste twee vragenlijst retourneren, zal er geen vervolgvragenlijst worden toegestuurd, doch een bedankbrief voor deelname aan de studie.
Na patiënt 1056 worden er zowel geen KEA als Kwaliteit van leven-vragenlijsten meer uitgestuurd.

E5. Worden de proefpersonen getest op bepaalde aandoeningen/condities?

○ ja (motiveer)
◉ nee

E6. Welke extra (invasieve) ingrepen (anders dan bij de standaard behandeling) moeten de proefpersonen in het kader van het onderzoek ondergaan:

☑ Niet van toepassing

E9. Geef aan welke risico's er voor proefpersonen zijn verbonden aan deelname aan het onderzoek.

Hoewel de hypothese luidt dat het achterwege laten van de schildwachtklierprocedure geen nadelige invloed heeft op de lokale controle van de ziekte en de overlevingskans, bestaat er toch een kans dat proefpersonen in de experimentele arm een hoger risico hebben op een recidief in de oksel (regionaal recidief) en daardoor tijdens follow-up alsnog een vorm van okselklierbehandeling moeten ondergaan. Uit onderzoek blijkt dat het uitgesteld uitvoeren van okselklierbehandeling (bijvoorbeeld met okselklierdissectie) géén nadelige gevolgen heeft voor de overleving.

E9a. Geef op grond van uw eigen afweging aan waarom het uitvoeren van het onderzoek, in het licht van de belasting en/of risico's die voor proefpersonen aan deelname verbonden zijn, gerechtvaardigd is?

Het mogelijk hogere risico op een recidief in de oksel (regionaal recidief) weegt op tegen de lagere kans op ernstige okselklierbehandeling-gerelateerde morbiditeit (lymfoedeem, bewegingsbeperking in de schouder, arm- en schouderpijn, verminderd gevoel of zenuwpijn) en de verbetering van kwaliteit van leven.

E10. Indien het onderzoek bij minderjarige en/of wilsonbekwame proefpersonen wordt uitgevoerd en geen direct therapeutisch effect wordt beoogd: waarom kunnen belasting en risico's als minimaal worden beschouwd? (Verwijs eventueel naar de relevante pagina's in het protocol.

☑ niet van toepassing

E11. Kan de eventuele therapie na beëindiging van het onderzoek worden voortgezet?

○ ja (motiveer)
○ nee (motiveer)
◉ niet van toepassing

E12. Heeft deelname aan het onderzoek voor de proefpersoon tot gevolg dat van de standaardbehandeling of -diagnostiek kan worden afgeweken of deze kan worden uitgesteld?

◉ ja
○ nee
○ niet van toepassing

E12a. Zo ja, waaruit bestaat de afwijking of het uitstel en waarom is afwijking/uitstel

Proefpersonen in de controlegroep ondergaan de standaard behandeling, namelijk schildwachtklierprocedure volgens de Nederlandse borstkankerrichtlijn. Proefpersonen in de experimentele arm ondergaan primair géén schildwachtklierprocedure. Beide groepen ondergaan dezelfde follow-up middels lichamelijk onderzoek en aanvullend onderzoek op indicatie. Indien een proefpersoon zich in de follow-up periode presenteert met lymfekliermetastasen, kan alsnog besloten worden om een vorm van okselklierbehandeling uit te voeren. Deze beslissing wordt gemaakt tijdens het multidisciplinair overleg in het desbetreffende centrum waar de patiënt onder behandeling staat.

F. Sectie - Informatie en privacy

F1. Hoe worden de proefpersonen geworven en door wie (onderzoeker, behandelend arts, andere persoon) wordt de proefpersoon/wettelijke vertegenwoordiger geïnformeerd en om toestemming gevraagd?

Op het moment in de behandeling van borstkanker dat de patiënt voldoet aan de in/exclusiecriteria zal de behandeld arts informatie geven over de mogelijkheid tot deelname aan deze studie. Een schriftelijke patiëntinformatiebrief zal worden meegegeven aan de patiënt. De patiënt krijgt ruim de tijd (ten minste één week) om deze informatie thuis na te lezen en een beslissing te nemen. De patiënt kan haar interesse in deelname kenbaar maken aan haar behandelend arts bij een volgend polibezoek. De behandelend arts zal haar dan om toestemming vragen voor deelname aan de studie.

F2. Hoeveel bedenktijd krijgen de proefpersonen/wettelijke vertegenwoordigers om te beslissen over deelname?

Zo lang als nodig, met ten minste één week

F3. Wordt de huisarts, behandelend specialist en/of apotheker van de proefpersoon geïnformeerd over diens deelname aan het onderzoek?

◉ ja (de proefpersoon dient hiervoor toestemming te geven)
○ nee

F4. Worden persoonsgegevens gecodeerd?

◉ ja
○ nee

F4a. Zo ja, hoe is deze codering opgebouwd?
Alle proefpersonen krijgen een uniek studienummer. Het unieke studienummer betreft een opvolgnummer zonder verdere specifieke codering voor bijvoorbeeld centrum.

F4b. Wie heeft toegang tot de sleutel van deze code?
De behandelend arts en de onderzoeker

F4c. Wie hebben toegang tot de brondocumenten en eventuele andere tot de persoon herleidbare gegevens?
De lokaal datamanagers en de onderzoeker

F5. Hoe wordt het lichaamsmateriaal gedurende het onderzoek bewaard?
○ in tot de proefpersoon herleidbare vorm (gecodeerd)
○ in niet tot de proefpersoon herleidbare vorm (volledig geanonimiseerd)
◉ niet van toepassing

F6. Wordt afgenomen lichaamsmateriaal na afloop van het onderzoek vernietigd?
○ ja
○ nee (motiveer)
◉ niet van toepassing

F7. Kunnen proefpersonen na afloop van het onderzoek opnieuw benaderd worden (bijvoorbeeld voor nader onderzoek of follow-up)?
○ ja
◉ nee

G. Sectie - Financieel

G1. Door welke geldstroom wordt het onderzoek gefinancierd?

☐ eerste geldstroom (Geld van Ministerie van OC&W aan universiteiten)
☐ tweede geldstroom (NWO of KNAW), namelijk

☑ derde geldstroom (anders dan 1e of 2e geldstroom, zoals collectebusfondsen, Europese Unie, vakministeries of bedrijven), namelijk
KWF Kankerbestrijding

G2. Wordt het onderzoek (mede) gefinancierd door de industrie/bedrijven?
○ ja - door de industrie/bedrijf zoals is opgegeven bij vraag B6/B7 (opdrachtgever van het onderzoek)
○ ja - (ook) door andere industrie/bedrijven dan de opdrachtgever
◉ nee

G3. Wat is de hoogte van de vergoeding die de arts/onderzoeker cq onderzoeksafdeling/maatschap ontvangt voor de uitvoering van het onderzoek?
○ Per patiënt of proefpersoon
◉ Per deelnemend centrum
bedrag afgerond in hele euro's: € 100,00

G3a. Hoe is de vergoeding opgebouwd?
De vergoeding betreft een financiële compensatie van € 100 excl. BTW als opstartvergoeding voor ieder deelnemend centrum.

G4. Heeft/hebben de onderzoeker(s) gedurende de afgelopen vijf jaar op een of andere wijze een persoonlijke financiële relatie (gehad) met de verrichter/sponsor van het huidige onderzoek?
○ ja (licht toe)
◉ nee

I. Sectie - Indiening en beoordeling

I1. Sla het formulier eerst op en selecteer vervolgens een METC of de CCMO
Commissie
METC NedMec

J. Sectie - Aanvullende opmerkingen

Aanvullende opmerkingen

K. Sectie - Samenvatting

Achtergrond van het onderzoek:

De schildwachtklierprocedure is de standaard methode voor lymfeklierstadiëring in borstkankerpatiënten met een klinisch negatieve okselklierstatus. In de algemene bevolking bevat de schildwachtklier in ongeveer 25% een uitzaaiing. Dit percentage is lager in patiënten met een kleine borsttumor en in borstsparend behandelde patiënten. De ACOSOG-Z0011 studie en de IBCSG 23-01 studie hebben recent aangetoond dat een completerende okselklierdissectie veilig achterwege gelaten kan worden bij borstsparend en met systemische therapie behandelde patiënten met een beperkt aantal schildwachtkliermetastasen. Dit roept de vraag op of de schildwachtklierprocedure nog wel noodzakelijk is voor borstsparend behandelde patiënten met een klinisch negatieve okselklierstatus. De klinisch negatieve okselklierstatus van de patiënten in de beschreven studies werd gesteld op basis van lichamelijk onderzoek. Het toevoegen van een preoperatieve echografie van de oksel zal zorgen voor een betere voorselectie van patiënten zonder lymfekliermetastasen, ten opzichte van lichamelijk onderzoek alleen. Daarbij sluit een negatieve echografie uitgevoerd in Nederland bijna alle patiënten uit met uitgebreide lymfekliermetastasen (≥4 lymfekliermetastasen). Bovendien omvat gehele borstbestraling na de borstsparende operatie in het algemeen het lagere deel van de oksel en behandelt daarmee eventueel achtergebleven uitzaaiingen. Hoewel de schildwachtklierprocedure minder invasief is vergeleken met een okselklierdissectie, komen complicaties nog steeds voor. Zo lijdt 6% van de patiënten die alleen een schildwachtklierprocedure ondergingen aan ernstige lymfoedeem na slechts 3 jaar. Deze studie gaat onderzoeken of de schildwachtklierprocedure veilig achterwege gelaten kan worden bij borstsparend behandelde patiënten, met een negatieve echografie van de oksel.

Doel van het onderzoek:

Het doel van deze studie is het verminderen van overbehandeling van de oksel bij patiënten met borstkanker en een klinisch negatieve okselklierstatus die behandeld worden met borstsparende therapie, door het achterwege laten van de schildwachtklierprocedure, om zodoende de kans op korte en lange termijn complicaties te verminderen en te zorgen voor een verbetering van de kwaliteit van leven met een even goede ziektecontrole en algehele overleving.

Onderzoeksopzet:

Het betreft een gerandomiseerde non-inferiority multicenter onderzoek. Patienten die voldoen aan de in- en exclusiecriteria worden na toestemming middels loting behandeld volgens één van de volgende behandelmethoden:

- Behandelmethode A (controle groep): schildwachtklierprocedure volgens de Nederlandse borstkankerrichtlijn.
- Behandelmethode B (studie groep): geen verdere okselklierstadiëring.

Stratificatiefactoren: leeftijd (≤50, 50≤75, >75 jaar), oestrogeen receptor status (positief vs. negatief), HER2neu status (geamplificeerd vs. niet geamplificeerd), klinisch tumor stadium (<3 cm vs. ≥3 cm), tumorgraad volgens gemodificeerde Bloom-Richardson (graad I-II vs. graad III), is of zal worden behandeld met primaire systemische therapie (ja/nee), participerend centrum.
Overige behandelingen (o.a. hormonale therapie, chemotherapie) worden op indicatie gegeven volgens de Nederlandse borstkankerrichtlijn.
Tijdens de follow-up periode van 10 jaar wordt de oksel nauwkeurig onderzocht middels lichamelijk onderzoek en echografie op indicatie. Indien sprake is van een cyto-/histologisch bewezen lymfekliermetastase tijdens follow-up, dan kan in het multidisciplinair overleg van de desbetreffende patiënt worden besloten tot het uitvoeren van een vorm van okselklierbehandeling.
Gevalideerde vragenlijsten worden verstrekt voor het onderzoeken van de invloed op het aantal complicaties en kwaliteit van leven (Lymph-ICF, QLQ-C30, QLQ-BR23) en om te meten of angst en persoonlijkheidstrekken invloed hebben op de uitkomst van kwaliteit van leven (STAI-trait, NEO-FFI). Deze vragenlijsten worden verstrekt tot aan 1056 van de 1644 patiënten (528 per arm), éénmaal voor de randomisatie en vijfmaal na de randomisatie (6 maanden, 1 jaar, 2 jaar, 5 jaar en 10 jaar). Vragenlijsten met betrekking to kosten-effectiviteit worden éénmaal voor de randomisatie en viermaal na de randomisatie (6 maanden, 1,2 en 3 jaar) afgenomen.

Onderzoekspopulatie:

Vrouwen van 18 jaar of ouder met pathologisch bewezen invasief mammacarcinoom die borstsparende therapie ondergaan.
cT1-2: primaire tumor is bij klinisch onderzoek kleiner dan 5 cm.
cN0: bij lichamelijk onderzoek en echografie van de oksel worden geen aanwijzingen voor lymfekliermetastasen gevonden of de cytologie/histologie na punctie/biopt van een lymfeklier is negatief.
Neoadjuvante systemische therapie is toegestaan indien de patiënt voor de start van de neoadjuvante systemische therapie een klinische T1-2N0 status heeft en waarvoor borstsparende therapie op dat moment al geschikt is.
De patiënt heeft informed consent persoonlijk ondertekend.

Interventie (indien van toepassing):

De controlegroep ondergaat wél de schildwachtklierprocedure volgens de Nederlands borstkankerrichtlijn. De experimentele arm ondergaan géén schildwachtklierprocedure.

Primaire onderzoeksvariabelen/uitkomstmaten:

Primaire uitkomstmaat:
- Regionaal recidief percentage

Secundaire onderzoeksvariabelen/uitkomstmaten (indien van toepassing):
Secundaire uitkomstmaten:
- Distant-disease free survival

- Overall survival
- Later uitgevoerde okselbehandeling
- Lokaal recidief percentage
- Overig-regionaal recidief percentage
- Contralaterale borstkanker
- Percentage (neo)adjuvante systemische therapie
- Kwaliteit van leven en okselbehandeling-gerelateerde morbiditeit
- Kosten-effectiviteit

Omschrijving en inschatting van belasting en risico (indien van toepassing):

Wat betreft belasting is er in deze studie géén sprake van extra diagnostiek of interventies (juist minder) en hoeft de patiënt niet vaker dan normaal voor controle naar het ziekenhuis. De eventuele extra belasting voor de patiënt zal alleen bestaan uit het invullen van kwaliteit van leven vragenlijsten (bij 1056 van 1644 patiënten) éénmaal voor de randomisatie en vijfmaal na randomisatie (6 maanden, 1 jaar, 2 jaar, 5 jaar en 10 jaar). Vragenlijsten met betrekking to kosten-effectiviteit worden éénmaal voor de randomisatie en viermaal na de randomisatie (6 maanden, 1,2 en 3 jaar) afgenomen.
De hypothese luidt dat het achterwege laten van de schildwachtklierprocedure even veilig is als het wél uitvoeren van deze procedure. Desondanks is er een risico dat in de experimentele arm meer regionale recidieven optreden en dat bij patiënten op een later tijdstip alsnog een vorm van okselklierbehandeling moet worden uitgevoerd. Daartegenover staat dat er in de experimentele arm door het niet uitvoeren van de schildwachtklierprocedure minder kans is op morbiditeit, minder operaties en opnames in het ziekenhuis en een betere kwaliteit van leven. Patiënten zullen op geen enkele manier vertraging ondervinden als gevolg van deelname aan de studie. Verder wordt de kosteneffectiviteit onderzocht van het achterwege laten van de schildwachtklierprocedure.

K2.Engelse Samenvatting

Background of the study:
The NSABP B-04 trial revealed that omitting primary axillary treatment of occult positive lymph nodes in clinically node negative breast cancer patients does not affect survival, even after 25 years of follow-up and without the use of adjuvant systemic or radiation therapy. A delayed axillary dissection in case lymph nodes become clinically positive does not affect survival and prevents axillary overtreatment in the majority of patients. The ACOSOG Z0011 and IBCSG 23-01 trials revealed that completion axillary dissection can be safely omitted in clinically node negative patients with metastatic sentinel nodes. Patients randomized for watchful waiting were likely to have residual nodal disease (13-27%) that was not surgically removed. Nevertheless, survival rates were not affected and regional recurrence rates low. Use of axillary ultrasound will improve the preoperative selection of node negative patients, as it selects patients with a more favourable tumour load and accurately excludes advanced nodal disease (≥4 metastatic nodes). Biology and adjuvant systemic and radiation therapy are all factors that most likely diminish the risk that possible metastases left in situ develop into clinically detectable lymph nodes. Patients treated with breast conserving therapy are significantly more often diagnosed with pathologic node negative disease or with micrometastatic disease, compared to patients treated with mastectomy. The risk for occult (macro)metastases in cT1-2N0 breast cancer patients treated with breast conserving therapy is low: about 88% will be node negative and about 95% of node positive patients will have no lymph node metastases beyond the sentinel node. Reflected by the low regional recurrence rate after a (false) negative sentinel node, only a small amount of patients (0.8%) with node positive disease is expected to develop clinically detectable nodal disease.

Objective of the study:
The objective of this study is to decrease the number of breast cancer patients receiving overtreatment of the axilla, in order to positively influence the axillary morbidity rate and quality of life. Therefore, we investigate whether the sentinel lymph node procedure can be safely omitted in clinically node negative breast cancer patients undergoing breast conserving therapy, in terms of non-inferior regional control, distant-disease free and overall survival. All objectives are measured during a follow-up of 10 years.

Study design:
A randomized controlled non-inferiority multicenter study. Patients who meet the in- and exclusioncriteria will be randomized after informed consent for one of the two treatment methods:
- Arm A (control arm): lumpectomy with sentinel lymph node procedure, followed by radiotherapy of the breast with or without completion axillary treatment according to the Dutch breast cancer guideline.
- Arm B (experimental arm): lumpectomy without further axillary staging, followed by radiotherapy of the breast.

Stratification: age (≤50, 50≤75, >75 years old), oestrogen receptor status (positive vs. negative), HER2neu status (amplified vs. not amplified), clinical tumour stage prior to any treatment (<3 cm vs. ≥3 cm), grading (grade I-II vs. III - according to Bloom-Richardson grading system), is or will be treated with primary systemic therapy (yes/no) and participating centre.
Adjuvant systemic treatment is administered if indicated according to the Dutch breast cancer guideline.
Primary systemic therapy is allowed if the patient has a clinical T1-2N0 status (initial stage) that is amenable to lumpectomy pre-systemic therapy.
Yearly follow-up with physical examination of the axilla for 10 years. Axillary ultrasound (+/- tissue sampling) on indication. Staging for distant metastases and/or a delayed axillary lymph node dissection if indicated by the multidisciplinary team. Validated questionnaires are used to assess axillary morbidity rate and quality of life (Lymph-ICF, QLQ-C30, QLQ-BR23) and to measure if anxiety and personality traits influence the outcome of quality of life (STAI-trait, NEO-FFI) in 1056 of 1644 patients. These questionnaires are provided before randomization and sequentially post randomization at 6 months, 1, 2, 5 and 10 years. Cost-effectiveness questionnaires are provided before randomization and sequentially post randomization at 6 months, 1, 2 and 3 years.

Study population:
Women aged 18 years or older with pathologically confirmed invasive breast carcinoma about to undergo breast conserving surgery followed by radiotherapy of the breast.
cT1-2: primary tumour is smaller than 5 cm.
cN0: no signs of axillary lymph node metastases at physical examination and axillary ultrasound (+/- tissue sampling).
Primary systemic treatment is allowed.
Patient is able to and signed the informed consent form.

Intervention (if applicable):
Patients in the control arm will be treated with the sentinel lymph node procedure according to the Dutch breast cancer guideline. Patients in the experimental arm will not be treated with the sentinel lymph node procedure.

Primary study parameters/outcome of the study:
Primary endpoint:
- Regional recurrence rate

Secundary study parameters/outcome of the study (if applicable):
Secondary endpoints:
- Distant-disease free survival
- Overall survival
- Delayed axillary treatment
- Local recurrence rate
- Other-regional recurrence rate
- Contralateral breast cancer rate
- Percentage difference in the administration of (neo)adjuvant systemic therapy
- Quality of life and axillary morbidity rate
- Cost-effectiveness

Nature and extent of the burden and risks associated with participation, benefit and group relatedness (if applicable):
There is no extra burden for participating patients as regard to additional diagnostic tests/interventions and follow-up. Any additional burden for the patient will only consist of completing quality of life questionnaires in 1056 of 1644 study patients. These questionnaires are provided before randomization and sequentially post randomization at 6 months, 1, 2, 5 and 10 years. The cost effectiveness analysis study (CEA) was set up later and added to the Qol-study. The inclusion rate of the CEA was 66%. Since this sub-study needed to include 474 patients, still another 74 patients need tob e approached until a total of 1056 patients.
If patients do not return the questionnaires or only one out of the first two, a letter to thank the patient for taking part in the study. They will not be bothered with further questionnaires. After patient 1056 no questionnaires will be sent out.
We hypothesize that omitting the sentinel lymph node procedure is not inferior to the standard treatment. Nevertheless, possible disadvantages are a worse regional recurrence rate, and delayed axillary treatment if indicated. Patients in the experimental arm have the possible advantages of less morbidity, fewer operations and hospitalizations and an improved quality of life, compared to patients treated with the sentinel lymph node procedure.
No patient will encounter any delay in their treatment as a result of inclusion.

ONDERTEKENING

De verrichter en indiener verklaren hierbij:
a. het formulier (en samenvatting) volledig en naar waarheid te hebben ingevuld;
b. de antwoorden op de vragen uit het ABR-formulier niet in strijd zijn met het bijbehorende onderzoeksdossier en onderzoekscontract

Naar waarheid getekend, door de verrichter (=opdrachtgever)		door de indiener	
datum	....	datum	....
Handtekening		Handtekening	
naam	U.P. Neumann	naam	F. van Duijnhoven
functie	Hoofd afdeling Chirurgie, MUMC+	functie	Chirurg, NKI-AVL

Sluiten Print

Sluiten Print

Formulier voor medisch-ethische beoordeling en registratie

ABR-formulier, versie mei 2021

Onderzoeksdossiernummer

ABR Nummer	**49315**	Status	**Beoordeeld - Positief**
Versie	**13**	Status per	**31-08-2022**
Jaar	**22**		
Dossiernummer	**NL49315.031.14**		
Reden voor	**5% protocol violations**		

A. Sectie - Openbaar maken gegevens medisch wetenschappelijk onderzoek

A1. Het CCMO-register is een voor ieder toegankelijk openbaar trial register. De antwoorden op de vragen gemarkeerd met een wereldbol en de samenvatting bij dit formulier worden openbaar gemaakt in het CCMO register.

B. Sectie - Administratief

B1. Betreft het onderzoek met geneesmiddelen (inclusief gentherapie, somatische celtherapie, vaccinonderzoek, GGO's, zie verdere toelichting) als bedoeld in de Wet medisch-wetenschappelijk onderzoek met mensen (WMO)?

○ ja
◉ nee

B2. Houdt het onderzoek verband met een eerder door een erkende METC of door de CCMO beoordeelde studie of is het onderzoek reeds eerder bij een erkende METC ter beoordeling voorgelegd?

○ ja, het onderzoek houdt verband met – of is het vervolg op – een eerder beoordeelde studie
○ ja, het onderzoek is eerder ter beoordeling aan een erkende METC of de CCMO voorgelegd (stuur kopie besluit mee)
◉ nee

B4. Is het protocol (nog) in een ander openbaar trial register geregistreerd?

○ ja
◉ nee

B5. Naam indiener/contactpersoon voor de oordelende toetsingscommissie

**B5a.**	Achternaam indiener/contactpersoon	Duijnhoven
	Titel en voorletters	dr. F.
	Tussenvoegsels	van
**B5b.**	Type organisatie/bedrijf	Overige Ziekenhuizen
	Organisatie/bedrijf	Antoni van Leeuwenhoek Ziekenhuis
	Naam organisatie/bedrijf	IKNL trialbureau
	Afdeling	Chirurgie
	Adres	Plesmanlaan 121
	Postcode en plaats	1066 CX Amsterdam
	Land	NL
**B5c.**	Intern adres	afdeling Heelkunde
	Telefoon	020 512 9111
	Fax	020 512 2554
	E-mail	f.v.duijnhoven@nki.nl

B6. Is de indiener werkzaam bij de opdrachtgever/sponsor (verrichter) van het onderzoek?

○ Ja ◉ Nee

B7. Opdrachtgever/sponsor van het onderzoek (verrichter volgens de WMO)

Type Organisatie/Bedrijf	Universitair Medisch Centrum
Organisatie/Bedrijf	Medisch Universitair Ziekenhuis Maastricht

Adres P. Debyelaan 25
Postcode en plaats 6229 HX Maastricht
Land NL
Telefoon 0433877477
Fax 0433875473
E-mail m.smidt@mumc.nl

C. Sectie - Onderzoek

C1. Volledige titel van het onderzoek

C1a. In het Engels

Clinically node negative breast cancer patients undergoing breast conserving therapy: Sentinel lymph node procedure versus follow-up. A Dutch randomized controlled multicentre trial.

C1b. In het Nederlands

Patiënten met borstkanker en een klinisch negatieve okselklierstatus die een borstsparende behandeling ondergaan: Schildwachtklierprocedure versus follow-up. Een Nederlandse gerandomiseerde multicenter studie.

C2. Verkorte titel van het onderzoek/acroniem

C2a. In het Engels

BOOG 2013-08

C2b. In het Nederlands (Let op: deze korte titel wordt vermeld binnen ToetsingOnline)

BOOG 2013-08

C3. Trefwoorden (maximaal 4, plaats elk trefwoord op een aparte regel)

C3a. In het Engels

Breast neoplasms
Breast conserving therapy
Sentinel lymph node biopsy
Axilla

C3b. In het Nederlands

Borstkanker
Borstsparende behandeling
Schildwachtklierprocedure
Oksel

C4. Beschrijf het belang van het onderzoek en de beoogde toepassing van de resultaten (verwijs eventueel naar de relevante pagina's in het protocol).

Het doel van dit onderzoek is om:
1) Overbehandeling van de oksel te verminderen;
2) Het aantal korte en lange termijn complicaties te verminderen;
3) De kwaliteit van leven te verbeteren;
4) Met een even goede ziektecontrole en algehele overleving

Dit willen wij bereiken door te onderzoeken of de schildwachtklierprocedure veilig achterwege gelaten kan worden bij patiënten met borstkanker en een klinisch negatieve okselklierstatus behandeld met borstsparende therapie.

C6. Betreft het onderzoek een multicenter-onderzoek?

○ nee
◉ ja - alleen in Nederland
○ ja - internationaal binnen de Europese Unie
○ ja - internationaal ook buiten de Europese Unie

C7. Is er bij multicenter-onderzoek sprake van een coördinerend onderzoeker?

◉ ja namelijk — Naam onderzoeker: M.L. Smidt
○ nee

C8. Wie is/zijn medisch verantwoordelijk voor de proefpersonen die deelnemen aan het onderzoek

Behandelend arts en lokale hoofdonderzoeker

C9. In welk centrum/welke centra (incl. huisartsenpraktijken) in Nederland wordt het onderzoek uitgevoerd?

Centrum	Proefpersonen	Hoofdonderzoeker	Onafhankelijk arts
Amphia Ziekenhuis	69	E.J.T. Luiten	S.O. Breukink (Maastricht UMC+)
Zuyderland Medisch Centrum	39	E.R.M. van Haaren	S.O. Breukink (Maastricht UMC+)

Canisius Wilhelmina Ziekenhuis	46	L.J.A. Strobbe	S.O. Breukink (Maastricht UMC+)
Catharina-ziekenhuis	46	G.A.P. Nieuwenhuijzen	S.O. Breukink (Maastricht UMC+)
Deventer Ziekenhuis	39	H. de Vries	S.O. Breukink (Maastricht UMC+)
Diakonessenhuis Utrecht	39	J.Volders	S.O. Breukink (Maastricht UMC+)
Gelre Ziekenhuizen	37	J.H.G. Klinkenberg	S.O. Breukink (Maastricht UMC+)
HagaZiekenhuis	41	I. Jannink	S.O. Breukink (Maastricht UMC+)
Isala Klinieken	54	A.B. Francken	S.O. Breukink (Maastricht UMC+)
Laurentius Ziekenhuis	23	A.V.R.J. Bell	S.O. Breukink (Maastricht UMC+)
Academisch Ziekenhuis Maastricht	39	M.L. Smidt	S.O. Breukink (Maastricht UMC+)
Maxima Medisch Centrum	31	K. Schenk	S.O. Breukink (Maastricht UMC+)
Antoni van Leeuwenhoek Ziekenhuis	93	F. van Duijnhoven	S.O. Breukink (Maastricht UMC+)
Universitair Medisch Centrum Sint Radboud	31	J.H.W. de Wilt	S.O. Breukink (Maastricht UMC+)
Sint Antonius Ziekenhuis	58	R. Koelemij	S.O. Breukink (Maastricht UMC+)
Tergooiziekenhuizen locatie Hilversum	42	E.J.C. Vriens	S.O. Breukink (Maastricht UMC+)
Universitair Medisch Centrum Groningen	17	J. de Vries	S.O. Breukink (Maastricht UMC+)
Universitair Medisch Centrum Utrecht	23	A.J. Witkamp	S.O. Breukink (Maastricht UMC+)
Viecuri Medisch Centrum voor Noord-Limburg	39	P.H.A Nijhuis	S.O. Breukink (Maastricht UMC+)
Ziekenhuisvoorzieningen Gelderse Vallei	32	W.K. de Roos	S.O. Breukink (Maastricht UMC+)
Rivierenland Ziekenhuis	23	M.F. Sier	S.O. Breukink (Maastricht UMC+)
Noordwest Ziekenhuisgroep	54	K.M. Blaauwendraat	S.O. Breukink (Maastricht UMC+)
Rijnstate Ziekenhuis	62	R. van Eekeren	S.O. Breukink (Maastricht UMC+)
Jeroen Bosch Ziekenhuis	20	M. Bessems	B. Biesma (Jeroen Bosch ziekenhuis)
Alrijne ziekenhuis	50	P. Neijenhuis	S.O. Breukink (Maastricht UMC+)
Spaarne Gasthuis	30	B. Kortmann	S.O. Breukink (Maastricht UMC+)
St. Jansdal	40	Schouten van der Velden	S.O. Breukink (Maastricht UMC+)
Treant Zorggroep	50	L. Smit	S.O. Breukink (Maastricht UMC+

C10. Betreft het onderzoek met:

- [x] mensen
- [ ] geslachtscellen
- [ ] (rest-)embryo's
- [ ] foetussen in utero

C10a. Indien mensen aangevinkt (meerdere antwoorden mogelijk):

- [ ] preterme pasgeborenen (< 37 weken zwangerschap)
- [ ] pasgeborenen (0-27 dagen)
- [ ] babies en peuters (28 dagen – 23 maanden)
- [ ] kinderen (2-11 jaar)
- [ ] jongeren (12-15 jaar)
- [ ] adolescenten (16-17 jaar)
- [x] volwassenen (18-64 jaar)
- [x] ouderen (65 jaar en ouder)
- [ ] zwangere vrouwen
- [ ] vrouwen die borstvoeding geven

C11. Beoogd totaal aantal proefpersonen/(rest)embryo's/foetussen in utero:
C11a. In Nederland
1730

C13. Onderzoeksgebied

- ☐ etiologie
- ☐ organisatorisch/zorgonderzoek
- ☐ diagnostiek
- ☐ preventie
- ☑ therapie
- ☐ veiligheid
- ☐ werkzaamheid
- ☐ farmacokinetiek
- ☐ farmacodynamiek
- ☐ bio-equivalentie
- ☐ dosis-respons
- ☐ farmacogenomics
- ☐ farmaco-economie
- ☐ anders

C14. Type onderzoek

- ○ observationeel onderzoek zonder invasieve metingen
- ○ observationeel onderzoek met invasieve metingen
- ◉ interventie-onderzoek

C15. In welke fase kan het onderzoek worden ingedeeld?

- ○ fase I (a)
- ○ fase II (b)
- ◉ fase III (c)
- ○ fase IV (d)
- ○ overige onderzoeken waarbij geneesmiddelen worden toegepast (e)
- ○ niet van toepassing

C17. Is er sprake van een ander(e) onderzoeksproduct en/of interventie dan vermeld bij vraag C16 (zie toelichting, kan zowel voor observationeel als interventie-onderzoek worden ingevuld als het product (verder) onderzocht wordt.)

- ◉ Ja
- ○ Nee

C17a. ☐ Medisch hulpmiddel (medisch hulpmiddel, actief implantaat of in-vitro diagnosticum), namelijk
C17b. ☐ Operatie, namelijk
C17c. ☐ Psychosociale interventie, namelijk
C17d. ☐ Voeding(stoffen), namelijk
C17e. ☐ Bewegingstherapie, namelijk
C17f. ☐ interventie met radioactieve straling, namelijk
C17g. ☐ Blootstellingsonderzoek (bijv pesticidenonderzoek), namelijk
C17h.
C17i. ☑ andere interventie, namelijk

In het Engels: No sentinel lymph node procedure
In het Nederlands: Géén schildwachtklierprocedure

C18. Worden de onderzoeksproducten voor deze studie door de verrichter gratis verstrekt?

- ○ ja
- ○ nee
- ○ gedeeltelijk namelijk
- ◉ niet van toepassing

C19. Is/zijn er (een) controlegroep (en)?

- [x] ja
- [ ] nee

- [ ] ja, geneesmiddel
- [ ] ja, placebo
- [ ] ja, geen interventie
- [x] ja, anders

namelijkDe standaard behandeling: schildwachtklierprocedure

C20. Betreft het een gerandomiseerd onderzoek?

- [x] ja
- [ ] nee

namelijk

- [x] open
- [ ] enkelblind
- [ ] dubbelblind
- [ ] parallel
- [ ] cross-over
- [ ] anders namelijk

C21. Op welke klasse(n) van aandoeningen heeft het onderzoek betrekking (maximaal 3)

- [ ] hartaandoeningen
- [ ] congenitale, familiaire en genetische aandoeningen
- [ ] bloed- en lymfestelsel aandoeningen
- [ ] zenuwstelsel aandoeningen
- [ ] oogaandoeningen
- [ ] evenwichtsorgaan- en ooraandoeningen
- [ ] ademhalingsstelsel-, thorax- en mediastinumaandoeningen
- [ ] maagdarmstelselaandoeningen
- [ ] nier- en urinewegaandoeningen
- [ ] huid- en onderhuidaandoeningen
- [ ] skeletspierstelsel- en bindweefselaandoeningen
- [ ] endocriene aandoeningen
- [ ] voedingsstoornissen en metabole ziekten
- [ ] infecties en parasitaire aandoeningen
- [ ] letsels, intoxicaties en verrichtingscomplicaties
- [x] neoplasmata, benigne, maligne en niet-gespecificeerd (incl cysten en poliepen)
  borstneoplasmata maligne en niet-gespecificeerd (incl. tepel)
- [x] chirurgische en medische verrichtingen
  borst therapeutische verrichtingen
- [ ] bloedvataandoeningen
- [ ] algemene aandoeningen en aandoeningen op de plek van toediening
- [ ] zwangerschap, perinatale periode en puerperium
- [ ] sociale omstandigheden
- [ ] immuunsysteemaandoeningen
- [ ] lever- en galaandoeningen
- [ ] voortplantingsstelsel- en borstaandoeningen
- [ ] psychische stoornissen
- [ ] overig, namelijk

C22.Geef twee synoniemen voor de aandoening die bestudeerd wordt, waarvan tenminste één lekenterm.

In het Engels:	breast cancer, sentinel lymph nodes, sentinel lymph node metastases
In het Nederlands:	borstkanker, schildwachtklieren, schildwachtkliermetastasen

C23. Beoogde start- en einddatum van het onderzoek

C23a. Start Datum (dd-mm-jjjj) 01-01-2015
C23b. Eind Datum (dd-mm-jjjj) 01-01-2027

C24. Zijn patiënten betrokken bij het ontwikkelen van het onderzoek? (Voor patiënten lees ook: patiëntenorganisaties, naasten of nabestaanden van patiënten en gezonde vrijwilligers)?

C30. Wordt de ervaring van proefpersonen na deelname aan het onderzoek geëvalueerd?

D. Sectie - Proefpersonen

D1. Is er een proefpersonenverzekering conform de WMO-eisen afgesloten of wordt aan de oordelende toetsingscommissie ontheffing gevraagd?

- (●) proefpersonenverzekering is afgesloten bij verzekeringsmaatschappij
- ( ) ontheffing van de verzekering wordt gevraagd
- ( ) niet van toepassing, het onderzoek valt onder de Embryowet en er is geen sprake van proefpersonenn

Verzekeringsmaatschappij Marketform (onderdeel van Lloyd's)

D2. Gezonde proefpersonen en/of patiënten

- [ ] Gezonde proefpersonen
- [x] Patiënten

Aantal 1736

D4. Voornaamste inclusiecriteria
D4a. In het Engels
- Female
- Aged 18 years or older
- Pathologically confirmed invasive unilateral breast carcinoma
- A clinical T1-2 tumour (≤ 5cm)
- Will be treated with lumpectomy followed by whole breast radiotherapy
- Clinically node negative status: no signs of axillary lymph node metastases at physical examination and preoperative axillary ultrasound (or negative cyto-/histopathology)
- Written informed consent

D4b. In het Nederlands
- Vrouw
- Leeftijd 18 jaar of ouder
- Pathologisch bewezen invasief unilaterale borstkanker
- Klinisch T1-2 tumor (≤ 5cm)
- Zal worden behandeld met lumpectomie gevolgd door gehele borstbestraling
- Klinisch negatieve okselklierstatus: negatief lichamelijk onderzoek en echografie van de oksel (of negatieve cyto-/histologie)
- Schriftelijke informed consent

D5. Voornaamste exclusiecriteria
D5a. In het Engels
- Clinically node positive pre-operative
- Bilateral breast cancer
- Evidence of metastatic disease
- History of invasive breast cancer
- Previous treatment of the ipsilateral axilla with surgery or radiotherapy, except surgery for hidradenitis suppurativa or for other superficially located skin lesions, such as naevi
- Pregnant or nursing
- Other prior malignancies within the past 5 years, except successfully treated malignancies that occurred more than five years before randomization, and except successfully treated basal cell and squamous cell skin cancer, carcinoma in situ of the cervix or carcinoma in situ of the ipsilateral or contralateral breast
- Unable or unwilling to give informed consent

D5b. In het Nederlands
- Klinisch positieve okselklierstatus (preoperatief)
- Bilaterale borstkanker
- Primair (afstands-)gemetastaseerde borstkanker
- Invasieve borstkanker in de voorgeschiedenis
- Chirurgie of radiotherapie van ipsilaterale oksel in de voorgeschiedenis, behalve chirurgie voor hidradenitis suppurativa of andere oppervlakkig gelegen huidlaesies, zoals naevi
- Zwanger of lacterend
- Andere maligniteit in de afgelopen 5 jaar, behalve succesvol behandelde maligniteit die meer dan vijf jaar voor randomisatie optrad, en behalve succesvol behandelde huidkanker van type basaalcel of plaveiselcel, carcinoma in situ van de cervix, carcinoma in situ van de ipsilaterale of contralaterale mamma
- Niet in staat of niet bereid tot informed consent

D6. Bij welke categorie proefpersonen wordt het onderzoek uitgevoerd (meerdere antwoorden mogelijk)

- [x] 16 jaar of ouder en wilsbekwaam (ga naar vraag D10)
- [ ] 16 jaar of ouder en wilsonbekwaam (ga naar vraag D7)
- [ ] 12 t/m 15 jaar en in staat tot het geven van geïnformeerde toestemming (ga naar vraag D8)
- [ ] 12 t/m 15 jaar en niet in staat tot het geven van geïnformeerde toestemming (wilsonbekwaam) (ga naar vraag D7)
- [ ] jonger dan 12 jaar (ga naar vraag D8)

D10. Verkeren (sommige) proefpersonen in een afhankelijkheidssituatie ten opzichte van de onderzoeker of degene die de deelnemers werft? (lees de toelichting voor voorbeelden wanneer er sprake kan zijn van een afhankelijkheidssituatie)

- (●) ja
- ( ) nee

D10a. Zo ja, waardoor?
Soms is de lokale onderzoeker ook de behandelend arts die de patiënt vraagt om deel te nemen aan de studie.

D10b. Waarom wordt het onderzoek juist met deze proefpersonen uitgevoerd en hoe worden de belangen van de proefpersonen gewaarborgd?
Het onderzoek moet met proefpersonen worden uitgevoerd die voldoen aan de beschreven in/exclusie criteria zodat de resultaten van de studie uiteindelijk ook naar de kliniek vertaald kunnen worden binnen deze patiëntenpopulatie. De belangen van de proefpersonen worden gewaarborgd door de geldende medisch ethische normen en richtlijn zoals vermeld in het protocol (o.a. onafhankelijk arts, uitgebreide patiëntinformatiefolder).

D11. Waaruit bestaat de vergoeding voor de proefpersonen?

- [x] geen vergoeding
- [ ] reiskosten
- [ ] financiële vergoeding (in Euro's)
- [ ] andere vergoeding

D12. Is deze vergoeding afhankelijk van bepaalde voorwaarden, bijvoorbeeld het voltooien van (een deel van) het onderzoek?

- ( ) ja (motiveer)
- ( ) nee
- (●) niet van toepassing

E. Sectie - Voor- en nadelen

E1. Wordt er bij dit onderzoek een rechtstreeks therapeutisch effect beoogd bij de proefpersonen / patiënten?

- (●) ja (therapeutisch onderzoek)
- ( ) nee (niet-therapeutisch onderzoek)

E1a. Zo ja, geef kort aan waaruit dit therapeutisch effect bestaat.
Het therapeutische effect bestaat uit:
- Het verlagen van het risico op okselbehandeling-gerelateerde korte en lange termijn complicaties;
- Het verbeteren van de kwaliteit van leven;
- Met een even goede ziektecontrole en algehele overleving.

E2. Waaruit bestaat de belasting van het onderzoek (en een eventueel daaraan voorafgaande keuring) voor de proefpersonen?

Tijdbeslag	per bezoek	20 min per keer voor invullen kwaliteit van leven vragenlijst
	totaal	20 min x 6 = 120 min
	totale duur van de studie voor de individuele proefpersoon	Tav de behandeling minder belasting dan normaal voor de studie-arm (geen schildwachtklierprocedure). Voor beide armen is extra het invullen van de kwaliteit van leven vragenlijst prerandomisatie en postrandomisatie op 5 momenten.

E3. Worden de proefpersonen in verband met het onderzoek in het ziekenhuis opgenomen of wordt een opname verlengd?

- ( ) ja - het verblijf in het ziekenhuis/instituut wordt in verband met het onderzoek verlengd
- ( ) ja - ze worden voor het onderzoek in het ziekenhuis/instituut opgenomen
- (●) nee

E4. Beschrijf in hoeverre proefpersonen worden onderworpen aan handelingen dan wel een gedragswijze krijgen opgelegd, zoals vragenlijst, interviews, lichamelijk/psychologisch onderzoek, ontzegging, dieet (voor invasieve ingrepen: zie vraag E6)

Initieel zouden 700 proefpersonen (350 in de experimentele en 350 in de controlegroep) deelnemen aan het kwaliteit van leven onderzoek, bestaande uit een vragenlijst voor de randomisatie en op 5 tijdstippen na de randomisatie (na een half jaar, één, twee, drie en vijf jaar).
Later werd een kosteneffectiviteitsstudie (KEA) gestart en gekoppeld aan de kwaliteit van leven studie. Het inclusiepercentage van de KEA bedraagt 66%. Voor deze sub-studie dienen ten minste 474 patiënten geïncludeerd te worden. Bij dit inclusiepercentage, dienen er nu nog 74 patiënten benaderd te worden (toelichting: 1056 nodig voor de sub-studie, inmiddels 983 geïncludeerd.)
Gezien het kwaliteit van leven -onderzoek van groot belang is voor de kosteneffectiviteitsstudie, wordt de kwaliteit van leven-vragenlijst dus verlengd uitgestuurd tot de inclusie van de kosteneffectiviteitsstudie volledig is.
Indien patiënten geen of slechts een van de eerste twee vragenlijst retourneren, zal er geen vervolgvragenlijst worden toegestuurd, doch een bedankbrief voor deelname aan de studie.
Na patiënt 1056 worden er zowel geen KEA als Kwaliteit van leven-vragenlijsten meer uitgestuurd.

E5. Worden de proefpersonen getest op bepaalde aandoeningen/condities?

- ○ ja (motiveer)
- ◉ nee

E6. Welke extra (invasieve) ingrepen (anders dan bij de standaard behandeling) moeten de proefpersonen in het kader van het onderzoek ondergaan:

- ☑ Niet van toepassing

E9. Geef aan welke risico's er voor proefpersonen zijn verbonden aan deelname aan het onderzoek.

Hoewel de hypothese luidt dat het achterwege laten van de schildwachtklierprocedure geen nadelige invloed heeft op de lokale controle van de ziekte en de overlevingskans, bestaat er toch een kans dat proefpersonen in de experimentele arm een hoger risico hebben op een recidief in de oksel (regionaal recidief) en daardoor tijdens follow-up alsnog een vorm van okselklierbehandeling moeten ondergaan. Uit onderzoek blijkt dat het uitgesteld uitvoeren van okselklierbehandeling (bijvoorbeeld met okselklierdissectie) géén nadelige gevolgen heeft voor de overleving.

E9a. Geef op grond van uw eigen afweging aan waarom het uitvoeren van het onderzoek, in het licht van de belasting en/of risico's die voor proefpersonen aan deelname verbonden zijn, gerechtvaardigd is?

Het mogelijk hogere risico op een recidief in de oksel (regionaal recidief) weegt op tegen de lagere kans op ernstige okselklierbehandeling-gerelateerde morbiditeit (lymfoedeem, bewegingsbeperking in de schouder, arm- en schouderpijn, verminderd gevoel of zenuwpijn) en de verbetering van kwaliteit van leven.

E10. Indien het onderzoek bij minderjarige en/of wilsonbekwame proefpersonen wordt uitgevoerd en geen direct therapeutisch effect wordt beoogd: waarom kunnen belasting en risico's als minimaal worden beschouwd in vergelijking met de standaard behandeling (verwijs eventueel naar de relevante pagina's in het protocol)?

- ☑ niet van toepassing

E11. Kan de eventuele therapie na beëindiging van het onderzoek worden voortgezet?

- ○ ja (motiveer)
- ○ nee (motiveer)
- ◉ niet van toepassing

E12. Heeft deelname aan het onderzoek voor de proefpersoon tot gevolg dat van de standaardbehandeling of -diagnostiek kan worden afgeweken of deze kan worden uitgesteld?

- ◉ ja
- ○ nee
- ○ niet van toepassing

E12a. Zo ja, waaruit bestaat de afwijking of het uitstel en waarom is afwijking/uitstel

Proefpersonen in de controlegroep ondergaan de standaard behandeling, namelijk schildwachtklierprocedure volgens de Nederlandse borstkankerrichtlijn. Proefpersonen in de experimentele arm ondergaan primair géén schildwachtklierprocedure. Beide groepen ondergaan dezelfde follow-up middels lichamelijk onderzoek en aanvullend onderzoek op indicatie. Indien een proefpersoon zich in de follow-up periode presenteert met lymfekliermetastasen, kan alsnog besloten worden om een vorm van okselklierbehandeling uit te voeren. Deze beslissing wordt gemaakt tijdens het multidisciplinair overleg in het desbetreffende centrum waar de patiënt onder behandeling staat.

F. Sectie - Informatie en privacy

F1. Hoe worden de proefpersonen geworven en door wie (onderzoeker, behandelend arts, andere persoon) wordt de proefpersoon/wettelijke vertegenwoordiger geïnformeerd en om toestemming gevraagd?

Op het moment in de behandeling van borstkanker dat de patiënt voldoet aan de in/exclusiecriteria zal de behandeld arts informatie geven over de mogelijkheid tot deelname aan deze studie. Een schriftelijke patiëntinformatiebrief zal worden meegegeven aan de patiënt. De patiënt krijgt ruim de tijd (ten minste één week) om deze informatie thuis na te lezen en een beslissing te nemen. De patiënt kan haar interesse in deelname

kenbaar maken aan haar behandelend arts bij een volgend polibezoek. De behandelend arts zal haar dan om toestemming vragen voor deelname aan de studie.

F2. Hoeveel bedenktijd krijgen de proefpersonen/wettelijke vertegenwoordigers om te beslissen over deelname?
Zo lang als nodig, met ten minste één week

F3. Wordt de huisarts, behandelend specialist en/of apotheker van de proefpersoon geïnformeerd over diens deelname aan het onderzoek?
- (●) ja (de proefpersoon dient hiervoor toestemming te geven)
- ( ) nee

F4. Worden persoonsgegevens gecodeerd?
- (●) ja
- ( ) nee

F4a. Zo ja, hoe is deze codering opgebouwd?
Alle proefpersonen krijgen een uniek studienummer. Het unieke studienummer betreft een opvolgnummer zonder verdere specifieke codering voor bijvoorbeeld centrum.

F4b. Wie heeft toegang tot de sleutel van deze code?
De behandelend arts en de onderzoeker

F4c. Wie hebben toegang tot de brondocumenten en eventuele andere tot de persoon herleidbare gegevens?
De lokaal datamanagers en de onderzoeker

F5. Hoe wordt het lichaamsmateriaal <u>gedurende</u> het onderzoek bewaard?
- ( ) in tot de proefpersoon herleidbare vorm (gecodeerd)
- ( ) in niet tot de proefpersoon herleidbare vorm (volledig geanonimiseerd)
- (●) niet van toepassing

F6. Wordt afgenomen lichaamsmateriaal na afloop van het onderzoek vernietigd?
- ( ) ja
- ( ) nee (motiveer)
- (●) niet van toepassing

F7. Kunnen proefpersonen na afloop van het onderzoek opnieuw benaderd worden (bijvoorbeeld voor nader onderzoek of follow-up)?
- ( ) ja
- (●) nee

G. Sectie - Financieel

G1. Door welke geldstroom wordt het onderzoek gefinancierd?
- [ ] eerste geldstroom (Geld van Ministerie van OC&W aan universiteiten)
- [ ] tweede geldstroom (NWO of KNAW), namelijk
- [x] derde geldstroom (anders dan 1e of 2e geldstroom, zoals collectebusfondsen, Europese Unie, vakministeries of bedrijven), namelijk
  KWF Kankerbestrijding

G2. Wordt het onderzoek (mede) gefinancierd door de industrie/bedrijven?
- ( ) ja - door de industrie/bedrijf zoals is opgegeven bij vraag B6/B7 (opdrachtgever van het onderzoek)
- ( ) ja - (ook) door andere industrie/bedrijven dan de opdrachtgever
- (●) nee

G3. Wat is de hoogte van de vergoeding die de arts/onderzoeker cq onderzoeksafdeling/maatschap ontvangt voor de uitvoering van het onderzoek?
- ( ) Per patiënt of proefpersoon
- (●) Per deelnemend centrum

bedrag afgerond in hele euro's: € 100,00

G3a. Hoe is de vergoeding opgebouwd?
De vergoeding betreft een financiële compensatie van € 100 excl. BTW als opstartvergoeding voor ieder deelnemend centrum.

G4. Heeft/hebben de onderzoeker(s) gedurende de afgelopen vijf jaar op een of andere wijze een persoonlijke financiële relatie (gehad) met de verrichter/sponsor van het huidige onderzoek?

○ ja (licht toe)

◉ nee

I. Sectie - Indiening en beoordeling

I1. Sla het formulier eerst op en selecteer vervolgens een METC of de CCMO

Commissie
METC NedMec

J. Sectie - Aanvullende opmerkingen

Aanvullende opmerkingen

K. Sectie - Samenvatting

Achtergrond van het onderzoek:

De schildwachtklierprocedure is de standaard methode voor lymfeklierstadiëring in borstkankerpatiënten met een klinisch negatieve okselklierstatus. In de algemene bevolking bevat de schildwachtklier in ongeveer 25% een uitzaaiing. Dit percentage is lager in patiënten met een kleine borsttumor en in borstsparend behandelde patiënten. De ACOSOG-Z0011 studie en de IBCSG 23-01 studie hebben recent aangetoond dat een completerende okselklierdissectie veilig achterwege gelaten kan worden bij borstsparend en met systemische therapie behandelde patiënten met een beperkt aantal schildwachtkliermetastasen. Dit roept de vraag op of de schildwachtklierprocedure nog wel noodzakelijk is voor borstsparend behandelde patiënten met een klinisch negatieve okselklierstatus. De klinisch negatieve okselklierstatus van de patiënten in de beschreven studies werd gesteld op basis van lichamelijk onderzoek. Het toevoegen van een preoperatieve echografie van de oksel zal zorgen voor een betere voorselectie van patiënten zonder lymfekliermetastasen, ten opzichte van lichamelijk onderzoek alleen. Daarbij sluit een negatieve echografie uitgevoerd in Nederland bijna alle patiënten uit met uitgebreide lymfekliermetastasen (≥4 lymfekliermetastasen). Bovendien omvat gehele borstbestraling na de borstsparende operatie in het algemeen het lagere deel van de oksel en behandelt daarmee eventueel achtergebleven uitzaaiingen. Hoewel de schildwachtklierprocedure minder invasief is vergeleken met een okselklierdissectie, komen complicaties nog steeds voor. Zo lijdt 6% van de patiënten die alleen een schildwachtklierprocedure ondergingen aan ernstige lymfoedeem na slechts 3 jaar. Deze studie gaat onderzoeken of de schildwachtklierprocedure veilig achterwege gelaten kan worden bij borstsparend behandelde patiënten, met een negatieve echografie van de oksel.

Doel van het onderzoek:

Het doel van deze studie is het verminderen van overbehandeling van de oksel bij patiënten met borstkanker en een klinisch negatieve okselklierstatus die behandeld worden met borstsparende therapie, door het achterwege laten van de schildwachtklierprocedure, om zodoende de kans op korte en lange termijn complicaties te verminderen en te zorgen voor een verbetering van de kwaliteit van leven met een even goede ziektecontrole en algehele overleving.

Onderzoeksopzet:

Het betreft een gerandomiseerde non-inferiority multicenter onderzoek. Patienten die voldoen aan de in- en exclusiecriteria worden na toestemming middels loting behandeld volgens één van de volgende behandelmethoden:

- Behandelmethode A (controle groep): schildwachtklierprocedure volgens de Nederlandse borstkankerrichtlijn.
- Behandelmethode B (studie groep): geen verdere okselklierstadiëring.

Stratificatiefactoren: leeftijd (≤50, 50≤75, >75 jaar), oestrogeen receptor status (positief vs. negatief), HER2neu status (geamplificeerd vs. niet geamplificeerd), klinisch tumor stadium (<3 cm vs. ≥3 cm), tumorgraad volgens gemodificeerde Bloom-Richardson (graad I-II vs. graad III), is of zal worden behandeld met primaire systemische therapie (ja/nee), participerend centrum.
Overige behandelingen (o.a. hormonale therapie, chemotherapie) worden op indicatie gegeven volgens de Nederlandse borstkankerrichtlijn.
Tijdens de follow-up periode van 5 jaar wordt de oksel nauwkeurig onderzocht middels lichamelijk onderzoek en echografie op indicatie. Indien sprake is van een cyto-/histologisch bewezen lymfekliermetastase tijdens follow-up, dan kan in het multidisciplinair overleg van de desbetreffende patiënt worden besloten tot het uitvoeren van een vorm van okselklierbehandeling.
Gevalideerde vragenlijsten worden verstrekt voor het onderzoeken van de invloed op het aantal complicaties en kwaliteit van leven (Lymph-ICF, QLQ-C30, QLQ-BR23) en om te meten of angst en persoonlijkheidstrekken invloed hebben op de uitkomst van kwaliteit van leven (STAI-trait, NEO-FFI). Deze vragenlijsten worden verstrekt tot aan 1056 van de 1730 patiënten (528 per arm), éénmaal voor de randomisatie en viermaal na de randomisatie (6 maanden, 1 jaar, 2 jaar, 3 jaar en 5 jaar). Vragenlijsten met betrekking to kosten-effectiviteit worden éénmaal voor de randomisatie en viermaal na de randomisatie (6 maanden, 1,2 en 3 jaar) afgenomen.

Onderzoekspopulatie:

Vrouwen van 18 jaar of ouder met pathologisch bewezen invasief mammacarcinoom die borstsparende therapie ondergaan.
cT1-2: primaire tumor is bij klinisch onderzoek kleiner dan 5 cm.
cN0: bij lichamelijk onderzoek en echografie van de oksel worden geen aanwijzingen voor lymfekliermetastasen gevonden of de cytologie/histologie na punctie/biopt van een lymfeklier is negatief.
Neoadjuvante systemische therapie is toegestaan indien de patiënt voor de start van de neoadjuvante systemische therapie een klinische T1-2N0 status heeft en waarvoor borstsparende therapie op dat moment al geschikt is.
De patiënt heeft informed consent persoonlijk ondertekend.

Interventie (indien van toepassing):

De controlegroep ondergaat wél de schildwachtklierprocedure volgens de Nederlands borstkankerrichtlijn. De experimentele arm ondergaan géén schildwachtklierprocedure.

Primaire onderzoeksvariabelen/uitkomstmaten:

Primaire uitkomstmaat:
- Regionaal recidief percentage

Secundaire onderzoeksvariabelen/uitkomstmaten (indien van toepassing):
Secundaire uitkomstmaten:
- Distant-disease free survival
- Overall survival
- Later uitgevoerde okselbehandeling
- Lokaal recidief percentage
- Overig-regionaal recidief percentage
- Contralaterale borstkanker
- Percentage (neo)adjuvante systemische therapie
- Kwaliteit van leven en okselbehandeling-gerelateerde morbiditeit
- Kosten-effectiviteit

Omschrijving en inschatting van belasting en risico (indien van toepassing):

Wat betreft belasting is er in deze studie géén sprake van extra diagnostiek of interventies (juist minder) en hoeft de patiënt niet vaker dan normaal voor controle naar het ziekenhuis. De eventuele extra belasting voor de patiënt zal alleen bestaan uit het invullen van kwaliteit van leven vragenlijsten (bij 1056 van 1730 patiënten) éénmaal voor de randomisatie en vijfmaal na randomisatie (6 maanden, 1 jaar, 2 jaar, 3 jaar en 5 jaar). Vragenlijsten met betrekking to kosten-effectiviteit worden éénmaal voor de randomisatie en viermaal na de randomisatie (6 maanden, 1,2 en 3 jaar) afgenomen.
De hypothese luidt dat het achterwege laten van de schildwachtklierprocedure even veilig is als het wél uitvoeren van deze procedure. Desondanks is er een risico dat in de experimentele arm meer regionale recidieven optreden en dat bij patiënten op een later tijdstip alsnog een vorm van okselklierbehandeling moet worden uitgevoerd. Daartegenover staat dat er in de experimentele arm door het niet uitvoeren van de schildwachtklierprocedure minder kans is op morbiditeit, minder operaties en opnames in het ziekenhuis en een betere kwaliteit van leven. Patiënten zullen op geen enkele manier vertraging ondervinden als gevolg van deelname aan de studie. Verder wordt de kosteneffectiviteit onderzocht van het achterwege laten van de schildwachtklierprocedure.

K2.Engelse Samenvatting

Background of the study:
The NSABP B-04 trial revealed that omitting primary axillary treatment of occult positive lymph nodes in clinically node negative breast cancer patients does not affect survival, even after 25 years of follow-up and without the use of adjuvant systemic or radiation therapy. A delayed axillary dissection in case lymph nodes become clinically positive does not affect survival and prevents axillary overtreatment in the majority of patients. The ACOSOG Z0011 and IBCSG 23-01 trials revealed that completion axillary dissection can be safely omitted in clinically node negative patients with metastatic sentinel nodes. Patients randomized for watchful waiting were likely to have residual nodal disease (13-27%) that was not surgically removed. Nevertheless, survival rates were not affected and regional recurrence rates low. Use of axillary ultrasound will improve the preoperative selection of node negative patients, as it selects patients with a more favourable tumour load and accurately excludes advanced nodal disease (≥4 metastatic nodes). Biology and adjuvant systemic and radiation therapy are all factors that most likely diminish the risk that possible metastases left in situ develop into clinically detectable lymph nodes. Patients treated with breast conserving therapy are significantly more often diagnosed with pathologic node negative disease or with micrometastatic disease, compared to patients treated with mastectomy. The risk for occult (macro)metastases in cT1-2N0 breast cancer patients treated with breast conserving therapy is low: about 88% will be node negative and about 95% of node positive patients will have no lymph node metastases beyond the sentinel node. Reflected by the low regional recurrence rate after a (false) negative sentinel node, only a small amount of patients (0.8%) with node positive disease is expected to develop clinically detectable nodal disease.

Objective of the study:
The objective of this study is to decrease the number of breast cancer patients receiving overtreatment of the axilla, in order to positively influence the axillary morbidity rate and quality of life. Therefore, we investigate whether the sentinel lymph node procedure can be safely omitted in clinically node negative breast cancer patients undergoing breast conserving therapy, in terms of non-inferior regional control, distant-disease free and overall survival. All objectives are measured during a follow-up of 5 years.

Study design:
A randomized controlled non-inferiority multicenter study. Patients who meet the in- and exclusioncriteria will be randomized after informed consent for one of the two treatment methods:
- Arm A (control arm): lumpectomy with sentinel lymph node procedure, followed by radiotherapy of the breast with or without completion axillary treatment according to the Dutch breast cancer guideline.
- Arm B (experimental arm): lumpectomy without further axillary staging, followed by radiotherapy of the breast.
Stratification: age (≤50, 50≤75, >75 years old), oestrogen receptor status (positive vs. negative), HER2neu status (amplified vs. not amplified), clinical tumour stage prior to any treatment (<3 cm vs. ≥3 cm), grading (grade I-II vs. III - according to Bloom-Richardson grading system), is or will be treated with primary systemic therapy (yes/no) and participating centre.
Adjuvant systemic treatment is administered if indicated according to the Dutch breast cancer guideline.
Primary systemic therapy is allowed if the patient has a clinical T1-2N0 status (initial stage) that is amenable to lumpectomy pre-systemic therapy.
Yearly follow-up with physical examination of the axilla for 5 years. Axillary ultrasound (+/- tissue sampling) on indication. Staging for distant metastases and/or a delayed axillary lymph node dissection if indicated by the multidisciplinary team. Validated questionnaires are used to assess axillary morbidity rate and quality of life (Lymph-ICF, QLQ-C30, QLQ-BR23) and to measure if anxiety and personality traits influence the outcome of quality of life (STAI-trait, NEO-FFI) in 1056 of 1730 patients. These

questionnaires are provided before randomization and sequentially post randomization at 6 months, 1, 2, 3 and 5 years. Cost-effectiveness questionnaires are provided before randomization and sequentially post randomization at 6 months, 1, 2 and 3 years.

Study population:
Women aged 18 years or older with pathologically confirmed invasive breast carcinoma about to undergo breast conserving surgery followed by radiotherapy of the breast.
cT1-2: primary tumour is smaller than 5 cm.
cN0: no signs of axillary lymph node metastases at physical examination and axillary ultrasound (+/- tissue sampling).
Primary systemic treatment is allowed.
Patient is able to and signed the informed consent form.

Intervention (if applicable):
Patients in the control arm will be treated with the sentinel lymph node procedure according to the Dutch breast cancer guideline.
Patients in the experimental arm will not be treated with the sentinel lymph node procedure.

Primary study parameters/outcome of the study:
Primary endpoint:
- Regional recurrence rate

Secundary study parameters/outcome of the study (if applicable):
Secondary endpoints:
- Distant-disease free survival
- Overall survival
- Delayed axillary treatment
- Local recurrence rate
- Other-regional recurrence rate
- Contralateral breast cancer rate
- Percentage difference in the administration of (neo)adjuvant systemic therapy
- Quality of life and axillary morbidity rate
- Cost-effectiveness

Nature and extent of the burden and risks associated with participation, benefit and group relatedness (if applicable):
There is no extra burden for participating patients as regard to additional diagnostic tests/interventions and follow-up. Any additional burden for the patient will only consist of completing quality of life questionnaires in 1056 of 1644 study patients. These questionnaires are provided before randomization and sequentially post randomization at 6 months, 1, 2, 3 and 5 years. The cost-effectiveness analysis study (CEA) was set up later and added to the Qol-study. The inclusion rate of the CEA was 66%. Since this sub-study needed to include 474 patients, still another 74 patients need to be approached until a total of 1056 patients.
If patients do not return the questionnaires or only one out of the first two, a letter to thank the patient for taking part in the study. They will not be bothered with further questionnaires. After patient 1056 no questionnaires will be sent out.
We hypothesize that omitting the sentinel lymph node procedure is not inferior to the standard treatment. Nevertheless, possible disadvantages are a worse regional recurrence rate, and delayed axillary treatment if indicated. Patients in the experimental arm have the possible advantages of less morbidity, fewer operations and hospitalizations and improved quality of life, compared to patients treated with the sentinel lymph node procedure.
No patient will encounter any delay in their treatment as a result of inclusion.

ONDERTEKENING

De verrichter en indiener verklaren hierbij:
a. het formulier (en samenvatting) volledig en naar waarheid te hebben ingevuld;
b. de antwoorden op de vragen uit het ABR-formulier niet in strijd zijn met het bijbehorende onderzoeksdossier en onderzoekscontract

Naar waarheid getekend, door de verrichter (=opdrachtgever)		door de indiener	
datum	....	datum	....
Handtekening		Handtekening	
naam	U.P. Neumann	naam	F. van Duijnhoven
functie	Hoofd afdeling Chirurgie, MUMC+	functie	Chirurg, NKI-AVL

Sluiten Print

Sluiten Print

Formulier voor medisch-ethische beoordeling en registratie

ABR-formulier, versie mei 2021

Onderzoeksdossiernummer

ABR Nummer	**49315**	Status	**Beoordeeld - Positief**
Versie	**14**	Status per	**28-12-2022**
Jaar	**22**		
Dossiernummer	**NL49315.031.14**		
Reden voor	**vervanging PI Deventer**		

A. Sectie - Openbaar maken gegevens medisch wetenschappelijk onderzoek

A1. Het CCMO-register is een voor ieder toegankelijk openbaar trial register. De antwoorden op de vragen gemarkeerd met een wereldbol en de samenvatting bij dit formulier worden openbaar gemaakt in het CCMO register.

B. Sectie - Administratief

B1. Betreft het onderzoek met geneesmiddelen (inclusief gentherapie, somatische celtherapie, vaccinonderzoek, GGO's, zie verdere toelichting) als bedoeld in de Wet medisch-wetenschappelijk onderzoek met mensen (WMO)?

○ ja
◉ nee

B2. Houdt het onderzoek verband met een eerder door een erkende METC of door de CCMO beoordeelde studie of is het onderzoek reeds eerder bij een erkende METC ter beoordeling voorgelegd?

○ ja, het onderzoek houdt verband met – of is het vervolg op – een eerder beoordeelde studie
○ ja, het onderzoek is eerder ter beoordeling aan een erkende METC of de CCMO voorgelegd (stuur kopie besluit mee)
◉ nee

B4. Is het protocol (nog) in een ander openbaar trial register geregistreerd?

○ ja
◉ nee

B5. Naam indiener/contactpersoon voor de oordelende toetsingscommissie

**B5a.**	Achternaam indiener/contactpersoon	Duijnhoven
	Titel en voorletters	dr. F.
	Tussenvoegsels	van
**B5b.**	Type organisatie/bedrijf	Overige Ziekenhuizen
	Organisatie/bedrijf	Antoni van Leeuwenhoek Ziekenhuis
	Naam organisatie/bedrijf	IKNL trialbureau
	Afdeling	Chirurgie
	Adres	Plesmanlaan 121
	Postcode en plaats	1066 CX Amsterdam
	Land	NL
**B5c.**	Intern adres	afdeling Heelkunde
	Telefoon	020 512 9111
	Fax	020 512 2554
	E-mail	f.v.duijnhoven@nki.nl

B6. Is de indiener werkzaam bij de opdrachtgever/sponsor (verrichter) van het onderzoek?

○ Ja ◉ Nee

B7. Opdrachtgever/sponsor van het onderzoek (verrichter volgens de WMO)

Type Organisatie/Bedrijf	Universitair Medisch Centrum
Organisatie/Bedrijf	Medisch Universitair Ziekenhuis Maastricht

Adres	P. Debyelaan 25
Postcode en plaats	6229 HX Maastricht
Land	NL
Telefoon	0433877477
Fax	0433875473
E-mail	m.smidt@mumc.nl

C. Sectie - Onderzoek

C1. Volledige titel van het onderzoek
C1a. In het Engels
Clinically node negative breast cancer patients undergoing breast conserving therapy: Sentinel lymph node procedure versus follow-up. A Dutch randomized controlled multicentre trial.

C1b. In het Nederlands
Patiënten met borstkanker en een klinisch negatieve okselklierstatus die een borstsparende behandeling ondergaan: Schildwachtklierprocedure versus follow-up. Een Nederlandse gerandomiseerde multicenter studie.

C2. Verkorte titel van het onderzoek/acroniem
C2a. In het Engels
BOOG 2013-08

C2b. In het Nederlands (Let op: deze korte titel wordt vermeld binnen ToetsingOnline)
BOOG 2013-08

C3. Trefwoorden (maximaal 4, plaats elk trefwoord op een aparte regel)
C3a. In het Engels
Breast neoplasms
Breast conserving therapy
Sentinel lymph node biopsy
Axilla

C3b. In het Nederlands
Borstkanker
Borstsparende behandeling
Schildwachtklierprocedure
Oksel

C4. Beschrijf het belang van het onderzoek en de beoogde toepassing van de resultaten (verwijs eventueel naar de relevante pagina's in het protocol).
Het doel van dit onderzoek is om:
1) Overbehandeling van de oksel te verminderen;
2) Het aantal korte en lange termijn complicaties te verminderen;
3) De kwaliteit van leven te verbeteren;
4) Met een even goede ziektecontrole en algehele overleving

Dit willen wij bereiken door te onderzoeken of de schildwachtklierprocedure veilig achterwege gelaten kan worden bij patiënten met borstkanker en een klinisch negatieve okselklierstatus behandeld met borstsparende therapie.

C6. Betreft het onderzoek een multicenter-onderzoek?
○ nee
◉ ja - alleen in Nederland
○ ja - internationaal binnen de Europese Unie
○ ja - internationaal ook buiten de Europese Unie

C7. Is er bij multicenter-onderzoek sprake van een coördinerend onderzoeker?
◉ ja namelijk
○ nee

Naam onderzoeker:
M.L. Smidt

C8. Wie is/zijn medisch verantwoordelijk voor de proefpersonen die deelnemen aan het onderzoek
Behandelend arts en lokale hoofdonderzoeker

C9. In welk centrum/welke centra (incl. huisartsenpraktijken) in Nederland wordt het onderzoek uitgevoerd?

Centrum	Proefpersonen	Hoofdonderzoeker	Onafhankelijk arts
Amphia Ziekenhuis	69	E.J.T. Luiten	S.O. Breukink (Maastricht UMC+)
Zuyderland Medisch Centrum	39	E.R.M. van Haaren	S.O. Breukink (Maastricht UMC+)

Canisius Wilhelmina Ziekenhuis	46	L.J.A. Strobbe	S.O. Breukink (Maastricht UMC+)
Catharina-ziekenhuis	46	G.A.P. Nieuwenhuijzen	S.O. Breukink (Maastricht UMC+)
Deventer Ziekenhuis	39	H. Torrenga	S.O. Breukink (Maastricht UMC+)
Diakonessenhuis Utrecht	39	J.Volders	S.O. Breukink (Maastricht UMC+)
Gelre Ziekenhuizen	37	J.H.G. Klinkenberg	S.O. Breukink (Maastricht UMC+)
HagaZiekenhuis	41	I. Jannink	S.O. Breukink (Maastricht UMC+)
Isala Klinieken	54	A.B. Francken	S.O. Breukink (Maastricht UMC+)
Laurentius Ziekenhuis	23	A.V.R.J. Bell	S.O. Breukink (Maastricht UMC+)
Academisch Ziekenhuis Maastricht	39	M.L. Smidt	S.O. Breukink (Maastricht UMC+)
Maxima Medisch Centrum	31	K. Schenk	S.O. Breukink (Maastricht UMC+)
Antoni van Leeuwenhoek Ziekenhuis	93	F. van Duijnhoven	S.O. Breukink (Maastricht UMC+)
Universitair Medisch Centrum Sint Radboud	31	J.H.W. de Wilt	S.O. Breukink (Maastricht UMC+)
Sint Antonius Ziekenhuis	58	R. Koelemij	S.O. Breukink (Maastricht UMC+)
Tergooiziekenhuizen locatie Hilversum	42	E.J.C. Vriens	S.O. Breukink (Maastricht UMC+)
Universitair Medisch Centrum Groningen	17	J. de Vries	S.O. Breukink (Maastricht UMC+)
Universitair Medisch Centrum Utrecht	23	A.J. Witkamp	S.O. Breukink (Maastricht UMC+)
Viecuri Medisch Centrum voor Noord-Limburg	39	P.H.A Nijhuis	S.O. Breukink (Maastricht UMC+)
Ziekenhuisvoorzieningen Gelderse Vallei	32	W.K. de Roos	S.O. Breukink (Maastricht UMC+)
Rivierenland Ziekenhuis	23	M.F. Sier	S.O. Breukink (Maastricht UMC+)
Noordwest Ziekenhuisgroep	54	K.M. Blaauwendraat	S.O. Breukink (Maastricht UMC+)
Rijnstate Ziekenhuis	62	R. van Eekeren	S.O. Breukink (Maastricht UMC+)
Jeroen Bosch Ziekenhuis	20	M. Bessems	B. Biesma (Jeroen Bosch ziekenhuis)
Alrijne ziekenhuis	50	P. Neijenhuis	S.O. Breukink (Maastricht UMC+)
Spaarne Gasthuis	30	B. Kortmann	S.O. Breukink (Maastricht UMC+)
St. Jansdal	40	Schouten van der Velden	S.O. Breukink (Maastricht UMC+)
Treant Zorggroep	50	L. Smit	S.O. Breukink (Maastricht UMC+

C10. Betreft het onderzoek met:

- [x] mensen
- [ ] geslachtscellen
- [ ] (rest-)embryo's
- [ ] foetussen in utero

C10a. Indien mensen aangevinkt (meerdere antwoorden mogelijk):

- [ ] preterme pasgeborenen (< 37 weken zwangerschap)
- [ ] pasgeborenen (0-27 dagen)
- [ ] babies en peuters (28 dagen – 23 maanden)
- [ ] kinderen (2-11 jaar)
- [ ] jongeren (12-15 jaar)
- [ ] adolescenten (16-17 jaar)
- [x] volwassenen (18-64 jaar)
- [x] ouderen (65 jaar en ouder)
- [ ] zwangere vrouwen
- [ ] vrouwen die borstvoeding geven

C11. Beoogd totaal aantal proefpersonen/(rest)embryo's/foetussen in utero:
C11a. In Nederland
1730

C13. Onderzoeksgebied

- ☐ etiologie
- ☐ organisatorisch/zorgonderzoek
- ☐ diagnostiek
- ☐ preventie
- ☑ therapie
- ☐ veiligheid
- ☐ werkzaamheid
- ☐ farmacokinetiek
- ☐ farmacodynamiek
- ☐ bio-equivalentie
- ☐ dosis-respons
- ☐ farmacogenomics
- ☐ farmaco-economie
- ☐ anders

C14. Type onderzoek

- ○ observationeel onderzoek zonder invasieve metingen
- ○ observationeel onderzoek met invasieve metingen
- ◉ interventie-onderzoek

C15. In welke fase kan het onderzoek worden ingedeeld?

- ○ fase I (a)
- ○ fase II (b)
- ◉ fase III (c)
- ○ fase IV (d)
- ○ overige onderzoeken waarbij geneesmiddelen worden toegepast (e)
- ○ niet van toepassing

C17. Is er sprake van een ander(e) onderzoeksproduct en/of interventie dan vermeld bij vraag C16 (zie toelichting, kan zowel voor observationeel als interventie-onderzoek worden ingevuld als het product (verder) onderzocht wordt.)

- ◉ Ja
- ○ Nee

C17a. ☐ Medisch hulpmiddel (medisch hulpmiddel, actief implantaat of in-vitro diagnosticum), namelijk
C17b. ☐ Operatie, namelijk
C17c. ☐ Psychosociale interventie, namelijk
C17d. ☐ Voeding(stoffen), namelijk
C17e. ☐ Bewegingstherapie, namelijk
C17f. ☐ interventie met radioactieve straling, namelijk
C17g. ☐ Blootstellingsonderzoek (bijv pesticidenonderzoek), namelijk
C17h.
C17i. ☑ andere interventie, namelijk

In het Engels: No sentinel lymph node procedure
In het Nederlands: Géén schildwachtklierprocedure

C18. Worden de onderzoeksproducten voor deze studie door de verrichter gratis verstrekt?

- ○ ja
- ○ nee
- ○ gedeeltelijk namelijk
- ◉ niet van toepassing

C19. Is/zijn er (een) controlegroep (en)?
