# Supplementary material for: Three-year patient-reported outcomes of the BOOG 2013-08 RCT evaluating omission of sentinel lymph node biopsy in early-stage breast cancer patients treated with breast conserving surgery: Impact of personality traits on health-related quality of life
Source: Br J Surg. 2025 May 14;112(5):znaf031. doi: 10.1093/bjs/znaf031 (PMC12076147; doi:10.1093/bjs/znaf031)
Supplement: znaf031_Supplementary_Data [file znaf031_supplementary_data.zip › Supplementary_material.docx]

Three-year patient-reported outcomes of the BOOG 2013-08 RCT evaluating omission of sentinel lymph node biopsy in early-stage breast cancer patients treated with breast conserving surgery: Impact of personality traits on health-related quality of life

Veerle M. Wintraecken, MSc*^1,2^, Lori M. van Roozendaal, MD, PhD^3^, Janine M. Simons, MD, PhD^1,4^, Jolanda de Vries PhD^5,6^, Sander M.J. van Kuijk, PhD^7^, Marissa L.G. Vane, MD, PhD^1,2^, Thijs van Dalen, MD, PhD^8,9^, Helena Sackey^10,11^, Jos A. van der Hage, MD PhD^12^, Luc J.A. Strobbe, MD PhD^13^, Sabine C. Linn, MD, PhD^14^, Marc B.I. Lobbes, MD, PhD^15^, Philip M.P Poortmans, MD, Phd^16,17^, Vivianne C.G. Tjan-Heijnen, MD PhD^1,18^, Koen K.B.T. van de Vijver, MD, PhD^19,20,21^, Helen H. Westenberg, MD^22^, Carmen D. Dirksen, PhD^7,23^, Johan H.W. de Wilt, MD, PhD^24^, Liesbeth J. Boersma, MD, PhD^25^, Marjolein L. Smidt, MD, PhD^1,2^, on behalf of the BOOG 2013-08 group.

^1^ GROW – Research Institute for Oncology and Reproduction, Maastricht University, Maastricht, The Netherlands.
^2^ Department of Surgery, Maastricht University Medical Centre+, Maastricht, The Netherlands.
^3^ Department of Surgical Oncology, Zuyderland Medical Center, Sittard-Geleen, the Netherlands.
^4^ Department of Radiotherapy, Erasmus Medical Centre, Rotterdam, The Netherlands.
^5^ Department of Medical and Clinical Psychology, Tilburg University, Tilburg, the Netherlands.
^6^ Board member Adrz, Goes, The Netherlands.
^7^ Department of Clinical Epidemiology and Medical Technology Assessment, Maastricht University Medical Centre+, Maastricht, the Netherlands.
^8^ Division of Surgical Oncology, Diakonessenhuis Hospital, Utrecht, the Netherlands.
^9^ Department of Surgery, Erasmus Medical Centre, Rotterdam, The Netherlands.
^10^Institutet, Department of Molecular Medicine and Surgery, Karolinska Institutet, Stockholm, Sweden.
^11^Department of Breast- Endocrine Tumours and Sarcoma, Karolinska University Hospital, Stockholm, Sweden.
^12^ Division of Surgical Oncology, Leids University Medical Center, Leiden, the Netherlands.
^13^ Division of Surgical Oncology, Canisius-Wilhelmina Hospital, Nijmegen, the Netherlands.
^14^ Division of Medical Oncology, Netherlands Cancer Institute - Antoni van Leeuwenhoek Hospital, Amsterdam, the Netherlands.
^15^ Department of Medical Imaging, Zuyderland Medical Center, Sittard-Geleen, the Netherlands.
^16^ Department of Radiation Oncology, Iridium Network, Antwerp, Belgium.
^17^ Faculty of Medicine and Health Sciences, University of Antwerp, Belgium.
^18^ Division of Medical Oncology, Maastricht University Medical Centre, Maastricht, the Netherlands.
^19^ Department of Pathology, Ghent University Hospital, Ghent, Belgium.
^20^ Department of Diagnostic Sciences, Cancer Research Institute Ghent (CRIG), Ghent University, Ghent, Belgium.
^21^ Center for Gynaecological Oncology Amsterdam (CGOA), Department of Gynaecology, Netherlands Cancer Institute - Antoni van Leeuwenhoek Hospital, Amsterdam, the Netherlands.
^22^ Radiation Oncology, Radiotherapiegroep location Arnhem, Arnhem, the Netherlands.
^23^ Care and Public Health Research Institute (CAPHRI), University Maastricht, the Netherlands.
^24^ Division of Surgical Oncology, Radboud University Medical Centre, Nijmegen, the Netherlands.
^25^ Department of Radiation Oncology (Maastro), GROW Research Institute for Oncology and Reproduction, Maastricht University Medical Centre+, Maastricht, the Netherlands.

*Corresponding author: Veerle Marieke Wintraecken
Maastricht University
veerle.wintraecken@mumc.nl
ORCID ID: 0000-0002-2642-8385

**Supplementary Materials - Index**

| **Supplementary Figures and Tables** |  |
| --- | --- |
| **Table S1** Arm function and HRQoL scores at 6 months, 1-, 2-, and 3 years after study inclusion in a subselection of participants from the BOOG2013-08 trial | Page 3 |
| **Table S2** Baseline clinicopathological characteristics of BOOG 2013-08 participants categorized per personality trait | Page 6 |
| **Table S3** Arm function and HRQoL scores for participants from the BOOG2013-08 trial based on personality trait scores with low TA and N levels up to 3 years after study inclusion | Page 8 |
| **Table S4** Linear mixed model analysis on total arm function scores according to the as-treated principle | Page 15 |
| **Table S5 L**inear mixed model analysis on total arm function scores according to intention-to-treat principle | Page 17 |
| **Table S6** Linear mixed model analysis on global HRQoL scores according to the as-treated principle | Page 19 |
| **Table S7** Linear mixed model analysis results on global HRQoL scores: intention-to-treat principle | Page 21 |

**Supplementary Figures and Tables**

**Table S1** Arm function and HRQoL scores at 6 months, 1-, 2-, and 3 years after study inclusion in a subselection of participants from the BOOG2013-08 trial

|  | **Pre-surgery (SD)** | ***P* value** | **6 months (SD)** | ***P* value** | **1 year (SD)** | ***P* value** | **2 years (SD)** | ***P* value** | **3 years (SD)** | ***P* value** |
| --- | --- | --- | --- | --- | --- | --- | --- | --- | --- | --- |
| **Arm function domain scores (SD)*** |  |  |  |  |  |  |  |  |  |  |
| **Total score** |  | .119 |  | .090 |  | **.048** |  | .241 |  | .300 |
| SLNB; N = 383 | 5.6 (8.9) |  | 11.3 (13.7) |  | 10.1 (13.1) |  | 9.4 (12.5) |  | 15.9 (13.7) |  |
| No-SLNB; N = 438 | 5.9 (7.7) |  | 9.7 (12.0) |  | 8.7 (12.2) |  | 8.4 (11.5) |  | 14.4 (12.4) |  |
| **Physical function** |  | .212 |  | **<.001** |  | **<.001** |  | **.025** |  | .334 |
| SLNB | 2.6 (7.3) |  | 8.9 (13.7) |  | 8.5 (13.5) |  | 7.4 (13.4) |  | 15.2 (16.8) |  |
| No-SLNB | 2.9 (7.7) |  | 5.2 (8.5) |  | 5.8 (12.1) |  | 5.4 (10.8) |  | 13.6 (15.1) |  |
| **Mental** |  | .907 |  | **.042** |  | **.032** |  | **.040** |  | .261 |
| SLNB | 6.6 (13.2) |  | 6.3 (13.6) |  | 6.6 (14.6) |  | 5.7 (13.6) |  | 10.7 (15.3) |  |
| No-SLNB | 6.1 (11.6) |  | 5.1 (12.8) |  | 5.4 (13.3) |  | 4.5 (12.4) |  | 9.3 (14.1) |  |
| **Household domain** |  | .222 |  | .326 |  | .299 |  | .606 |  | .291 |
| SLNB | 5.7 (13.0) |  | 12.1 (18.4) |  | 10.3 (16.6) |  | 9.9 (16.2) |  | 16.1 (19.0) |  |
| No-SLNB | 6.4 (12.3) |  | 11.0 (16.4) |  | 9.6 (16.3) |  | 10.3 (17.5) |  | 14.7 (17.8) |  |
| **Mobility domain** |  | .184 |  | .316 |  | .082 |  | .628 |  | .446 |
| SLNB | 7.3 (13.2) |  | 16.2 (19.9) |  | 15.0 (18.9) |  | 14.4 (18.5) |  | 20.3 (19.1) |  |
| No-SLNB | 7.7 (12.4) |  | 14.7 (17.9) |  | 12.8 (17.0) |  | 13.5 (17.3) |  | 19.3 (17.9) |  |
| **Life and social activities** |  | .050 |  | .542 |  | .885 |  | .484 |  | .166 |
| SLNB | 7.4 (14.3) |  | 14.0 (20.1) |  | 10.9 (17.2) |  | 10.5 (15.8) |  | 16.8 (18.2) |  |
| No-SLNB | 7.9 (13.1) |  | 13.3 (18.6) |  | 11.3 (17.6) |  | 10.1 (16.5) |  | 14.8 (16.9) |  |
| **EORTC QLQ C30** |  |  |  |  |  |  |  |  |  |  |
| **Global Health**** |  | .107 |  | .460 |  | .529 |  | .562 |  | .579 |
| SLNB | 80.9 (17.4) |  | 75.9 (19.2) |  | 80.4 (18.3) |  | 80.9 (16.8) |  | 73.8 (20.5) |  |
| No-SLNB | 79.6 (16.4) |  | 75.1 (18.8) |  | 80.1 (17.0) |  | 80.1 (17.0) |  | 73.7 (20.2) |  |
| ***Functioning scales*** |  |  |  |  |  |  |  |  |  |  |
| **Physical** |  | **.022** |  | .128 |  | .628 |  | .472 |  | .349 |
| SLNB | 92.8 (12.6) |  | 88.2 (14.9) |  | 89.2 (15.0) |  | 89.2 (14.3) |  | 84.7 (15.7) |  |
| No-SLNB | 91.6 (12.0) |  | 87.3 (14.3) |  | 89.5 (13.1) |  | 88.9 (13.6) |  | 85.7 (15.2) |  |
| **Role** |  | .191 |  | .596 |  | .486 |  | .710 |  | .397 |
| SLNB | 91.6 (17.8) |  | 81.7 (25.0) |  | 85.8 (23.8) |  | 88.3 (20.1) |  | 81.4 (24.6) |  |
| No-SLNB | 90.3 (19.1) |  | 81.3 (24.1) |  | 87.1 (21.9) |  | 87.5 (20.6) |  | 83.0 (23.1) |  |
| **Emotional** |  | .448 |  | .910 |  | .827 |  | .798 |  | .467 |
| SLNB | 78.2 (19.0) |  | 83.2 (20.6) |  | 85.2 (19.0) |  | 86.3 (17.9) |  | 82.4 (20.4) |  |
| No-SLNB | 78.1 (17.7) |  | 83.7 (19.2) |  | 85.2 (19.3) |  | 86.2 (18.4) |  | 83.3 (19.9) |  |
| **Cognitive** |  | .475 |  | .527 |  | .610 |  | .461 |  | .605 |
| SLNB | 89.7 (16.5) |  | 84.7 (22.3) |  | 86.4 (20.0) |  | 87.1 (19.2) |  | 85.9 (19.5) |  |
| No-SLNB | 89.7 (14.8) |  | 84.5 (20.4) |  | 86.0 (19.7) |  | 86.2 (19.5) |  | 86.3 (19.0) |  |
| **Social** |  | .308 |  | .831 |  | .746 |  | .278 |  | .420 |
| SLNB | 93.3 (16.0) |  | 86.8 (20.3) |  | 91.1 (18.2) |  | 93.1 (15.9) |  | 90.1 (16.6) |  |
| No-SLNB | 93.7 (12.9) |  | 87.5 (18.6) |  | 91.0 (17.3) |  | 91.5 (17.9) |  | 90.3 (17.0) |  |
| ***Symptom scale/ items****** |  |  |  |  |  |  |  |  |  |  |
| **Fatigue** |  | **.003** |  | .598 |  | .886 |  | .654 |  | .083 |
| SLNB | 13.2 (17.5) |  | 27.0 (24.0) |  | 21.9 (22.5) |  | 19.1 (21.3) |  | 27.1 (25.2) |  |
| No-SLNB | 17.0 (19.4) |  | 27.9 (24.2) |  | 21.2 (21.2) |  | 19.3 (20.5) |  | 30.3 (25.2) |  |
| **Nausea/ vomiting** |  | .736 |  | .519 |  | .706 |  | .706 |  | .461 |
| SLNB | 2.6 (9.9) |  | 4.9 (12.8) |  | 3.4 (9.9) |  | 2.5 (8.6) |  | 5.1 (10.1) |  |
| No-SLNB | 2.7 (9.3) |  | 4.7 (13.2) |  | 3.2 (9.7) |  | 2.5 (8.5) |  | 5.6 (11.4) |  |
| **Pain** |  | .204 |  | .341 |  | .435 |  | .710 |  | .450 |
| SLNB | 8.0 (17.4) |  | 16.6 (22.3) |  | 14.6 (21.6) |  | 12.1 (19.1) |  | 23.8 (25.7) |  |
| No-SLNB | 9.2 (17.6) |  | 15.1 (20.8) |  | 13.9 (21.5) |  | 12.1 (18.7) |  | 24.5 (26.7) |  |
| **Dyspnea** |  | **.014** |  | .387 |  | .856 |  | .745 |  | .442 |
| SLNB | 6.3 (16.4) |  | 15.4 (23.9) |  | 11.2 (21.0) |  | 11.3 (20.3) |  | 17.9 (23.9) |  |
| No-SLNB | 8.8 (18.3) |  | 13.8 (22.8) |  | 10.6 (19.3) |  | 11.3 (19.9) |  | 19.4 (24.3) |  |
| **Insomnia** |  | .896 |  | .723 |  | .724 |  | .462 |  | .466 |
| SLNB | 23.3 (26.1) |  | 24.2 (27.9) |  | 22.2 (27.7) |  | 20.1 (25.2) |  | 25.0 (29.3) |  |
| No-SLNB | 23.8 (27.1) |  | 23.9 (28.8) |  | 22.2 (26.4) |  | 21.6 (26.0) |  | 25.8 (28.3) |  |
| **Appetite loss** |  | .163 |  | .182 |  | .773 |  | .632 |  | .192 |
| SLNB | 5.5 (15.0) |  | 7.4 (17.8) |  | 5.0 (15.8) |  | 3.8 (13.5) |  | 6.6 (14.8) |  |
| No-SLNB | 6.9 (16.1) |  | 9.8 (22.0) |  | 5.2 (15.8) |  | 4.6 (16.1) |  | 8.2 (16.3) |  |
| **Constipation** |  | .916 |  | .380 |  | .494 |  | .616 |  | .490 |
| SLNB | 3.9 (13.8) |  | 7.4 (18.9) |  | 6.5 (16.8) |  | 8.7 (20.2) |  | 10.1 (20.3) |  |
| No-SLNB | 4.0 (14.0) |  | 8.1 (18.6) |  | 7.1 (17.0) |  | 8.8 (19.3) |  | 10.7 (20.8) |  |
| **Diarrhea** |  | .354 |  | .899 |  | .324 |  | .376 |  | .321 |
| SLNB | 4.3 (13.5) |  | 6.0 (18.1) |  | 3.7 (12.8) |  | 5.0 (14.8) |  | 9.9 (20.0) |  |
| No-SLNB | 5.6 (16.7) |  | 6.2 (18.1) |  | 4.8 (14.9) |  | 4.1 (13.5) |  | 8.2 (18.4) |  |
| **Financial difficulties** |  | .383 |  | **.032** |  | .294 |  | .739 |  | .359 |
| SLNB | 2.0 (10.7) |  | 5.9 (16.5) |  | 5.2 (14.3) |  | 3.7 (12.5) |  | 6.2 (14.6) |  |
| No-SLNB | 2.3 (10.4) |  | 3.7 (13.2) |  | 5.0 (16.3) |  | 3.9 (14.6) |  | 5.4 (13.7) |  |
| **EORTC QLQ BR-23** |  |  |  |  |  |  |  |  |  |  |
| *Functioning scales*** |  |  |  |  |  |  |  |  |  |  |
| **Body image** |  | .755 |  | .805 |  | .605 |  | .743 |  | .584 |
| SLNB | 92.4 (14.8) |  | 89.2 (18.7) |  | 90.0 (17.3) |  | 92.1 (15.3) |  | 89.4 (15.8) |  |
| No-SLNB | 93.2 (13.4) |  | 89.2 (18.8) |  | 90.2 (18.2) |  | 91.4 (17.6) |  | 89.3 (17.0) |  |
| **Sexual functioning** |  | .854 |  | .837 |  | .199 |  | .495 |  | .297 |
| SLNB | 78.1 (23.0) |  | 77.1 (20.6) |  | 77.3 (21.7) |  | 75.5 (21.5) |  | 78.5 (23.2) |  |
| No-SLNB | 78.2 (23.4) |  | 76.6 (22.0) |  | 75.0 (23.2) |  | 76.7 (21.4) |  | 78.8 (21.6) |  |
| **Sexual enjoyment** |  | .377 |  | .129 |  | .216 |  | .083 |  | .517 |
| SLNB | 50.6 (34.1) |  | 44.5 (30.9) |  | 46.5 (31.1) |  | 41.2 (26.6) |  | 43.6 (26.8) |  |
| No-SLNB | 52.0 (33.7) |  | 48.6 (30.2) |  | 50.3 (29.8) |  | 45.2 (25.6) |  | 43.6 (26.7) |  |
| **Future perspective** |  | .269 |  | .278 |  | .683 |  | .332 |  | .192 |
| SLNB | 67.6 (24.3) |  | 72.1 (25.3) |  | 73.1 (23.1) |  | 76.2 (23.2) |  | 71.0 (28.6) |  |
| No-SLNB | 65.2 (25.1) |  | 70.8 (23.9) |  | 72.5 (23.4) |  | 74.9 (22.3) |  | 68.6 (28.6) |  |
| *Symptom scale/ items **** |  |  |  |  |  |  |  |  |  |  |
| **Systemic therapy side effects** |  | .088 |  | .746 |  | .615 |  | .278 |  | .274 |
| SLNB | 8.5 (11.0) |  | 15.8 (14.7) |  | 13.0 (12.7) |  | 11.8 (11.1) |  | 16.0 (15.1) |  |
| No-SLNB | 9.8 (11.5) |  | 15.9 (15.6) |  | 13.0 (13.5) |  | 13.1 (12.3) |  | 17.7 (16.1) |  |
| **Breast symptoms** |  | .541 |  | .446 |  | **.048** |  | .594 |  | .339 |
| SLNB | 7.5 (12.9) |  | 22.0 (20.0) |  | 18.3 (18.3) |  | 13.6 (15.6) |  | 17.4 (18.5) |  |
| No-SLNB | 7.4 (11.2) |  | 20.2 (17.1) |  | 15.6 (16.4) |  | 12.4 (13.6) |  | 17.7 (17.7) |  |
| **Arm symptoms** |  | .424 |  | **.049** |  | **<.001** |  | .480 |  | .557 |
| SLNB | 4.3 (11.2) |  | 12.6 (17.0) |  | 11.1 (15.7) |  | 9.4 (15.4) |  | 14.7 (18.9) |  |
| No-SLNB | 4.5 (10.9) |  | 10.5 (16.2) |  | 7.5 (14.6) |  | 8.4 (14.6) |  | 14.3 (17.8) |  |
| **Upset by hair loss** |  | .530 |  | .416 |  | .526 |  | .356 |  | .457 |
| SLNB | 24.1 (24.2) |  | 26.8 (25.3) |  | 23.6 (23.9) |  | 19.3 (20.7) |  | 21.7 (22.4) |  |
| No-SLNB | 24.2 (23.8) |  | 27.6 (26.8) |  | 24.2 (23.8) |  | 21.0 (21.7) |  | 22.5 (22.5) |  |

* Higher scores indicates worse arm function ** Higher score indicates better functioning *** Higher score indicates more symptoms

**Table S2** Baseline clinicopathological characteristics of BOOG 2013-08 participants categorized per personality trait

| **Characteristic** | **Low TA and N**  **N = 619** | **High TA**  **N = 167** | **High N**  **N = 35** | ***P* value** |
| --- | --- | --- | --- | --- |
| **Demographical characteristics** |  |  |  |  |
| **Age, years** |  |  |  | **.004** |
| Mean; SD (range) | 61.1;9.5 (37–87) | 63.7;7.8 (40-79) | 60.6;10.1 (38-83) |  |
| **BMI** |  |  |  | .218 |
| Mean; SD (range) | 27.0;4.9 (18-53) | 27.7;5.0 (19-44) | 26.9;4.1 (18-35) |  |
| **Current smoker** N (%) |  |  |  | .649 |
| Yes | 84 (15.6%) | 23 (16.2%) | 8 (25.8%) |  |
| Missing | 80 | 26 | 4 |  |
| **Relationship status** N (%) |  |  |  | .194 |
| Married/ committed | 218 (71.58%) | 58 (79.5%) | 6 (50%) |  |
| Divorced, separated, widowed or single | 75 (24.6%) | 13 (17.8%) | 6 (50%) |  |
| Other |  |  |  |  |
| Missing | 12 (3.9%) | 1 (1.4%) | - |  |
| **Children** N (%) |  |  |  | .172 |
| Yes | 262 (85.9%) | 57 (78.1%) | 9 (75.0%) |  |
| Missing | 314 | 94 | 23 |  |
| **Ethnicity** N (%) |  |  |  | .900 |
| Dutch | 298 (97.7%) | 69 (94.5%) | 12 (100%) |  |
| European | 4 (1.3%) | 2 (2.7%) | - |  |
| Asian | 1 (0.3%) | 1 (1.4%) | - |  |
| Antillean | 1 (0.3%) | - | - |  |
| Surinamese | 1 (0.3%) | 1 (1.4%) | - |  |
| Missing | 314 | 94 | 23 |  |
| **Educational level** N (%) |  |  |  | .385 |
| Low | 131 (43.0%) | 35 (47.9%) | 7 (58.3%) |  |
| Moderate | 78 (25.7%) | 19 (26.0%) | 3 (25.0%) |  |
| High | 95 (31.3%) | 18 (24.7%) | 2 (16.7%) |  |
| Missing | 315 | 94 | 23 |  |
| **Paid work** N (%) |  |  |  | .076 |
| Yes | 144 (47.8%) | 20 (27.4%) | 6 (50%) |  |
| Missing | 318 | 94 | 23 |  |
| **Number of comorbidities** N (%) |  |  |  | .083 |
| 0 | 28 (10.0%) | 2 (3.2%) | - |  |
| 1 | 92 (32.7%) | 15 (24.2%) | 1 (12.5%) |  |
| ≥ 2 | 161 (57.3%) | 45 (72.6%) | 7 (87.5%) |  |
| Missing | 338 | 105 | 27 |  |
| **Clinical factors** |  |  |  |  |
| **cT stage** N (%) |  |  |  | .610 |
| cT1 | 500 (80.8%) | 140 (83.8%) | 28 (80.0%) |  |
| cT2 | 119 (19.2%) | 27 (16.2%) | 7 (20.0%) |  |
| **Histological subtype** N (%) |  |  |  | .773 |
| Invasive ductal carcinoma / invasive carcinoma no special type | 478 (77.7%) | 127 (76.5%) | 30 (85.7%) |  |
| Invasive lobular carcinoma | 72 (11.7%) | 22 (13.3%) | 3 (8.6%) |  |
| Other | 65 (10.6%) | 16 (9.6%) | 2 (5.7%) |  |
| Missing | 4 | 1 | - |  |
| **Pathological grade (Bloom Richardson)** N (%) |  |  |  | .052 |
| Grade I | 183 (38.0%) | 57 (42.2%) | 7 (20.0%) |  |
| Grade II | 245 (50.8%) | 61 (45.2%) | 19 (54.3%) |  |
| Grade III | 54 (11.2%) | 17 (12.6%) | 9 (25.7%) |  |
| Missing | 137 | 32 | - |  |
| **Hormone receptor status** N (%) |  |  |  | .907 |
| ER+ and HER2+ | 46 (7.5%) | 9 (5.4%) | 3 (8.6%) |  |
| ER- and HER2+ | 13 (2.1%) | 3 (1.8%) | 1 (2.9%) |  |
| ER+ and HER2 - | 513 (83.6%) | 142 (85.0%) | 30 (85.7%) |  |
| Triple negative | 42 (6.8%) | 12 (7.2%) | 1 (2.9%) |  |
| Missing | 5 | - |  |  |
| **Neo-adjuvant therapy** N (%) |  |  |  | .094 |
| No neo-adjuvant therapy | 547 (88.3%) | 156 (93.4%) | 30 (85.7%) |  |
| Chemotherapy | 32 (5.2%) | 7 (4.2%) | 1 (2.9%) |  |
| Immuno- or targeted therapy | - | - | - |  |
| Hormonal therapy | 14 (2.3%) | 2 (1.2%) | - |  |
| Chemo- and immuno- or targeted therapy | 26 (4.2%) | 2 (1.2%) | 4 (11.4%) |  |
| **Pathological N stage** N (%) |  |  |  | .801 |
| pN0 | 277 (44.7%) | 74 (44.3%) | 13 (37.1%) |  |
| pN0(+i) | 13 (2.1%) | 4 (2.4%) |  |  |
| pN1mi | 15 (2.4%) | 1 (0.6%) | 2 (5.7%) |  |
| pN1 | 19 (3.1%) | 7 (4.2%) | 1 (2.9%) |  |
| pN2 | - | - | - |  |
| pNX | 295 (47.7%) | 81 (48.5%) | 19 (54.3%) |  |
| **Additional axillary treatment** N (%) |  |  |  | .811 |
| ALND only | 1 (0.2%) | - | - |  |
| Regional RT only | 28 (4.5%) | 6 (3.7%) | - |  |
| ALND and regional RT | 2 (0.3%) | 2 (1.2%) | 2 (5.7%) |  |
| **Adjuvant therapy** N (%) |  |  |  | .300 |
| No adjuvant therapy | 330 (53.3%) | 94 (56.3%) | 14 (40.0%) |  |
| Chemotherapy | 17 (2.7%) | 4 (2.4%) | - |  |
| Immuno- or targeted therapy | 6 (1.0%) | - | - |  |
| Hormonal therapy | 185 (29.9%) | 52 (31.1%) | 14 (40.0%) |  |
| Chemo- and immuno- or targeted therapy | 7 (1.1%) | 3 (1.8%) | 1 (2.9%) |  |
| Chemo- and hormonal therapy | 45 (7.3%) | 8 (4.8%) | 3 (8.6%) |  |
| Immuno-and hormonal therapy | 19 (3.1%) | 1 (0.6%) | 3 (8.6%) |  |
| Chemo, immuno- or targeted, and hormonal therapy | 10 (1.6%) | 4 (2.4%) | - |  |

Note: missing values were not included for calculation of the percentages.
* The breast specimen of one participant in the no-SLNB arm contained a pathological macrometastasis.
Abbreviations: SLNB sentinel lymph node biopsy; SD standard deviation; BMI body mass index; mm millimeter; ER estrogen receptor; HER2 Human Epidermal growth factor Receptor 2 ALND axillary lymph node dissection; RT radiation therapy
BMI was categorized into 4 groups: Underweight (≤18.5); Normal weight (BMI 18.5 – 24.9); Overweight (25.0 – 29.9); and Obese (≥ 30.0).
Educational level was categorized into low, moderate and high educational level. Low educational level includes primary school, lower vocational, low or intermediate general education. Moderate educational level includes intermediate vocational education and higher general education. High educational level refers to higher vocational education and university

## **Table S3** Arm function and HRQoL scores for participants from the BOOG2013-08 trial based on personality trait scores with low TA and N levels up to 3 years after study inclusion

|  | **Personality group** | **Baseline** |  | **6 months** |  | **1 year** |  | **2 years** |  | **3 years** |  |
| --- | --- | --- | --- | --- | --- | --- | --- | --- | --- | --- | --- |
|  |  | **Mean score** | ***P* value** | **Mean score** | ***P* value** | **Mean score** | ***P* value** | **Mean score** | ***P* value** | **Mean score** | ***P* value** |
| **LYMPH-ICF** |  |  |  |  |  |  |  |  |  |  |  |
| **Total** | **Low TA and N** |  |  |  |  |  |  |  |  |  |  |
| SLNB; N = 294 |  | 4.5 | .196 | 10.0 | .152 | 9.2 | .070 | 8.6 | .398 | 15.0 | .398 |
| No-SLNB; N = 325 |  | 5.0 |  | 8.5 |  | 7.5 |  | 7.1 |  | 13.4 |  |
|  | **High TA** |  |  |  |  |  |  |  |  |  |  |
| SLNB; N = 72 |  | 7.8 | .736 | 13.2 | .120 | 10.4 | .378 | 10.6 | .211 | 17.9 | 2.55 |
| No-SLNB; N = 95 |  | 7.8 |  | 10.1 |  | 9.9 |  | 10.5 |  | 16.0 |  |
|  | **High N** |  |  |  |  |  |  |  |  |  |  |
| SLNB; N = 17 |  | 15.3 | .556 | 26.7 | .794 | 25.0 | .861 | 19.1 | .795 | 21.8 | .690 |
| No-SLNB; N = 18 |  | 13.4 |  | 27.7 |  | 24.5 |  | 20.0 |  | 24.9 |  |
| ***Domain scores**** |  |  |  |  |  |  |  |  |  |  |  |
| **Physical function** | **Low TA and N** |  |  |  |  |  |  |  |  |  |  |
| SLNB |  | 2.2 | .375 | 8.1 | **<.001** | 7.8 | **<.001** | 7.0 | .054 | 14.4 | .393 |
| No-SLNB |  | 2.3 |  | 4.5 |  | 4.9 |  | 4.5 |  | 12.7 |  |
|  | **High TA** |  |  |  |  |  |  |  |  |  |  |
| SLNB |  | 2.8 | .811 | 10.0 | .080 | 8.3 | **.013** | 7.2 | .156 | 16.7 | .400 |
| No-SLNB |  | 4.7 |  | 6.0 |  | 6.3 |  | 7.3 |  | 14.9 |  |
|  | **High N** |  |  |  |  |  |  |  |  |  |  |
| SLNB |  | 9.6 | .477 | 18.9 | .616 | 21.3 | .773 | 16.3 | .545 | 21.8 | .734 |
| No-SLNB |  | 3.7 |  | 13.5 |  | 19.4 |  | 11.1 |  | 21.7 |  |
| **Mental function** | **Low TA and N** |  |  |  |  |  |  |  |  |  |  |
| SLNB |  | 4.1 | .856 | 3.9 | .200 | 4.9 | .071 | 4.5 | .237 | 9.7 | .391 |
| No-SLNB |  | 4.4 |  | 3.7 |  | 4.1 |  | 3.2 |  | 8.2 |  |
|  | **High TA** |  |  |  |  |  |  |  |  |  |  |
| SLNB |  | 11.5 | .647 | 12.1 | **.028** | 8.5 | .291 | 6.7 | **.021** | 11.9 | .265 |
| No-SLNB |  | 9.6 |  | 5.5 |  | 7.3 |  | 5.4 |  | 11.1 |  |
|  | **High N** |  |  |  |  |  |  |  |  |  |  |
| SLNB |  | 28.6 | .484 | 26.4 | .655 | 28.5 | .272 | 21.3 | .795 | 22.1 | .681 |
| No-SLNB |  | 19.6 |  | 27.6 |  | 17.8 |  | 22.9 |  | 19.9 |  |
| **Household domain** | **Low TA and N** |  |  |  |  |  |  |  |  |  |  |
| SLNB |  | 4.7 | .610 | 11.0 | .231 | 9.7 | .267 | 9.3 | .477 | 15.7 | .374 |
| No-SLNB |  | 5.5 |  | 9.4 |  | 8.3 |  | 8.5 |  | 13.7 |  |
|  | **High TA** |  |  |  |  |  |  |  |  |  |  |
| SLNB |  | 8.7 | .809 | 12.5 | .637 | 11.0 | .423 | 11.1 | .768 | 17.5 | .302 |
| No-SLNB |  | 7.6 |  | 11.7 |  | 10.6 |  | 14.4 |  | 16.0 |  |
|  | **High N** |  |  |  |  |  |  |  |  |  |  |
| SLNB |  | 11.0 | .404 | 30.1 | .534 | 17.8 | .182 | 14.5 | .334 | 18.0 | .302 |
| No-SLNB |  | 15.5 |  | 33.6 |  | 27.4 |  | 20.7 |  | 26.0 |  |
| **Mobility domain** | **Low TA and N** |  |  |  |  |  |  |  |  |  |  |
| SLNB |  | 6.3 | .384 | 14.3 | .532 | 13.8 | .135 | 13.6 | .547 | 19.5 | .456 |
| No-SLNB |  | 6.6 |  | 13.3 |  | 11.0 |  | 11.9 |  | 18.1 |  |
|  | **High TA** |  |  |  |  |  |  |  |  |  |  |
| SLNB |  | 9.4 | .759 | 20.6 | .118 | 15.8 | .314 | 15.2 | .703 | 22.2 | .633 |
| No-SLNB |  | 10.0 |  | 15.1 |  | 15.2 |  | 15.1 |  | 20.9 |  |
|  | **High N** |  |  |  |  |  |  |  |  |  |  |
| SLNB |  | 15.7 | .105 | 35.1 | .718 | 31.3 | .706 | 25.9 | .291 | 25.2 | .333 |
| No-SLNB |  | 16.9 |  | 37.4 |  | 31.7 |  | 33.7 |  | 32.8 |  |
| **Life and social activities** | **Low TA and N** |  |  |  |  |  |  |  |  |  |  |
| SLNB |  | 6.2 | .076 | 13.0 | .533 | 10.4 | .603 | 9.4 | .492 | 15.8 | .292 |
| No-SLNB |  | 7.2 |  | 12.3 |  | 10.1 |  | 8.9 |  | 13.7 |  |
|  | **High TA** |  |  |  |  |  |  |  |  |  |  |
| SLNB |  | 10.1 | .785 | 15.1 | .248 | 9.6 | .481 | 13.5 | .603 | 20.4 | .239 |
| No-SLNB |  | 8.7 |  | 12.4 |  | 12.2 |  | 12.6 |  | 16.8 |  |
|  | **High N** |  |  |  |  |  |  |  |  |  |  |
| SLNB |  | 15.8 | .190 | 26.9 | .272 | 24.1 | .514 | 17.7 | .713 | 20.1 | .590 |
| No-SLNB |  | 17.8 |  | 34.9 |  | 27.9 |  | 19.2 |  | 25.1 |  |
| **EORTC QLQ-C30** |  |  |  |  |  |  |  |  |  |  |  |
| **Global Health**** | **Low TA and N** |  |  |  |  |  |  |  |  |  |  |
| SLNB |  | 83.4 | .065 | 77.2 | .499 | 81.6 | .737 | 82.1 | .489 | 74.4 | .496 |
| No-SLNB |  | 81.8 |  | 76.3 |  | 81.8 |  | 80.9 |  | 74.1 |  |
|  | **High TA** |  |  |  |  |  |  |  |  |  |  |
| SLNB |  | 74.1 | .795 | 73.8 | .765 | 77.9 | .579 | 78.2 | .756 | 72.1 | .446 |
| No-SLNB |  | 75.1 |  | 73.5 |  | 77.3 |  | 78.8 |  | 74.1 |  |
|  | **High N** |  |  |  |  |  |  |  |  |  |  |
| SLNB |  | 65.2 | .810 | 61.6 | .785 | 69.7 | .474 | 71.6 | .748 | 69.7 | .547 |
| No-SLNB |  | 63.9 |  | 62.6 |  | 64.9 |  | 71.3 |  | 64.2 |  |
| ***Functioning scales***** |  |  |  |  |  |  |  |  |  |  |  |
| **Physical** | **Low TA and N** |  |  |  |  |  |  |  |  |  |  |
| SLNB |  | 93.6 | .057 | 88.9 | .191 | 90.1 | .509 | 90.2 | .592 | 85.7 | .598 |
| No-SLNB |  | 92.7 |  | 87.8 |  | 90.3 |  | 90.2 |  | 86.0 |  |
|  | **High TA** |  |  |  |  |  |  |  |  |  |  |
| SLNB |  | 90.0 | .771 | 87.2 | .839 | 87.7 | .691 | 86.4 | .794 | 81.2 | .057 |
| No-SLNB |  | 89.5 |  | 87.4 |  | 88.1 |  | 86.5 |  | 86.2 |  |
|  | **High N** |  |  |  |  |  |  |  |  |  |  |
| SLNB |  | 89.8 | .065 | 80.3 | .288 | 79.3 | .835 | 82.8 | .389 | 82.6 | .518 |
| No-SLNB |  | 82.6 |  | 76.7 |  | 82.6 |  | 79.2 |  | 77.7 |  |
| **Role** | **Low TA and N** |  |  |  |  |  |  |  |  |  |  |
| SLNB |  | 92.2 | .652 | 82.4 | .641 | 87.0 | .611 | 89.5 | .557 | 82.5 | .492 |
| No-SLNB |  | 91.6 |  | 82.1 |  | 88.3 |  | 88.6 |  | 83.9 |  |
|  | **High TA** |  |  |  |  |  |  |  |  |  |  |
| SLNB |  | 90.4 | .370 | 81.1 | .842 | 84.6 | .386 | 85.8 | .608 | 78.0 | .324 |
| No-SLNB |  | 88.6 |  | 81.6 |  | 86.8 |  | 85.4 |  | 82.3 |  |
|  | **High N** |  |  |  |  |  |  |  |  |  |  |
| SLNB |  | 86.2 | .168 | 70.6 | .427 | 68.5 | .682 | 77.2 | .800 | 77.5 | .577 |
| No-SLNB |  | 75.3 |  | 65.3 |  | 67.8 |  | 77.9 |  | 71.6 |  |
| **Emotional** | **Low TA and N** |  |  |  |  |  |  |  |  |  |  |
| SLNB |  | 81.5 | 2.72 | 86.4 | .695 | 88.2 | .796 | 88.6 | .752 | 84.6 | .631 |
| No-SLNB |  | 81.2 |  | 86.1 |  | 87.9 |  | 88.5 |  | 84.9 |  |
|  | **High TA** |  |  |  |  |  |  |  |  |  |  |
| SLNB |  | 70.6 | .667 | 77.9 | .794 | 78.6 | .717 | 81.0 | .668 | 77.1 | .341 |
| No-SLNB |  | 71.8 |  | 80.3 |  | 80.3 |  | 83.5 |  | 81.0 |  |
|  | **High N** |  |  |  |  |  |  |  |  |  |  |
| SLNB |  | 54.3 | .812 | 50.9 | .470 | 61.7 | .832 | 69.2 | .633 | 68.6 | .606 |
| No-SLNB |  | 56.9 |  | 58.9 |  | 62.3 |  | 60.1 |  | 66.9 |  |
| **Cognitive** | **Low TA and N** |  |  |  |  |  |  |  |  |  |  |
| SLNB |  | 90.4 | .665 | 86.1 | .632 | 87.9 | .511 | 88.0 | .400 | 86.1 | .627 |
| No-SLNB |  | 91.2 |  | 86.0 |  | 87.0 |  | 87.1 |  | 86.7 |  |
|  | **High TA** |  |  |  |  |  |  |  |  |  |  |
| SLNB |  | 90.1 | .125 | 84.0 | .749 | 85.8 | .716 | 86.2 | .604 | 86.8 | .565 |
| No-SLNB |  | 86.6 |  | 84.9 |  | 85.8 |  | 86.6 |  | 87.1 |  |
|  | **High N** |  |  |  |  |  |  |  |  |  |  |
| SLNB |  | 75.3 | .573 | 64.4 | .462 | 62.4 | .589 | 75.2 | .483 | 79.2 | .535 |
| No-SLNB |  | 78.7 |  | 55.3 |  | 69.3 |  | 67.9 |  | 73.9 |  |
| **Social** | **Low TA and N** |  |  |  |  |  |  |  |  |  |  |
| SLNB |  | 94.6 | .280 | 87.7 | .854 | 92.3 | .416 | 94.0 | .352 | 90.8 | .569 |
| No-SLNB |  | 94.9 |  | 88.0 |  | 92.0 |  | 92.5 |  | 91.0 |  |
|  | **High TA** |  |  |  |  |  |  |  |  |  |  |
| SLNB |  | 90.3 | .883 | 85.4 | .300 | 88.8 | .561 | 91.6 | .732 | 87.6 | .417 |
| No-SLNB |  | 91.5 |  | 88.1 |  | 89.4 |  | 91.0 |  | 89.7 |  |
|  | **High N** |  |  |  |  |  |  |  |  |  |  |
| SLNB |  | 85.0 | .886 | 77.2 | .693 | 78.4 | .630 | 83.6 | .434 | 87.7 | .506 |
| No-SLNB |  | 85.2 |  | 75.4 |  | 82.1 |  | 77.4 |  | 82.2 |  |
| ***Symptom scale/ items****** |  |  |  |  |  |  |  |  |  |  |  |
| **Fatigue** | **Low TA and N** |  |  |  |  |  |  |  |  |  |  |
| SLNB |  | 11.9 | **.002** | 26.3 | .790 | 20.8 | .709 | 17.2 | .496 | 25.4 | .081 |
| No-SLNB |  | 15.9 |  | 26.9 |  | 20.0 |  | 18.1 |  | 29.3 |  |
|  | **High TA** |  |  |  |  |  |  |  |  |  |  |
| SLNB |  | 15.2 | .785 | 26.8 | .722 | 22.1 | .745 | 23.3 | .440 | 30.9 | .755 |
| No-SLNB |  | 17.4 |  | 28.1 |  | 22.6 |  | 20.1 |  | 30.7 |  |
|  | **High N** |  |  |  |  |  |  |  |  |  |  |
| SLNB |  | 25.7 | .195 | 40.9 | .532 | 39.6 | .744 | 34.4 | .627 | 40.8 | .479 |
| No-SLNB |  | 35.8 |  | 44.9 |  | 36.2 |  | 37.0 |  | 47.1 |  |
| **Nausea/ vomiting** | **Low TA and N** |  |  |  |  |  |  |  |  |  |  |
| SLNB |  | 2.5 | .883 | 4.3 | .849 | 2.8 | .607 | 2.1 | .550 | 4.6 | .552 |
| No-SLNB |  | 2.1 |  | 4.5 |  | 2.6 |  | 2.3 |  | 5.1 |  |
|  | **High TA** |  |  |  |  |  |  |  |  |  |  |
| SLNB |  | 2.6 | .703 | 5.9 | .562 | 3.6 | .417 | 2.4 | .704 | 6.1 | .595 |
| No-SLNB |  | 3.4 |  | 5.1 |  | 5.1 |  | 3.0 |  | 7.2 |  |
|  | **High N** |  |  |  |  |  |  |  |  |  |  |
| SLNB |  | 4.0 | .603 | 11.9 | .359 | 12.1 | .191 | 10.4 | .473 | 9.0 | .625 |
| No-SLNB |  | 10.2 |  | 5.6 |  | 5.2 |  | 3.7 |  | 7.5 |  |
| **Pain** | **Low TA and N** |  |  |  |  |  |  |  |  |  |  |
| SLNB |  | 7.3 | .609 | 15.5 | .226 | 13.6 | .239 | 11.0 | .713 | 21.8 | .445 |
| No-SLNB |  | 7.4 |  | 13.6 |  | 11.7 |  | 10.9 |  | 22.8 |  |
|  | **High TA** |  |  |  |  |  |  |  |  |  |  |
| SLNB |  | 10.1 | .404 | 19.0 | .810 | 15.4 | .797 | 14.9 | .269 | 29.9 | .397 |
| No-SLNB |  | 12.5 |  | 18.1 |  | 17.0 |  | 13.2 |  | 26.9 |  |
|  | **High N** |  |  |  |  |  |  |  |  |  |  |
| SLNB |  | 12.1 | .135 | 25.9 | .638 | 28.8 | .515 | 17.7 | .113 | 31.9 | .436 |
| No-SLNB |  | 24.1 |  | 26.2 |  | 36.0 |  | 28.3 |  | 42.5 |  |
| **Dyspnea** | **Low TA and N** |  |  |  |  |  |  |  |  |  |  |
| SLNB |  | 5.1 | **.040** | 13.7 | .462 | 9.4 | .795 | 9.9 | .757 | 16.7 | .409 |
| No-SLNB |  | 7.1 |  | 12.5 |  | 8.6 |  | 10.1 |  | 18.4 |  |
|  | **High TA** |  |  |  |  |  |  |  |  |  |  |
| SLNB |  | 8.8 | .262 | 18.7 | .854 | 14.6 | .695 | 15.5 | .766 | 21.3 | .570 |
| No-SLNB |  | 12.1 |  | 17.4 |  | 16.0 |  | 14.1 |  | 21.3 |  |
|  | **High N** |  |  |  |  |  |  |  |  |  |  |
| SLNB |  | 16.0 | .678 | 30.8 | .343 | 27.9 | .509 | 18.9 | .803 | 23.5 | .557 |
| No-SLNB |  | 20.4 |  | 20.0 |  | 17.6 |  | 17.8 |  | 29.3 |  |
| **Insomnia** | **Low TA and N** |  |  |  |  |  |  |  |  |  |  |
| SLNB |  | 21.4 | .898 | 23.3 | .140 | 20.1 | .795 | 18.6 | .548 | 24.0 | .427 |
| No-SLNB |  | 20.9 |  | 20.5 |  | 19.9 |  | 19.9 |  | 23.7 |  |
|  | **High TA** |  |  |  |  |  |  |  |  |  |  |
| SLNB |  | 26.9 | .533 | 25.1 | .259 | 26.7 | .783 | 24.1 | .642 | 27.2 | .456 |
| No-SLNB |  | 31.2 |  | 30.1 |  | 25.9 |  | 25.1 |  | 29.3 |  |
|  | **High N** |  |  |  |  |  |  |  |  |  |  |
| SLNB |  | 39.4 | .899 | 35.9 | .155 | 39.8 | .724 | 30.6 | .799 | 32.0 | .372 |
| No-SLNB |  | 38.9 |  | 53.3 |  | 42.8 |  | 33.5 |  | 44.0 |  |
| **Appetite loss** | **Low TA and N** |  |  |  |  |  |  |  |  |  |  |
| SLNB |  | 5.6 | .756 | 6.7 | .220 | 4.4 | .713 | 2.9 | .277 | 5.9 | .174 |
| No-SLNB |  | 5.4 |  | 9.2 |  | 4.6 |  | 4.6 |  | 7.6 |  |
|  | **High TA** |  |  |  |  |  |  |  |  |  |  |
| SLNB |  | 5.1 | .095 | 8.1 | .612 | 5.9 | .694 | 5.4 | .242 | 7.9 | .498 |
| No-SLNB |  | 9.9 |  | 9.3 |  | 6.0 |  | 3.6 |  | 9.5 |  |
|  | **High N** |  |  |  |  |  |  |  |  |  |  |
| SLNB |  | 5.9 | .348 | 16.0 | .633 | 11.3 | .609 | 12.5 | .809 | 12.9 | .746 |
| No-SLNB |  | 16.7 |  | 23.5 |  | 10.6 |  | 10.7 |  | 11.5 |  |
| **Constipation** | **Low TA and N** |  |  |  |  |  |  |  |  |  |  |
| SLNB |  | 3.3 | .832 | 7.3 | .579 | 6.2 | .553 | 7.8 | .467 | 10.0 | .442 |
| No-SLNB |  | 3.6 |  | 7.9 |  | 6.6 |  | 8.1 |  | 10.2 |  |
|  | **High TA** |  |  |  |  |  |  |  |  |  |  |
| SLNB |  | 4.4 | .804 | 5.7 | .530 | 6.8 | .759 | 9.6 | .655 | 9.2 | .525 |
| No-SLNB |  | 3.5 |  | 7.7 |  | 7.4 |  | 9.0 |  | 10.9 |  |
|  | **High N** |  |  |  |  |  |  |  |  |  |  |
| SLNB |  | 13.8 | .724 | 16.0 | .713 | 11.3 | .461 | 21.2 | .799 | 15.2 | .591 |
| No-SLNB |  | 14.8 |  | 15.0 |  | 15.7 |  | 21.3 |  | 20.4 |  |
| **Diarrhea** | **Low TA and N** |  |  |  |  |  |  |  |  |  |  |
| SLNB |  | 4.2 | .665 | 5.7 | .834 | 2.4 | .210 | 3.9 | .540 | 9.1 | .442 |
| No-SLNB |  | 4.4 |  | 5.7 |  | 3.5 |  | 3.0 |  | 7.8 |  |
|  | **High TA** |  |  |  |  |  |  |  |  |  |  |
| SLNB |  | 5.3 | .805 | 7.2 | .705 | 7.5 | .871 | 9.1 | .156 | 12.5 | .329 |
| No-SLNB |  | 7.2 |  | 6.7 |  | 7.9 |  | 5.7 |  | 8.5 |  |
|  | **High N** |  |  |  |  |  |  |  |  |  |  |
| SLNB |  | 2.1 | .267 | 7.4 | .770 | 10.5 | .888 | 8.0 | .605 | 12.2 | .708 |
| No-SLNB |  | 18.5 |  | 13.3 |  | 110.3 |  | 14.8 |  | 13.2 |  |
| **Financial difficulties** | **Low TA and N** |  |  |  |  |  |  |  |  |  |  |
| SLNB |  | 1.8 | .490 | 4.7 | .139 | 3.9 | .868 | 3.2 | .754 | 5.5 | .524 |
| No-SLNB |  | 2.1 |  | 3.5 |  | 4.8 |  | 3.5 |  | 5.4 |  |
|  | **High TA** |  |  |  |  |  |  |  |  |  |  |
| SLNB |  | 0.9 | .381 | 9.1 | **.038** | 7.4 | .158 | 4.2 | .590 | 7.1 | .306 |
| No-SLNB |  | 2.2 |  | 3.1 |  | 4.7 |  | 3.8 |  | 5.2 |  |
|  | **High N** |  |  |  |  |  |  |  |  |  |  |
| SLNB |  | 9.8 | .889 | 12.7 | .820 | 17.2 | .394 | 10.1 | .790 | 14.4 | .472 |
| No-SLNB |  | 5.6 |  | 11.5 |  | 9.3 |  | 12.6 |  | 6.9 |  |
| **EORTC QLQ BR-23** |  |  |  |  |  |  |  |  |  |  |  |
| ***Functioning scales***** |  |  |  |  |  |  |  |  |  |  |  |
| **Body image** | **Low TA and N** |  |  |  |  |  |  |  |  |  |  |
| SLNB |  | 93.5 | .358 | 91.1 | .717 | 91.5 | .904 | 93.6 | .658 | 90.8 | .561 |
| No-SLNB |  | 94.4 |  | 90.0 |  | 91.0 |  | 92.2 |  | 90.1 |  |
|  | **High TA** |  |  |  |  |  |  |  |  |  |  |
| SLNB |  | 90.3 | .304 | 85.4 | .783 | 86.8 | .175 | 86.6 | .187 | 84.7 | .099 |
| No-SLNB |  | 90.8 |  | 89.6 |  | 91.2 |  | 92.7 |  | 89.0 |  |
|  | **High N** |  |  |  |  |  |  |  |  |  |  |
| SLNB |  | 82.1 | .599 | 72.1 | .883 | 76.4 | .708 | 89.3 | .350 | 85.3 | .355 |
| No-SLNB |  | 83.2 |  | 70.6 |  | 69.8 |  | 69.7 |  | 75.8 |  |
| **Sexual functioning** | **Low TA and N** |  |  |  |  |  |  |  |  |  |  |
| SLNB |  | 76.5 | .817 | 75.7 | .813 | 75.4 | .426 | 74.5 | .431 | 77.7 | .424 |
| No-SLNB |  | 76.6 |  | 75.6 |  | 73.7 |  | 76.0 |  | 78.3 |  |
|  | **High TA** |  |  |  |  |  |  |  |  |  |  |
| SLNB |  | 83.6 | .711 | 82.1 | .461 | 84.1 | .096 | 78.1 | .585 | 80.6 | .428 |
| No-SLNB |  | 83.0 |  | 79.6 |  | 78.0 |  | 78.3 |  | 79.5 |  |
|  | **High N** |  |  |  |  |  |  |  |  |  |  |
| SLNB |  | 82.5 | .847 | 80.6 | .735 | 80.5 | .793 | 81.6 | .621 | 82.8 | .670 |
| No-SLNB |  | 81.5 |  | 79.7 |  | 80.6 |  | 80.9 |  | 83.0 |  |
| **Sexual enjoyment** | **Low TA and N** |  |  |  |  |  |  |  |  |  |  |
| SLNB |  | 48.9 | .429 | 43.6 | .208 | 44.9 | .194 | 40.6 | .089 | 43.0 | .561 |
| No-SLNB |  | 50.6 |  | 47.4 |  | 48.7 |  | 44.7 |  | 43.3 |  |
|  | **High TA** |  |  |  |  |  |  |  |  |  |  |
| SLNB |  | 55.7 | .520 | 49.3 | .482 | 51.8 | .499 | 44.0 | .369 | 45.0 | .419 |
| No-SLNB |  | 55.2 |  | 51.5 |  | 54.3 |  | 47.5 |  | 44.3 |  |
|  | **High N** |  |  |  |  |  |  |  |  |  |  |
| SLNB |  | 57.1 | .499 | 39.5 | .243 | 52.4 | .564 | 39.6 | .644 | 48.5 | .409 |
| No-SLNB |  | 59.8 |  | 55.9 |  | 57.2 |  | 43.1 |  | 44.4 |  |
| **Future perspective** | **Low TA and N** |  |  |  |  |  |  |  |  |  |  |
| SLNB |  | 70.2 | .260 | 74.4 | .308 | 75.8 | .607 | 78.5 | .328 | 73.6 | .169 |
| No-SLNB |  | 67.8 |  | 73.3 |  | 74.9 |  | 76.6 |  | 69.7 |  |
|  | **High TA** |  |  |  |  |  |  |  |  |  |  |
| SLNB |  | 62.4 | .532 | 67.6 | .681 | 64.2 | .491 | 72.0 | .795 | 64.2 | .490 |
| No-SLNB |  | 59.2 |  | 66.5 |  | 67.1 |  | 73.6 |  | 68.1 |  |
|  | **High N** |  |  |  |  |  |  |  |  |  |  |
| SLNB |  | 43.3 | .598 | 51.7 | .745 | 64.1 | .499 | 54.8 | .633 | 56.0 | .665 |
| No-SLNB |  | 50.0 |  | 48.0 |  | 57.2 |  | 50.6 |  | 51.3 |  |
| ***Symptom scale/ items ****** |  |  |  |  |  |  |  |  |  |  |  |
| **Systemic therapy side effects** | **Low TA and N** |  |  |  |  |  |  |  |  |  |  |
| SLNB |  | 7.6 | .062 | 15.1 | .458 | 11.8 | .652 | 10.7 | .254 | 15.6 | .331 |
| No-SLNB |  | 8.7 |  | 14.9 |  | 11.7 |  | 12.0 |  | 17.1 |  |
|  | **High TA** |  |  |  |  |  |  |  |  |  |  |
| SLNB |  | 11.5 | .826 | 16.9 | .553 | 16.2 | .663 | 14.8 | .750 | 16.9 | .597 |
| No-SLNB |  | 12.6 |  | 18.2 |  | 15.3 |  | 14.3 |  | 18.9 |  |
|  | **High N** |  |  |  |  |  |  |  |  |  |  |
| SLNB |  | 12.7 | .44 | 22.5 | .777 | 20.5 | .598 | 18.2 | .252 | 20.2 | .530 |
| No-SLNB |  | 16.2 |  | 21.1 |  | 25.3 |  | 26.4 |  | 22.0 |  |
| **Breast symptoms** | **Low TA and N** |  |  |  |  |  |  |  |  |  |  |
| SLNB |  | 6.5 | .303 | 21.3 | .352 | 18.0 | **.007** | 13.0 | .465 | 17.0 | .460 |
| No-SLNB |  | 7.0 |  | 19.4 |  | 14.3 |  | 11.6 |  | 17.3 |  |
|  | **High TA** |  |  |  |  |  |  |  |  |  |  |
| SLNB |  | 8.1 | .914 | 24.2 | .671 | 18.5 | .591 | 15.2 | .894 | 18.5 | .385 |
| No-SLNB |  | 7.8 |  | 20.9 |  | 17.2 |  | 13.5 |  | 17.5 |  |
|  | **High N** |  |  |  |  |  |  |  |  |  |  |
| SLNB |  | 21.9 | .256 | 24.7 | .258 | 23.3 | .395 | 16.2 | .537 | 20.0 | .535 |
| No-SLNB |  | 13.0 |  | 30.6 |  | 29.7 |  | 21.6 |  | 25.7 |  |
| **Arm symptoms** | **Low TA and N** |  |  |  |  |  |  |  |  |  |  |
| SLNB |  | 3.7 | .426 | 12.4 | **.037** | 10.8 | **<.001** | 8.3 | .769 | 14.1 | .583 |
| No-SLNB |  | 3.9 |  | 10.1 |  | 6.7 |  | 7.7 |  | 13.3 |  |
|  | **High TA** |  |  |  |  |  |  |  |  |  |  |
| SLNB |  | 4.9 | .722 | 12.4 | .511 | 9.6 | .140 | 11.4 | .226 | 16.3 | .748 |
| No-SLNB |  | 6.3 |  | 10.5 |  | 8.4 |  | 9.7 |  | 16.3 |  |
|  | **High N** |  |  |  |  |  |  |  |  |  |  |
| SLNB |  | 11.7 | .793 | 16.4 | .586 | 23.6 | .415 | 19.6 | .568 | 18.9 | .634 |
| No-SLNB |  | 7.4 |  | 17.0 |  | 17.0 |  | 14.4 |  | 20.6 |  |
| **Upset by hair loss** | **Low TA and N** |  |  |  |  |  |  |  |  |  |  |
| SLNB |  | 24.2 | .520 | 26.5 | .496 | 23.3 | .468 | 19.3 | .408 | 21.6 | .405 |
| No-SLNB |  | 24.2 |  | 27.4 |  | 22.9 |  | 20.3 |  | 21.9 |  |
|  | **High TA** |  |  |  |  |  |  |  |  |  |  |
| SLNB |  | 24.1 | .559 | 28.7 | .487 | 23.8 | .465 | 18.3 | .309 | 22.1 | .624 |
| No-SLNB |  | 23.7 |  | 28.5 |  | 26.4 |  | 22.5 |  | 23.4 |  |
|  | **High N** |  |  |  |  |  |  |  |  |  |  |
| SLNB |  | 22.0 | .554 | 24.8 | .707 | 28.3 | .462 | 23.2 | .515 | 21.2 | .288 |
| No-SLNB |  | 26.4 |  | 25.1 |  | 37.0 |  | 25.6 |  | 29.5 |  |

* Higher scores indicate worse arm function ** Higher score indicate better functioning *** Higher score indicate more symptoms

## **Table S4** Results from linear mixed model analysis on total arm function scores according to the as-treated principle

|  | **LMM without personality** |  | **LMM with personality** |  |
| --- | --- | --- | --- | --- |
| **Parameter** | **Regression coefficient (95% CI)** | ***P* value** | **Regression coefficient (95% CI)** | ***P* value** |
| **Time, (in time points)** | 1.3 (0.87 – 1.8) | **<.001** | 1.3 (0.86 – 1.7) | **<.001** |
| **Treatment arm** |  |  |  |  |
| - No-SLNB | Reference group |  | Reference group |  |
| - SLNB | 2.7 (0.53 – 4.9) | **.015** | 2.7 (0.51 – 4.8) | **.015** |
| **Age (in years)** | -0.03 (-0.20 - 0.15) | .784 | .02 (-0.15 – 0.19) | .833 |
| **Axillary treatment** |  |  |  |  |
| - No additional axillary treatment | Reference group |  | Reference group |  |
| - ALND or Regional RT | -2.6 (-8.1 – 3.0) | .365 | -2.4 (-7.8 – 3.1) | .394 |
| - ALND and regional RT | -9.4 (-27.5 – 8.7) | .309 | -9.2 (-26.9 – 8.4) | .304 |
| **(Neo)adjuvant treatment** |  |  |  |  |
| - No (neo-) adjuvant therapy | Reference group |  | Reference group |  |
| - Chemotherapy | 7.8 (-0.43 – 16.0) | .063 | 8.2 (0.14 – 16.2) | **.046** |
| - Hormonal therapy | -0.51 (-3.2 – 2.2) | .705 | -1.3 (-3.9 – 1.4) | .340 |
| - Chemo- and immunotherapy | -12.0 (-29.4 – 5.5) | .178 | -10.6 (-27.7 – 6.4) | .220 |
| - Chemo- and hormonal therapy | -1.3 (-5.5 – 2.9) | .535 | -1.1 (-5.2 – 3.0) | .610 |
| - Chemo-, immune- and hormonal therapy | 1.1 (-7.1 – 9.3) | .786 | 0.80 (-7.2 – 8.8) | .845 |
| **cT stage** |  |  |  |  |
| - cT1 | Reference group |  | Reference group |  |
| - cT2 | 0.19 (-4.3 – 3.9) | .927 | 0.61 (-3.4 – 4.6) | .765 |
| **pT stage** |  |  |  |  |
| - pT0 | Reference group |  | Reference group |  |
| - pT1 | 10.1 (-2.5 – 22.7) | .117 | 9.2 (-3.1 – 21.4) | .144 |
| - pT2 | 10.9 (-2.2 – 23.9) | .102 | 10.2 (-2.5 – 22.9) | .115 |
| - pTis | 1.9 (-14.1 – 17.8) | .819 | 1.9 (-13.6 – 17.4) | .812 |
| **Personality** |  |  |  |  |
| - Low personality level |  |  | Reference group |  |
| - High TA level |  |  | 2.2 (-0.66 – 5.0) | .133 |
| - High N level |  |  | 13.1 (6.3 – 19.8) | **<.001** |
| **Comorbidities (the amount of)** | 1.5 (0.74 – 2.2) | **<.001** | 1.2 (0.47 – 1.9) | **.001** |
| **Educational level** |  |  |  |  |
| - Low educational level | Reference group |  | Reference group |  |
| - Moderate educational level | 1.4 (-1.3 – 4.1) | .315 | 2.0 (-0.69– 4.7) | .147 |
| - High educational level | -0.59 (-3.2 – 2.1) | .659 | -0.13 (-2.7 – 2.5) | .924 |
| **BMI** | 0.38 (0.17 - 0.59) | **<.001** | 0.39 (0.19 – 0.60) | **<.001** |
| **Smoking (no)** | -0.70 (-1.9 - 0.53) | .263 | -0.62 (-1.8 – 0.58) | .309 |
| **Marital status** |  |  |  |  |
| - Single | Reference group |  | Reference group |  |
| - Married | -0.53 (-4.8 – 3.7) | .808 | 0.33 (-3.9 – 4.5) | .879 |
| - Divorced/ widow | 0.52 (-4.4 – 5.5) | .837 | 1.5 (-3.3 – 6.4) | .538 |
| - Living together | 0.43 (-5.7 – 4.9) | .873 | 0.22 (-4.9 – 5.4) | .933 |
| - Other | 0.54 (-6.0 – 7.0) | .871 | 1.9 (-4.5 – 8.2) | .566 |
| **Children (the number of)** | 1.1 (-2.2 – 4.4) | .505 | 1.3 (-2.0 – 4.5) | .449 |
| **Employment status** |  |  |  |  |
| - No, no paid job | Reference group |  | Reference group |  |
| - No, retired | -4.0 (-7.4 - -0.55) | **.023** | -3.9 (-7.3 - -0.55) | **.023** |
| - No, disabled | -5.1 (-11.5 – 1.3) | .117 | -4.1 (-10.3 – 2.2) | .203 |
| - Paid job | -0.34 (-3.6 – 3.0) | .841 | 0.45 (-2.8 – 3.7) | .786 |

Abbreviations: CI confidence interval; TA trait anxiety; N neuroticism; cT clinical tumor stage; pT pathological tumor stage;
Low educational level includes primary school, lower vocational, low or intermediate general education. Moderate educational level includes intermediate vocational education and higher general education. High educational level refers to higher vocational education and university.

**Table S5** Results from linear mixed model analysis on total arm function scores according to intention-to-treat principle

|  | **LMM without personality** |  | **LMM with personality** |  |
| --- | --- | --- | --- | --- |
| **Parameter** | **Regression coefficient (95% CI)** | ***P* value** | **Regression coefficient (95% CI)** | ***P* value** |
| **Time, (in time points)** | 1.3 (0.87 – 1.8) | **<.001** | 1.3 (-37.8 - -2.8) | **.023** |
| **Randomization arm** |  |  |  |  |
| - No-SLNB | Reference group |  | Reference group |  |
| - SLNB | 2.5 (0.31 – 4.7) | **.026** | 2.5 (0.31 – 4.6) | **.025** |
| **Age (in years)** | -0.03 (-0.21 – 0.15) | .759 | 0.02 (-0.2 – 0.2) | .857 |
| **Axillary treatment** |  |  |  |  |
| - No additional axillary treatment | Reference group |  | Reference group |  |
| - ALND or Regional RT | -2.4 (-8.0 – 3.1) | .391 | -2.2 (-7.7 – 3.2) | .419 |
| - ALND and regional RT | -9.3 (-27.4 – 8.9) | .317 | -9.1 (-26.8 – 8.5) | .311 |
| **(Neo)adjuvant treatment** |  |  |  |  |
| - No (neo-) adjuvant therapy | Reference group |  | Reference group |  |
| - Chemotherapy | 7.8 (-0.44 – 16.0) | .064 | 8.2 (0.13 – 16.2) | **.046** |
| - Hormonal therapy | -0.50 (-3.2 – 2.2) | .714 | -1.3 (-3.9 – 1.4) | .345 |
| - Chemo- and immunotherapy | -12.0 (-29.5 – 5.4) | .177 | -10.7 (-27.7 – 6.4) | .219 |
| - Chemo- and hormonal therapy | -1.3 (-5.5 – 2.9) | .548 | -1.0 (-5.2 – 3.1) | .623 |
| - Chemo-, immune- and hormonal therapy | 1.2 (-7.1 – 9.4) | .782 | 0.83 (-7.2 – 8.8) | .840 |
| **cT stage** |  |  |  |  |
| - cT1 | Reference group |  | Reference group |  |
| - cT2 | -0.13 (-4.2 – 4.0) | .950 | 0.67 (-3.4 – 4.7) | .745 |
| **pT stage** |  |  |  |  |
| - pT0 | Reference group |  | Reference group |  |
| - pT1 | 9.9 (-2.7 – 22.6) | .123 | 9.0 (-3.3 – 21.3) | .151 |
| - pT2 | 10.7 (-2.3 – 23.8) | .107 | 10.1 (-2.7 – 22.8) | .121 |
| - pTis | 1.8(-14.2 – 17.7) | .828 | 1.8 (-13.8 – 17.3) | .822 |
| **Personality** |  |  |  |  |
| - Low personality level |  |  | Reference group |  |
| - High TA level |  |  | 2.2 (-0.68 – 5.0) | .136 |
| - High N level |  |  | 13.1 (6.4 (19.9) | **<.001** |
| **Comorbidities (the amount of)** | 1.5 (0.73 – 2.2) | **<.001** | 1.2 (0.46 – 1.9) | **.001** |
| **Educational level** |  |  |  |  |
| - Low educational level | Reference group |  | Reference group |  |
| - Moderate educational level | 1.4 (-1.3 – 4.2) | .301 | 2.0 (-0.66 – 4.7) | .139 |
| - High educational level | -0.55 (-3.2 – 2.1) | .682 | -0.08 (-2.7 – 2.5) | .949 |
| **BMI** | 0.38 (0.17 – 0.59) | **<.001** | 0.39 (0.19 – 0.60) | **<.001** |
| **Smoking (no)** | -0.73 (-2.0 – 0.51) | .248 | -0.64 (-1.8 – 0.55) | .292 |
| **Marital status** |  |  |  |  |
| - Single | Reference group |  | Reference group |  |
| - Married | -0.46 (-4.7 – 3.8) | .831 | 0.39 (-3.8 – 4.6) | .854 |
| - Divorced/ widow | 0.63 (-4.3 – 5.6) | .804 | 1.6 (-3.2 – 6.5) | .510 |
| - Living together | -0.45 (-5.7 – 4.8) | .868 | 0.21 (-5.0 – 5.4) | .936 |
| - Other | 0.36 (-6.2 – 6.9) | .914 | 1.7 (-4.7 – 8.1) | .603 |
| **Children (the number of)** | 1.1 (-2.2 – 4.4) | .525 | 1.2 (-2.1 – 4.5) | .469 |
| **Employment status** |  |  |  |  |
| - No, no paid job | Reference group |  | Reference group |  |
| - No, retired | -4.0 (-7.4 - -0.52) | **.024** | -3.9 (-7.2 - -0.51) | **.024** |
| - No, disabled | -5.1 (-11.5 – 1.3) | .118 | -4.1 (-10.3 – 2.2) | .205 |
| - Paid job | -0.38 (-3.7 – 2.9) | .821 | 0.41 (-2.9 – 3.7) | .807 |

Abbreviations: CI confidence interval; TA trait anxiety; N neuroticism; cT clinical tumor stage; pT pathological tumor stage;
Low educational level includes primary school, lower vocational, low or intermediate general education. Moderate educational level includes intermediate vocational education and higher general education. High educational level refers to higher vocational education and university

**Table S6** Results linear mixed model analysis on global HRQoL scores according to the as-treated principle

|  | **LMM without personality groups** |  | **LMM with personality groups** |  |
| --- | --- | --- | --- | --- |
| **Parameter** | **Regression coefficient (95% CI)** | ***P* value** | **Regression coefficient (95% CI)** | ***P* value** |
| **Time, (in time points)** | -0.45 (-1.2 – 0.25) | .205 | -0.45(-1.2 – 0.25) | .203 |
| **Treatment arm** |  |  |  |  |
| - No-SLNB | Reference group |  | Reference group |  |
| - SLNB | -0.90 (-3.9 – 2.1) | .557 | -0.80 (-3.8 – 2.2) | .602 |
| **Age (in years)** | -0.01 (-0.26 – 0.24) | .942 | -0.05 (-0.30 – 0.19) | .685 |
| **Axillary treatment** |  |  |  |  |
| - No additional axillary treatment | Reference group |  | Reference group |  |
| - ALND or Regional RT | 0.90 (-6.6 – 8.4) | .815 | 0.63 (-6.8 – 8.1) | .868 |
| - ALND and regional RT | 17.8 (-6.7 – 42.2) | .154 | 17.6 (-6.6 – 41.8) | .154 |
| **Neo-adjuvant treatment** |  |  |  |  |
| - No (neo)-adjuvant treatment | Reference group |  | Reference group |  |
| - Chemotherapy | -12.1 (-23.2 - -0.91) | **.034** | -12.4 (-23.4 – -1.3) | **.028** |
| - Hormonal therapy | -0.07 (-3.7 – 3.6) | .969 | -0.63 (-3.0 – 4.3) | .736 |
| - Chemo- and immunotherapy | -7.8 (-31.5 – 15.8) | .515 | -8.9 (-32.3 – 14.5) | .456 |
| - Chemo- and hormonal therapy | 1.4 (-4.3 – 7.1) | .634 | -1.2 (-5.5 – 6.8) | .685 |
| - Chemo-, immune- and hormonal therapy | -3.0 (-14.2 – 8.1) | .593 | -2.8 (-13.8 – 8.3) | .622 |
| **cT stage** |  |  |  |  |
| - cT1 | Reference group |  | Reference group |  |
| - cT2 | 3.7 (-1.9 – 9.2) | .192 | 2.9 (-2.6 – 8.4) | .297 |
| **pT stage** |  |  |  |  |
| - pT0 | Reference group |  | Reference group |  |
| - pT1 | 3.8 (-13.2 – 20.8) | .660 | 4.8 (-11.9 – 21.6) | .572 |
| - pT2 | 3.6 (-14.0 – 21.1) | .692 | 4.3 (-13.1 – 21.7) | .626 |
| - pTis | 7.9 (-13.9 – 29.6) | .479 | 7.9 (-13.7 – 29.4) | .474 |
| **Personality group** |  |  |  |  |
| - Low TA and N group |  |  | Reference group |  |
| - High TA group |  |  | -1.8 (-5.6 – 2.0) | .350 |
| - High N group |  |  | -12.1 (-21.5 – -2.6) | **.012** |
| **Comorbidities (the amount of)** | -3.1 (-4.1 – -2.1) | **<.001** | -2.9 (-3.9 – -1.8) | **<.001** |
| **Educational level** |  |  |  |  |
| - Low educational level | Reference group |  | Reference group |  |
| - Moderate educational level | -5.0 (-8.7 - -1.3) | **.009** | -5.5 (-9.2 – -1.8) | **.004** |
| - High educational level | -1.7 (-5.3 – 2.0) | .365 | -2.0 (-5.6 – 1.6) | .266 |
| **BMI** | -0.30 (-0.59 - -0.02) | **.038** | -0.31 (-0.6 – -0.3) | **.030** |
| **Smoking (no)** | -0.72 (-2.4 – 1.0) | .398 | -0.81 (-2.5 – 0.8) | .338 |
| **Marital status** |  |  |  |  |
| - Single | Reference group |  | Reference group |  |
| - Married | 0.67 (-5.4 – 6.7) | .827 | -0.11 (-6.1 – 6.0) | .972 |
| - Divorced/ widow(er) | 0.97 (-5.9 – 7.8) | .781 | 0.05 (-6.8 – 6.9) | .813 |
| - Living together | -2.8 (-10.1 – 4.4) | .441 | -3.4 (-10.6 – 3.8) | .351 |
| - Other | -4.7 (-13.5 – 4.2) | .303 | -5.8 (-14.6 – 3.0) | .198 |
| **Children (the number of)** | -1.7 (-6.4 – 3.0) | .470 | -1.9 (-6.6 – 2.9) | .441 |
| **Employment status** |  |  |  |  |
| - No, no paid job | Reference group |  | Reference group |  |
| - No, retired | 3.0 (-1.7 – 7.6) | .215 | 2.9 (-1.8 – 7.5) | .223 |
| - No, disabled | -8.3 (-17.0 – 0.44) | .063 | -9.3 (-18.0 – -0.6) | **.035** |
| - Paid job | 0.29 (-4.3 – 4.9) | .901 | -0.47 (-5.1 – 4.1) | .841 |

Abbreviations: CI confidence interval; TA trait anxiety; N neuroticism; cT clinical tumour stage; pT pathological tumour stage;
Low educational level includes primary school, lower vocational, low or intermediate general education. Moderate educational level includes intermediate vocational education and higher general education. High educational level refers to higher vocational education and university.

**Table S7** Results from linear mixed model analysis on global HRQoL scores according to intention-to-treat principle

|  | **LMM without personality** |  | **LMM with personality** |  |
| --- | --- | --- | --- | --- |
| **Parameter** | **Regression coefficient (95% CI)** | ***P* value** | **Regression coefficient (95% CI)** | ***P* value** |
| **Time, (in time points)** | -0.45 (-1.2 – 0.25) | .205 | -0.45 (-1.2 – 0.25) | .203 |
| **Randomization arm** |  |  |  |  |
| - No-SLNB | Reference group |  | Reference group |  |
| - SLNB | -0.77 (-3.8 – 2.2) | .615 | -0.69 (-3.7 – 2.3) | .652 |
| **Age (in years)** | -0.01 (-0.25 – 0.24) | .950 | -0.05 (-0.30 – 0.20) | .692 |
| **Axillary treatment** |  |  |  |  |
| - No additional axillary treatment | Reference group |  | Reference group |  |
| - ALND or Regional RT | 0.83 (-6.7 – 8.4) | .830 | 0.57 (-6.9 – 8.0) | .881 |
| - ALND and regional RT | 17.7 (-6.8 – 42.1) | .156 | 17.5 (-6.7 – 41.7) | .156 |
| **(Neo)adjuvant treatment** |  |  |  |  |
| - No (neo-) adjuvant therapy | Reference group |  | Reference group |  |
| - Chemotherapy | -12.0 (-23.2 - -0.90) | **.034** | -12.4 (-23.4 - -1.3) | **.028** |
| - Hormonal therapy | -0.07 (-3.7 – 3.6) | .970 | 0.63 (-3.0 – 4.3) | .735 |
| - Chemo- and immunotherapy | -7.8 (-31.4 – 15.8) | .518 | -8.9 (-32.3 – 14.6) | .458 |
| - Chemo- and hormonal therapy | 1.4 (-4.3 – 7.1) | .632 | 1.2 (-4.5 – 6.9) | .686 |
| - Chemo-, immune- and hormonal therapy | -3.0 (-14.2 – 8.1) | .594 | -2.8 (-13.8 – 8.3) | .622 |
| **cT stage** |  |  |  |  |
| - cT1 | Reference group |  | Reference group |  |
| - cT2 | 3.7 (-1.9 – 9.2) | .197 | 2.9 (-2.6 – 8.4) | .302 |
| **pT stage** |  |  |  |  |
| - pT0 | Reference group |  | Reference group |  |
| - pT1 | 3.9 (-13.1 – 20.9) | .653 | 4.9 (-11.9 – 21.7) | .566 |
| - pT2 | 3.6 (-13.9 – 21.2) | .686 | 4.4 (-13.0 – 21.8) | .621 |
| - pTis | 7.9 (-13.8 – 29.7) | .476 | 7.9 (-13.6 – 29.4) | .471 |
| **Personality** |  |  |  |  |
| - Low personality level |  |  | Reference group |  |
| - High TA level |  |  | -1.8 (-5.6 – 2.0) | .353 |
| - High N level |  |  | -12.1 (-21.5 - -2.7) | **.012** |
| **Comorbidities (the amount of)** | -3.1 (-4.1 - -2.1) | **<.001** | -2.8 (-2.9 - -1.8) | **<.001** |
| **Educational level** |  |  |  |  |
| - Low educational level | Reference group |  | Reference group |  |
| - Moderate educational level | -5.0 (-8.7 - -1.3) | **.008** | -5.5 (-9.2 - -1.8) | **.004** |
| - High educational level | -1.7 (-5.3 – 1.9) | .363 | -2.1 (-5.7 – 1.5) | .264 |
| **BMI** | -0.30 (-0.59 - -0.02) | **.039** | -0.32 (-0.61 - -0.03) | **.032** |
| **Smoking (no)** | -0.72 (-2.4 – 1.0) | .402 | -0.80 (-2.5 – 0.85) | .342 |
| **Marital status** |  |  |  |  |
| - Single | Reference group |  | Reference group |  |
| - Married | 0.65 (-5.4 – 6.7) | .833 | -0.14 (-6.2 – 5.9) | .965 |
| - Divorced/ widow | 0.93 (-5.9 - 7.8) | .791 | 0.01 (-6.8 – 6.9) | .997 |
| - Living together | -2.8 (-10.1 – 4.4) | .444 | -3.4 (-10.6 – 3.8) | .354 |
| - Other | -4.6 (-13.5 – 4.3) | .309 | -5.7 (-14.6 – 3.1) | .203 |
| **Children (the number of)** | -1.7 (-6.4 – 3.0) | .474 | -1.8 (-6.5 – 2.9) | .445 |
| **Employment status** |  |  |  |  |
| - No, no paid job | Reference group |  | Reference group |  |
| - No, retired | 3.0 (-1.7 – 7.6) | .217 | 2.9 (-1.8 – 7.5) | .225 |
| - No, disabled | -8.3 (-17.1 – 0.43) | .062 | -9.4 (-18.1 - -.66) | **.035** |
| - Paid job | 0.30 (-4.3 – 4.9) | .899 | -0.46 (-5.1 – 4.1) | .844 |

Abbreviations: CI confidence interval; TA trait anxiety; N neuroticism; cT clinical tumor stage; pT pathological tumor stage;
Low educational level includes primary school, lower vocational, low or intermediate general education. Moderate educational level includes intermediate vocational education and higher general education. High educational level refers to higher vocational education and university.
