# Supplementary material for: Three-year patient-reported outcomes of the BOOG 2013-08 RCT evaluating omission of sentinel lymph node biopsy in early-stage breast cancer patients treated with breast conserving surgery: Impact of personality traits on health-related quality of life
Source: Br J Surg. 2025 May 14;112(5):znaf031. doi: 10.1093/bjs/znaf031 (PMC12076147; doi:10.1093/bjs/znaf031)
Supplement: znaf031_Supplementary_Data [file znaf031_supplementary_data.zip › Trial_protocol_BOOG.docx]

**RESEARCH PROTOCOL**


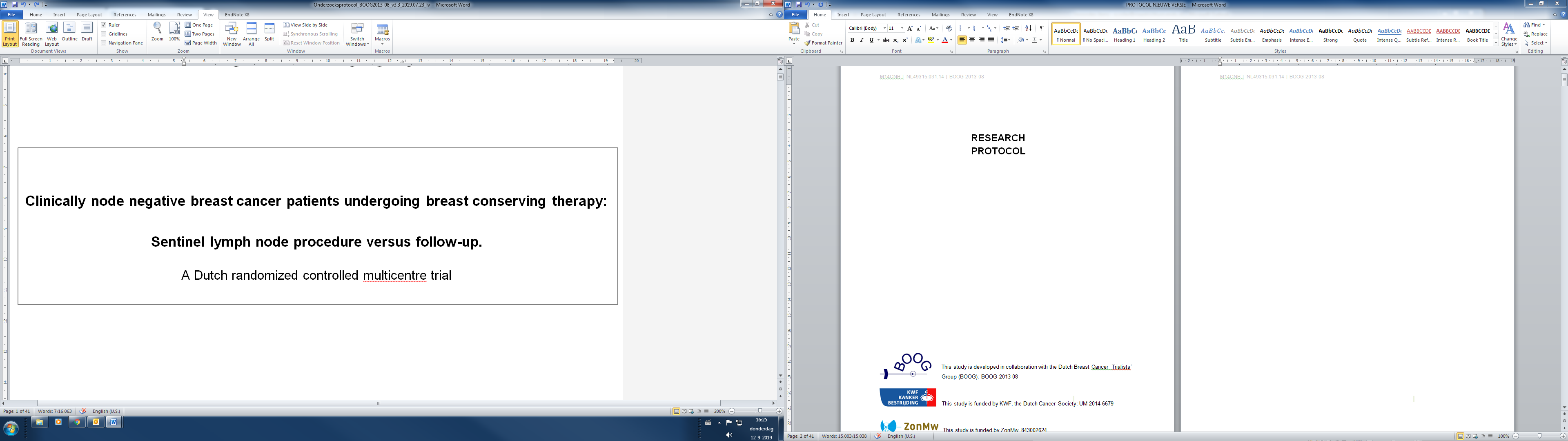


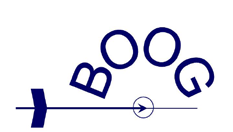


This study is developed in collaboration with the Dutch Breast Cancer Trialists´ Group (BOOG): BOOG 2013-08


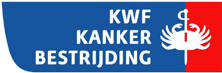


This study is funded by KWF, the Dutch Cancer Society: UM 2014-6679


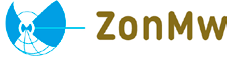


This study is funded by ZonMw, 843002624

**PROTOCOL TITLE** Clinically node negative breast cancer patients undergoing breast conserving

therapy: Sentinel lymph node procedure versus follow-up. A Dutch
randomized controlled multicentre trial.

**Short title** BOOG 2013-08

**Version** 3.3

**Date** July 23th, 2019

**Study coordinators ML Smidt, MD, PhD**

**and principal** Maastricht University Medical Centre+

**investigators**  Department of Surgical Oncology

P.O. Box 5800, 6202 AZ Maastricht
Tel: +31 (0)43 387 74 77

Fax: +31 (0)43 387 54 73

E-mail: m.smidt@mumc.nl

PhD via: mamma.research@mumc.nl
Tel: +31 6 40244513

**JHW de Wilt, MD, PhD**

Radboud University Medical Centre

Department of Surgical Oncology
P.O. Box 9101, 6500 HB Nijmegen

Tel: +31 (0)24 361 73 65

Fax: +31 (0)24 354 05 01
E-mail: hans.dewilt@radboudumc.nl

**Co-Principal E Colier, MD**

**Investigator** Maastricht University Medical Centre+

Department of Surgical Oncology

P.O. Box 5800, 6202 AZ Maastricht
Tel: +31 (0)43 387 74 77

Fax: +31 (0)43 387 54 73

E-mail: evie.colier@mumc.nl

**Sponsor Maastricht University Medical Centre+**

**Independent expert SO Breukink, MD, PhD**
 Maastricht University Medical Centre+

Department of Surgery

Tel: +31 (0)43 387 54 92
Fax: +31 (0)43 387 54 73
E-mail: s.breukink@mumc.nl

**Coordinating Data IKNL Clinical Trial Centre**

**Centre** Tel: +31 (0)88 234 65 00

E-mail: trialbureau@iknl.nl

| **Name** | **Site** | **Signature** | **Date** |
| --- | --- | --- | --- |
| **Principal Investigators**  M.L. Smidt  J.H.W. de Wilt | Maastricht University Medical Centre+  Radboud University Medical Centre | 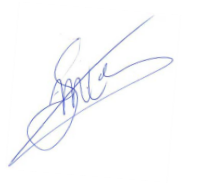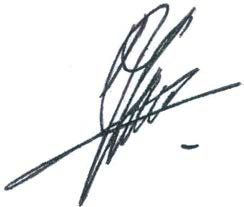 | 23-07-2019  23-07-2019 |
| **Co-principal Investigator**  E. Colier | Maastricht University Medical Centre+ | 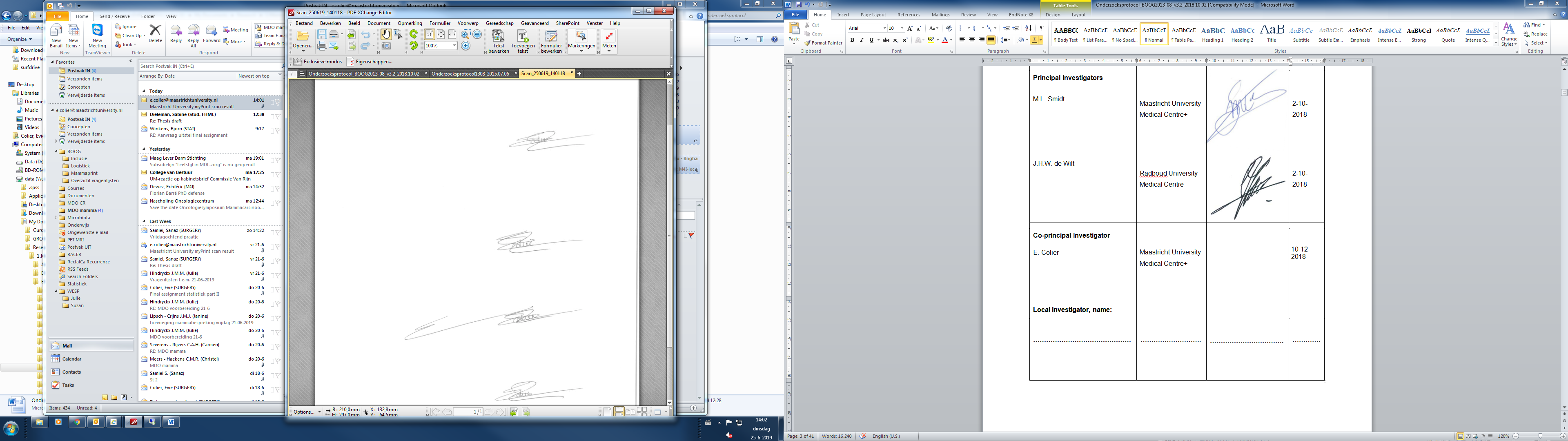 | 23-07-2019 |
| **Local Investigator, name:**  **………………………………………** | **……………………….** | **…………………………….** | **………….** |

**Writing committee**

***Surgical Oncology***

Smidt ML MD, PhD Maastricht University Medical Centre+ Maastricht

De Wilt JHW MD, PhD Radboud University Medical Centre Nijmegen

Van Roozendaal LM MD, PhD Maastricht University Medical Centre+ Maastricht

Strobbe LJA MD, PhD Canisius-Wilhelmina Hospital Nijmegen

Van der Hage JA MD, PhD Antoni van Leeuwenhoek Hospital Amsterdam

Van Dalen T MD, PhD Diakonessenhuis Hospital Utrecht

***Medical Oncology***

Tjan-Heijnen VCG MD, PhD Maastricht University Medical Centre+ Maastricht

Linn SC MD, PhD Antoni van Leeuwenhoek Hospital Amsterdam

***Radiation Oncology***

Boersma LJ MD, PhD MAASTRO clinic Maastricht

Westenberg AH MD Arnhem lnstitute for Radiation Oncology Arnhem

Poortmans PMP MD, PhD Radboud university medical centre Nijmegen

***Breast Radiology***

Lobbes MBI MD, PhD Maastricht University Medical Centre+ Maastricht

***Breast Pathology***

Van de Vijver KKBT MD, PhD Antoni van Leeuwenhoek Hospital Amsterdam

***Medical Psychology***

De Vries J PhD Tilburg University Tilburg

***Statistics/methodology***

Kessels AGH PhD Maastricht University Medical Centre+ Maastricht

**CONTENTS**

1. ABBREVIATIONS AND RELEVANT DEFINITIONS ........................................................................ 6
2. SUMMARY ........................................................................................................................................ 7
3. INTRODUCTION ............................................................................................................................... 9
4. OBJECTIVES .................................................................................................................................. 16
5. STUDY DESIGN ............................................................................................................................. 17
   1. Description of the study design .................................................................................................. 17
   2. Schematic study design ............................................................................................................. 17
   3. Timeline ..................................................................................................................................... 18
6. STUDY POPULATION .................................................................................................................... 19
   1. Population .................................................................................................................................. 19
   2. Inclusion criteria ......................................................................................................................... 19
   3. Exclusion criteria ........................................................................................................................ 19
   4. Sample size calculation ............................................................................................................. 19
7. METHODS ...................................................................................................................................... 21
   1. Study endpoints ......................................................................................................................... 21
   2. Study procedures - diagnosis and randomization ..................................................................... 22
   3. Study procedures - treatment .................................................................................................... 24
   4. Study procedures - follow-up ..................................................................................................... 27
   5. Study procedures - axillary morbidity rate and quality of life ..................................................... 28
   6. Cost-effectiveness ...................................................................................................................... 29
   7. Burden on informal caregivers of breast cancer patients .......................................................... 30
   8. Withdrawal of individual subjects ............................................................................................... 30
   9. Premature termination of the study ........................................................................................... 30
   10. Statistical analysis ..................................................................................................................... 30
8. SAFETY REPORTING .................................................................................................................... 31
   1. Section 10 WMO event .............................................................................................................. 31
   2. Adverse events .......................................................................................................................... 31
   3. Data Safety Monitoring Board (DSMB) ...................................................................................... 31
   4. Stopping rule .............................................................................................................................. 32
9. ETHICAL CONSIDERATIONS ....................................................................................................... 33
   1. Regulation statement ................................................................................................................. 33
   2. Recruitment and consent ........................................................................................................... 33
   3. Benefits and risk assessment .................................................................................................... 33
   4. Compensation for injury ............................................................................................................. 34
10. ADMINISTRATIVE ASPECTS AND PUBLICATION ...................................................................... 35
    1. Handling and storage of data ..................................................................................................... 35
    2. Amendments .............................................................................................................................. 35
    3. Annual Progression Report ........................................................................................................ 35
    4. Final Report ............................................................................................................................... 36
    5. Public disclosure and publication policy .................................................................................... 36
11. REFERENCES ................................................................................................................................ 37
12. ANNEX ............................................................................................................................................ 41

**1. ABBREVIATIONS AND RELEVANT DEFINITIONS**

**ALND** Axillary lymph node dissection

**BIA** Budget impact analysis

**CEA** Cost-effectiveness analysis

**cN0** Clinically node negative: no signs of axillary lymph node metastases at physical
examination and preoperative axillary ultrasound (or negative cyto-/histopathology) Clinically (physical examination and imaging) tumour size of 2 cm or less in greatest dimension

**cT2** Clinically (physical examination and imaging) tumour size of more than 2 cm but not more than 5 cm in greatest dimension

**ER** Estrogen receptor

**IC** Informed caregiver

**METC** Medical Research Ethics Committee (MREC); in Dutch: Medisch Ethische Toetsing Commisie (METC)

**SLN** Sentinel lymph node

**TNM** TNM classification 7^th^ edition. T: Primary tumour; N: Regional lymph nodes; M: Distant metastases

**WMO** Medical Research Involving Human Subjects Act; in Dutch: Wet Medisch-wetenschappelijk Onderzoek met Mensen (WMO)

**2. SUMMARY**

**Rationale -** NSABP B-04 trial revealed that omitting primary axillary treatment of occult positive lymph nodes in clinically node negative breast cancer patients does not affect survival, even after 25 years of follow-up and without use of adjuvant systemic or radiation therapy. Delayed axillary dissection in case lymph nodes become clinically positive does not affect survival and prevents axillary overtreatment in majority of patients. The ACOSOG Z0011 and IBCSG 23-01 trials revealed that completion axillary dissection can be safely omitted in clinically node negative patients with metastatic sentinel nodes. Patients randomized for watchful waiting were likely to have residual nodal disease (13-27%) that was not surgically removed. Nevertheless, survival rates were not affected and regional recurrence rates low. Use of axillary ultrasound will improve preoperative selection of node negative patients, as it selects patients with a more favourable tumour load and accurately excludes advanced nodal disease (≥4 metastatic nodes). Biology and adjuvant systemic and radiation therapy are factors that most likely diminish the risk that possible metastases left in situ develop into clinically detectable lymph nodes. Patients treated with breast conserving therapy are significantly more often diagnosed with pathologic node negative disease or with micrometastatic disease, compared to patients treated with mastectomy. Risk of occult (macro)metastases in cT1-2N0 breast cancer patients treated with breast conserving therapy is low: about 88% will be node negative and about 95% of node positive patients will have no lymph node metastases beyond the sentinel node. Reflected by the low regional recurrence rate after a (false) negative sentinel node, only a small amount of patients (0.8%) with node positive disease is expected to develop clinically detectable nodal disease.

**Objectives -** The primary objective of this study is to determine whether omitting the sentinel lymph node procedure is non-inferior to the current axillary staging regimens in clinically node negative breast cancer patients undergoing breast conserving therapy, in terms of regional recurrence rate.

**Study design -** A Dutch prospective non-inferiority randomized controlled multicentre trial.

**Study population** - Women aged 18 years or older diagnosed with unilateral cT1-2N0 invasive breast cancer with a negative preoperative axillary ultrasound (or negative cyto-/histology).

**Intervention -** Eligible patients will be randomized for one of the following treatment groups:

Arm A (control arm): lumpectomy with sentinel lymph node procedure, followed by radiotherapy of the breast with or without completion axillary treatment according to the Dutch guideline.

Arm B (study arm): lumpectomy without further axillary staging, followed by radiotherapy of the breast.

**Main study endpoints -** Regional recurrence rate is the primary endpoint, and distant-disease free survival, overall survival, delayed axillary lymph node dissections, axillary morbidity, quality of life, local recurrence, contralateral breast cancer, and percentage difference in administered (neo)adjuvant systemic therapy and cost-effectiveness are secondary endpoints, analyzed after 1, 3-, 5- and 10 years of follow-up^1^. Prior data indicate 5-year regional recurrence free survival rate of 99% among controls and of 96% among study subjects, resulting in a sample size of 747 per arm, to be able to reject the null hypothesis that the rate for experimental and control subjects is inferior by at least 5% (delta = -5%) with probability of 0.8.

**Hypothesis -** The sentinel lymph node procedure can be safely omitted in clinically node negative breast cancer patients undergoing breast conserving therapy. This will lead to a decreased axillary morbidity rate, with non-inferior regional control, distant-disease free survival and overall survival.

**Nature and extend of the burden, risks and benefit associated with participation -** Treatment procedures are all performed according to the Dutch breast cancer guideline. No patient will encounter any delay in their treatment as a result of inclusion. No additional interventions will be performed, only less if randomized for the study arm. Possible burden and risks for patients in study arm: worse regional recurrence rate, overall survival or distant-disease free survival and performance of delayed axillary lymph node dissection. Possible benefit for patients in study arm: lower axillary morbidity rate, less axillary surgery, shorter recovery time and improved quality of life.

Any additional burden for all the patient will only consist of completing quality of life (QoL) and cost-effectiveness questionnaires. Since the inclusion rate of the cost effectiveness analysis is approximately 66%, more patients than the previously estimated 700 need to be included in the QoL study. The cost-effectiveness analysis requires 474 patients; If the inclusion rate continues at 66%, 1056 patients are needed. This means that another 105 patients need to be approached for both the QoL and cost-effectiveness study.

**3. INTRODUCTION**

Breast cancer is by far the most common cancer among women worldwide, with 1.38 million newly diagnosed breast cancer patients in 2008 [1, 2]. The highest incidence rates of breast cancer are reported in Western countries. Survival rates have increased in recent years thanks to more extensive (adjuvant) treatment regimens [3, 4]. Consequently, more attention is warranted for overtreatment of breast cancer patients resulting in lifetime morbidity.

We propose a randomized controlled multicentre trial to investigate whether the sentinel lymph node procedure can be omitted in clinically node negative breast cancer patients treated with breast conserving therapy. We aim to significantly decrease the number of breast cancer patients receiving overtreatment of the axilla, thereby diminishing the risk for complications, while maintaining excellent regional control and not affecting survival.

**Axillary lymph node dissection**
For a long time the standard procedure to assess the axillary lymph node status in breast cancer patients was an axillary lymph node dissection (ALND). An ALND is associated with significant short- and long- term morbidity. Most reported complications are seroma (15-75%), lymph oedema (20%), nerve injury (55-75%) and reduced shoulder function (16%) [5-7].

The supposed therapeutic effect of the ALND - improving overall survival and maintaining regional control - has been questioned for a long time, substantiated among others by the National Surgical Adjuvant Breast and Bowel Project (NSABP) B-04 trial with 25 years of follow-up, which was initiated in 1971. This trial aimed to determine whether clinically node negative breast cancer patients who received local or regional treatments other than radical mastectomy (total mastectomy plus ALND) would have outcomes similar to those achieved with radical mastectomy. A total of 1079 breast cancer patients, who were clinically node negative (based purely on physical examination) with a mean tumour size of 3.3 cm (± 2 cm), were randomly assigned to undergo radical mastectomy, or mastectomy with postoperative axillary radiation, or mastectomy followed by delayed ALND for those patients who subsequently develop clinically apparent nodes. None of the patients received adjuvant systemic therapy [8].

In the radical mastectomy group, the ALND specimen contained lymph node metastases in about 40% of the women. Because all women were randomly assigned to the different treatment groups, about 40% of those who underwent mastectomy alone are estimated to have had positive nodes that were not removed at the time of initial surgery. During follow-up of the mastectomy only group, involved ipsilateral nodes became clinically apparent in 68 of 365 women (18.6%). Thus, occult lymph node metastases never became clinically positive in 53.5% of patients (21.4% of 40%). The clinically detected metastatic nodes were removed by a delayed ALND within 2 years in 75.0%, in year 2-5 in 14.7%, in year 5-10 in 8.8% and in year 10-25 in 1.5% (mean 14.8 months [3.0-134.5 months]).

After 25 years of follow-up, the (actual) regional recurrence rate (including supra- and subclavicular, internal mammary and ipsilateral axillary nodes) was significant different (p=0.002) between the three groups, with 4% (15/362) in the radical mastectomy group, 4% (15/352) in the mastectomy with postoperative radiation group and 6% (23/365) in the mastectomy only group. Despite the need for a delayed ALND in 18.6% of patients in the mastectomy only group and the significantly different regional recurrence rate, no significant differences were observed among the three groups with respect to overall survival and disease-free survival (p=0.68 and 0.65 respectively).

In conclusion, the NSABP B-04 randomized controlled trial revealed that omitting primary axillary treatment of occult positive lymph nodes in clinically node negative breast cancer patients does not

affect survival, even after 25 years of follow-up and without the use of adjuvant systemic or radiation therapy. The performance of a delayed ALND in case axillary lymph nodes become clinically positive does not affect survival and prevents axillary overtreatment in the majority of patients. Despite these favourable results, the ALND remained to be the standard procedure to assess the axillary lymph node status, partly due to the in that time newly recognised value of adjuvant systemic therapy that appeared to be mainly beneficial for node-positive breast cancer patients.

**Sentinel lymph node procedure**

In the past 10 years, the sentinel lymph node (SLN) procedure has become the standard, less invasive technique to evaluate the nodal status in clinically node negative breast cancer patients. This procedure is based on the pattern of lymphatic drainage to one or more regional lymph nodes (the SLN’s), assuming that if these SLN’s contain no metastatic disease, all other regional lymph nodes are negative as well. High accuracy, low false-negative rates and safety of the SLN procedure were proven by several studies and randomized controlled trials [6, 9]. In the general population, the SLN is negative in 74% of clinically node negative patients [10, 11]. Axillary surgery could therefore have been avoided and is thus overtreatment in the majority of clinically node negative patients.

The SLN procedure is often referred to as a simple fifteen-minute operation, although that does not include the performance of the lymphoscintigraphies prior to the procedure. Besides, the SLN’s are not infrequently removed (under local anaesthesia) prior to the breast surgery, making it a totally separate procedure. Further, complications occur in 25% of patients treated with the SLN procedure and breast conserving therapy; most reported are axillary seroma, wound infections, hematoma, anaphylactic reaction, lymphoedema, and axillary paraesthesia [12-15]. Severe lymphoedema occurs in still 8% after a follow-up of only 3 years [16]. The total costs of the procedure and additional costs for complications are substantial.

According to a meta-analysis, the false negative rate for the SLN procedure ranges from 0-40%, with a median of 7% [17]. So in theory, lymph node metastases are left in situ in 2.8% of clinically node negative breast cancer patients (7% of 40%). This rate however, did not result in the expected regional recurrence rate of 4.45% (2.8% / 62.8%) after a negative SLN procedure. In contrast, after a negative SLN procedure the regional recurrence rate amounts only 0.3% (median follow-up was 34 months; median time interval to the recurrence was 20 months (range 4-63 months)) [18]. So, the true regional recurrence rate is 14.86 times lower (4.45% / 0.3%) than expected. So only a small amount of patients with false negative SLN’s will develop clinically positive nodes.

Several retrospective studies assessed the difference in recurrence and survival for SLN procedure alone versus SLN procedure with completion ALND in SLN positive breast cancer patients. Most studies concluded that neither the regional recurrence rate nor overall survival was significantly improved in the completion ALND group [19-26]. However, selection bias might have influenced the results of these retrospective studies.

**Results of the ACOSOG Z0011 trial: omitting ALND following positive sentinel lymph nodes**
Recently, the results of the American College of Surgeons Oncology Group (ACOSOG) Z0011 trial were published. This randomized controlled trial aimed to determine the effects of omitting completion ALND on overall survival in patients with SLN metastases. The study included patients undergoing breast conserving therapy (i.e. lumpectomy followed by whole breast radiation therapy), who were clinically node negative (based on physical findings only), had a primary tumour size of less than 5 cm and one or two metastatic SLN’s [27]. Patients with SLN metastases only identified by immunohistochemical staining were excluded.

Patients were randomly assigned to undergo completion ALND (control arm) or watchful waiting (study arm). In the control arm, 97 of 355 patients (27.3%) had additional lymph node metastases removed by ALND. At a median follow-up of 6.3 years (range 5.2-7.7), the overall survival and disease-free survival rate was 91.8% and 82.8% in the completion ALND arm, and 92.5% and 83.9% in the watchful waiting arm. A regional recurrence rate of 0.5% (2 events) was observed in the completion ALND arm and of 0.9% (4 events) in the SLN procedure only arm. All differences between groups were non-significant. It was concluded that omitting the completion ALND in this population does not result in inferior survival results nor a higher regional recurrence rate.

Findings of the ACOSOG Z0011 trial are supported by the results of the International Breast Cancer Study Group (IBCSG) 23-01 trial. This randomized controlled trial investigated whether no ALND was non-inferior to ALND in clinically node negative patients with one or more micrometastatic sentinel nodes and a primary tumour of 5 cm or less. Both patients treated with breast conserving therapy and mastectomy were included. In the ALND group, 11% of patients had additional nodal disease in the ALND specimen. However, the 5-year regional recurrence rate was only 1% and overall survival was not affected, in accordance to other studies [28].

The current Dutch breast cancer guideline (2012) implemented the results of the ACOSOG Z0011, describing that omitting completion ALND may be considered when adjuvant systemic therapy and breast conserving therapy are performed [29]. Safety of omitting completion axillary treatment in mastectomy patients will be further employed in the Dutch multicentre trial entitled “The value of completion axillary treatment in sentinel node positive breast cancer patients undergoing a mastectomy”, designed by our writing committee and the Dutch Breast Cancer Trialists´ Group.

In conclusion, the ACOSOG Z0011 and the IBCSG 23-01 randomized controlled trials revealed that completion ALND can be safely omitted in clinically node negative breast cancer patients with metastatic sentinel nodes. The ALND specimen of patients in the control arm contained additional nodal metastases beyond the SLN metastasis in 11-27%. Patients randomized to the SLN only arm were therefore likely to have residual nodal disease that was not surgically removed. Nevertheless, survival rates were not affected and regional recurrence rates low.

**Preoperative axillary ultrasound**

Physical examination and the axillary ultrasound are performed routinely in the Netherlands to assess the axillary lymph node status prior to the SLN procedure or ALND. If the patient’s axillary staging is negative, the SLN procedure is performed for further staging. If a suspicious lymph node is detected by ultrasound, this node will be sampled and if pathology shows a metastasis, the SLN procedure is omitted and an ALND is indicated. The ALND can then be performed simultaneously with the tumour excision. This approach prevents 19.8% of breast cancer patients from having to undergo an additional operation [30].

Clinically node negative breast cancer patients in the NSABP B-04 and the ACOSOG Z0011 trial were selected by physical examination. The accuracy of physical examination of the axilla for pre-operative staging is low, with a sensitivity of 25-32.3% for detecting axillary metastases [31-34]. Preoperative staging with axillary ultrasound selects node negative patients more accurately. The sensitivity of axillary ultrasound combined with biopsy is 79.6%, with a specificity of 98.3% [30]. A prospective study of Rautiainen et al. showed that the sensitivity of axillary ultrasound combined with fine-needle aspiration is 72.5%, and 88.2% when core-biopsy is used (specificity 100% for both methods) [35]. Furthermore, an axillary ultrasound helps to select patients with a more favourable tumour load, as the total number of lymph node metastases per patient is significantly lower after a negative axillary ultrasound compared to when the clinically node negative status is based solely on physical examination [36]. Besides, a negative axillary ultrasound accurately excludes advanced axillary nodal disease (≥4 lymph node metastases), with a negative predictive value of 93-96% in the general breast cancer population, according to two single-centre studies [37, 38]. In a Dutch nationwide cohort study it was investigated whether the axillary ultrasound performed in the Netherlands accurately excludes advanced nodal disease in patients who meet the criteria of the BOOG 2013-08 trial. A total of 12113 patients were included from 90 Dutch centres, of whom 208 had a pN2-3 status as final diagnosis (≥4 lymph node metastases), resulting in a negative predictive value of 98.1% for a negative axillary ultrasound to exclude advanced nodal disease (data submitted to the Dutch Journal of Oncology, July 2014).

**Low regional recurrence rate**

The Oxford overview analysis of the Early Breast Cancer Trialists’ Collaborative Group (EBCTCG) shows that the 10-year regional recurrence rates have always been relatively low for patients treated with breast conserving surgery. The supplementary webappendix of this meta-analysis nicely presents that the regional recurrence rate is decreased by adjuvant therapy, however more subtle when compared to, for instance, the local recurrence rate [39]. In the section below is described why a low regional recurrence rate in our proposed study is expected.

***Biology*:** In the NSABP B-04 trial, less than half of the patients with nodal metastases (based on the incidence of metastases in the ALND arm) developed clinically apparent regional recurrence [8]. None of these patients received adjuvant systemic or radiation therapy. It is therefore likely that the biology of lymph node metastases plays an important role in the growth of metastases in the axilla.

***Adjuvant systemic therapy*:** At present, adjuvant systemic therapy (i.e. chemo-, endocrine- and immunotherapy) is administered in both node positive and increasingly in node negative patients due to the development of more effective and targeted systemic therapy. Adjuvant systemic therapy is known to decrease local and regional recurrence rates [39]. Reported pathologic complete response rates for axillary lymph node metastases following primary systemic therapy of about 20-40% demonstrate that systemic therapy eradicates lymph node metastases left in situ [40-44].

The SLN procedure is omitted in the study arm of our proposed study. Lack of knowledge concerning the pathologic nodal status might have an impact on the adjuvant treatment recommendations in our study population. The impact however might not be as high as expected, because the lymph node status is no longer the only indicator for adjuvant systemic therapy but has become mostly a prognostic factor in breast cancer patients. Instead, clinicopathologic characteristics are now more and more used to recommend adjuvant systemic therapy. In a retrospective cohort of clinically node negative breast cancer patients with a primary tumour size of ≤5 cm who were treated with breast conserving therapy at Maastricht University Medical Centre+, we simulated the actual impact of omitting the SLN procedure on adjuvant treatment recommendations. We found that without pathologic lymph node status, adjuvant treatment recommendation changed in 1.0-3.6% of the patients (using Adjuvant! Online or the 2012 Dutch breast cancer guideline, respectively) [45]. This difference seems reasonable and will most likely not affect survival, nor disease control in this patient population.

The use of traditional prognostic factors as described above is helpful in the adjuvant treatment recommendations, but not optimal because breast cancer is a heterogeneous disease. A more accurate way to predict patient prognosis would be molecular profiling. Several gene-expression profiles have been developed with the aim to better define patient prognosis and additionally to better select patients who most likely benefit from adjuvant chemotherapy, compared to when traditional predictive factors are used. These signatures have shown that some patients do not need adjuvant chemotherapy, despite poor traditional predictive factors and vice versa. An improvement in selection of patients could avoid both over- and undertreatment and therefore reduce the risk for complications and improve survival.

The 70-gene signature is now being prospectively validated in the Microarray In Node-negative and 1 to 3 positive lymph node Disease may Avoid ChemoTherapy (MINDACT) trial to investigate its clinical relevance, its performance compared to traditional predictive factors and its ability to predict response to adjuvant systemic treatments. It is expected that 10 to 20% of patients can be safely spared adjuvant chemotherapy by using the 70-gene signature, while it was indicated based on the traditional predictive factors.

***Adjuvant radiation therapy*:** Radiation therapy following lumpectomy may also contribute to the elimination of possible nodal metastases left in situ. A systemic review of van Wely et al. showed that breast irradiation in the context of breast conserving therapy is associated with a significantly lower regional recurrence rate after a negative SLN procedure [46]. A possible explanation is that the lower part of the axilla receives a nearly therapeutic dose in standard tangential breast irradiation. Three- dimensional computed tomography planning has mostly replaced the conventional two-dimensional planning, resulting in more accurate irradiation of the targeted volume. However, still 55% of level I and II of the axilla receive 95% of the prescribed dose by whole breast irradiation [47]. Furthermore it is found that the location of the SLN is radiated by standard tangential radiotherapy in 78-94% of the patients in 2D-radiotherapy [48, 49], and in 76% when using a 3D radiotherapy technique [personal data].

**Nodal tumour load**

As previously mentioned, the SLN is negative in 74% of clinically node negative patients in the general breast cancer population. This percentage is lower in breast cancer patients with small primary breast tumours and if treated with breast conserving therapy. Patients treated with breast conserving therapy are significantly more often diagnosed with pathologic node negative disease or with micrometastatic disease, compared to patients treated with mastectomy (p <0.0001) [50]. The addition of axillary ultrasound to the preoperative nodal staging will increase the percentage of SLN negative patients to 84% in cT1-2N0 breast cancer patients treated with breast conserving therapy [51].

**Micrometastases in the sentinel node**

In the above mentioned 84% SLN negative patients, micrometastatic disease is considered as node- positive. The presence of micrometastasis in the SLN however, has no significant impact on overall survival and it is therefore questionable whether these patients benefit from adjuvant systemic therapy [50, 52, 53]. Other analyses have shown that small occult lymph node metastases (mostly isolated tumour cells or micrometastases) in patients with sentinel nodes that were negative on initial examination, were not associated with worse survival [54, 55]. Routine immunohistochemical examination and serial sections of haematoxylin-eosin-negative SLN’s are therefore no longer standard procedure in many clinics worldwide. Exclusion of these techniques in the Netherlands as well, would increase the number of SLN negative patients even further. In the study of Weaver et al., 4.4% of occult metastases were micrometastases (11.1% isolated tumour cells; 0.4% macrometastases) [54]. About 20-

25% of node positive patients would therefore be counted as node negative when excluding routine immunohistochemical examination and serial sectioning, resulting in an increased percentage of SLN negative patients with 4% to 88%.

**Nodal tumour load in our proposed study population**

Combining the above described results leads to the following percentage of node negative patients and leads to the following nodal tumour load in (occult) node positive patients in our proposed study population:


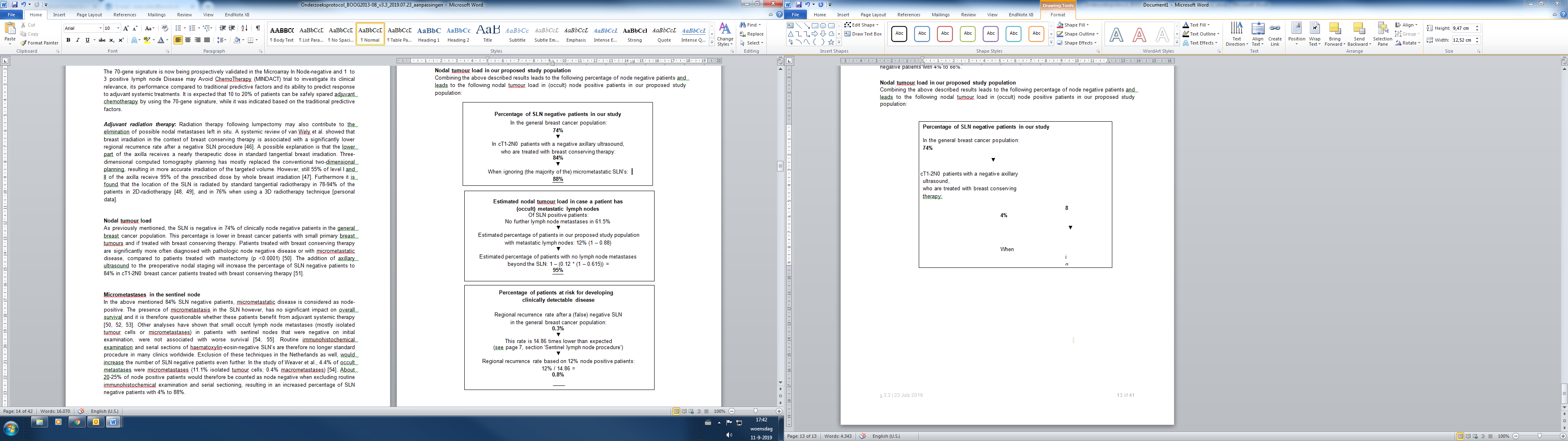


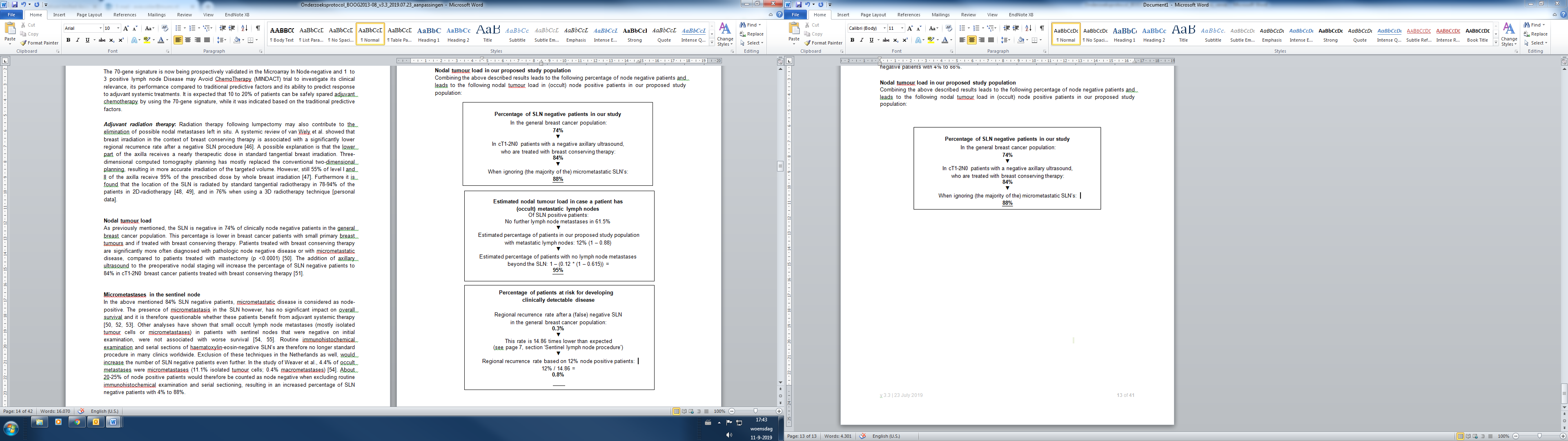


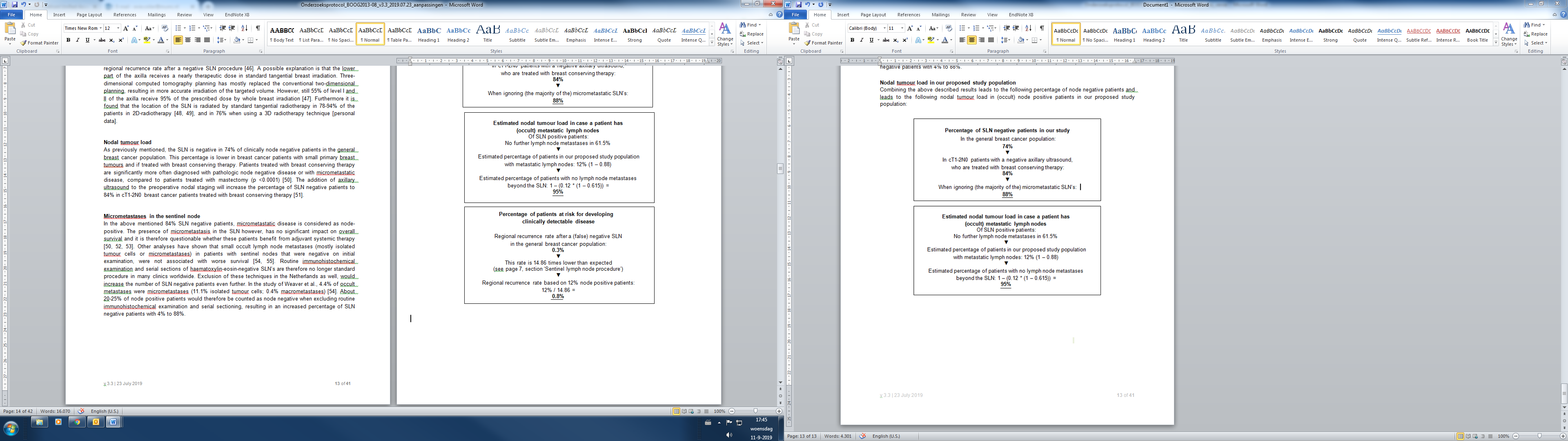


**Conclusion**

In conclusion, the NSABP B-04 randomized controlled trial showed us that omitting primary axillary
treatment of occult positive lymph nodes in clinically node negative breast cancer patients does not affect survival, even after 25 years of follow-up and without the use of adjuvant systemic or radiation therapy. The performance of a delayed ALND in case axillary lymph nodes become clinically positive does not affect survival and prevents axillary overtreatment in the majority of patients. The ACOSOG Z0011 and the IBCSG 23-01 randomized controlled trials revealed that completion ALND can be safely omitted in clinically node negative breast cancer patients with metastatic sentinel nodes. Patients randomized for watchful waiting were likely to have residual nodal disease (13-27%) that was not surgically removed. Nevertheless, survival rates were not affected and regional recurrence rates low. Clinically node negative patients in these trials were selected by physical examination. Adding axillary ultrasound will improve the preoperative selection of node negative patients, as it selects patients with a more favourable tumour load and accurately excludes advanced nodal disease (≥4 metastatic nodes). Biology, adjuvant systemic therapy and standard tangential breast irradiation are all factors that most likely diminish the risk that possible metastases left in situ develop into clinically detectable lymph nodes. Patients treated with breast conserving therapy are more likely to have pathologic node negative or micrometastatic disease, compared to patients treated with mastectomy. The risk for occult (macro)metastases in our study population is low: about 88% will be node negative and about 95% of node positive patients will have no lymph node metastases beyond the SLN. Reflected by the low regional recurrence rate after a (false) negative SLN, only a small amount of patients (0.8%) with node positive disease is expected to develop clinically detectable nodal disease.

**The study**

Therefore, we propose a randomized controlled trial to prove that the sentinel lymph node procedure can be omitted in clinically node negative breast cancer patients treated with breast conserving therapy. This study will significantly decrease the number of breast cancer patients receiving overtreatment of the axilla, thereby diminishing the risk for complications, while maintaining excellent regional control and not affecting survival, with an expected decrease of the annual breast cancer care expenditures.

**4. OBJECTIVES**

**Primary objective**

The primary objective of this study is to investigate whether omitting the sentinel lymph node procedure is not inferior to the current axillary staging regimen in clinically node negative breast cancer patients undergoing breast conserving therapy, in terms of regional recurrence rate.

**Secondary objectives**

Secondary objectives of this study are to investigate whether omitting the sentinel lymph node procedure is not inferior to the current axillary staging regimen in clinically node negative breast cancer patients undergoing breast conserving therapy, in terms of distant-disease free survival, overall survival rate, the local recurrence rate and the occurrence of contralateral breast cancer.

Other secondary objectives are the influence of omitting the sentinel lymph node procedure on the number of delayed axillary lymph node dissections, the administration of adjuvant systemic therapy, the axillary morbidity rate, quality of life and cost-effectiveness.

All objectives are measured during a follow-up of 10 years, and analyzed after 1, 3^1^, 5 and 10 years of follow-up.

**Hypothesis**

The sentinel lymph node procedure can be safely omitted in clinically node negative breast cancer patients undergoing breast conserving therapy. This will lead to a decreased axillary morbidity rate, with non-inferior regional control, distant-disease free survival and overall survival.

**5. STUDY DESIGN**

***a. Description of the study design***

A prospective non-inferiority randomized controlled multicentre trial, randomizing women aged 18 years

or older with pathologically confirmed unilateral cT1-2N0* invasive breast cancer about to undergo breast conserving surgery followed by radiotherapy of the breast to:

Arm A - control arm: lumpectomy with sentinel lymph node procedure, followed by radiotherapy of the breast with or without completion axillary treatment according to the Dutch breast cancer guideline.

Arm B - study arm: lumpectomy without further axillary staging, followed by radiotherapy of the breast.

* cT1-2N0: primary tumour not larger than 5 cm and clinically node negative based on a negative axillary ultrasound (or negative cyto-/histology).

*Stratification for the randomization arm*

Patients will be stratified by: age (≤50, 50≤75, >75 years old), oestrogen receptor status (positive vs. negative), HER2neu status (amplified vs. not amplified), clinical tumour stage prior to any treatment (<3 cm vs. ≥3 cm), grading (grade I-II vs. III - according to Bloom-Richardson grading system), will be or is treated with primary systemic therapy (yes vs. no), participating centre.

***b. Schematic study design***

Study design 1: if primary treated with breast conserving therapy


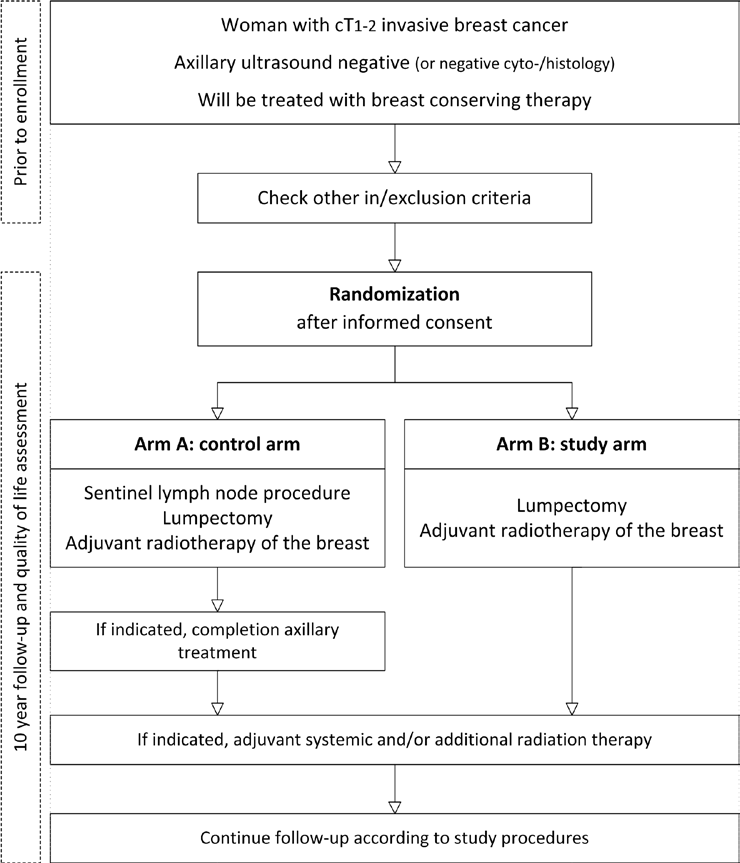


Study design 2: if primary with systemic therapy:


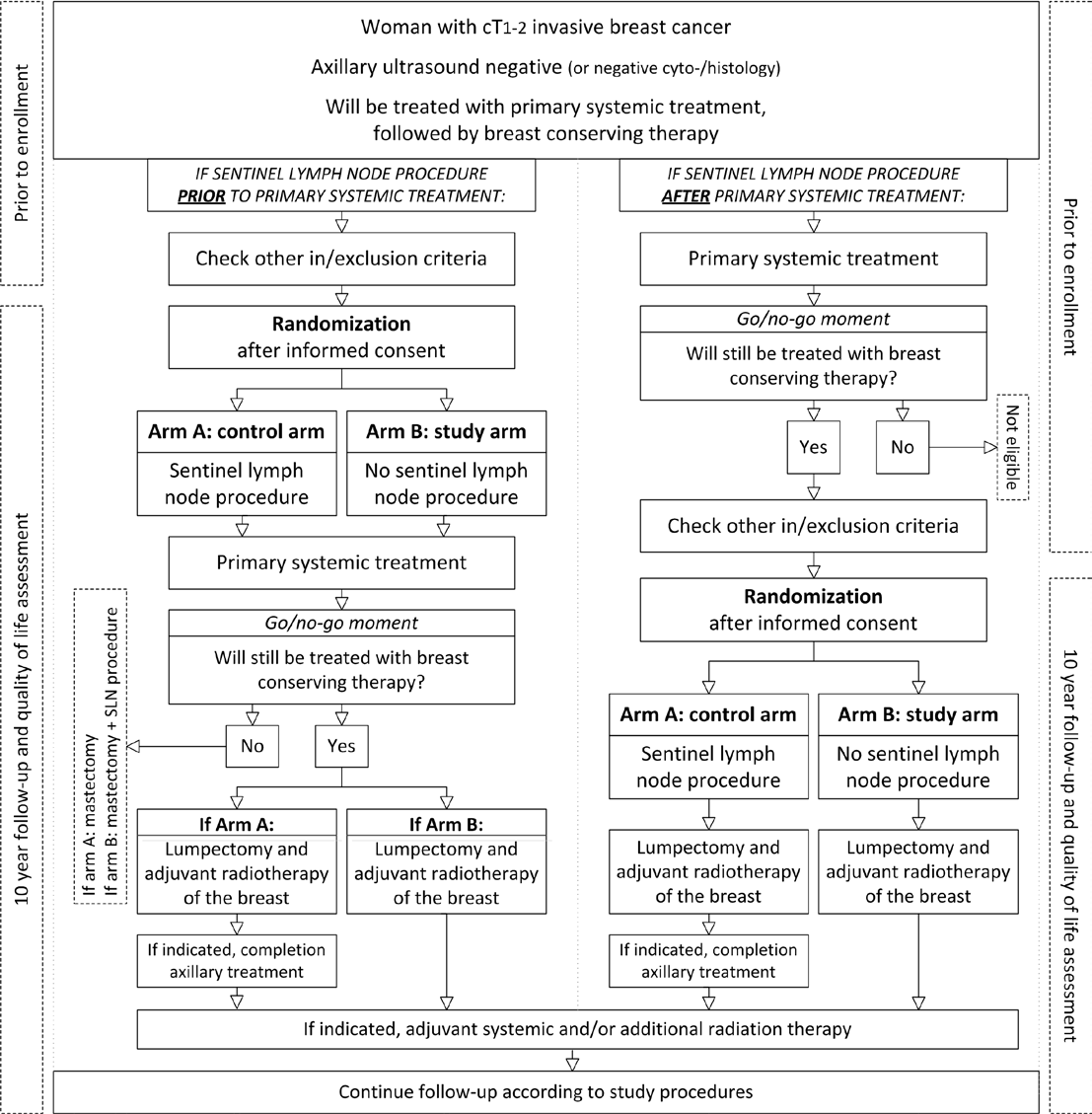


***c. Timeline***

**Jul 2014 - Jan 2015** review of the study

**May2015 - Apr 2021** Inclusion of patients (1644; 36 months)

Start of 10 year follow-up

**Apr 2021 - Apr 2026** Completion of 5 year follow-up of every patient

**Apr 2026 - Apr 2031** Completion of 10 year follow-up of every patient

**6. STUDY POPULATION**

***a. Population***

The incidence of invasive breast cancer in female patients in the Netherlands was approximately

14.000 in 2011.

***b. Inclusion criteria***

In order to be eligible to participate in this study, a subject must meet all the following criteria:

1. Female
2. Aged 18 years or older
3. Pathologically confirmed invasive unilateral breast carcinoma
4. A clinical T1-2 tumour
5. Will be treated with lumpectomy and whole breast radiotherapy
6. Clinically node negative status: no signs of axillary lymph node metastases at physical examination and preoperative axillary ultrasound (or negative cyto-/histopathology)
7. Written informed consent

Furthermore, primary systemic therapy is allowed if the patient has a clinical T1-2N0 status (initial stage) that is amenable to lumpectomy pre-systemic therapy.

***c. Exclusion criteria***

A potential subject who meets any of the following criteria will be excluded from participating in this

study:

1. Clinically node positive pre-operative
2. Bilateral breast cancer
3. Evidence of metastatic disease
4. History of invasive breast cancer
5. Previous treatment of the ipsilateral axilla with surgery or radiotherapy, except surgery for

hidradenitis suppurativa or for other superficially located skin lesions, such as naevi

1. Pregnant or nursing
2. Other prior malignancies within the past 5 years, except successfully treated malignancies that occurred more than five years before randomization, and except successfully treated basal
   cell and squamous cell skin cancer, carcinoma in situ of the cervix or carcinoma in situ of the
   ipsilateral or contralateral breast
3. Unable or unwilling to give informed consent

***d. Sample size calculation***

Sample size calculation for a non-inferiority design including clinically node negative breast cancer

patients treated with breast conserving therapy, who will be randomized between SLN procedure versus no further axillary staging is as follows: Our hypothesis is that clinically node negative patients treated with breast conserving therapy who do not undergo the SLN procedure will have a non-inferior regional recurrence rate compared to patients in the control group and should therefore be treated as such.

Prior data indicate a 5-year regional recurrence free survival rate of 99% for the control patient group. The expected true regional recurrence free survival rate for the experimental patient group is 96% (taking into consideration the higher morbidity rate caused by axillary treatment in the control arm), and a difference of more than 5% in favour of the control patient group (the upper limit of a one-sided 95% confidence interval) is not accepted. The expected regional recurrence free survival rates and the non- inferiority limit of 5% (delta) result in a sample size of 747 per arm, to be able to reject the null hypothesis that the rate for experimental and control subjects is inferior by at least 5%, with a probability of 0.8.

|  | **p1** | **p2** | **Alpha  (type I error)** | **Power** | **Epsilon (p1-p2)** | **delta** | **Sample Size**  **(per arm)** |
| --- | --- | --- | --- | --- | --- | --- | --- |
| **Non-Inferiority** | 0,96 | 0,99 | 0,05 | 0,8 | -0,03 | -0,05 | 747 |

When taking into account a lost to follow-up rate of 10%, **1644** patients need to be randomized. In the Netherlands, every year **14.000** female patients are diagnosed with invasive breast cancer. About 80 % is operated on primarily (when excluding patients treated with systemic therapy only, or patients with primary metastatic disease and frail elderly).

| 14.000 * 80% = **11.200** patients  Of these patients, 80% is cN0.  11.200 * 80% = **8.960** patients  In the Netherlands 54% of the patients are treated with breast conserving therapy and 46% undergo  mastectomy.  8.960 * 54% = **4.838** patients  With the now known interest in participating of 43 hospitals in the Netherlands, 59% of the patients will visit a study hospital.  4.838 * 59% = **2.854** patients  We expect an accrual rate of 30%  2.854 * 30% = 856 patients yearly  This means that **3 years** will suffice to include **1644 patients (including 10% lost to follow-up)** |
| --- |
|  |
|  |
|  |

**7. METHODS**

1. ***Study endpoints***

**Main study endpoint**

⮚ *Regional recurrence rate*

Regional recurrence is defined as tumour recurrence and as residual tumour that became clinically

apparent in ipsilateral axillary, infraclavicular and supraclavicular lymph nodes (pathologically proven).

**Secondary study endpoints**

⮚ *Regional recurrence free survival*

Regional recurrence free survival is defined as the time interval between the date of randomization and the date of first suspicion of regional recurrence or date of death, whichever comes first, measured in days. Patients who did not experience regional recurrence and are still alive are censored at the date of last follow-up.

⮚ *Delayed axillary treatment*

This is defined as the number of patients in in whom delayed axillary treatment (any kind) >6 months

after randomization was performed in case of regional recurrence, with the time interval between the date of randomization and the date of the delayed ALND, measured in days.

⮚ *Distant-disease free survival*

Distant-disease free survival is defined as the time interval between the date of randomization and the

date of first suspicion of distant recurrence or death, whichever comes first, measured in days. Patients in whom a distant recurrence was not observed and are still alive are censored at the date of last follow- up.

Included are: distant recurrence (distant metastasis), death from breast cancer and its treatment, death from second primary invasive (nonbreast) cancer death from unknown cause. N.B.: lymph node recurrence in cervical lymph nodes or in contralateral internal mammary lymph nodes or in the contralateral axilla is coded as distant metastasis.

Excluded are: ipsilateral breast tumour recurrence, regional invasive recurrence, contralateral breast cancer, and all in situ carcinomas, as these events are potentially nonlethal.

⮚ *Overall survival*

Overall survival is defined as the time interval between the date of randomization until death from any

cause. Patients who are still alive are censored at the date of last follow up.

Included are: death from breast cancer, death from nonbreast invasive cancer cause, death from unknown cause.

⮚ *Local recurrence rate*

Local recurrence is defined as any pathologically proven invasive in-breast recurrence in the ipsilateral

breast after breast surgery. The local recurrence free interval is defined as the time interval between the date of randomization and the date of first clinical suspicion of local recurrence, measured in days.

⮚ *Other-regional recurrence rate*

Other-regional recurrence is defined as pathologically proven recurrence in the region of the ipsilateral

internal mammary lymph node chain (lymph nodes in the intercostal spaces along the edge of the sternum in the endothoracic fascia). Other-regional recurrence free interval is defined as the time interval between the date of randomization and the date of first clinical suspicion of other regional recurrence, measured in days.

⮚ *Contralateral breast cancer rate*

The occurrence of (pathologically proven) contralateral invasive breast cancer will be registered. Contralateral breast cancer free interval is defined as the time interval between the date of randomization and the date of first clinical suspicion of contralateral breast cancer, measured in days.

⮚ *Diagnosis of recurrence outside the axillary region*

One or more of the following must be positive for the diagnosis of tumour recurrence to be accepted:
I. Histology or cytology

II. Autopsy examination

Diagnosis based on radiological findings can be considered if I or II is not possible/available (these
cases will be presented to the Data Safety Monitoring Board (DSMB) for an independent review).

⮚ *Percentage difference in the administration of (neo)adjuvant systemic therapy*

This is defined as the percentage difference in the administration of (neo)adjuvant systemic therapy

between both study arms.

- *Quality of Life*

Quality of Life will be assessed using validated questionnaires, as shown in chapter 7 ‘METHODS’, section e ‘Study procedures - axillary morbidity rate and quality of life’ on page 27.

⮚ *Axillary morbidity rate*

The axillary morbidity rate will be assessed using validated questionnaires, as shown in chapter 7

‘METHODS’, section e ‘Study procedures - axillary morbidity rate and quality of life’ on page 27.
⮚ *Cost-effectiveness*

The cost-effectiveness will be assessed by using patient questionnaires, as shown in chapter 7 ‘METHODS’, section f ‘Cost-effectiveness analysis’ on page 28 and electronic Case Report Forms (eCRF) as shown in chapter 10 ADMINISTRATIVE ASPECTS AND PUBLICATION on page 34.

1. ***Study procedure – diagnosis and randomization***

*Diagnosis*

The study population will be selected from the group of patients visiting the hospital with breast complaints or patients referred by the breast cancer screening program. Eligibility for the study should be verified in every patient with invasive breast cancer. According to the standard care and work-up of breast cancer patients, general patient information and medical history will be obtained, and all patients will undergo standard preoperative physical and radiological examination (which may include mammography, ultrasound of the breast and axillary region, MRI of the breast, cytological and/or histological biopsies) according to the Dutch breast cancer guideline. Screening for axillary lymph node metastases with physical examination and an axillary ultrasound is obligatory.

*Axillary ultrasound*

The axillary ultrasound is standard of care in the preoperative diagnostic work-up of breast cancer patients according to the Dutch breast cancer guideline. The performance of the axillary ultrasound is standardized in this study to ensure its quality. Therefore the radiologist should perform the axillary ultrasound according to the following method:

Preferably, the patient is positioned with the ipsilateral hand placed behind the head. The entire axilla is examined in a standardized fashion, starting at the low axilla (level I: inferior and lateral to the pectoralis minor muscle), and continuing upwards toward mid-axilla (level II: medial and lateral to the pectoralis minor muscle and interpectoral) and apical axilla (level III: superior and medial to the pectoralis minor muscle with apical lymph nodes).

The following criteria are used during ultrasound to identify positive lymph nodes: long to short axis ratio of <2 (i.e. round), diffuse or focal cortical thickening, effacement or replacement of the fatty hilum, and/or nonhilar blood flow (using Doppler ultrasound, if detectable). As described in the Dutch breast cancer guideline, cortical thickening of more than 2.3 mm is considered as the optimal cut-off point to perform fine-needle aspiration biopsy. Additionally, a subjective assessment of thickening can be made by the radiologist during real-time imaging, similar to the studies by Koelliker et al, Abe et al, and Neal et al [37, 56, 57]. Fine-needle aspiration biopsy or core biopsy is recommended when suspicious lymph nodes are identified. Preferably, a core biopsy of a suspicious lymph node is performed instead of fine-needle aspiration biopsy, because core biopsy is more sensitive, as described by Rautiainen et al. [35]. In case of two or more abnormal lymph nodes, the lymph node with the most suspicious findings is selected for tissue sampling.

*Randomization*

In case a patient meets the inclusion criteria and breast conserving therapy is the choice of treatment (according the considerations of the surgeon and the patients’ preference), the patient will be asked to participate in the study by the attending surgeon. We will start the inclusion of patients immediately after the approval of the METC and the Executive Board.

⮚ *Written informed consent in patients undergoing primary surgical treatment*

This section is applicable in cases undergoing primary surgical treatment. Before the surgical treatment,

patients are informed about the goal, the randomization procedure and the consequences of participating. Written informed consent must be obtained and randomization performed preoperatively (lumpectomy ± SLN procedure). Randomization is performed centrally.

⮚ *Written informed consent in patients undergoing primary systemic treatment*

This section is applicable in cases undergoing primary systemic treatment. Primary systemic therapy is

allowed if the patient has a clinical T1-2 tumour (initial stage) that is amenable to lumpectomy pre- systemic therapy.

In case the SLN procedure is standardly performed *prior* to the primary systemic therapy, written informed consent must be obtained and randomization performed prior to the SLN procedure and primary systemic treatment. Subsequently, the SLN procedure is or is not performed according to the randomization arm, followed by the primary systemic treatment. If patients are not treated with breast conserving therapy after completion of the primary systemic treatment (for any reason) and are treated with a mastectomy, the SLN procedure should be performed for patients randomized to arm B.

In case the SLN procedure is standardly performed *after* the primary systemic therapy, written informed consent must be obtained and randomization performed after completion of the primary systemic treatment when the final decision is made that the patient is treated with breast conserving therapy.
Randomization is performed centrally.

⮚ *Stratification*

Patients will be stratified by: age (≤50, 50≤75, >75 years old), oestrogen receptor status (positive vs.

negative vs. unknown), HER2neu status (amplified vs. not amplified vs. unknown), clinical tumour stage prior to any treatment (<3 cm vs. ≥3 cm), grading (grade I-II vs. III - according to Bloom-Richardson grading system vs. unknown), will be or is treated with primary systemic therapy (yes vs. no), participating centre.

***c. Study procedures - treatment***

*Randomization arm A: lumpectomy with sentinel lymph node procedure, followed by radiotherapy of the*

*breast*

Patients randomized for control arm A will be treated with lumpectomy and the SLN procedure, followed by radiotherapy of the breast. In case a (non-)SLN metastasis is detected, completion axillary treatment is performed if indicated by the Dutch breast cancer guideline; i.e. either no completion axillary treatment, or completion axillary lymph node dissection, or radiation therapy of the axilla +/- periclavicular lymph nodes. During follow-up the axilla will be assessed by physical examination. In case of the suspicion of an axillary lymph node metastasis with physical examination, an axillary ultrasound (+/- tissue sampling) is indicated. When a metastasis is cyto- or histopathologically confirmed, accurate staging for distant metastases is performed according to the Dutch breast cancer guideline. A delayed ALND is performed if indicated by the multidisciplinary team.

*Randomization arm B: lumpectomy without further axillary staging, followed by radiotherapy of the breast*

Patients randomized for study arm B will be treated with lumpectomy followed by radiotherapy of the breast. These patients will not undergo the SLN procedure. During follow-up the axilla will be accurately assessed by physical examination. In case of the suspicion of an axillary lymph node metastasis at physical examination, an axillary ultrasound (+/- tissue sampling) is indicated. When metastasis is cyto- or histopathologically confirmed, accurate staging for distant metastases is performed. A delayed ALND is performed if indicated by the multidisciplinary team.

*Sentinel lymph node procedure*

The SLN procedures will be performed using technetium-99m Nanocolloid as a radioactive tracer and blue dye / patent blue for lymphatic mapping. Both are injected into breast parenchymal tissue surrounding the tumour, biopsy cavity or periareolar. The tracer will search its way through draining lymph vessels to the first receiving lymph node. This SLN will be identified and harvested by use of the following triple technique: lymphoscintigraphy, intraoperative use of the gamma probe, and intraoperative detection of the blue lymphatic vessels. Palpation of the axilla through the incision after removal of tracer-labelled SLN’s should be performed to identify and remove suspicious (non-SLN) nodes.

⮚ *Pathological assessment - the sentinel node*

As a minimal requirement three histological levels (500 micron distance) for each SLN are examined.

On each level two parallel sections are performed, one for immunohistochemistry and one for haematoxylin and eosin (H&E) staining. Immunohistochemical (IHC) staining is performed for markers containing at least cytokeratin 8 and 18 (e.g. CAM 5.2, NCL5D3). Cytokeratin IHC staining is performed only when H&E staining is negative. Lymph nodes submitted for pathological examination which are marked by the surgeon as non-sentinel nodes are examined with H&E and if negative with cytokeratin IHC staining.

The exact diameter of every SLN must be determined, as well as describing the occurrence of extranodal growth. Isolated tumour cells (<0.2mm) are considered as SLN negative.

*Local treatment primary tumour - breast conserving therapy*

Breast conserving therapy is defined as lumpectomy followed by whole breast irradiation, according to the Dutch breast cancer guideline. Patients are amenable to undergo breast conserving therapy when a good cosmetic result and an equally good locoregional control can be expected according to physical and radiological findings.

As described in the Dutch breast cancer guideline, re-excision is required if invasive tumour or ductal carcinoma in situ reaches into a large area (>4mm) or multiple small areas in the inked resection margin (non-radical). In case a mastectomy is performed as re-excision, the SLN procedure should be performed at time of the mastectomy for patients initially treated according to arm B. Re-excision is not required if the tumour reaches into a limited area (≤4 mm) in the inked resection margin (focal non- radical). Local control can be achieved by adjusting radiotherapy fields and dose.

To facilitate accurate tumour bed localization for irradiation, MRI-compatible clips are positioned intraoperative around the tumour bed after the lumpectomy, as followed: in the medial, lateral, superior and inferior edge of the tumour bed (between skin and fascia, at the level of the tumour) and one clip is placed posterior on the deepest point (usually the pectoral fascia). Radiation therapy is given according to the guidelines described below.

*Radiation therapy details*

*Dose and fractionation*

A fractionation scheme equivalent to 25 x 2 Gy, 5 fractions per week must be applied. Most Dutch centres use a scheme of 15 x 2.67 Gy or 16 x 2.66 Gy, 5 fractions per week. Possible indications for a boost (dose equivalent to 8 x 2 Gy) to the rim of tissue of 1.5 cm around the primary tumour are: patients 50 years or younger, grade 3, or vascular invasion. In case of focal irradical resection, a higher boost dose to an equivalent to 10-13 x 2 Gy can be considered. A simultaneous integrated boost is recommended, with the high fraction size not exceeding 2.67 Gy.

Partial breast irradiation is not allowed. In case a participating centre is also involved in a study concerning partial breast irradiation, we suggest the following: eligible patients aged ≥49 years will be informed about participation in the trial concerning partial breast irradiation, and eligible patients aged <49 years will be informed about participation in the BOOG 2013-08 trial.

*Delineation*

Delineation or target volumes and organs at risk should be performed using the European Society for Radiotherapy & Oncolology (ESTRO) guidelines of v. Offeresen et al. [58].

Delineation of the tumour bed, including a clinical target volume (CTV) and a planning target volume (PTV) is obligatory, irrespective whether a boost is applied or not. In addition, delineation of the axillary nodal regions, axilla level 1, 2, Rotter and 3 is obligatory, even when there is no indication for axillary radiation. Delineation of the supraclavicular nodes is only required if part of the target volume. Delineation of breast tissue is optional.

In case of left-sided breast cancer, delineation of the heart and lungs is obligatory; delineation of the other normal structures is optional.

*Technique/ dose distribution*

The dose in the target volume (breast +/- axilla +/- periclavicular nodes) must be between 95%-107% of the prescribed dose. The Central Lung Distance (CLD) must be < 3 cm (in case of tangential fields) and/or the mean lung dose < 7.5 Gy; the Maximum Heart Distance (in case of tangential fields) must be < 1 cm, and the heart volume receiving > 10 Gy should be < 15%. If lung or heart constraints cannot be met, some underdose in the breast can be accepted to reach the constraints, provided that the PTV of the tumour bed is adequately covered. Breath holding techniques to reduce heart dose in left sided breast cancer are highly recommended.

For evaluation purposes: the minimum, maximum and mean dose of the axilla level 1, 2, Rotter, and 3 should always be recorded.

*Pathological assessment - the primary tumour*

The pathological size and surgical margin (tumour-free margin) of the primary tumour must be assessed. The hormone receptor status must be determined by immunohistochemical staining and is considered positive if ≥10% of the cells stain positive. HER2-status is determined by immunohistochemistry and in case of 2+, by CISH or FISH. Histological tumour grading will be assessed according to the Bloom- Richardson grading system. The histological tumour type must be defined according to the World Health Organization. Presence of lymphovascular invasion is defined as one or more tumour cells in a lymphatic or vascular structure.

⮚ *Modified Bloom-Richardson grading system*

The grading system consists of three components of the tumour morphology: the extent of tubule

formation, the nuclear polymorphism and mitotic activity defined as the number of mitoses per 2 mm^2^. A score of 1, 2 of 3 is assigned to each of these components. The histological grade is determined by the sum of these scores.

Level of tubule formation: 1 = > 75%

2 = 10-75%

3 = < 10%

Nuclear polymorphism: 1 = comparable to normal epithelium

2 = enlarged, vesicular, small nucleoli

3 = polymorphic, vesicular, large nucleoli

Mitotic activity: 1 = 0 through to 7 mitoses per 2 mm^2^

2 = 8 through 12 mitoses per 2 mm^2^

3 = 13 or more mitoses per 2 mm^2^

The histological grade is I for the scores 3-5, II for 6-7, and III for 8-9.

*Primary or adjuvant systemic therapy*

The administration of systemic therapy is not mandatory in this trial. The indication for adjuvant systemic therapy is determined according to the current Dutch breast cancer guideline and multidisciplinary approach. Adjuvant! Online can also be used to estimate the 10-years breast cancer specific survival and the risk reduction by systemic therapy, with or without knowledge of the pathological nodal status. Validated gene expression profiling can be used as an addition to clinicopathologic characteristics, in case of doubt about the indication for adjuvant systemic therapy based on the traditional prognostic factors.

Primary systemic therapy is allowed, but study patients must have a clinical T1-2 status amenable to breast conserving surgery pre-systemic therapy. In patients in study arm B in whom breast conserving surgery is no longer feasible after primary systemic therapy, the SLN procedure will be performed afterwards.

***d. Study procedures - follow-up***

After inclusion a follow-up period of 10 years follows:

Year 1 - 5: physical examination of the axilla and a mammography once yearly

Year 6 - 10: physical examination of the axilla once yearly

≤ 60 years: mammography once yearly

> 60 years: mammography once every two years

Additional imaging techniques for detecting possible recurrence/metastasis will only be performed on indication.

⮚ An axillary ultrasound is performed in patients with a clinical suspicion (physical examination)
of axillary lymph node metastases during follow-up. If an axillary lymph node metastasis is
confirmed (cyto-/histology), staging for distant metastatic disease is mostly performed according
to the Dutch breast cancer guideline.

⮚ Staging for metastatic disease is mostly performed (according to the Dutch breast cancer
guideline) in patients with a clinical suspicion of distant metastatic disease during follow-up.
Physical examination of the axilla must then always be performed for the detection of possible
axillary lymph node metastases. An axillary ultrasound is only performed in patients with a
clinical suspicion (physical examination) of axillary lymph node metastases.

**Table 1. Timeline BOOG 20913-08 study**

|  |  |  | **Year** | | | | | | | | | |
| --- | --- | --- | --- | --- | --- | --- | --- | --- | --- | --- | --- | --- |
| **Required Investigations** | **Prestudy** | **0.5** | **1** | **2** | **3** | **4** | **5** | **6** | **7** | **8** | **9** | **10** |
| Mammography | X |  | Once yearly | | | | | ≤ 60 years: once yearly  ≥ 60 years: once in two years | | | | |
| FNA-core biopsy ¨rimairy tumour | X |  |  |  |  |  |  |  |  |  |  |  |
| Receptor status (ER, PR, HER2, if known) | X |  |  |  |  |  |  |  |  |  |  |  |
| Physical examination axilla | X | Once yearly – outpatient clinic | | | | | | | | | | |
| Axillary ultrasound | X | On indication | | | | | | | | | | |
| Staging for distant metastatic disease |  | On indication | | | | | | | | | | |
| QoL questionnaires* | X | X | X | X | X |  | X |  |  |  |  | X |
| Questionnaire Health care utilization and costs patients* | X | X | X | X | X |  |  |  |  |  |  |  |

* = until a total of 1056 patients is reached

***e. Study procedures - axillary morbidity rate and quality of life***

**Introduction**

Omitting the SLN procedure in clinically node negative breast cancer patients undergoing breast conserving therapy might positively influence the axillary morbidity rate and quality of life. Although the SLN procedure is less invasive compared to an axillary lymph node dissection, complications do not rarely occur; most reported are seroma, hematoma, anaphylactic reaction, lymphoedema, numbness, tingling and pain [12-14]. Severe lymphoedema occurs in still 8% of patients undergoing only this axillary procedure after a follow-up of only 3 years [16]. These findings however are influenced by other therapies, such as the breast surgery itself, breast irradiation or adjuvant systemic therapy. Patients in this study are randomized to undergo either the SLN procedure (either with or without completion axillary treatment) or no SLN procedure. All patients will undergo the same breast treatment (lumpectomy followed by radiation of the breast). The SLN procedure is omitted in the experimental study group. Our study will therefore be the first to provide precise information on the influence of the SLN procedure on the axillary morbidity rate and quality of life.

Further, not performing the sentinel lymph node procedure and thereby possibly leaving supposed tumour load behind, could negatively influence patient’s well-being, with more insecurity and anxiety towards their future. Though, a study showed that trait anxiety rather than the diagnosis breast cancer determined whether patients experienced a low quality of life [59].

**Patient selection**

All patients eligible for the study will be asked to complete the questionnaires once pre-operative and on regular basis post-operative, as shown in the timeline, until a total of 1056 patients eligible for evaluation is reached, equally divided over both treatment arms. Patients are eligible for evaluation only when at least two following questionnaires are completed, of which a completed preoperative questionnaire is mandatory. No patient selection will be performed in order to accomplish a representative study population in accordance to the general breast cancer population.

**Organisation**

The surgeons of the participating centers are responsible for inclusion of patients and handing out the first (preoperative) questionnaire. IKNL Clinical Trial Centre is responsible for the transmission of the questionnaires during follow-up to all included patients. Addresses are collected on the informed consent form, on which patients sign the informed consent, as well as if they do or do not agree that their address can be collected from their general practitioner after 10 years of follow-up, in order to prevent lost to follow-up due to migration. Patients can return the completed questionnaires in self-addressed envelopes to study coordinator M.L. Smidt, Maastricht University Medical Centre+.

**Methods**

To assess the Quality of Life of breast cancer patients in this study, we will use the Dutch version of two validated Quality of Life questionnaires of the EORTC, QLQ-C30 and QLQ-BR 23 (developed specifically for the use in breast cancer patients) and the Dutch version of the EQ-5D-5L [60, 61]. To assess the subjective morbidity we will use the validated “Lymph oedema Functioning, Disability and Health” (Lymph-ICF) questionnaire, which assesses the impairments in function, activity limitations and participation restrictions of patients with arm lymph oedema [62]. A validated short Dutch version of the Spielberger State-Trait Anxiety Inventory (STAI-trait) and the NEO Five Factor Inventory (NEO-FFI) is used to measure if anxiety and personality traits influence the outcome of Quality of Life [63]. These questionnaires are provided pre-randomization and sequentially post-randomization at 6 months 1, 2, 3, 5 and 10 years. The questionnaires will be sent out to up and including patient 1056.

**Informed consent for quality of life**

All patients included in QoL study will be informed about the aims and procedure of the study. They will be informed about the strict confidentiality of their patient data. Axillary morbidity and quality of life assessment is part of the study procedures, therefore written informed consent is obtained simultaneously with the informed consent of the other study procedures, as described previously. The informed consent form is part of the documents to be submitted to the METC for approval. It is the responsibility of the METC to guarantee that this form is conform ICH-GCP guidelines.

It will be emphasized that the participation is voluntary and that the patient is allowed to refuse further participation in the protocol whenever she wants. This will not prejudice the patient’s subsequent care. Documented informed consent must be obtained for all patients included in the study before they are randomized in the study. The written informed consent form should be signed and personally dated by the patient.

***f. Cost-effectiveness analysis***

**Introduction**

In order to identify the cost-effectiveness of omitting the sentinel node biopsy for clinically T1-2N0 breast cancer patients undergoing breast conserving therapy, a Cost-Effectiveness Analysis (CEA) will be conducted. The CEA will take both a societal and health care perspective and will be expressed as incremental societal costs per Quality Adjusted Life Years (QALY) and healthcare costs per case regional recurrence and case of lymphedema. The CEA will include 474 patients.

The inclusion percentage of the cost-effectiveness study is 66%. At least 474 patients needed to be included for this sub-study. Since the quality of life research is of great importance for estimating QALYs, the quality of life- questionnaire is extended until the inclusion of the CEA –study is completed. With an inclusion rate of 66%, 1056 patients should be included (explanation: 1056 needed for the study, now 951 already included).

**Data-collection and analysis**

The cost-analysis will be performed according to the micro-costing method, whereby a detailed and complete analysis of the resource use and costs of each patient up to 3 years after inclusion will be performed. Costs will be calculated by multiplying resource use with the prices per unit of resource.

Volumes of use will be collected by means of the electronic Case Report Forms (eCRF) as shown in chapter 10 ”ADMINISTRATIVE ASPECTS AND PUBLICATION”, section a “handling and storage of data’ on page 35’ Resource use outside the hospital such as general practitioner costs and costs due to effect on work participation/productivity losses will be collected by means of a standardized retrospective cost questionnaire with a recall period of 3 months, which will be administered on the same

moments as the quality of life questionnaires. Guideline prices will be used [64, 65]. Otherwise, integral cost prices will be obtained from the Maastricht University Medical Centre, or cost-price calculations will be performed. Costs and effects will be discounted according to Dutch pharmaco-economic guidelines. Standard sensitivity analyses and bootstrap analysis will be performed to investigate the uncertainty surrounding the cost-effectiveness ratios [66]. Based on the bootstrap results, cost-effectiveness acceptability curves will be constructed, showing for a wide range of cost-effectiveness threshold values, the probability that omission of the SLNB is cost-effective. Additionally, a decision analytical model will be developed in which the costs and benefits will extend to a lifetime period. The lifetime course of the cohort will be modelled with a Markov model. The model will contain health states like “disease-free”, “regional recurrence”, “lymphedema”, “death from cancer”, “death from other causes” and possible combinations.

***g. Budget impact analysis***

A budget-impact analysis (BIA) will be performed according to the ISPOR guidelines [67]. The BIA addresses the financial stream of consequences related to omitting of the SLNB. The budget impact will depend on the savings due to omission of the SLNB and its consequences (as calculated in the cost- analysis), as well as the uptake of this new strategy by surgical oncologists. Analyses will be focused from the perspective of the national government and the health care insurers over a 5-year time horizon. In addition, the budget impact will be calculated from a broad societal perspective and from the perspective of the “budgettair kader zorg (BKZ)”. Different scenarios will be the scenarios in which SLNB is omitted in 25%, 50%, 75% and 100% of the Dutch hospitals. Furthermore, scenarios will be modelled in which the timeline of de-implementation of SLNB in 100% of the hospitals is varied between direct implementation to implementation in 5 years The BIA will be performed alongside the cost effectiveness analysis.

***h. Withdrawal of individual subjects***

Patients can leave the study at any time for any reason if they wish to do so without any consequences,

and without having to specify the reason for withdrawal. The investigator can decide to withdraw a subject from the study for urgent medical reasons. Withdrawal of patients during the follow-up period will be counted to the 10% of lost to follow-up.

***i. Premature termination of the study***

After premature termination of the study, the investigators must contact all participating subjects within

4 weeks. No new patients are included in the trial. Follow-up of participating subjects will continue, following the study protocol. In case of premature termination of the study due to a higher regional recurrence rate than expected as determined by the DSMB, this will not have a direct consequence for the treatment or follow-up of every study patient that has been randomized for study arm B. So in these patients, delayed axillary treatment is not standardly performed, but only if nodal metastases become clinically apparent.

***j. Final statistical analysis***

Primary and secondary endpoints will be analysed per protocol and in the intention to treat population

after 5 and 10 years of follow-up. Primarily, uncorrected chi-squared statistics will be used to evaluate the null hypothesis, as described in chapter 6, section d ‘Sample size calculation’ on page 19. The chi- square test will be based on the Kaplan-Meier estimator, in case of censored data. Additionally, cox proportional hazards models and Kaplan Meier estimates will be used to analyse the outcome of both groups and to assess the univariable and multivariable association between prognostic variables, treatment and events, using the stratification factors. All statistical tests are 1-sided and a P value of 0.05 or less is considered statistically significant.

**8. SAFETY REPORTING**

***a. Section 10 WMO event***

In accordance to section 10, subsection 1, of the WMO (In Dutch: Wet Medisch-wetenschappelijk

Onderzoek met mensen), the investigator will inform the subjects and the reviewing accredited METC if anything occurs, on the basis of which it appears that the disadvantages of participation may be significantly greater than was foreseen in the research protocol. The study will be suspended pending further review by the accredited METC, except insofar as suspension would jeopardize the subjects’ health. The investigator will take care that all subjects are kept informed.

***b. Adverse events***

**Adverse events**

Adverse events (AE’s) are defined as any undesirable experience occurring to a subject during the study, whether or not considered related to the protocol treatment. All AE’s reported spontaneously by the subject or observed by the investigator or his staff will be recorded.

**Serious adverse events**

A serious adverse event (SAE) is defined as an untoward medical occurrence or effect related to protocol treatment that results in:

- death;
- hospitalisation or prolongation of existing inpatients hospitalisation;
- surgery.

Protocol treatment is defined as breast conserving surgery of the primary tumour, whole breast radiotherapy, SLN procedure, and completion axillary treatment. Any other operation or adjuvant treatment (such as chemotherapy) is not considered protocol treatment.

Information about SAE’s that occur within 30 days after protocol treatment is collected and recorded on the Serious Adverse Report Form. The principal investigator of the participating centre where the SAE occurs is responsible to report the SAE by e-mail to the central data centre (IKNL Clinical Trial Centre) within 24 hours.

The Principal Investigators (“verrichter” in the terminology of the Dutch law) are responsible for SAE assessment and expedited reporting through the web portal *ToetsingOnline* to the accredited METC that approved the protocol. The expedited reporting will occur not later than 15 days after the sponsor has first knowledge of the adverse reactions. For fatal or life threatening cases the term will be maximal 7 days for a preliminary report with another 8 days for completion of the report.

All SAE’s will be followed until they have abated, or until a stable situation has been reached. Depending on the event, follow-up may require additional tests or medical procedures as indicated and/or referral to the general physician or a medical specialist.

***c. Data Safety Monitoring Board (DSMB)***

An independent DSMB will be established comprising an independent surgeon, medical oncologist,

radiation oncologist, and statistician. The DSMB will meet annually to discuss the occurrence and nature of regional recurrences, other events (described in chapter 7, section 7.a ‘Study endpoints’ on page 21), the occurrence of AE’s, and the percentage difference in administration of adjuvant systemic therapy between both study arms. During the study, the DSMB may decide to change the frequency of discussion. All cases with a lesion that is highly suspicious for tumour recurrence on imaging, but not accessible for histology or cytology are presented to the DSMB for an independent review. An interim analysis is performed by a statistician, and results will be presented to the DSMB for further interpretation. Rules for the interim analysis will be described in the Statistical Analysis Plan. As

described earlier, premature termination of the study will not directly lead to additional axillary treatment of patients in study arm B, but only in case nodal metastases become clinically apparent during follow- up.

Advice(s) of the DSMB are send to the principal investigators. Should the principle investigators decide not to fully implement the advice of the DSMB, the principle investigators will send the advice to the reviewing METC, including a note to substantiate why (part of) the advice of the DSMB will not be followed.

***d. Stopping rule***

The Principle Investigators reserved the right to discontinue the study prior to inclusion of the intended

number of subjects, but intends only to exercise this right for valid scientific or administrative reasons such as; a negative advice for continuing the study by the DSMB; in case of a percentage difference in the administration of adjuvant systemic therapy of more than 5% between both study arms; or disappointing accrual so that the total enrolment of 1644 patients seems not feasible within the planned study period.

**9. ETHICAL CONSIDERATIONS**

***a. Regulation statement***

This study will be conducted in accordance to the standards of Good Clinical Practice, in agreement

with the Declaration of Helsinki and with Dutch law in general and with the Medical Research Involving Human Subjects Act (In Dutch: *Wet Medisch-wetenschappelijk Onderzoek met mensen*) in particular. This protocol will be submitted for central approval to an authorized METC.

***b. Recruitment and consent***

The population being researched will be selected from the group of patients visiting one of the

participating hospitals with breast complaints or after referral from the breast cancer screening program. The diagnostic work-up in these breast cancer patients will be performed according to the Dutch breast cancer guideline. Before they agree to participate in this trial, all patients will be provided with written information in the form of a Patient Information Folder.

All patients will be informed on the aims of the study, the possible adverse events, the procedures and possible hazards to which they will be exposed and the mechanism of treatment allocation. They will be informed that their identity will be protected. A sequential identification number will be allocated to each patient randomized in the study. The number will identify the patient and must be included in all case report forms including SAE forms. Patients must also be informed that their medical records may be reviewed for study purposes by authorized individuals other than their treating physician and their study files and materials are being saved for 15 years as described in the patient information folder.

The informed consent form is part of the documents to be submitted to the METC for approval. It is the responsibility of the METC to guarantee that this form is conform ICH-GCP guidelines. It will be emphasized that the participation is voluntary and that the patient is allowed to refuse further participation in the protocol whenever she wants. This will not prejudice the patient’s subsequent care. Documented informed consent must be obtained for all patients included in the study before they are randomized in the study. The written informed consent form should be signed and personally dated by the patient. The formal written consent of the patient must be obtained before initiation of any study- specific procedure.

***c. Benefits and risk assessment***

All treatment procedures will be performed according to the Dutch breast cancer guideline. No additional

interventions will be performed, only less if randomized for arm B (no further axillary staging). When randomized for arm A (SLN procedure), patients will receive the SLN procedure and treatment according to the Dutch breast cancer guideline.

Possible advantages and disadvantages depend on the treatment arm. Patients randomized to arm B have the possible advantages of less morbidity, less surgery and hospitalizations compared to patients randomized to arm A (SLN procedure). Possible disadvantages for patients randomized to arm B are a worse regional recurrence rate. In case of regional recurrence during follow-up, delayed axillary treatment is performed if indicated.

No patient will encounter any delay in their treatment as a result of inclusion. Patients can leave the study at any time for any reason if they wish to do so without any consequences and without having to specify the reason for withdrawal. The investigator can decide to withdraw a subject from the study for urgent medical reasons.

***d. Compensation for injury***

The Principal Investigators have a liability insurance which is in accordance with article 7, subsection 6

of the WMO. The Principal Investigators and the Local Investigators (also) have an insurance which is in accordance with the legal requirements in the Netherlands (Article 7 WMO and the Measure regarding Compulsory Insurance for Clinical Research in Humans of 23th June 2003). This insurance provides cover for damage to research subjects through injury or death caused by the study.

1. € 450.000,-- (i.e. four hundred and fifty thousand Euro) for death or injury for each subject
   who participates in the Research;
2. € 3.500.000,-- (i.e. three million five hundred thousand Euro) for death or injury for all
   subjects who participate in the Research;
3. € 5.000.000,-- (i.e. five million Euro) for the total damage incurred by the organisation for all
   damage disclosed by scientific research for the Sponsor as ‘verrichter’ in the meaning of
   said Act in each year of insurance coverage.

The insurance applies to the damage that becomes apparent during the study or within 4 years after the end of the study.

**10. ADMINISTRATIVE ASPECTS AND PUBLICATION**

***a. Handling and storage of data***

IKNL Clinical Trial Centre, is responsible for randomization of patients, supply of electronic Case Report

Forms (eCRF), receipt of eCRF pages and generation of queries and SAE processing. Also, IKNL Clinical Trial Centre can give advice or perform audits in participating institutions, when requested by the Principal Investigators.

TRIAS is a web-based clinical data management system for automated and safe registration, administration and information service of patient data for clinical studies. A brochure of the system is attached to the protocol.

IKNL Clinical Trial Centre, is also responsible for the transmission of the questionnaires to all included patients.

The eCRF’s must be completed, dated and signed as soon as the requested information is available. eCRF’s will contain common information, but this information will be kept to a minimum. The time between the patient’s visit and completion of eCRF pages should be kept to a reasonable minimum. The data managers are responsible for the correct completion of the eCRF’s of all study patients.

To enable peer review and/or inspections from Health Authorities, the investigator must agree to keep records, including the identity of all participating subjects (sufficient information to link records, e.g. hospital records), and all original signed Informed Consent Forms. To comply with international regulations, the Investigator should retain the records for 15 years, including assessments like mammographies.

A study initiation meeting to fully inform the investigator of his/her responsibilities and the procedures for assuring adequate and correct documentation is strongly recommended and will be organized by the Principal Investigators.

The decision to perform monitoring visits on-site lies with the Principal Investigators, who may also decide who will perform the monitoring visits. Initial monitoring on informed consent, eligibility and safety will be performed by the data managers. Any major problems identified during monitoring will be reported to the Principal Investigators. All records will be maintained in accordance with local regulations and in a manner that ensures security and confidentiality.

The investigator must assure that the subject’s anonymity will be maintained on all documents submitted to the central data managers of IKNL Clinical Trial Centre. Each subject will be identified in the eCRF by a subject identification number and months and year of birth. The subject identification number will be a sequential number. To ensure that the subject identification number is linked to the right person, the subject identification log will be kept in the Investigator Site File on site.

***b. Amendments***

Amendments are changes made to the research after a favourable opinion by the accredited METC

has been given. All amendments will be notified to the METC that gave a favourable opinion.

***c. Annual Progression Report***

The Principal Investigators will submit a summary of the progress of the trial to the accredited METC

once a year. Information will be provided on the date of inclusion of the first subject, numbers of subjects included and numbers of subjects that have completed the trial, serious adverse events, other problems and amendments.

***d. Final Report***

The Principal Investigators will notify the accredited METC of the end of the study within a period of 8

weeks. The end of the study is defined as the last patient’s last visit. In case of a preliminary end of the study, the METC will be informed within 15 days, and the reason of the ending of the study will be provided. Within one year after the end of the study, the Principal Investigators will submit a final study report with the results of the study, including any publications/abstracts of the study, to the accredited METC.

***e. Public disclosure and publication policy***

The publication guidelines of the Dutch CCMO (Central Committee for Research in Humans,

www.ccmo.nl) will be adhered to the full. These guidelines consist of a number of basic principles. First of all the results of scientific research involving human subjects must be disclosed unreservedly. All parties concerned must justify their actions in this regard. Both positive and negative research results will be disclosed and submitted to peer-reviewed scientific journals. The Principal Investigators will prepare the manuscripts together with the statistician and other active writing committee members. Co- authorship is reserved for those investigators (one per centre) that enter more than 7% of the patients, in addition to those who constructively contributed to the study at the discretion of the Principal Investigators; all other participating centres/physicians will be acknowledged.

**11. REFERENCES**

1. Jemal, A., et al., *Global cancer statistics.* CA Cancer J Clin, 2011. **61**(2): p. 69-90.
2. Ferlay, J., et al. *GLOBOCAN 2008 v2.0, Cancer Incidence and Mortality Worldwide: IARC*

*CancerBase No. 10 [Internet]*. 2010; Available from: http://globocan.iarc.fr.

1. Howlader, N., et al. *SEER Cancer Statistics Review, 1975-2010, National Cancer Institute*.

2013; Available from: http://seer.cancer.gov/csr/1975_2010/.

1. Louwman, W.J., et al., *On the rising trends of incidence and prognosis for breast cancer patients diagnosed 1975-2004: a long-term population-based study in southeastern Netherlands.*
   Cancer Causes Control, 2008. **19**(1): p. 97-106.
2. Petrek, J.A. and M.C. Heelan, *Incidence of breast carcinoma-related lymphedema.* Cancer,
   1998. **83**(12 Suppl American): p. 2776-81.
3. Krag, D.N., et al., *Sentinel-lymph-node resection compared with conventional axillary-lymph-*
   *node dissection in clinically node-negative patients with breast cancer: overall survival findings*
   *from the NSABP B-32 randomised phase 3 trial.* Lancet Oncol, 2010. **11**(10): p. 927-33.
4. Disipio, T., et al., *Incidence of unilateral arm lymphoedema after breast cancer: a systematic*
   *review and meta-analysis.* Lancet Oncol, 2013. **14**(6): p. 500-15.
5. Fisher, B., et al., *Twenty-five-year follow-up of a randomized trial comparing radical*
   *mastectomy, total mastectomy, and total mastectomy followed by irradiation.* N Engl J Med,
   2002. **347**(8): p. 567-75.
6. Giuliano, A.E., et al., *Improved axillary staging of breast cancer with sentinel lymphadenectomy.*
   Ann Surg, 1995. **222**(3): p. 394-9; discussion 399-401.
7. Voogd, A.C., et al., *The risk of nodal metastases in breast cancer patients with clinically negative*
   *lymph nodes: a population-based analysis.* Breast Cancer Res Treat, 2000. **62**(1): p. 63-9.
8. Krag, D.N., et al., *Technical outcomes of sentinel-lymph-node resection and conventional*
   *axillary-lymph-node dissection in patients with clinically node-negative breast cancer: results*
   *from the NSABP B-32 randomised phase III trial.* Lancet Oncol, 2007. **8**(10): p. 881-8.
9. Peintinger, F., et al., *Comparison of quality of life and arm complaints after axillary lymph node*
   *dissection vs sentinel lymph node biopsy in breast cancer patients.* Br J Cancer, 2003. **89**(4): p.
   648-52.
10. Schulze, T., et al., *Long-term morbidity of patients with early breast cancer after sentinel lymph*
    *node biopsy compared to axillary lymph node dissection.* J Surg Oncol, 2006. **93**(2): p. 109-19.
11. Ohsumi S, K.S., Takahashi M, Hara F, Takabatake D, Takashima S, Aogi K, Shimozuma K,
    *Sensory Disturbance of the Ipsilateral Upper Arm after Breast Cancer Surgery with Sentinel*
    *Node Biopsy Alone Compared with Axillary Dissection - A Prospective Study*, in *34th Annual*

*San Antonio Breast Cancer Symposium*. 2011: San Antonio, Texas.

1. Lucci, A., et al., *Surgical complications associated with sentinel lymph node dissection (SLND)*

*plus axillary lymph node dissection compared with SLND alone in the American College of*
*Surgeons Oncology Group Trial Z0011.* J Clin Oncol, 2007. **25**(24): p. 3657-63.

1. Ashikaga, T., et al., *Morbidity results from the NSABP B-32 trial comparing sentinel lymph node*
   *dissection versus axillary dissection.* J Surg Oncol, 2010. **102**(2): p. 111-8.
2. Nieweg, O.E., et al., *Lymphatic mapping and sentinel lymph node biopsy in breast cancer.* Eur
   J Nucl Med, 1999. **26**(4 Suppl): p. S11-6.
3. van der Ploeg, I.M., et al., *Axillary recurrence after a tumour-negative sentinel node biopsy in*
   *breast cancer patients: A systematic review and meta-analysis of the literature.* Eur J Surg
   Oncol, 2008. **34**(12): p. 1277-84.
4. Bilimoria, K.Y., et al., *Comparison of sentinel lymph node biopsy alone and completion axillary*
   *lymph node dissection for node-positive breast cancer.* J Clin Oncol, 2009. **27**(18): p. 2946-53.
5. Fant, J.S., et al., *Preliminary outcome analysis in patients with breast cancer and a positive*
   *sentinel lymph node who declined axillary dissection.* Ann Surg Oncol, 2003. **10**(2): p. 126-30.
6. Guenther, J.M., et al., *Axillary dissection is not required for all patients with breast cancer and positive sentinel nodes.* Arch Surg, 2003. **138**(1): p. 52-6.
7. Hwang, R.F., et al., *Low locoregional failure rates in selected breast cancer patients with tumor-*

*positive sentinel lymph nodes who do not undergo completion axillary dissection.* Cancer, 2007.
**110**(4): p. 723-30.

1. Jeruss, J.S., et al., *Axillary recurrence after sentinel node biopsy.* Ann Surg Oncol, 2005. **12**(1):
   p. 34-40.
2. 2Langer, I., et al., *Axillary recurrence rate in breast cancer patients with negative sentinel lymph*
   *node (SLN) or SLN micrometastases: prospective analysis of 150 patients after SLN biopsy.*
   Ann Surg, 2005. **241**(1): p. 152-8.
3. Naik, A.M., et al., *The risk of axillary relapse after sentinel lymph node biopsy for breast cancer*
   *is comparable with that of axillary lymph node dissection: a follow-up study of 4008 procedures.*
   Ann Surg, 2004. **240**(3): p. 462-8; discussion 468-71.
4. de Boer, M., et al., *Micrometastases or isolated tumor cells and the outcome of breast cancer.*
   N Engl J Med, 2009. **361**(7): p. 653-63.
5. Giuliano, A.E., et al., *Axillary dissection vs no axillary dissection in women with invasive breast*
   *cancer and sentinel node metastasis: a randomized clinical trial.* JAMA, 2011. **305**(6): p. 569-
   75.
6. Ridolfi, E., et al., *Expression and Genetic Analysis of MicroRNAs Involved in Multiple Sclerosis.*
   Int J Mol Sci, 2013. **14**(3): p. 4375-84.
7. NABON, *Mammacarcinoom, Landelijke richtlijn, Versie: 2.0.* 2012.
8. Houssami, N., et al., *Preoperative ultrasound-guided needle biopsy of axillary nodes in invasive breast cancer: meta-analysis of its accuracy and utility in staging the axilla.* Ann Surg, 2011.
   **254**(2): p. 243-51.
9. Kvistad, K.A., et al., *Axillary lymph node metastases in breast cancer: preoperative detection*
   *with dynamic contrast-enhanced MRI.* Eur Radiol, 2000. **10**(9): p. 1464-71.
10. Nori, J., et al., *Role of axillary ultrasound examination in the selection of breast cancer patients*
    *for sentinel node biopsy.* Am J Surg, 2007. **193**(1): p. 16-20.
11. Pamilo, M., M. Soiva, and E.M. Lavast, *Real-time ultrasound, axillary mammography, and*
    *clinical examination in the detection of axillary lymph node metastases in breast cancer patients.*
    J Ultrasound Med, 1989. **8**(3): p. 115-20.
12. Valente, S.A., et al., *Accuracy of Predicting Axillary Lymph Node Positivity by Physical*
    *Examination, Mammography, Ultrasonography, and Magnetic Resonance Imaging.* Ann Surg
    Oncol, 2012.
13. Rautiainen, S., et al., *Axillary lymph node biopsy in newly diagnosed invasive breast cancer:*
    *comparative accuracy of fine-needle aspiration biopsy versus core-needle biopsy.* Radiology,
    2013. **269**(1): p. 54-60.
14. Zgajnar, J., et al., *Low performance of the MSKCC nomogram in preoperatively ultrasonically*
    *negative axillary lymph node in breast cancer patients.* J Surg Oncol, 2007. **96**(7): p. 547-53.
15. Neal, C.H., et al., *Can preoperative axillary US help exclude N2 and N3 metastatic breast*
    *cancer?* Radiology, 2010. **257**(2): p. 335-41.
16. Schipper, R.J., et al., *Axillary ultrasound for preoperative nodal staging in breast cancer*
    *patients: Is it of added value?* Breast, 2013. **22**(6): p. 1108-13.
17. Early Breast Cancer Trialists' Collaborative, G., et al., *Effect of radiotherapy after breast-*
    *conserving surgery on 10-year recurrence and 15-year breast cancer death: meta-analysis of*
    *individual patient data for 10,801 women in 17 randomised trials.* Lancet, 2011. **378**(9804): p.
    1707-16.
18. Rastogi, P., et al., *Preoperative chemotherapy: updates of National Surgical Adjuvant Breast*
    *and Bowel Project Protocols B-18 and B-27.* J Clin Oncol, 2008. **26**(5): p. 778-85.
19. Straver, M.E., et al., *Towards rational axillary treatment in relation to neoadjuvant therapy in*
    *breast cancer.* Eur J Cancer, 2009. **45**(13): p. 2284-92.
20. Rouzier, R., et al., *Incidence and prognostic significance of complete axillary downstaging after*
    *primary chemotherapy in breast cancer patients with T1 to T3 tumors and cytologically proven*
    *axillary metastatic lymph nodes.* J Clin Oncol, 2002. **20**(5): p. 1304-10.
21. Alvarado, R., et al., *The role for sentinel lymph node dissection after neoadjuvant chemotherapy*
    *in patients who present with node-positive breast cancer.* Ann Surg Oncol, 2012. **19**(10): p.
    3177-84.
22. Koolen, B.B., et al., *Early assessment of axillary response with (1)(8)F-FDG PET/CT during*
    *neoadjuvant chemotherapy in stage II-III breast cancer: implications for surgical management*
    *of the axilla.* Ann Surg Oncol, 2013. **20**(7): p. 2227-35.
23. Van Roozendaal, L.M., et al., *The impact of the pathological lymph node status on adjuvant*
    *systemic treatment recommendations in clinically node negative breast cancer patients.*
    *Submitted to: Breast Cancer Res Treat.* 2013.
24. van Wely, B.J., et al., *Systematic review of the effect of external beam radiation therapy to the*
    *breast on axillary recurrence after negative sentinel lymph node biopsy.* Br J Surg, 2011. **98**(3):
    p. 326-33.
25. Reed, D.R., et al., *Axillary lymph node dose with tangential breast irradiation.* Int J Radiat Oncol
    Biol Phys, 2005. **61**(2): p. 358-64.
26. Chung, M.A., et al., *Treatment of the axilla by tangential breast radiotherapy in women with*
    *invasive breast cancer.* Am J Surg, 2002. **184**(5): p. 401-2.
27. Rabinovitch, R., et al., *Evaluation of breast sentinel lymph node coverage by standard radiation*
    *therapy fields.* Int J Radiat Oncol Biol Phys, 2008. **70**(5): p. 1468-71.
28. Maaskant-Braat, A.J., et al., *Sentinel node micrometastases in breast cancer do not affect*
    *prognosis: a population-based study.* Breast Cancer Res Treat, 2011. **127**(1): p. 195-203.
29. Schipper, R., et al., *Axillary ultrasound for preoperative nodal staging in breast cancer patients:*
    *Is it of added value?* The Breast, 2013. **Under review**.
30. Gobardhan, P.D., et al., *Prognostic value of micrometastases in sentinel lymph nodes of*
    *patients with breast carcinoma: a cohort study.* Ann Oncol, 2009. **20**(1): p. 41-8.
31. Hansen, N.M., et al., *Impact of micrometastases in the sentinel node of patients with invasive*
    *breast cancer.* J Clin Oncol, 2009. **27**(28): p. 4679-84.
32. Weaver, D.L., et al., *Effect of occult metastases on survival in node-negative breast cancer.* N
    Engl J Med, 2011. **364**(5): p. 412-21.
33. Giuliano, A.E., et al., *Association of occult metastases in sentinel lymph nodes and bone marrow*
    *with survival among women with early-stage invasive breast cancer.* JAMA, 2011. **306**(4): p.
    385-93.
34. Koelliker, S.L., et al., *Axillary lymph nodes: US-guided fine-needle aspiration for initial staging*
    *of breast cancer--correlation with primary tumor size.* Radiology, 2008. **246**(1): p. 81-9.
35. Abe, H., et al., *Axillary lymph nodes suspicious for breast cancer metastasis: sampling with US-*
    *guided 14-gauge core-needle biopsy--clinical experience in 100 patients.* Radiology, 2009.
    **250**(1): p. 41-9.
36. Nielsen, M.H., et al., *Delineation of target volumes and organs at risk in adjuvant radiotherapy*
    *of early breast cancer: national guidelines and contouring atlas by the Danish Breast Cancer*
    *Cooperative Group.* Acta Oncol, 2013. **52**(4): p. 703-10.
37. van der Steeg, A.F., et al., *Personality predicts quality of life six months after the diagnosis and*
    *treatment of breast disease.* Ann Surg Oncol, 2007. **14**(2): p. 678-85.
38. Aaronson, N.K., et al., *The European Organization for Research and Treatment of Cancer QLQ-*
    *C30: a quality-of-life instrument for use in international clinical trials in oncology.* J Natl Cancer
    Inst, 1993. **85**(5): p. 365-76.
39. Sprangers, M.A., et al., *The European Organization for Research and Treatment of Cancer*
    *breast cancer-specific quality-of-life questionnaire module: first results from a three-country field*
    *study.* J Clin Oncol, 1996. **14**(10): p. 2756-68.
40. Devoogdt, N., et al., *Lymphoedema Functioning, Disability and Health questionnaire (Lymph-*
    *ICF): reliability and validity.* Phys Ther, 2011. **91**(6): p. 944-57.
41. van der Bij, A.K., et al., *Validation of the dutch short form of the state scale of the Spielberger*
    *State-Trait Anxiety Inventory: considerations for usage in screening outcomes.* Community
    Genet, 2003. **6**(2): p. 84-7.
42. M.J., I., *Richtlijn voor het uitvoeren van economische evaluaties in de gezondheidszorg.* 2016.
43. Roijen, L.H.-v., S.S. Tan, and C.A.M. Bouwmans, *Handleiding voor kostenonderzoek: methoden en standaard kostprijzen voor economische evaluaties in de gezondheidszorg*. 2010.
44. Barber, J.A. and S.G. Thompson, *Analysis of cost data in randomized trials: an application of the non-parametric bootstrap.* Stat Med, 2000. **19**(23): p. 3219-36.
45. Sullivan, S.D., et al., *Budget impact analysis-principles of good practice: report of the ISPOR 2012 Budget Impact Analysis Good Practice II Task Force.* Value Health, 2014. **17**(1): p. 5-14.
46. Goren, A., et al., *Quantifying the burden of informal caregiving for patients with cancer in Europe.* Support Care Cancer, 2014. **22**(6): p. 1637-46.
47. Kim, Y. and B.A. Given, *Quality of life of family caregivers of cancer survivors: across the trajectory of the illness.* Cancer, 2008. **112**(11 Suppl): p. 2556-68.
48. Merckaert, I., et al., *Desire for formal psychological support among caregivers of patients with cancer: prevalence and implications for screening their needs.* Psychooncology, 2013. **22**(6): p.
    1389-95.
49. Wadhwa, D., et al., *Quality of life and mental health in caregivers of outpatients with advanced*
    *cancer.* Psychooncology, 2013. **22**(2): p. 403-10.
50. Yabroff, K.R. and Y. Kim, *Time costs associated with informal caregiving for cancer survivors.*
    Cancer, 2009. **115**(18 Suppl): p. 4362-73.
51. Herdman, M., et al., *Development and preliminary testing of the new five-level version of EQ-*
    *5D (EQ-5D-5L).* Qual Life Res, 2011. **20**(10): p. 1727-36.
52. Brouwer, W.B., et al., *The CarerQol instrument: a new instrument to measure care-related*
    *quality of life of informal caregivers for use in economic evaluations.* Qual Life Res, 2006. **15**(6):
    p. 1005-21.
53. Robinson, B.C., *Validation of a Caregiver Strain Index.* J Gerontol, 1983. **38**(3): p. 344-8.
54. Cardol, M., et al., *The development of a handicap assessment questionnaire: the Impact on Participation and Autonomy (IPA).* Clin Rehabil, 1999. **13**(5): p. 411-9.

**12. ANNEX**

The following attachments are enclosed:

- A0: Dutch Summary

- E1: Patient Information Folder

- E2: Consent Form

- F1: Quality of Life questionnaires

- K6: TNM Classification 7^th^ edition

- K6: TRIAS brochure
